# Aberrant epithelial cell interaction promotes esophageal squamous-cell carcinoma development and progression

**DOI:** 10.1038/s41392-023-01710-2

**Published:** 2023-12-15

**Authors:** Liping Chen, Shihao Zhu, Tianyuan Liu, Xuan Zhao, Tao Xiang, Xiao Hu, Chen Wu, Dongxin Lin

**Affiliations:** 1 Department of Etiology and Carcinogenesis, National Cancer Center/National Clinical Research Center for Cancer/Cancer Hospital, Chinese Academy of Medical Sciences and Peking Union Medical College, Beijing, 100021 China; 2 Key Laboratory of Cancer Genomic Biology, Chinese Academy of Medical Sciences and Peking Union Medical College, Beijing, 100021 China; 3 Collaborative Innovation Center for Cancer Personalized Medicine, Nanjing Medical University, Nanjing, 211166 China; 4 CAMS Oxford Institute, Chinese Academy of Medical Sciences, Beijing, 100006 China; 5 Sun Yat-sen University Cancer Center, State Key Laboratory of Oncology in South China, Guangzhou, 510060 China

**Keywords:** Gastrointestinal cancer, Oncogenes

## Abstract

Epithelial-mesenchymal transition (EMT) and proliferation play important roles in epithelial cancer formation and progression, but what molecules and how they trigger EMT is largely unknown. Here we performed spatial transcriptomic and functional analyses on samples of multistage esophageal squamous-cell carcinoma (ESCC) from mice and humans to decipher these critical issues. By investigating spatiotemporal gene expression patterns and cell–cell interactions, we demonstrated that the aberrant epithelial cell interaction via EFNB1-EPHB4 triggers EMT and cell cycle mediated by downstream SRC/ERK/AKT signaling. The aberrant epithelial cell interaction occurs within the basal layer at early precancerous lesions, which expands to the whole epithelial layer and strengthens along the cancer development and progression. Functional analysis revealed that the aberrant EFNB1-EPHB4 interaction is caused by overexpressed ΔNP63 due to TP53 mutation, the culprit in human ESCC tumorigenesis. Our results shed new light on the role of TP53-TP63/ΔNP63-EFNB1-EPHB4 axis in EMT and cell proliferation in epithelial cancer formation.

## Introduction

Esophageal cancer, one of the most prevalent gastrointestinal malignancies worldwide, consists of two histological types known as esophageal adenocarcinoma and squamous-cell carcinoma (ESCC). It is well known that ESCC development experiences chronic inflammation (INF), precancerous lesions including low-grade intraepithelial neoplasia (LGIN) and high-grade intraepithelial neoplasia (HGIN), and invasive ESCC.^1,2^ A previous prospective study has reported that there were 31% of LGIN cases and 74% of HGIN cases developed into ESCC, and most HGIN cases progressed into ESCC within 3 years after diagnosis.^3^ However, the molecular mechanisms underlying the transformation from normal to malignant phenotypes remain largely unknown, which impedes the recognition of precancerous lesions that may eventually progress into ESCC. Since ESCC prognosis is largely dependent on tumor stage and early staged diseases undergoing endoscopic or surgical therapy have relatively high 5-year survival rates,^4,5^ understanding of how precancerous lesions evolute to invasive ESCC is of fundamental significance for early detection, diagnosis and treatment.

Single-cell transcriptomic analysis is a powerful technique and has been applied to decipher the aberrant gene expression in epithelial cells and the cellular compositions in the microenvironment of different disease stages. For instance, we have deciphered the cell transition states in a multistep ESCC progression model in mice that mimics human ESCC development and identified a set of key transitional signatures associated with the oncogenic evolution of epithelial cells.^6^ More importantly, we have found that some key changes in this mouse model also occur in human esophageal tissue samples.^7^ Recently, we have performed another study using single cell and spatial transcriptomic analyses of multistage esophageal lesions and revealed that in the microenvironment, epithelial cells can activate fibroblasts to promote ESCC development. This effect is mediated by suppressed ANXA1-FPR2 ligand-receptor interaction due to the gradual loss of ANXA1 expression in epithelial cells during ESCC development.^8^ These previous studies have provided paradigms for discovering the critical molecular events that promote the progression of precancerous lesions to cancerous lesions.

It has been well recognized that during the development of ESCC, the epithelial tissue structure undergoes destructive changes. At LGIN stage, epithelial cells loss cell polarity and dysplastic cells show distinct boundaries from non-dysplastic cells; at HGIN stage, proliferative dysplastic cells break through the basement membrane; and at the invasive cancer stage, malignant cells infiltrate submucosa and lamina propria.^9–11^ However, despite having recognized the gradual histopathological changes for a long time, the exact in situ molecular mechanism and intercellular signaling that drive proliferation and invasion of diseased epithelial cells are not elucidated. Recently developed spatial transcriptomic analytical technology^12–14^ that combines in situ gene expression with tissue spatial imaging has provided us a revolutionized method for analyzing the tissue destruction-related molecular alterations during ESCC progression.

In this study, by using spatial and single-cell transcriptomic analysis, we have deciphered the spatial gene expression patterns and cellular communication networks in normal and multistage diseased esophageal tissue samples from mice receiving 4-nitroquinoline-1-oxide (4-NQO) and patients with ESCC.^6–8^ We have spatially identified an ESCC development region with significant expression of tumor hallmarks, outgrowing from the basal layer of the epithelium in diseased esophageal tissue samples. The progression of ESCC may be caused by aberrant ligand-receptor interaction of EFNB1-EPHB4 among epithelial cells. Functional experiments have demonstrated that aberrant EFNB1-EPHB4 interaction in epithelial cells accelerates ESCC progression via enhanced cell cycle and epithelial-mesenchymal transition (EMT) pathways both activated by SRC/ERK/AKT signaling. Furthermore, we have discovered for the first time that ΔNP63, overexpressed in precancerous and cancerous lesions of the esophagus due to TP53 dysfunction, is a transcription factor of both *EFNB1* and *EPHB4* genes. Thus, our results shed light on a role of the TP53-ΔNP63-EFNB1-EPHB4-EMT/cell cycle axis in oncogenic destruction of epithelial tissues during ESCC progression.

## Results

### Aberrant epithelial cell interaction via EFNB1-EPHB4 plays a role in mouse ESCC formation

**Fig. 1:**
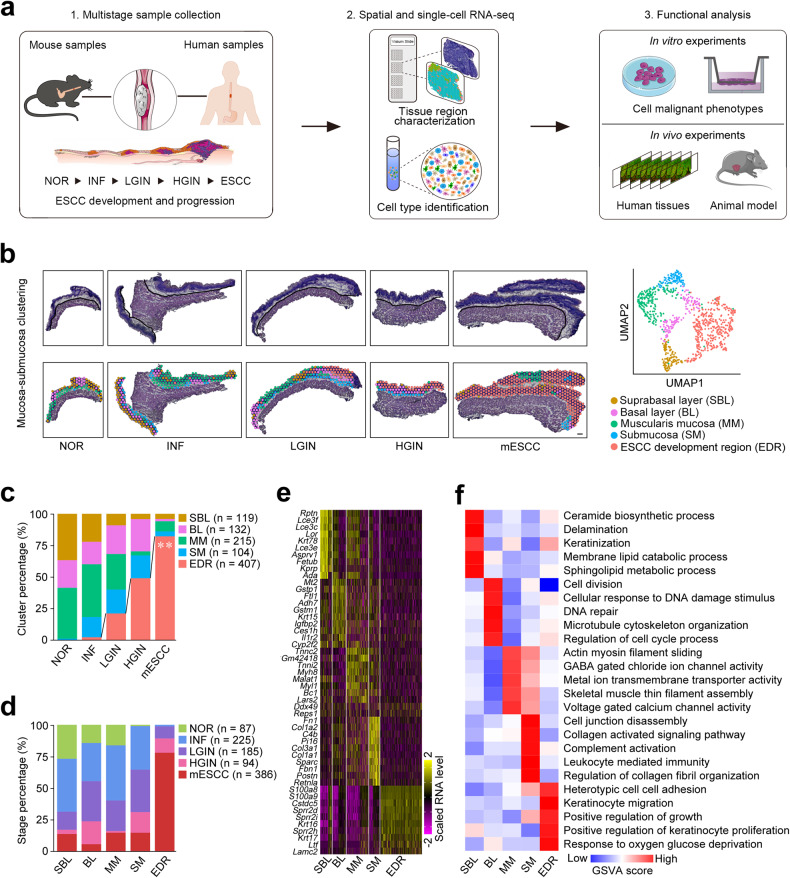
Spatial transcriptomic analysis reveals distinct molecular regionalization in mESCC development and progression. **a** Scheme of the overall study design. **b** Graph-based clustering of mucosa-submucosa in five stage samples identified five tissue regions. Upper left: H&E staining of 5 stage samples with mucosa-submucosa and muscularis delineated by black lines. Lower left: spatial distribution of five tissue regions in each sample. Scale bar, 200 μm. Right: UMAP plot showing unbiased clustering of mucosa-submucosa spots in all five stage samples. NOR normal, INF inflammation, LGIN low-grade intraepithelial neoplasia, HGIN high-grade intraepithelial neoplasia, mESCC mouse esophageal squamous-cell carcinoma. **c** Stacked histogram showing the composition of five tissue regions across five stage samples. The proportion of EDR increased gradually during five stages. **P < 0.01 of Mann–Kendal trend test for different stages. **d** Stacked histogram showing the composition of five stages in five tissue regions. **e** Heatmap of scaled and normalized expression levels of the top 10 highly expressed genes in five tissue regions. **f** Heatmap of scaled and normalized gene set enrichment analysis (GSVA) scores for selected Gene Ontology pathways in five tissue regions. Also see Supplementary Fig. S1 and Supplementary Tables S1 and S2

By Visium spatial transcriptomic sequencing, we analyzed five distinct tissues, i.e., normal (NOR), INF, LGIN, HGIN and ESCC, taken at different time from a mouse model treated with chemical carcinogen 4-NQO^15^ (Fig. 1a and Supplementary Fig. 1a). The initial spatial transcriptomic data consisted of 2022 spots covering mucosa-submucosa and muscularis (Supplementary Fig. 1b and Supplementary Table S1). Since mouse ESCC (mESCC) developed in epithelium, we manually isolated 977 spots in mucosa and submucosa for final analysis and these included 87 from NOR, 225 from INF, 185 from LGIN, 94 from HGIN and 386 from mESCC samples. Each spot had an average of 3849 genes and 10,037 unique transcripts after quality control (Supplementary Fig. 1c). Clustering analysis designated four transcriptome regions histologically as suprabasal layer (SBL, *n* = 119), basal layer (BL, *n* = 132), muscularis mucosa (MM, *n* = 215) and submucosa (SM, *n* = 104) in NOR and four diseased esophageal samples (Fig. 1b and Supplementary Fig. 1d). We identified another special cluster with high levels of cancer hallmark expression named as mESCC development region (EDR, *n* = 407) that appeared in INF stage, gradually enlarged in precancerous lesions and finally made up to 82.1% of ESCC (trend test, *P* = 0.007; Fig. 1b–d). The four canonical tissue regions substantially expressed genes corresponding to their distributions and functions, whereas EDR exhibited high levels of genes involved in keratinocyte proliferation, migration, and invasion (Fig. 1e, f and Supplementary Table S2). These results indicate disorganization of epithelial structure during the development of mESCC.

**Fig. 2:**
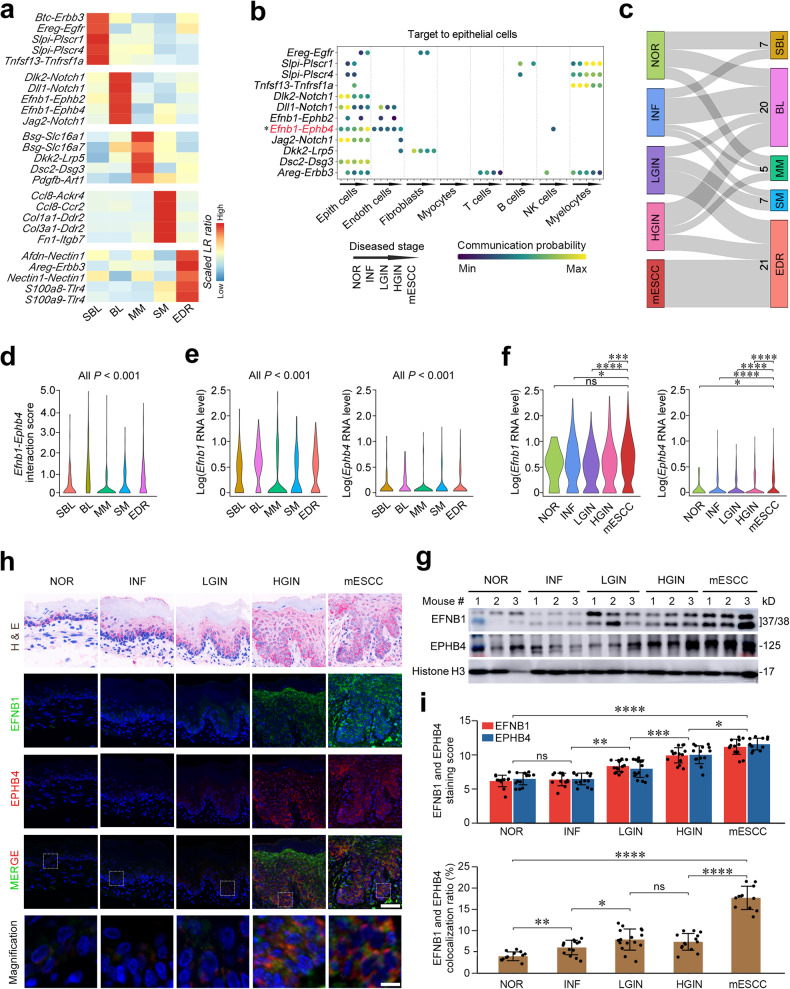
Aberrant epithelial cell interaction via EFNB1-EPHB4 plays a role in mESCC formation. **a** Heatmap of proportion of tissue region-specific ligand-receptor (LR) gene pairs enriched in five tissue regions. **b** Bubble plot showing interaction of tissue region-specific LR gene pairs between eight cell types and epithelial cells in single-cell transcriptome data from five stage tissues (CRA002118). Each dot represented is significant (*P* < 0.05). *Efnb1*-*Ephb4* interaction probability among epithelial cells is gradually and significantly elevated along mESCC progression. *P < 0.05 of Mann–Kendal trend test for different stages. **c** Sankey plot showing the distribution of spots with significant *Efnb1*-*Ephb4* interaction in five tissue regions of five stage samples. **d** Violin plots of *Efnb1*-*Ephb4* interaction score in five tissue regions. **e** Violin plots of log-normalized RNA levels of *Efnb1* (left panel) and *Ephb4* (right panel) in five tissue regions. BL and EDR had higher expression levels of *Efnb1* and *Ephb4* than the other three tissue regions. **f** Violin plots of log-normalized RNA levels of *Efnb1* (left panel) and *Ephb4* (right panel) in single-cell transcriptome data of epithelial cells in five stage samples (CRA002118). *P* for Wilcoxon rank-sum test in (**d**–**f**); *P < 0.05; ***P < 0.001; ****P < 0.0001; ns not significant. **g** Western blot of EFNB1 and EPHB4 in mouse esophageal tissues of different disease stages. Each stage had three samples and each blot assay had three biological repeats. **h** Representative immunofluorescence images of EFNB1 and EPHB4 in five disease stages showing that they were highly overexpressed in the basal layer of NOR and INF, which expanded to the whole layer along precancerous to cancerous progression. Scale bar on upper panel, 50 μm; scale bar on lower panel, 5 μm. **i** Upper panel: quantitative statistics of EFNB1 and EPHB4 staining scores in NOR (*n* = 13), INF (*n* = 14), LGIN (*n* = 16), HGIN (*n* = 14), and mESCC (*n* = 12). Data are mean ± SEM. Lower panel: quantitative statistics of EFNB1 and EPHB4 colocalization ratios in NOR (*n* = 13), INF (*n* = 14), LGIN (*n* = 16), HGIN (*n* = 14), and mESCC (*n* = 12). Data are mean ± SEM. Wilcoxon rank-sum test, **P < 0.01; ***P < 0.001; ****P < 0.0001 and ns not significant. NOR normal, INF inflammation, LGIN low-grade intraepithelial neoplasia, HGIN high-grade intraepithelial neoplasia, mESCC mouse esophageal squamous-cell carcinoma. Also see Supplementary Figs. S2 and S3 and Supplementary Table S3

We next examined the cell–cell interactions in the microenvironments of precancerous and cancerous lesions to find out their effects on epithelial disorganization. We computed the ratio of ligand-receptor gene pairs and found that different tissue regions had distinct and specific intercellular signal transductions. Notably, EDR had high ratios of ligand-receptor gene pairs in the cell growth, cell adhesion and tumor inflammation pathways (Fig. 2a and Supplementary Table S3). We further examined the cell types coordinating these interactions in the single-cell transcriptome dataset of the multistage mouse model^6^ (Fig. 2b and Supplementary Fig. 2a), and the results showed that the interaction probability of *Notch1*-related ligand-receptor gene pairs, *Dlk2*-*Notch1*, *Dll1*-*Notch1* and *Jag2*-*Notch1*, among epithelial cells in the basal layer was significantly decreased along the disease progression; however, the interaction probability of *Efnb1*-*Ephb4*, the members of the Ephrin/Eph tyrosine kinase receptor family, was significantly increased (trend test, *P* = 0.014; Fig. 2b).

Spatial transcriptomic analysis showed 60 spots with significant *Efnb1*-*Ephb4* interaction that were mainly located in the BL (20 spots) and EDR (21 spots) regions (Fig. 2c), which had the *Efnb1*-*Ephb4* interaction scores significantly higher than that of the spots in other three tissue regions (Fig. 2d and Supplementary Fig. 3a). In line with these results, the *Efnb1* and *Ephb4* RNA levels were significantly higher in BL and EDR than that in other regions (Fig. 2e and Supplementary Fig. 3b, c). Single-cell transcriptomic analysis also showed that in normal and all four disease stages, *Efnb1* was highly expressed in epithelial cells while *Ephb4* was significantly overexpressed in precancerous and cancerous epithelial cells (Fig. 2f and Supplementary Fig. 2b), which was confirmed by their protein levels in tissue samples of different disease stages (Fig. 2g). Immunofluorescence staining of the mouse tissue samples showed that EFNB1 and EPHB4 were colocalized on the membrane of epithelial cells in the basal layer and the interaction signals were significantly and gradually increased along the disease progression (Fig. 2h, i). These results suggest that the aberrant interaction of EFNB1 and EPHB4 may play an important role in 4-NQO-induced mESCC development.

### Aberrant epithelial cell interaction via EFNB1-EPHB4 promotes human ESCC formation

**Fig. 3:**
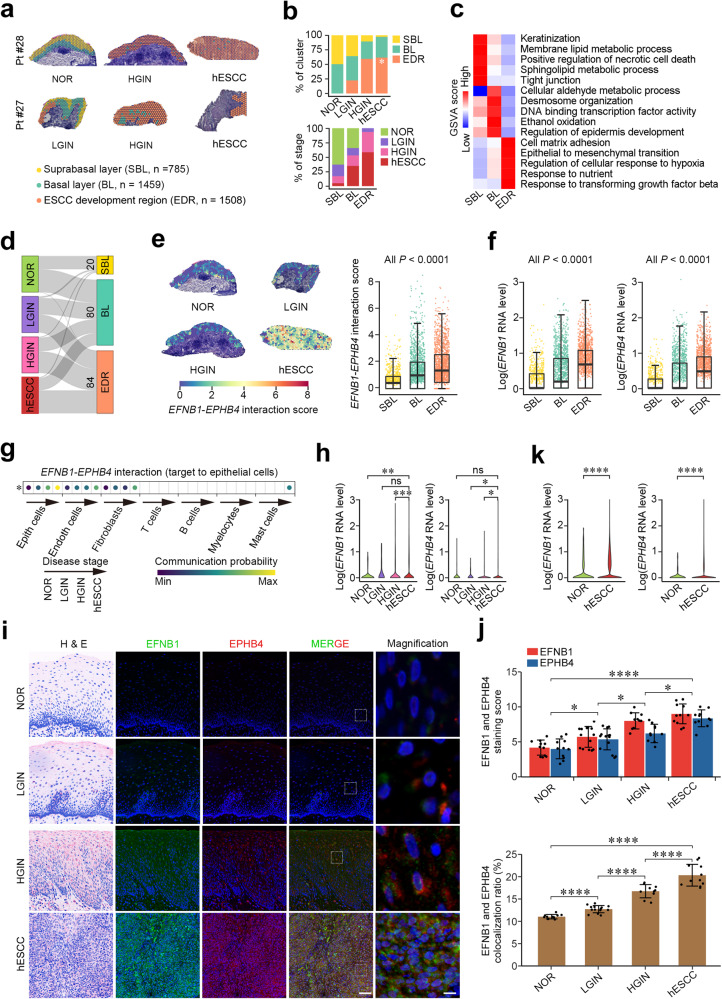
Aberrant epithelial cell interaction via EFNB1-EPHB4 promotes hESCC formation. **a** Spatial plots showing distribution of three tissue regions in four stage samples from ESCC patients. Scale bar, 200 μm. Pt patient. **b** Stacked histogram showing the composition of three tissue regions across four disease stages (upper panel) and four stages across three tissue regions (lower panel). The proportion of SBL decreased gradually and significantly but the proportion of EDR increased gradually and significantly along the disease progression. *P < 0.05 of Mann–Kendal trend test. **c** Heatmap of scaled and normalized gene set enrichment analysis (GSVA) scores for selected Gene Ontology pathways in three tissue regions. **d** Sankey plot showing the distribution of spots with significant *EFNB1*-*EPHB4* interaction in three tissue regions of four pathological stages. **e** *EFNB1*-*EPHB4* interaction score in three tissue regions of four stages. Left panel: spatial plots showing that *EFNB1-EPHB4* interaction score is higher in the basal layer of NOR, which expands to the whole layer along LGIN, HGIN, and hESCC. Scale bar, 200 μm. Right panel: boxplot of *EFNB1*-*EPHB4* interaction score in three tissue regions shown is median and 25th to 75th percentile distribution with 1.5× quantile range represented by whiskers and the difference was tested by Wilcoxon rank-sum test. **f** Boxplots of log-normalized *EFNB1* and *EPHB4* RNA levels in three tissue regions. Data are median and 25th to 75th percentile distribution with 1.5× quantile range represented by whiskers. *P* value for Wilcoxon rank-sum test. **g** Bubble plot of *EFNB1*-*EPHB4* interaction between seven cell types and epithelial cells in multistage single-cell transcriptome data (HRA000776). Each dot represented is significant (*P* < 0.05). *EFNB1*-*EPHB4* interaction probability between epithelial cells elevates gradually during four pathological stages. *P < 0.05 of Mann–Kendal trend test. **h** Violin plots showing log-normalized *EFNB1* and *EPHB4* RNA levels in epithelial cells of different stage samples by scRNA-seq (HRA000776). **i** Representative immunofluorescence images of EFNB1 and EPHB4 in four pathological stages showing they are highly expressed in the basal layer of NOR, which expand to the whole layer as ESCC developed. Scale bar on left panel, 50 μm and on right panel, 5 μm. **j** Upper panel: quantitative statistics of EFNB1 and EPHB4 staining scores in NOR (*n* = 11), LGIN (*n* = 14), HGIN (*n* = 10), and hESCC (*n* = 11). Data are mean ± SEM. Lower panel: quantitative statistics of EFNB1 and EPHB4 colocalization ratios in NOR (*n* = 11), LGIN (*n* = 14), HGIN (*n* = 10), and hESCC (*n* = 11). Data are mean ± SEM. *P < 0.05; **P < 0.01; ****P < 0.0001; and ns not significant of Wilcoxon rank-sum test. **k** Violin plots showing log-normalized *EFNB1* and *EPHB4* RNA levels in epithelial cells of hESCC and normal tissues by scRNA-seq (GSE160269). The significant differences were examined by Wilcoxon rank-sum test. *P < 0.05; **P < 0.01; ***P < 0.001; ****P < 0.0001; and ns not significant. NOR normal, LGIN low-grade intraepithelial neoplasia, HGIN high-grade intraepithelial neoplasia, hESCC human esophageal squamous-cell carcinoma. Also see Supplementary Fig. S4 and Supplementary Table S4

The results in the mouse model encouraged us to examine whether the aberrant EFNB1-EPHB4 interaction also exists and plays a role in human esophageal tumorigenesis. Using the similar experimental approaches as used in the mouse model, we manually separated 3752 spots of esophageal mucosa-submucosa of human ESCC (hESCC) from 4 patients, including 994 from NOR, 441 from LGIN, 888 from HGIN and 1429 from hESCC for spatial transcriptomic analysis (Supplementary Fig. 4a and Supplementary Table S4). After quality control, each spot had an average of 4,485 genes and 13,119 unique transcripts for final analysis (Supplementary Fig. 4b), which were derived from SBL (*n* = 785), BL (*n* = 1459) and EDR (*n* = 1508), respectively (Fig. 3a and Supplementary Fig. 4c). We found that the proportions of EDR were increased along the disease progression from LGIN, HGIN to hESCC (trend test, *P* = 0.02; Fig. 3b and Supplementary Fig. 4d), indicating similar epithelial disorganization occurred in hESCC development that was seen in mESCC model. We found that EDR in human esophageal tissues had extensively high expression of genes involving in cell matrix adhesion, cellular response to TGF-β and EMT although SBL and BL had high expression of genes in epithelial keratinization, cell necrosis and tight junction and oxidation metabolism, desmosome formation and transcription factor activity, respectively (Fig. 3c and Supplementary Fig. 4e). Furthermore, we found 184 spots with significantly high *EFNB1*-*EPHB4* interaction predominately within BL (80 spots) and EDR (84 spots) that had significantly higher interaction scores than that of spots in other regions (Fig. 3d, e). It is worth noting that the basal layers of NOR, LGIN and HGIN had higher *EFNB1*-*EPHB4* interaction scores than SBL while in the hESCC region, the interaction signals were more extensive and suffused (Fig. 3e). In line with the score levels, BL and EDR had significantly higher *EFNB1* and *EPHB4* RNA levels than SBL (Fig. 3f and Supplementary Fig. 4f). Single-cell transcriptomic analysis showed that the *EFNB1*-*EPHB4* interaction among epithelial cells was gradually enhanced along hESCC progression (trend test, *P* = 0.04; Fig. 3g) and the expression of *EFNB1* and *EPHB4* in epithelial cells were upregulated (Fig. 3h and Supplementary Fig. 4g). Immunofluorescence staining also validated the spatiotemporal alterations of EFNB1-EPHB4 interaction showing that high interaction scores were in the BL of NOR samples and were increased and spread in LGIN, HGIN, and hESCC (Fig. 3i, j). These results were verified in the single-cell transcriptomic data in hESCC samples from 60 patients (Fig. 3k and Supplementary Fig. 4g, h) and the bulk hESCC RNA sequencing data from 94 patients (Supplementary Fig. 4i, j) as reported previously.^7,16^ These results indicate that human esophageal cancer development has a similar molecular tissue segmentation to mESCC tumorigenic model and the aberrant EFNB1-EPHB4 interaction may play an important role in epithelial tissue oncogenic disorganization.

### Aberrant EFNB1-EPHB4 interaction triggers epithelial-mesenchymal transition and proliferation

**Fig. 4:**
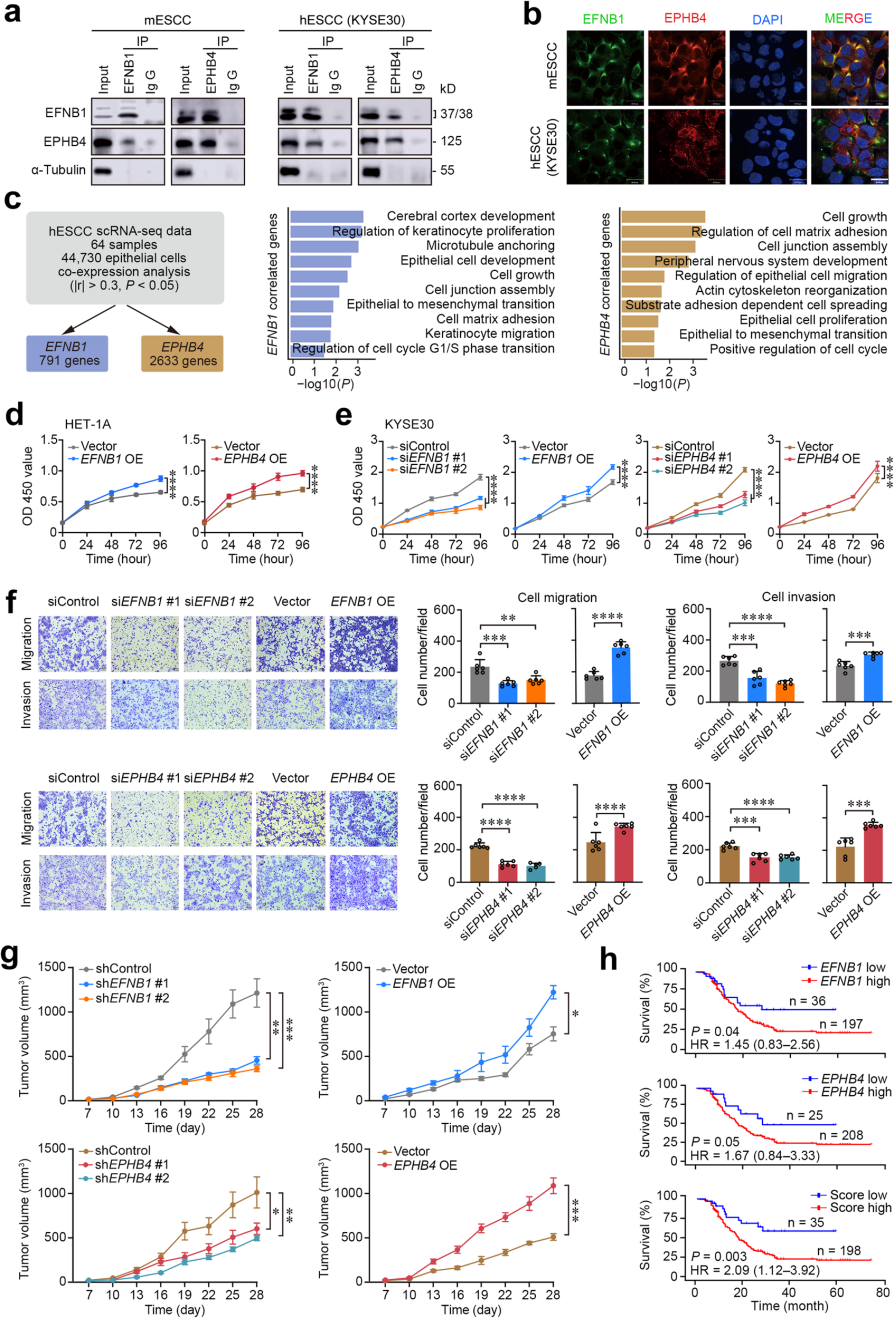
Aberrant EFNB1-EPHB4 interaction enhances the malignant phenotypes of mouse and human ESCC cells. **a** Western blotting of immunoprecipitation products with anti-EFNB1 or anti-EPHB4 antibody in mESCC and hESCC cells (KYSE30) showing interaction between these two proteins. **b** Immunofluorescent staining showing colocalization of EFNB1 and EPHB4 on epithelial cell membrane in mESCC and hESCC cells. The nuclei were stained with DAPI. Scale bars, 30 μm. **c** Analysis of genes their expression levels were correlated with *EFNB1* and *EPHB4* expression levels in epithelial cells by scRNA-seq (GSE160269 data). Left panel shows the analytical scheme, and the middle and right panels show bar plots of selected terms from GO enrichment results of genes correlated with *EFNB1* or *EPHB4*. **d** The effects of overexpressed *EFNB1* or *EPHB4* on cell proliferation of human immortalized esophageal epithelial cell line HET-1A. Each point represents mean ± SEM obtained from three independent experiments and each had six replications. ****P < 0.0001 of Student’s *t*-test. **e** The effects of forced *EFNB1* or *EPHB4* expression change on hESCC (KYSE30) cell proliferation. Each point represents mean ± SEM obtained from three independent experiments and each had six replications. ****P < 0.0001 of Student’s *t*-test. **f** The effec*t*s of forced *EFNB1* and *EPHB4* expression change on hESCC (KYSE30) cell migration and invasion. Left panel shows representative transwell images and the right panel shows quantitation statistics. Data are mean ± SEM from three independent experiments and each had two replications. Scale bar, 100 μm. **P < 0.01; ***P < 0.001; and ****P < 0.0001 of Wilcoxon rank-sum test. **g** The effects of forced *EFNB1* and *EPHB4* expression change in hESCC cells (KYSE30) on their xenograft growth in NSG mice. Tumor volumes are defined as length × width^2^ × 0.52. Data are mean ± SEM from five animals. *P < 0.05; **P < 0.01; and ***P < 0.001 of Student’s *t*-test. **h** Kaplan–Meier estimate of survival time in 233 ESCC patients by *EFNB1*, *EPHB4* or *EFNB1* and *EPHB4* RNA levels. Hazard ratio (HR) and 95% confidence interval (CI) were computed by multivariate Cox proportional hazard models with age, sex, and tumor stage as covariates. Also see Supplementary Figs. S5 and S6 and Supplementary Table S5

We next performed reciprocal co-immunoprecipitation assays using primary mESCC cells and hESCC cells (KYSE30) to examine the physical interaction between EFNB1 and EPHB4 and the results clearly demonstrated an interaction of these two proteins (Fig. 4a). Further in vitro immunofluorescence assays indicated that the interaction was located on the cell membrane (Fig. 4b). We then sought to know what happens in epithelial cells when this pair of ligand-receptor interacts with each other. We applied gene expression correlation analysis in our previously reported hESCC single-transcriptomic dataset containing 44,730 epithelial cells^7^ to explore genes their expression levels are correlated with EFNB1 and EPHB4 interaction. We found that 791 and 2633 genes had their mRNA levels that were significantly correlated with *EFNB1* and *EPHB4* RNA levels, respectively, and they were mainly enriched in the pathways related to epithelial cell proliferation, migration, cell cycle regulation, cell junction assembly and epithelial-to-mesenchymal transition (EMT) (Fig. 4c and Supplementary Table S5). We thus performed gene operation in mESCC cells, human immortalized normal esophageal epithelial cells (HET-1A) and hESCC cells (KYSE30 and KYSE150) to examine the effects of *EFNB1* and *EPHB4* on cell malignant phenotypes and found that knocking down either *EFNB1* or *EPHB4* in cancer cells significantly suppressed cell proliferation but ectopic overexpressing *EFNB1* or *EPHB4* had opposite effects in both normal and cancer cells (Fig. 4d, e and Supplementary Figs. 5a, b and 6a). Knocking down *EFNB1* or *EPHB4* also significantly inhibited cancer cell migration and invasion but their overexpressing had the opposite effects in both normal and tumor cells (Fig. 4f and Supplementary Figs. 5c, d and 6b). *EFNB1* and *EPHB4* stable knockdown or overexpression (Supplementary Fig. 6c) also significantly altered the growth rates of KYSE30 cells transplanted in mice (Fig. 4g and Supplementary Fig. 6d, e). IHC staining verified depletion or overexpression of EFNB1 and EPHB1 protein in the transplanted mouse xenografts (Supplementary Fig. 6f). We further performed survival analysis in our previously reported ESCC patient cohort (*n* = 233; Supplementary Table S6)^7^ using threshold with the minimum log-rank *P* value (see Methods). The results showed that patients with high RNA level of *EFNB1* (≥10.6 TPM) or *EPHB4* (≥9.2 TPM) in ESCC had shorter survival time than those with low RNA level of *EFNB1* (<10.6 TPM) or *EPHB4* (<9.2 TPM) although the statistical significance were marginally (*P* = 0.04 and *P* = 0.05 for log-rank test; Fig. 4h). However, patients with high score of both *EFNB1* and *EPBH4* levels (≥11.3 TPM) had significantly worse prognosis than those with low score of both *EFNB1* and *EPBH4* level (<11.3 TPM; log-rank *P* < 0.003) and the hazard ratio for death adjusted for sex, age, and tumor stage was 2.09 (95% CI = 1.12–3.92; Fig. 4h). These in vitro and in vivo results indicate that EFNB1 and EPHB4 play important roles in ESCC development and progression probably via enhancing malignant EMT and proliferation.

**Fig. 5:**
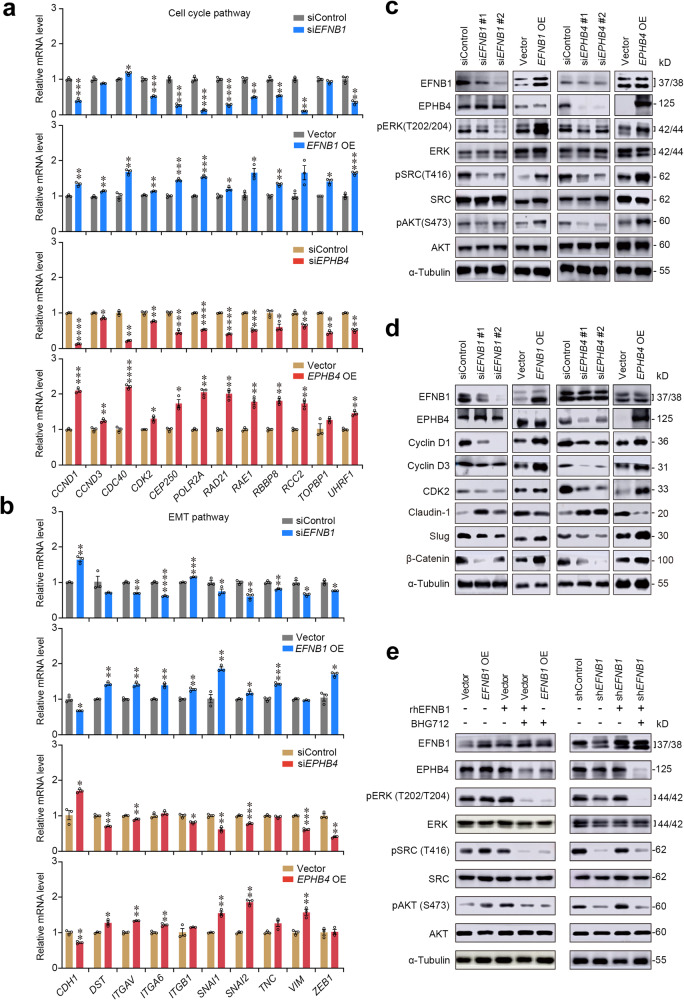
Aberrant EFNB1-EPHB4 interaction triggers epithelial-to-mesenchymal transition and cell proliferation. **a**, **b** Qualitative PCR verification of the effects of *EFNB1* (upper panel) and *EPHB4* (lower panel) knockdown or overexpression (OE) on the expression alterations of genes involved in cell cycle pathway and epithelial-to-mesenchymal transition (EMT) pathway in hESCC cells (KYSE30). Data are mean ± SEM from three independent experiments. *P < 0.05; **P < 0.01; ***P < 0.001; ****P < 0.0001; and ns not significant of Wilcoxon rank-sum test. **c** Western blotting analysis of total and phosphorylated levels of ERK, SRC and AKT proteins in hESCC cells (KYSE30) with *EFNB1* or *EPHB4* knockdown or OE. Each western blot had three biological repeats. **d** Western blotting analysis of cell cycle markers (Cyclin D1, Cyclin D3, and CDK2) and EMT markers (Claudin-1, SLUG, and β-Catenin) in hESCC cells (KYSE30) with *EFNB1* or *EPHB4* knockdown or OE. Each western blot had three biological repeats. **e** Western blotting analysis of total and phosphorylated levels of ERK, SRC and AKT in hESCC cells (KYSE30) treated with recombinant human EFNB1 or EPHB4 inhibitor. Each western blot had three biological repeats. Also see Supplementary Fig. S7

The above results led us to look at cell cycle and morphology of cells with forced *EFNB1* or *EPHB4* expression changes. We found that overexpression of *EFNB1* or *EPHB4* significantly accelerated but knockdown of *EFNB1* or *EPHB4* significantly inhibited cell cycle of hESCC cell KYSE30 at S and G0/G1 phases (Supplementary Fig. 7a). *EFNB1* or *EPHB4* overexpressing KYSE30 cells also showed morphological change, less cohesive among cells, and higher expression of EMT marker VIM as compared with control cells while knocking down of *EFNB1* or *EPHB4* had opposite effect on cells (Supplementary Fig. 7b). In addition, we found that the EMT scores in epithelial cells of both mice and humans were gradually increased as ESCC developed (Supplementary Fig. 7c). We then looked at the expression levels of genes involved in the cell cycle regulation and EMT process in cells with forced *EFNB1* or *EPHB4* expression change and found that *EFNB1* or *EPHB4* knockdown significantly decreased whereas *EFNB1* or *EPHB4* overexpression significantly increased the levels of the 12 genes such as *CCND1*, *CCND3*, *CDC40*, and *CDK2* in cell cycle regulation and 10 genes such as *DST*, *ITGAV*, *SNAl1*, *SNAI2*, and *VIM* in EMT pathway (Fig. 5a, b). It has been known that cell cycle and EMT of epithelial cells may be regulated by SRC/ERK/AKT signaling pathway^17,18^ and EPHB4 can activate these signaling proteins via receptor-mediated phosphorylation,^19,20^ we thus specifically examined the effects of forced *EFNB1* or *EPHB4* expression changes on the phosphorylation of SRC, ERK and AKT in KYSE30 cells. Evidently, *EFNB1* or *EPHB4* knockdown significantly decreased but *EFNB1* or *EPHB4* overexpression significantly increased the phosphorylation levels of SRC, ERK and AKT (Fig. 5c). More importantly, we found that the levels of cell cycle proteins, Cyclin D1, Cyclin D3 and CDK2, and EMT proteins, Claudin-1, Slug and β-Catenin, were consistently altered as SRC/ERK/AKT were phosphorylated (Fig. 5d). The protein products of genes in cell cycle and EMT pathways were also increased in HET-1A cells upon forced *EFNB1* or *EPHB4* overexpression (Supplementary Fig. 7d). Subsequently, we conducted rescue assays in KYSE30 using recombinant human EFNB1 (rhEFNB1) and EPHB4 receptor inhibitor BHG712 and found that rhEFNB1 significantly promoted cell proliferation while BHG712 significantly repressed cell proliferation (Supplementary Fig. 7e). Furthermore, rhEFNB1 treatment substantially increased the phosphorylation levels of SRC, ERK and AKT while BHG712 treatment substantially decreased these oncoprotein phosphorylation (Fig. 5e), suggesting that EFNB1-EPHB4 interaction triggers EMT and accelerates cell cycle via activation of SRC/ERK/AKT signaling.

### ∆NP63 overexpression during ESCC progression causes aberrant EFNB1-EPHB4 interaction

**Fig. 6:**
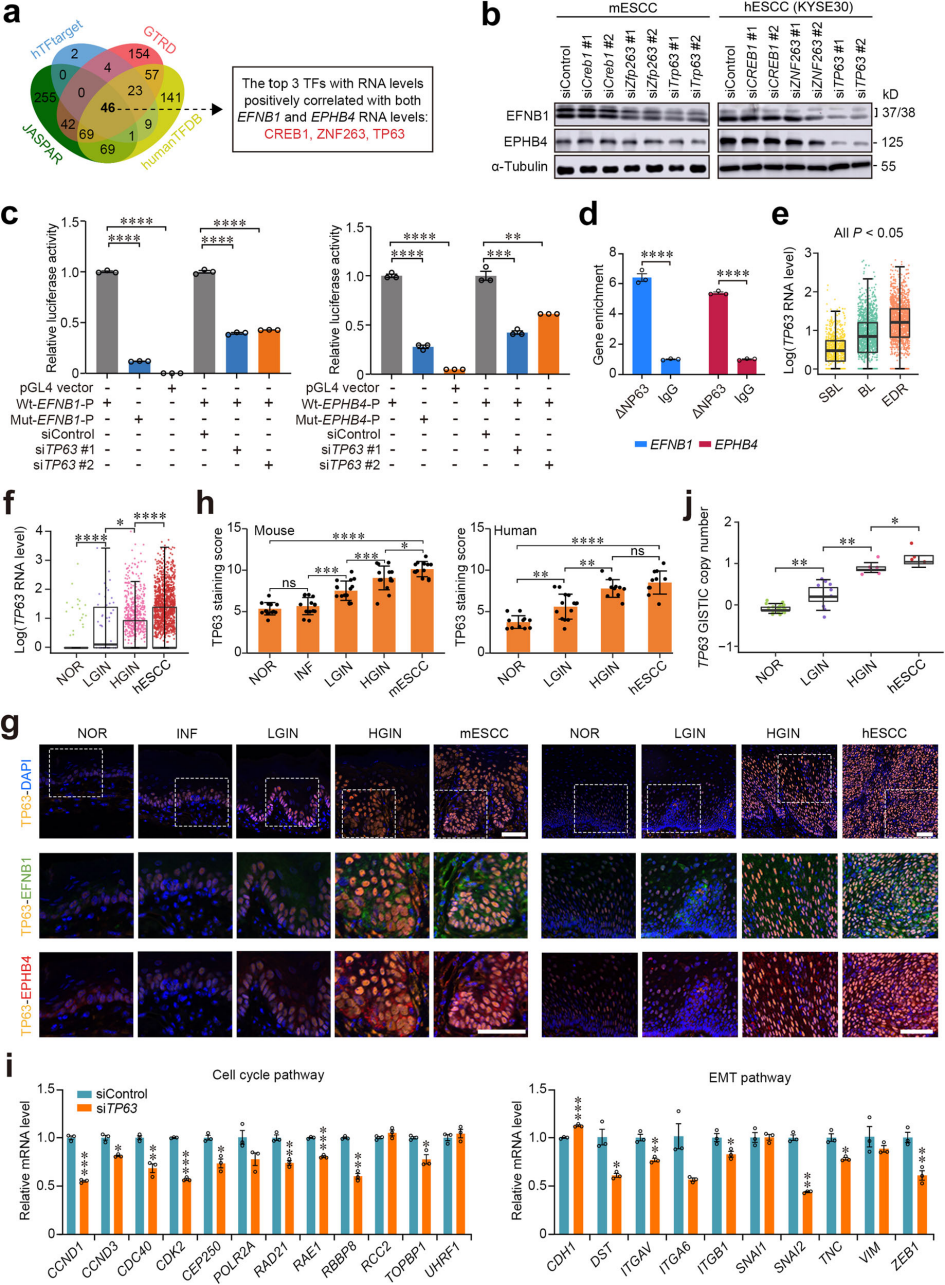
Overexpression of TP63/∆NP63 during ESCC progression causes aberrant EFNB1-EPHB4 interaction. **a** Venn diagram showing potential transcription factors (TFs) that are positively correlated with *EFNB1* and *EPHB4* RNA levels in epithelial cells in scRNA-seq data (GSE160269). **b** The effects of knockdown of three interested TFs on *EFNB1* and *EPHB4* protein levels in mESCC and hESCC cells (KYSE30). Each western blot had three biological replications. **c** Results of reporter gene assays in hESCC cells (KYSE30). Bar plots showing the relative luciferase activity in cells transfected with the indicated reporter plasmids or siRNA. Wt, wild-type *EFNB1* (left panel) or *EPHB4* (right panel) promoter; Mut, mutant *EFNB1* (left panel) or *EPHB4* (right panel) promoter without ΔNP63 binding motif; P, promoter. Data are mean ± SEM from three independent experiments and each had three replications. **P < 0.01; ***P < 0.001; and ****P < 0.0001 of Student’s *t*-test. **d** Chromatin immunoprecipitation and qPCR showing *EFNB1* and *EPHB4* enrichment in the lysate of cells treated with ΔNP63 antibody. Data are mean ± SEM of three biological replications. ****P < 0.0001 of Wilcoxon rank-sum test. **e** The difference of *TP63* RNA level in epithelial cells of different tissue regions of hESCC samples analyzed by spatial transcriptomic sequencing. **f** The difference of *TP63* RNA level in epithelial cells of different disease stages analyzed by scRNA-seq (HRA000776 data). Data in (**e**) and (**f**) are median and 25th to 75th percentile distribution with 1.5× quantile range represented by whiskers. The differences were examined by Wilcoxon rank-sum test. *P < 0.05 and ****P < 0.0001 of Wilcoxon rank-sum test. **g** Representative immunofluorescence images of TP63, EFNB1 and EPHB4 in multistage tissues from mouse (left panel) and human (right panel) esophageal tissues. The colocalization signals are high in the basal layer of NOR (also INF in mice) tissues but expand to the whole layer along the disease progression. Scale bar on upper panel, 50 μm and on lower panel, 25 μm. **h** Quantitative statistics of TP63 staining score in NOR (*n* = 13), INF (*n* = 14), LGIN (*n* = 16), HGIN (*n* = 14), and mESCC (*n* = 12) of mouse tissues and in NOR (*n* = 11), LGIN (*n* = 14), HGIN (*n* = 10), and hESCC (*n* = 11) of human tissues. Data are mean ± SEM and the differences were examined by Wilcoxon rank-sum test. *P < 0.05; ***P < 0.001; ****P < 0.0001; and ns not significant. **i** Verification of gene expression in cell cycle pathway (left panel) and epithelial-to-mesenchymal transition (EMT) pathway (right panel) in *TP63* knockdown hESCC cells (KYSE30). The mRNA levels were determined by qPCR. Data are mean ± SEM from three independent experiments. *P < 0.05; **P < 0.01; ***P < 0.001; ****P < 0.0001; and ns not significant of Student’s *t*-test. **j** GISTIC copy number of *TP63* in NOR (*n* = 22), LGIN (*n* = 11), HGIN (*n* = 5) and hESCC (*n* = 4) tissue samples. Data are median and 25th to 75th percentile. *P < 0.05 and **P < 0.01 of Wilcoxon rank-sum test. SBL suprabasal layer, BL basal layer, EDR ESCC development region, NOR normal tissue, INF inflammation, LGIN low-grade intraepithelial neoplasia, HGIN high-grade intraepithelial neoplasia, hESCC human esophageal squamous-cell carcinoma. Also see Supplementary Fig. S8

We next explored why epithelial cell EFNB1-EPHB4 interaction during ESCC progression is strongly enhanced. Since whole-genome bisulfite sequencing and whole-genome sequencing of multistage diseased esophageal epithelial tissues did not show significant differences in the promoter methylation modification and DNA copy number variation of *EFNB1* and *EPHB4*, we thus looked at the transcription factors that may regulate *EFNB1* and *EPHB4* expression. In silico analysis using four databases, we identified 46 transcription factors that may potentially regulate *EFNB1* and *EPHB4* expression (Fig. 6a and Supplementary Table S7). However, the correlation analysis showed that among them, only *TP63*, *ZNF263* and *CREB1* were significantly correlated with *EFNB1* and *EPHB4* in terms of expression levels (Supplementary Fig. 8a). In vitro gene operation assays showed that in mESCC and hESCC cells (KYSE30), only *TP63* but not *ZNF263* and *CREB1* knockdown significantly suppressed *EFNB1* and *EPHB4* mRNA and protein expression (Fig. 6b and Supplementary Fig. 8b, c). We then looked at the *EFNB1* and *EPHB4* promoter sequences and found that both contain a TP63 binding motif (Supplementary Fig. 8d). It has been reported that the primary TP63 isoform in human esophagus is ∆NP63.^21,22^ We thus conducted luciferase reporter gene assays and the results showed that the activity of reporter gene having wild-type *EFNB1* or *EPHB4* promoter was significantly higher than that having mutant *EFNB1* or *EPHB4* promoter, which lacks the ∆NP63 binding motif (Fig. 6c and Supplementary Fig. 8e). Moreover, knockdown of *TP63* in cells remarkedly suppressed the reporter gene activity (Fig. 6c). We also used anti-∆NP63 antibody to perform chromatin immunoprecipitation-coupled qPCR assays in KYSE30 cells and the results showed substantial enrichment of *EFNB1* and *EPHB4* (Fig. 6d), reflecting that ∆NP63 binds both *EFNB1* and *EPHB4* promoters in these cells.

We further investigated *TP63* spatiotemporal alteration and its effect on esophageal development. Spatiotemporal analysis showed that in human and mouse esophageal tissue samples, BL and EDR had significantly higher *TP63* expression levels than other tissue regions (Fig. 6e and Supplementary Fig. 8f). Single-cell transcriptomic analysis also showed that *TP63* expression levels in human precancerous and cancerous esophageal epithelial cells were significantly higher than that in normal epithelial cells (Fig. 6f). We reanalyzed one bulk RNA-seq and one scRNA-seq datasets of hESCC samples that we reported previously^7,16^ and found that *TP63* RNA levels in tumor samples were significantly higher than that in normal tissue samples (Supplementary Fig. 8h, i). We also performed immunofluorescence assays in both mouse and human tissue samples and the results showed that the staining scores of TP63 protein along with EFNB1 and EPHB4 proteins were significantly higher in precancerous and cancerous epithelia compared with normal epithelium and cells with high staining was mainly located in the basal layer but expanded to whole epithelial layer along the disease progression (Fig. 6g, h). However, we noted that in multistage mouse samples, the TP63 protein levels were not consistent with the RNA levels determined by scRNA-seq (Supplementary Fig. 8g). We further knocked down *TP63* in KYSE30 cells and found that the mRNA levels of genes in the cell cycle and EMT pathways were significantly reduced (Fig. 6i), which were like the effects of *EFNB1* or *EPHB4* knockdown, suggesting that *TP63, EFNB1* and *EPHB4* are in the same functional axis and *TP63* is in upstream.

We then explored why *TP63* expression was elevated in epithelial cells during hESCC progression. We analyzed *TP63* promoter methylation and copy number variation states in our previously reported multiple disease stage tissue samples from ESCC patients^8^ and found that although the *TP63* promoter methylation levels did not change significantly (Supplementary Fig. 8j), the copy numbers of *TP63* were gradually and significantly elevated along with disease progression (Fig. 6j). Since *TP63* expression is regulated by TP53, we thus analyzed the effect of *TP53* mutation state on the copy number of *TP63* using our whole-exon sequencing data of multiple disease state tissue samples.^23^ The results showed that compared with clones without *TP53* mutation, clones having *TP53* mutations had significantly increased *TP63* GISTIC copy numbers, and the amplification was significantly more in clones with multiple *TP53* mutations or loss of heterozygosity (Supplementary Fig. 8k). Together, these results suggest that the amplification and overexpression of *TP63* due to *TP53* loss of function causes elevated EFNB1-EPHB4 interaction among epithelial cells, which promotes ESCC progression via accelerated cell cycle and EMT.

## Discussion

**Fig. 7:**
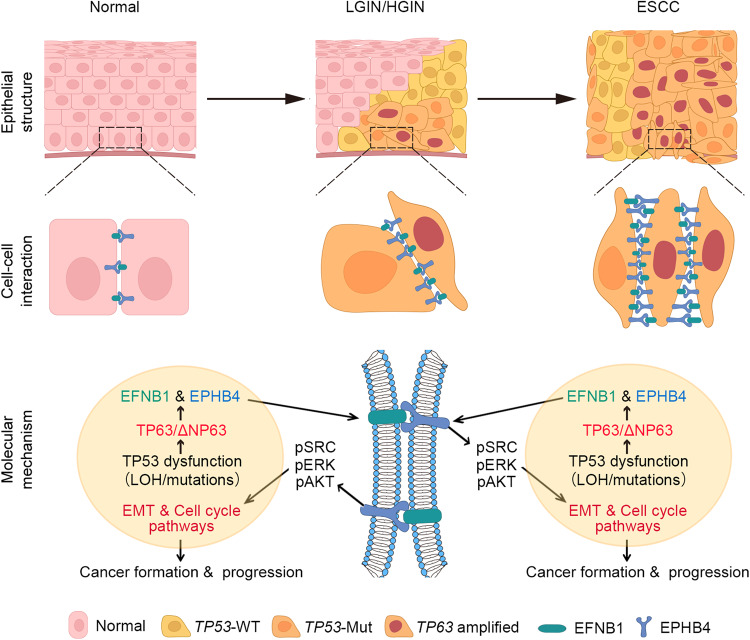
The schematic illustration for the possible mechanism that the aberrant TP53-TP63/ΔNP63-EFNB1-EPHB4 signaling triggers EMT and accelerates cell cycle during ESCC formation and development. In epithelial cells, TP53 dysfunction due to loss of heterozygosity (LOH) and mutations causes TP63/ΔNP63 amplification and overexpression, which upregulates *EFNB1* and *EPHB4* expression. The overexpressed EFNB1 and EPHB4 enhance their mediated aberrant cell–cell interaction among epithelial cells, resulting in the downstream SRC/ERK/AKT signaling activation, which triggers epithelial-mesenchymal transition and accelerates cell cycle to promote ESCC formation and progression

The multistage ESCC development involves dynamic intercellular signal transductions in the tissue microenvironment; however, the spatial distribution of the precancerous and cancerous cells and the key molecules in situ remain unclear. In the present study, we have performed spatial transcriptomic analysis of five different diseased esophageal tissues from mice induced by carcinogen 4-NQO^6^ to investigate how epithelial cells interact and what molecules mediate these interactions during ESCC progression, and the results are compared in multistage human tissue samples. We have obtained several novel findings. First, we have identified an ESCC development region (EDR) with conspicuous tumor hallmarks that emerged from INF and enlarged along disease progression, which has been validated in multistage human esophageal samples by spatial and single-cell transcriptomic analysis. Second, in the EDR, epithelial cells show aberrantly increased interaction via EFNB1-EPHB4 crosstalk, resulting in accelerated cell proliferation and EMT. Third, functional analysis demonstrates that the EFNB1-EPHB4 interaction activates SRC/ERK/AKT signaling, which may be the mechanism underlying increased proliferation and EMT of the diseased esophageal epithelial cells. Finally, we have found that aberrant EFNB1-EPHB4 expression and interaction in epithelial cells is caused by TP53 dysfunction-induced *TP63* amplification. Together, these results uncover for the first time the important role of TP53-TP63/∆NP63-EFNB1-EPHB4 signaling in ESCC development and progression (Fig. 7).

Ephrin and Eph proteins are located on cell membrane and their interaction can trigger short-range cell–cell communication between adjacent cells, resulting in a variety of biological processes.^24^ As members of Ephrin/Eph family, EFNB1 and EPHB4 share similar effects in cancer. It has been reported that overexpressed EFNB1 can interact with its receptor EPHB2 to stimulate tumor growth in gastric adenocarcinoma^25^ EPHB4 has been found to be upregulated in a wide range of cancer types, including head and neck squamous-cell carcinoma and esophageal squamous-cell carcinoma.^26–28^ A recent study has also shown that EPHB2 is the regulator of proto-oncogene *MYC* in Barrett Neoplasia.^29^ In the present study, we have demonstrated that *EFNB1* and *EPHB4* expression levels have gradually and significantly elevated in both mouse and human esophageal epithelial cells along with ESCC progression. More importantly, we have found that epithelial cells with aberrantly elevated EFNB1-EPHB4 interaction mainly originated from the basal layer of the epithelium and then expand to the up-layer when the disease progresses, which accelerates proliferation of diseased cells and triggers EMT, facilitating ESCC development and progression. These novel findings provide another important evidence to support a broad range of pharmacological designs targeting EPHB4 or the Eph/Ephrin interaction to prevent or cure some types of cancer.^30–32^ These findings may also be significant in ESCC early detection by using the EPHB4-specific examination established in the future.

EMT is a highly complex and coordinated process in which epithelial cells lose multiple biological characteristics associated with epithelial differentiation while acquiring behaviors resembling mesenchymal cells concurrently.^33^ It has been documented that EMT plays essential roles in tumor progression and metastasis;^34–36^ however, several recent studies have also highlighted a key role of EMT in cancer initiation and development. For instance, inflammation-induced EMT states in precancerous lesions of pancreatic ductal adenocarcinoma mouse models can facilitate tumor formation and early dissemination.^37^ In mouse models and human samples, YAP1 and SOX2 activation induced hybrid EMT states causes squamous cells loss of cell polarity and adhesion, which promotes tumor initiation and metastasis.^38^ In the present study, we have discovered for the first time that the aberrant EFNB1-EPHB4 interaction, occurring at INF/LGIN stage and increasing at HGIN and ESCC stages, can trigger esophageal epithelial cell EMT and proliferation. More importantly, we have demonstrated that the EFNB1-EPHB4 interaction enhances the transformation of normal epithelial cells into malignant phenotypes, implying that this aberrant interaction may be an early event in ESCC formation.

We have investigated why *EFNB1* and *EPHB4* in epithelial cells are aberrantly overexpressed during ESCC progression and discovered that transcription factor ΔNP63 is the prime culprit. It is well known that TP63 has two isoforms, TAP63 and ΔNP63, their expression is regulated by distinct promoters.^39^ The expression patterns of TAP63 and ΔNP63 differ in different tissue types and ΔNP63 is the primary isoform in the esophagus.^21,22^ Prevailing genomic analysis has shown that *TP63* is a key oncogene with frequent amplification and overexpression in hESCC.^40,41^ In addition to inducing certain oncogene expression,^42–44^ ΔNP63 has also been reported to promote the expression of EMT markers in human cancers including ESCC.^41,45^ In the present study, we have found that *TP63* amplification and overexpression occurs early in the precancerous lesions and increases gradually along ESCC progression, and demonstrated for the first time that ΔNP63 is the common transcription factor of both *EFNB1* and *EPHB4*. Interestingly, we have found that ΔNP63 overexpression in precancerous and cancerous epithelial cells may have resulted from copy number alteration induced by TP53 dysfunction. TP53 dysfunction can cause widespread genomic disruption such as genomic rearrangements.^46,47^ Recently, we have analyzed 1,275 micro-biopsies from normal, precancerous and cancerous esophageal samples to decipher driver mutations that promote ESCC development and progression and found that *TP53*-biallelic loss or multiple mutation is the major driver, probably by causing copy number alterations of certain chromosomal regions.^23^ By analyzing the genomic data, we have found that *TP63*, located on chromosome 3, is within TP53 dysfunction-induced amplified region, suggesting that *TP63* aberrance may be caused by *TP53* mutations in epithelial cells.

In conclusion, the present study clarifies the spatiotemporal alterations of intercellular signal transductions in tissue microenvironment during mouse and human ESCC development. Specifically, we have uncovered the aberrant EFNB1-EPHB4 interaction among epithelial cells coming from the basal layer that triggers EMT and cell proliferation in ESCC development and progression. We have also identified that *TP53* mutation-induced *TP63* overexpression is the cause of aberrant EFNB1-EPHB4 interaction. These results suggest an important effect of aberrant TP53-TP63/ΔNP63-EFNB1-EPHB4 signaling axis on oncogenic disorganization of esophageal tissues. Discovering these spatiotemporal gene regulatory networks within tissue microenvironment might have clinical implication in establishing early diagnosis and therapy based on these molecules for ESCC and perhaps other epithelial cancers.

## Materials and methods

### Mouse ESCC induction and sample collection

Animal experiments in this study were carried out in conformity with approved ethical protocols and guidelines from the Institutional Animal Care and Use Committee of the Chinese Academy of Medical Sciences. We established 4-NQO-induced mouse ESCC models as described previously.^6^ Briefly, 180 seven-week-old female C57BL/6 mice were administered with 4-NQO (Sigma-Aldrich) in drinking water (100 μg/ml). The esophagus was removed from three mice every 3 days from the beginning of 4-NQO treatment and end in 26 weeks. The esophageal samples were frozen at −80 °C for histopathological examination and spatial transcriptomic analysis.

### Spatial transcriptome sequencing and data processing

We performed Visium spatial transcriptome sequencing (10× Genomics) on five mouse esophageal tissues, i.e., NOR (taken at week 0), INF (taken at week 14), LGIN (taken at week 18), HGIN (taken at week 24), and mESCC (taken at week 26), with high RNA quality. The frozen samples were embedded in Optimal Cutting Temperature compound (OCT, Sakura Tissue-TEK) on dry ice, with NOR/INF in one cryomold and LGIN/HGIN/mESCC in the other. The OCT blocks were cut at 10 μm thickness with a pre-cooled cryostat and transferred onto the 6.5 mm × 6.5 mm oligo-barcoded capture areas of the Visium Spatial Gene Expression slides. We then acquired tissue H&E staining images and prepared sequence libraries and sequenced at the recommended depth using Illumina NovaSeq 6000 platform. Raw sequencing data were mapped to the pre-built mm10 reference genome using the Spaceranger workflow (version 1.0.0), generating a dataset of 2022 spots (i.e., 156 NOR, 503 INF, 342 LGIN, 303 HGIN, and 718 mESCC). We manually separated mucosa-submucosa and muscularis using Loupe Browser software (version 4.1.0) based on the tissue histology (Supplementary Fig. 1b). After quality control, mucosa-submucosa containing 977 spots (87 NOR, 225 INF, 185 LGIN, 94 HGIN, and 386 mESCC) was processed for further analysis. The median number of genes in each spot was 3849 and the median number of reads in each spot was 10,037. We applied the same approach to manually isolate the mucosa-submucosa of 3752 spots (994 NOR, 441 LGIN, 888 HGIN, and 1429 hESCC) in the human spatial transcriptome dataset^8,48^ for in-depth analysis. The median number of genes in each spot was 4485 and the median number of reads in each spot was 13,119.

### Dimensionality reduction and graph-based clustering

We conducted downstream analysis on mouse and human spatial transcriptome data using Seurat package (version 4.1.0) in R (version 4.1.3).^49^ Principal component analysis and UMAP dimensionality reduction were performed after *SCTransform* normalization.^50^ Batch effects across different pathological stages were eliminated by Harmony package (version 0.1.0).^51^ Unsupervised graph-based clustering was implemented using the shared nearest neighbor method *FindNeighbors* and *FindClusters* with resolution of 0.4. Each cluster was further designated by the histology distribution and the marker genes identified using *FindAllMarkers* function.

### Gene set variation analysis

We used GSVA package (version 1.42.0)^52^ with the default parameters to estimate the activities of Gene Ontology (GO) pathways exported from MSigDB package (version 7.5.1).^53^ Differential pathway activity of each cluster was calculated using Limma package (version 3.50.3).^54^ The significant differential pathways (*P* < 0.05) with high pathway activity scores were visualized using heatmap for each tissue region.

### Cell–cell interaction analysis of spatial transcriptome data

We carried out cell–cell interaction analysis in mouse and human spatial transcriptome data using stLearn software (version 0.4.8) in Python (version 3.10.5).^55^ The known ligand-receptor (LR) gene pairs of mouse and human were loaded from the connectomeDB (2020) database. We implemented *st.tl.cci.run* function to find spots with significant LR gene pairs (*P* < 0.05) using permutation test with standard settings. A union of 1,100 significant LR gene pairs were identified in the mouse spatial transcriptome data. To infer cluster-specific cell–cell interactions, we computed the ratio of each LR gene pairs in 5 tissue regions (spot counts with significant LR gene pairs in each tissue region/total spot counts of each tissue region)^56^ and presented the ratio of cluster-specific LR gene pairs by heatmap. The cell–cell interaction score of each LR gene pair was determined by *lr_scores* stored in *adata.obsm*.

### Cell–cell interaction analysis of single-cell transcriptome data

We performed cell–cell interaction analysis on mouse and human single-cell transcriptome data using CellChat package (version 1.6.1) in R (version 4.1.3).^57^ Briefly, the CellChatDB database was loaded with the known ligand-receptor gene pairs in mouse and human. Then we input our single-cell transcriptome data with cell type annotations to identify overexpressed ligand or receptor genes in each cell–cell group using the functions *identifyOverExpressedGenes* and *identifyOverExpressedInteractions*. Subsequently, the function *computeCommunProb* was used to infer the biologically significant cell–cell communication by assigning each interaction with a probability value and performing a permutation test. Finally, we integrated multistage datasets and used the function *netVisual_bubble* to visualize cell–cell communication probability of multistage samples.

### Gene correlation analysis and enrichment analysis

We extracted all detected genes in epithelial cells from single-cell transcriptome data by Zhang et al.^7^ and used R package psych (version 2.2.3) to calculate genes with RNA levels significantly correlated with *EFNB1* or *EPHB4* in each sample (*P* < 0.05 and |*r*| > 0.3). We also identified transcription factors with RNA levels that were highly correlated with *EFNB1* or *EPHB4* according to these results. After that, R package clusterProfiler (version 4.2.2) and gene sets from Msigdb database were used to perform further enrichment analysis.

### Survival analysis among patients with ESCC

We estimated the overall survival time of 233 patients with ESCC using the log_2_ transformed TPM data of bulk RNA-seq^7^ by the Kaplan–Meier method and the log-rank test. In brief, we estimated tumor purity of each sample using R package estimate (version 1.0.13) to normalize the RNA level of each gene. The best cutoffs were determined by the *surv_cutpoint* function in R package survminer (version 0.4.9) to obtain the most significant relationship with the survival probability using the maximally selected rank statistics. Hazard ratios (HRs) and their 95% confidence intervals (CIs) were calculated by Cox proportional hazards models using age, sex, and tumor stage as covariates.

### Spatiotemporal analysis of *TP63* expression

We conducted spatiotemporal analysis of *TP63* expression in spatial and single-cell transcriptome datasets of mouse and human multistage samples. The RNA levels of *TP63* in different tissue regions were analyzed in spatial transcriptome datasets, and the RNA levels of *TP63* in epithelial cells from multistage samples were determined in single-cell transcriptome datasets.

### Whole-genome bisulfite sequencing analysis

We performed whole-genome bisulfite sequencing (WGBS) in 40 multistage samples from 14 ESCC patients as reported previously.^8^ In brief, sequencing reads were mapped to the hg38 genome, and methylation levels were called using Bismark software (version 0.19.0). Duplications were removed by Picard tools (version 2.4.1). Subsequent methylation loci were analyzed by DSS package (version 2.38.0). We calculated the average methylation levels of all CpG sites 500 bp upstream of TSS in each sample for further analysis.

### Whole-genome sequencing analysis

We performed low-depth whole-genome sequencing (WGS) in 42 multistage samples from 28 ESCC patients in accordance with our previous paper.^8^ We used GISTIC2 software (version 2.0.23) to identify significantly altered gene regions and computed the GISTIC copy number of *TP63* in each sample. The cutoffs of gain and loss of gene copy number were set to log_2_(2.5/2) and log_2_(1.5/2), respectively.

### Multiplex immunofluorescence assay

We performed multiplex immunofluorescence staining on different FFPE sections of multistage mouse and human esophageal tissues by the seven-color multi-labeling kit (PANOVUE). Different fluorescence signals from Opal 520, Opal 570, and Opal 650 were generated corresponding to TP63 (ab124762, Abcam), EPHB4 (20883-1-AP, Proteintech), and EFNB1 (12999-1-AP, Proteintech), respectively. The sections were counterstained with DAPI for nuclear visualization and coverslipped with anti-fade mounting medium. Multispectral images were scanned using Vectra Polaris Automated Quantitative Pathology Imaging System (AKOYA) and then unmixed to remove autofluorescence by inForm software (AKOYA, version 2.6) as described.^58^ The protein expression levels were scored as 1 (weak), 2 (moderate), and 3 (strong), the positive cell percentages were scored as 1 (<10%), 2 (10–50%), 3 (50–80%), and 4 (>80%), and the product of these two results represented staining score ranked from 1 to 12 in each section.

### Immunohistochemical staining and analysis

We conducted immunohistochemical (IHC) staining assays in tissue sections from mouse subcutaneous xenografts using antibody against EFNB1 (12999-1-AP, Proteintech) or EPHB4 (20883-1-AP, Proteintech) and the SABC Kit (SA0041, Solarbio). Protein staining intensity and percentage of positive cells were quantified in three non-overlapping fields and the protein levels were evaluated as 1 (weak), 2 (moderate) and 3 (strong). The scores for the positive cell percentage of were determined as 1 (<10%), 2 (10–50%), 3 (51–80%) and 4 (>80%). The IHC scores were obtained by multiplying protein levels and corresponding cell percentages, ranking from 1 to 12.

### Isolation and culture of primary mouse ESCC cells

We isolated primary mESCC cells from mouse ESCC tumor tissues using tissue block method. The mESCC sample was cut into 1 mm^3^ tissue blocks and cultured in advanced DMEM/F12 (Gibco) supplemented with B-27 (17504-044, Invitrogen), N-2 (17502-048, Invitrogen), HEPES (Gibco), GlutaMAX^TM^ (Gibco), penicillin (100 U/ml), and streptomycin (100 mg/ml) in a humidified 5% CO_2_ incubator at 37 °C. When the adherent epithelial cells reached their proper density, they were digested and passaged with primary culture medium. Homogenous primary mESCC cells were acquired after three passages and cultured in 10% FBS DMEM for further analysis.

### Human esophageal cell lines and cell culture

hESCC cell line KYSE30 and KYSE150 was a generous gift from Dr Y. Shimada of Hyogo College of Medicine, Japan. Immortalized normal esophagus epithelial cell line HET-1A was purchased from American Type Culture Collection. All cell lines were validated by DNA fingerprinting analysis and tested free of mycoplasma infection. KYSE30 and KYSE150 cells were cultured in RPMI 1640 medium with 10% FBS under 5% CO_2_ conditions. For rhEFNB1 (10894-H08H, Sinobiological)^59,60^ or EPHB4 inhibitor BHG712 (MCE)^20,61^ treatment, the reagents were dissolved in water or DMSO, respectively, and added to cell culture medium containing 2% FBS at a final concentration of 3 μg/ml (rhEFNB1) or 1.0 μM (BHG712).

### Plasmids and small interfering RNA transfection

Mouse *Efnb1* and *Ephb4* and human *EFNB1* and *EPHB4* expression plasmids were purchased from GeneChem. Small interfering RNAs targeting mouse *Efnb1*/*Ephb4*/*Creb1*/*Zfp263*/*Trp63* and human *EFNB1*/*EPHB4*/*CREB1*/*ZNF263*/*TP63* were purchased from GenePharma (Supplementary Table S8). The transfection of plasmids and small interfering RNAs was performed using jetPRIME (Polyplus).

### Establishment of cell lines with altered *EFNB1* or *EPHB4* expression

Lentiviruses for stable *EFNB1* or *EPHB4* overexpression (Ubi-MCS-EGFP-IRES-Puromycin) and knockdown (hU6-MCS-CBh-gcGFP-IRES-puromycin) were from GeneChem as viral particles. The negative control sequence was TTCTCCGAACGTGTCACGT. The stable knockdown target sequences for *EFNB1* were CTTACGGACTACAGAGAACAA and CCAGAGCAGGAAATACGCTTT. The stable knockdown target sequences for *EPHB4* were GCGCACCTACGAAGTGTGTGA and GCACATGAAGTCCCAGGCCAA. hESCC cells (KYSE30) were infected with the viruses and cultured in a complete medium for 24 h followed by selection with puromycin (S7417, Selleck). The efficiency of overexpression or knockdown was examined by western blotting.

### Cell viability assays and analysis of invasion and migration

We seeded mouse and human ESCC cells in 96-well plates and assessed cell viability at 5 time points using the Cell Counting Kit-8 (DOJINDO). Each experiment was performed 3 times and each had 6 replicates. Invasion assays were conducted using Millicell chambers coated with 80μL of Matrigel (500 ng/μL). Cells were seeded onto the coated filters in serum-free medium and incubated for 18–22 hours. The migrated cells were subsequently fixed with methanol and stained. The migration assay was performed in a similar manner, but without the Matrigel coating on the filters.

### Real-time quantitative PCR

We extracted total RNA from mouse and human cells using the RNA-Quick Purification Kit (ES Science) and conducted reverse transcription using PrimeScript^TM^ RT Master Mix (Takara). The mRNA levels were detected in QuantStudio 5 Real-Time PCR system and Quantagene q900 qPCR system (Kubo Technology, Beijing) with TB Green® Premix Ex Taq^TM^ II (Takara) and the corresponding primers (Supplementary Table S8). The individual mRNA level was calculated relative to *GAPDH* mRNA level.

### Western blot assay

We extracted total protein from tissues or cells using RIPA lysis buffer (Solarbio) containing Protease and Phosphatase Inhibitor Cocktail (NCM Biotech) and the protein concentration was determined using BCA kit (Thermo Fisher Scientific). Total protein separated on SDS-PAGE (E303-01, Vazyme) was transferred to PVDF membrane (Millipore) and then incubated with primary antibody overnight at 4 °C followed by incubation with secondary antibody (Cell Signaling Technology) for 2 h at room temperature. Antibody against AKT (4691), Phospho-AKT (Ser473; 4060), ERK1/2 (4695), Phospho-ERK1/2 (T202/204; 4370), SRC (2109), Phospho-SRC (T416; 6943), CDK2 (2546), Cyclin D3 (2936), Claudin-1 (13255), Slug (9585), or β-Catenin (8480) was from Cell Signaling Technology. Antibody against α-Tubulin (11224-1-AP), EFNB1 (12999-1-AP), EPHB4 (20883-1-AP), or Cyclin D1 (60186-1-Ig) was from Proteintech. Antibody against Histone H3 (ab1791) was from Abcam. The protein signals were detected in Amersham Imager 600 using a high sensitivity ECL chemiluminescence detection kit (E412-01, Vazyme).

### Immunoprecipitation assays

Total proteins were extracted from mouse and human cells using weak RIPA lysis buffer (Beyotime) with Protease and Phosphatase Inhibitor Cocktail (NCM Biotech). We conducted immunoprecipitation with EFNB1 or EPHB4 using Pierce™ Classic Magnetic IP/Co-IP Kit (Invitrogen) and uploaded the eluted products to western blot. Secondary antibody against IgG light chain (A25012 and A25022) was from Abbkine.

### Immunofluorescence assays

mESCC and hESCC (KYSE30) were grown to 50–70% confluency on the coverslips (Solarbio). After PBS washes, cells on coverslips were fixed with 4% paraformaldehyde and permeabilized with 0.2% Triton X-100. The coverslips were blocked with 3% bovine serum albumin and then incubated with antibody against EFNB1, EPHB4 (sc-130081, Santa Cruz Biology) and VIM (ab8978, Abcam). Goat anti-mouse secondary antibody with Alexa Fluor 594 (A-11005) and goat anti-rabbit secondary antibody with Alexa Fluor 488 (A-11008) from Thermo Fisher were used as the secondary antibody. Confocal microscopy (AKOYA) was used to capture the images after coverslips were sealed with mounting medium contained DAPI (ZSGB-BIO).

### Chromatin immunoprecipitation-coupled quantitative PCR analysis

We used SimpleChIP® Plus Sonication Chromatin IP Kit (56383, Cell Signaling Technology) to carry out chromatin immunoprecipitation assays. KYSE30 cells were fixed and cross-linked with formaldehyde before immunoprecipitation with ΔNP63 antibody (67825, Cell Signaling Technology) or rabbit IgG antibody (3900, Cell Signaling Technology). DNA fragments were quantified using qPCR with the primers shown in Supplementary Table S8.

### Mouse xenograft experiment

KYSE30 cells (2.5 × 10^6^) with or without *EFNB1* or *EPHB4* stable overexpression or knockdown were suspended in 200 μl diluted Growth Factor Reduced Matrigel^®^ Matrix (Corning) and injected into the hind legs of 6-week-old NSG mice. We palpated tumor development and monitored growth rate by measuring tumor volumes with caliper every 3 days, which were defined as length × width^2^ × 0.52. Tumors were obtained 28 days after transplantation for further analysis.

### Cell cycle analysis

KYSE30 cells were collected and fixed in cold 70% ethanol overnight at 4 °C. Single-cell suspensions were labeled with propidium iodide/RNase working solution (DOJINDO) and detected by flow cytometry (BD). The data were analyzed by FLOW JO (version 10.8).

### Luciferase reporter gene assays

We cloned wild-type or mutant-type human *EFNB1* and *EPHB4* promoters (from −2000 bp to the transcription start site) into pGL4.10 vector and then conducted luciferase reporter assays according to the manufacturer’s instructions (E1960, Promega). The mutant-type *EFNB1* and *EPHB4* promoters lacked ∆NP63 binding motif.

### Statistical analysis

We performed statistical analysis using R 4.1.3 and GraphPad Prism 9. The statistical methods and details were described in the figure legends, text, or other part of Methods, respectively. The two-sided and unpaired Wilcoxon rank-sum test and Student’s *t*-test were used to compute *P*-values. In the experiments, error bars represented the standard error of the mean (SEM) for at least three independent assays.

### Supplementary information

Supplementary Materials

## Data Availability

The raw spatial transcriptome data of multistage mouse ESCC samples have been deposited in Genome Sequence Archive in BIG Data Center, Beijing Institute of Genomics, Chinese Academy of Sciences, https://bigd.big.ac.cn/gsa under the accession number CRA012359. The four published datasets are achievable in the GSA database (CRA002118), GEO Database (GSE160269) and GSA-HUMAN database (HRA000003 and HRA000776). All other data and information supporting the findings are available in the article and Supplementary files. The original code is accessible from the corresponding author upon reasonable request.

## References

[1] Abnet CC, Arnold M, Wei WQ (2018). Epidemiology of esophageal squamous cell carcinoma. Gastroenterology.

[2] Nagtegaal ID (2020). The 2019 WHO classification of tumours of the digestive system. Histopathology.

[3] Wang GQ (2005). Histological precursors of oesophageal squamous cell carcinoma: results from a 13 year prospective follow up study in a high risk population. Gut.

[4] Wei WQ (2015). Long-term follow-up of a community assignment, one-time endoscopic screening study of esophageal cancer in China. J. Clin. Oncol..

[5] Wei WQ (2020). Esophageal histological precursor lesions and subsequent 8.5-year cancer risk in a population-based prospective study in China. Am. J. Gastroenterol..

[6] Yao J (2020). Single-cell transcriptomic analysis in a mouse model deciphers cell transition states in the multistep development of esophageal cancer. Nat. Commun..

[7] Zhang X (2021). Dissecting esophageal squamous-cell carcinoma ecosystem by single-cell transcriptomic analysis. Nat. Commun..

[8] Chen Y (2023). Epithelial cells activate fibroblasts to promote esophageal cancer development. Cancer Cell.

[9] Shimizu M, Nagata K, Yamaguchi H, Kita H (2009). Squamous intraepithelial neoplasia of the esophagus: past, present, and future. J. Gastroenterol..

[10] Ohashi S (2015). Recent advances from basic and clinical studies of esophageal squamous cell carcinoma. Gastroenterology.

[11] Shah MA (2023). Improving outcomes in patients with oesophageal cancer. Nat. Rev. Clin. Oncol..

[12] Ståhl PL (2016). Visualization and analysis of gene expression in tissue sections by spatial transcriptomics. Science.

[13] Ji AL (2020). Multimodal analysis of composition and spatial architecture in human squamous cell carcinoma. Cell.

[14] Qi J (2022). Single-cell and spatial analysis reveal interaction of FAP(+) fibroblasts and SPP1(+) macrophages in colorectal cancer. Nat. Commun..

[15] Kanojia D, Vaidya MM (2006). 4-Nitroquinoline-1-oxide induced experimental oral carcinogenesis. Oral. Oncol..

[16] Chang J (2017). Genomic analysis of oesophageal squamous-cell carcinoma identifies alcohol drinking-related mutation signature and genomic alterations. Nat. Commun..

[17] Chang F (2003). Involvement of PI3K/Akt pathway in cell cycle progression, apoptosis, and neoplastic transformation: a target for cancer chemotherapy. Leukemia.

[18] Ortiz MA (2021). Src family kinases, adaptor proteins and the actin cytoskeleton in epithelial-to-mesenchymal transition. Cell Commun. Signal.

[19] Pradeep S (2015). Erythropoietin stimulates tumor growth via EphB4. Cancer Cell.

[20] Zhu M (2020). Cantharidin treatment inhibits hepatocellular carcinoma development by regulating the JAK2/STAT3 and PI3K/Akt pathways in an EphB4-dependent manner. Pharm. Res..

[21] Hu H (2002). Elevated expression of p63 protein in human esophageal squamous cell carcinomas. Int. J. Cancer.

[22] Pokorná Z, Vysloužil J, Hrabal V, Vojtěšek B, Coates PJ (2021). The foggy world(s) of p63 isoform regulation in normal cells and cancer. J. Pathol..

[23] Chang, J. et al. Genomic alterations driving precancerous to cancerous lesions in esophageal cancer development. *Cancer Cell*10.1016/j.ccell.2023.11.003 (2023). 10.1016/j.ccell.2023.11.003 38039962

[24] Kania A, Klein R (2016). Mechanisms of ephrin-Eph signalling in development, physiology and disease. Nat. Rev. Mol. Cell Biol..

[25] Kataoka H (2002). Expression profile of EFNB1, EFNB2, two ligands of EPHB2 in human gastric cancer. J. Cancer Res. Clin. Oncol..

[26] Kumar SR (2009). Preferential induction of EphB4 over EphB2 and its implication in colorectal cancer progression. Cancer Res..

[27] Bhatia S (2018). Inhibition of EphB4-Ephrin-B2 signaling enhances response to cetuximab-radiation therapy in head and neck cancers. Clin. Cancer Res..

[28] Hasina R (2013). Critical role for the receptor tyrosine kinase EPHB4 in esophageal cancers. Cancer Res..

[29] Venkitachalam S (2022). The Ephrin B2 receptor tyrosine kinase is a regulator of proto-oncogene MYC and molecular programs central to Barrett’s neoplasia. Gastroenterology.

[30] Kertesz N (2006). The soluble extracellular domain of EphB4 (sEphB4) antagonizes EphB4-EphrinB2 interaction, modulates angiogenesis, and inhibits tumor growth. Blood.

[31] Krasnoperov V (2010). Novel EphB4 monoclonal antibodies modulate angiogenesis and inhibit tumor growth. Am. J. Pathol..

[32] Pietanza MC (2012). Phase II study of the multitargeted tyrosine kinase inhibitor XL647 in patients with non-small-cell lung cancer. J. Thorac. Oncol..

[33] Huang Z (2022). Epithelial-mesenchymal transition: the history, regulatory mechanism, and cancer therapeutic opportunities. MedComm.

[34] Dongre A, Weinberg RA (2019). New insights into the mechanisms of epithelial-mesenchymal transition and implications for cancer. Nat. Rev. Mol. Cell Biol..

[35] Pastushenko I, Blanpain C (2019). EMT transition states during tumor progression and metastasis. Trends Cell Biol..

[36] Lambert AW, Weinberg RA, Linking EMT (2021). programmes to normal and neoplastic epithelial stem cells. Nat. Rev. Cancer.

[37] Rhim AD (2012). EMT and dissemination precede pancreatic tumor formation. Cell.

[38] Pastushenko I (2021). Fat1 deletion promotes hybrid EMT state, tumour stemness and metastasis. Nature.

[39] Yang A (1998). p63, a p53 homolog at 3q27-29, encodes multiple products with transactivating, death-inducing, and dominant-negative activities. Mol. Cell.

[40] Cancer Genome Atlas Research Network (2017). Integrated genomic characterization of oesophageal carcinoma. Nature.

[41] Fisher ML, Balinth S, Mills AA (2023). ΔNp63α in cancer: importance and therapeutic opportunities. Trends Cell Biol..

[42] Kajiwara C (2018). p63-Dependent Dickkopf3 expression promotes esophageal cancer cell proliferation via CKAP4. Cancer Res..

[43] Smirnov A (2019). ZNF185 is a p63 target gene critical for epidermal differentiation and squamous cell carcinoma development. Oncogene.

[44] Lambert AW (2022). ΔNp63/p73 drive metastatic colonization by controlling a regenerative epithelial stem cell program in quasi-mesenchymal cancer stem cells. Dev. Cell.

[45] Lee KB (2014). p63-Mediated activation of the β-catenin/c-Myc signaling pathway stimulates esophageal squamous carcinoma cell invasion and metastasis. Cancer Lett..

[46] Baslan T (2022). Ordered and deterministic cancer genome evolution after p53 loss. Nature.

[47] Kennedy MC, Lowe SW (2022). Mutant p53: it’s not all one and the same. Cell Death Differ..

[48] Liu T (2022). Computational identification of preneoplastic cells displaying high stemness and risk of cancer progression. Cancer Res..

[49] Hao Y (2021). Integrated analysis of multimodal single-cell data. Cell.

[50] Hafemeister C, Satija R (2019). Normalization and variance stabilization of single-cell RNA-seq data using regularized negative binomial regression. Genome Biol..

[51] Korsunsky I (2019). Fast, sensitive and accurate integration of single-cell data with Harmony. Nat. Methods.

[52] Hanzelmann S, Castelo R, Guinney J (2013). GSVA: gene set variation analysis for microarray and RNA-seq data. BMC Bioinforma..

[53] Subramanian A (2005). Gene set enrichment analysis: a knowledge-based approach for interpreting genome-wide expression profiles. Proc. Natl Acad. Sci. USA.

[54] Ritchie ME (2015). limma powers differential expression analyses for RNA-sequencing and microarray studies. Nucleic Acids Res..

[55] Pham D (2023). Robust mapping of spatiotemporal trajectories and cell-cell interactions in healthy and diseased tissues. Nat Commun.

[56] Quinn TP, Erb I, Richardson MF, Crowley TM (2018). Understanding sequencing data as compositions: an outlook and review. Bioinformatics.

[57] Jin S (2021). Inference and analysis of cell-cell communication using CellChat. Nat. Commun..

[58] Liu W (2021). VAV2 is required for DNA repair and implicated in cancer radiotherapy resistance. Signal Transduct. Target Ther..

[59] Ashley GR, Grace OC, Vanpoucke G, Thomson AA (2010). Identification of EphrinB1 expression in prostatic mesenchyme and a role for EphB-EphrinB signalling in prostate development. Differentiation.

[60] Popov C, Kohler J, Docheva D (2015). Activation of EphA4 and EphB2 reverse signaling restores the age-associated reduction of self-renewal, migration, and actin turnover in human tendon stem/progenitor cells. Front. Aging Neurosci..

[61] Su Q (2021). Sanguinarine combats hypoxia-induced activation of EphB4 and HIF-1α pathways in breast cancer. Phytomedicine.

