# Supplementary material for: Aberrant epithelial cell interaction promotes esophageal squamous-cell carcinoma development and progression
Source: Signal Transduct Target Ther. 2023 Dec 15;8:453. doi: 10.1038/s41392-023-01710-2 (PMC10721848; doi:10.1038/s41392-023-01710-2)
Supplement: Supplementary file 1 — Supplementary Materials [file 41392_2023_1710_MOESM1_ESM.docx]

Supplementary Materials for

**Aberrant epithelial cell interaction promotes esophageal squamous-cell carcinoma development and progression**

Liping Chen^1,6^, Shihao Zhu^1,6^, Tianyuan Liu^1^, Xuan Zhao^1^, Tao Xiang^1^, Xiao Hu^1^, Chen Wu^1,2,3,5,*^, and Dongxin Lin^1,2,3,4,*^

Correspondence to: lindx@cicams.ac.cn (D.L.), chenwu@cicams.ac.cn (C.W.)

**This PDF file includes:**

Supplementary Figures S1 to S8

Supplementary Tables S1 to S8


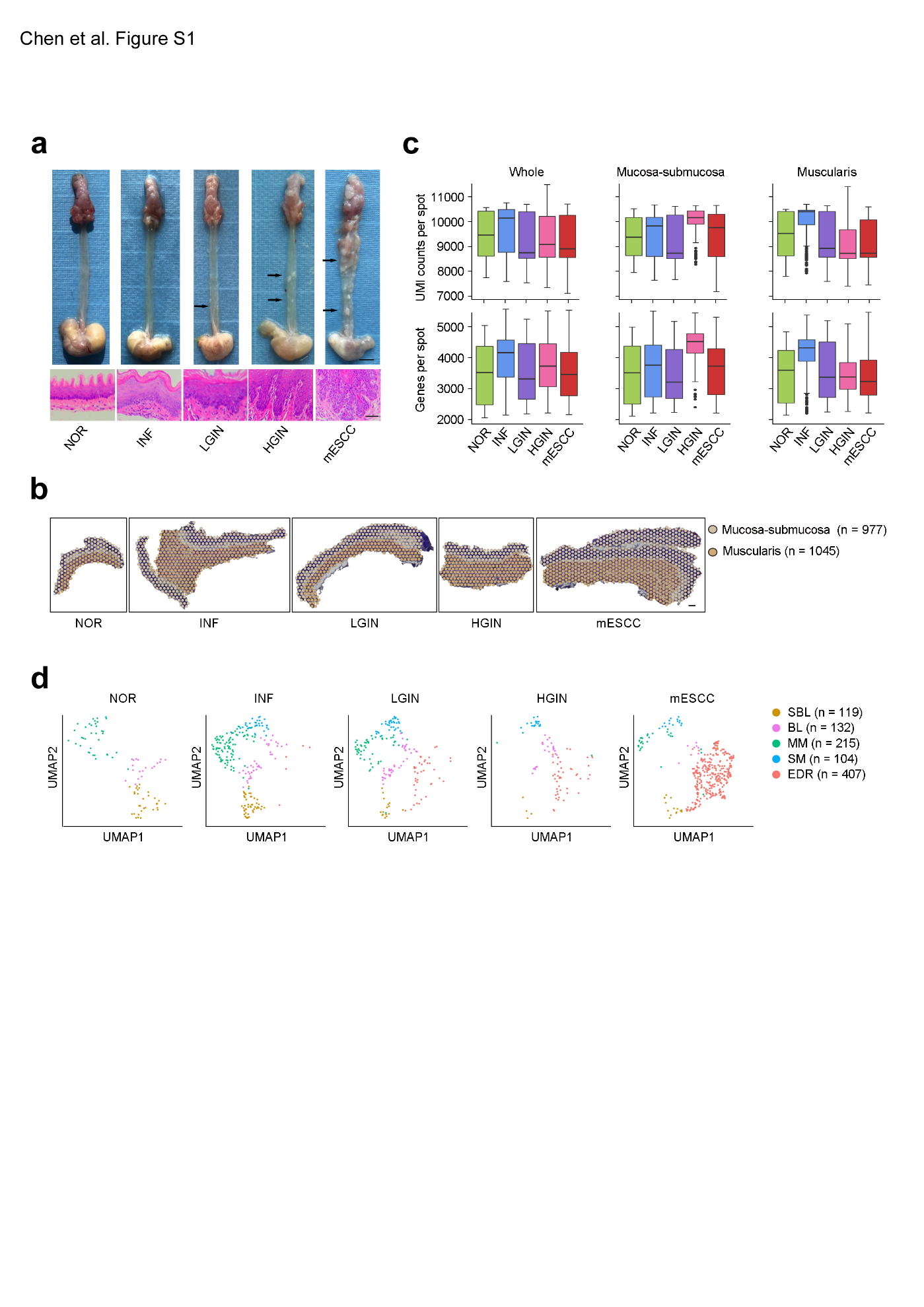


**Figure S1. Mouse ESCC model and spatial transcriptome data analysis related to Figure 1.** (**a**) Representative images of the mouse esophagus and the histopathology (H&E staining) of 5 disease stage samples. Scale bar on upper panel, 5 mm and lower panel, 50 μm. (**b**) Spatial plots showing distribution of all spots in 5 disease stage samples divided into mucosa-submucosa and muscularis. Scale bar, 200 μm. (**c**) Boxplots of the unique molecule identifier (UMI) counts (*upper panel*) and gene counts (*lower panel*) separated by tissue types after quality control. Shown are median and 25th to 75th percentile distribution with 1.5× quantile range represented by whiskers. (**d**) UMAP plots showing unbiased clustering of mucosa-submucosa spots in each of 5 disease stage samples.


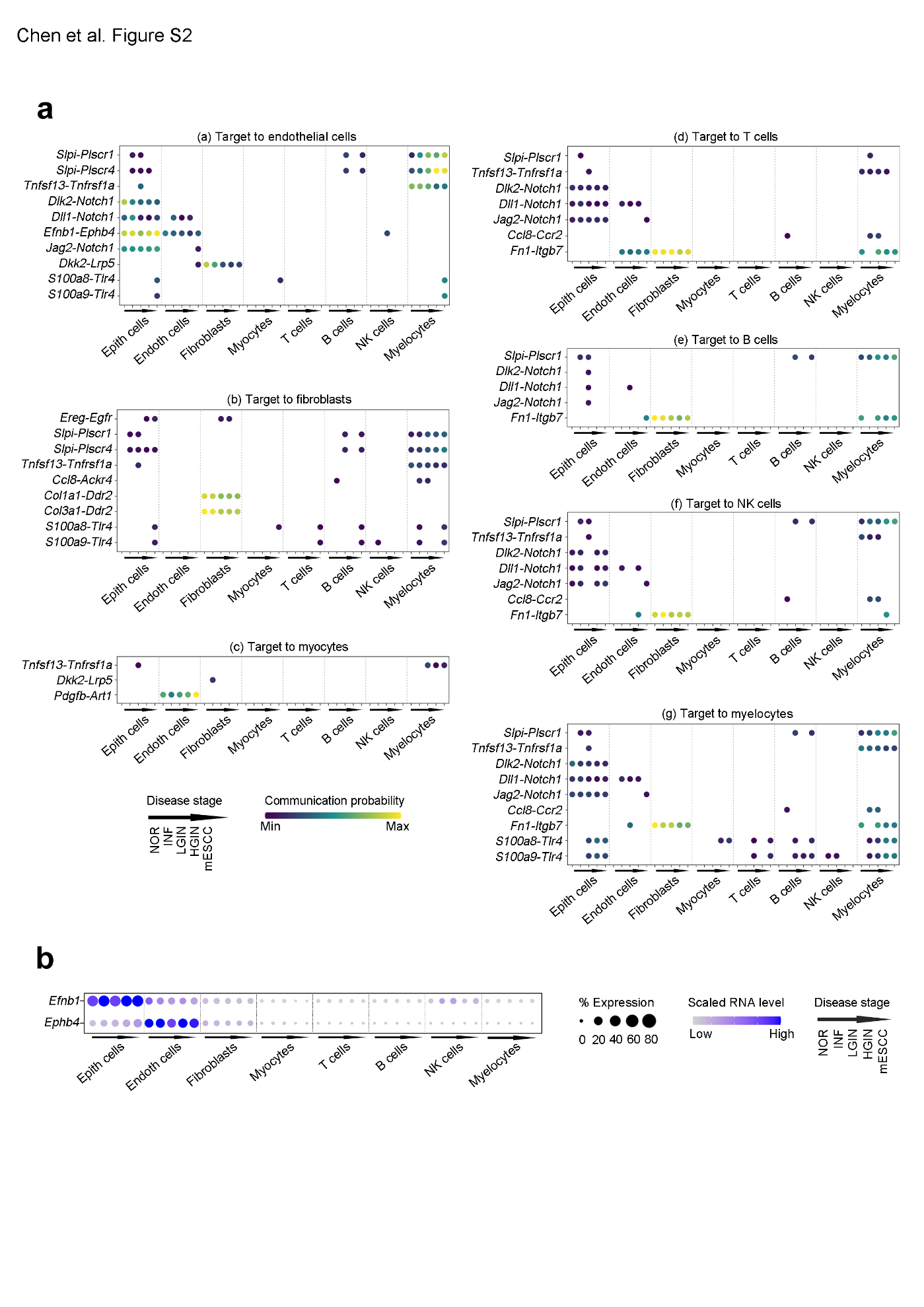


**Figure S2. Cell-cell interactions among various cell types in multistage mouse tissue samples related to Figure 2.** (**a**) Bubble plots of interaction of tissue region-specific ligand-receptor (LR) gene pairs among 8 cell types that target to different cell types from scRNA-seq data (CRA002118). All dots represented are statistically significant (*P* < 0.05). (**b**) Dot plot of the average expression level and the expressed cell percentage of *Efnb1* and *Ephb4* in 8 cell types from scRNA-seq data (CRA002118).


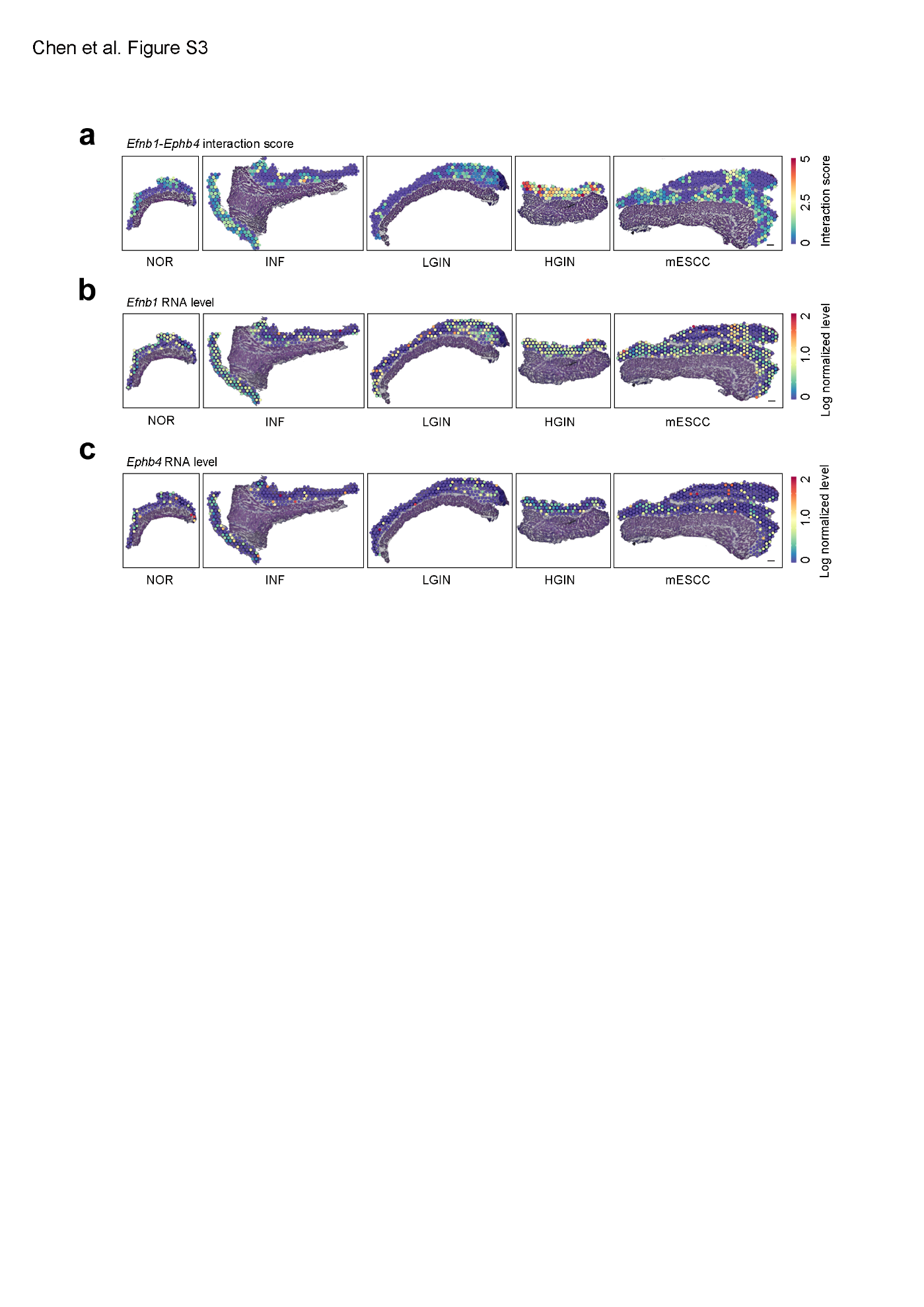


**Figure S3. Spatiotemporal interaction and expression of *Efnb1* and *Ephb4* in mouse esophageal tissues related to Figure 2.** (**a**) Spatial plots showing *Efnb1*-*Ephb4* interaction score in 5 disease stage samples. Scale bar, 200 μm. (**b, c**) Spatial plots showing RNA levels of *Efnb1* (**b**) and *Ephb4* (**c**) in 5 disease stage samples. Scale bar, 200 μm.


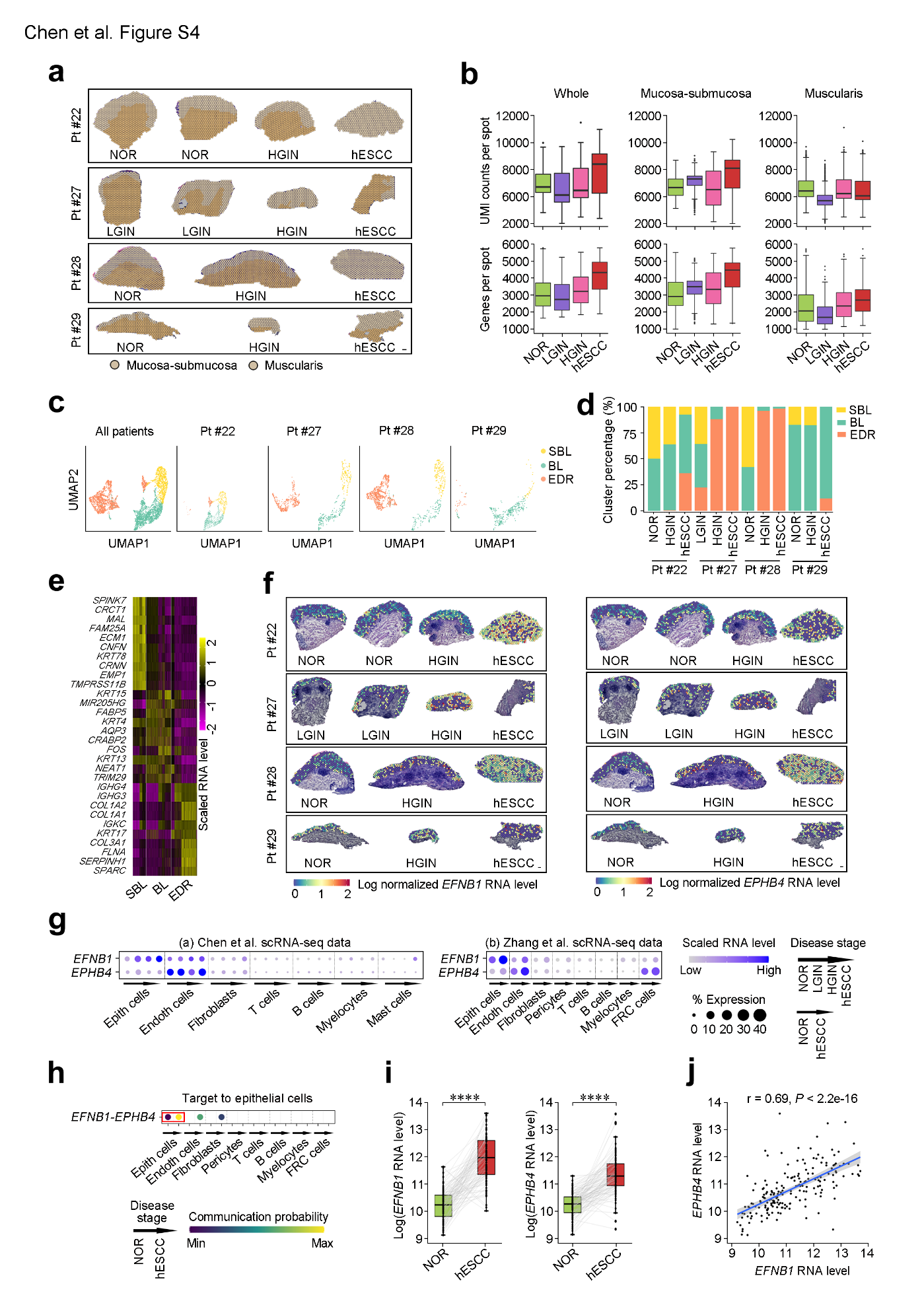


**Figure S4. Aberrant *EFNB1*-*EPHB4* interactions among epithelial cells facilitate hESCC development related to Figure 3.** (**a**) Spatial plots showing distribution of all spots in 4 disease stage samples divided into mucosa-submucosa and muscularis. Scale bar, 200 μm. (**b**) Boxplots of the unique molecule identifier (UMI) counts (*upper panel*) and gene counts (*lower panel*) separated by tissue types. Shown are median and 25th to 75th percentile distribution with 1.5× quantile range represented by whiskers. (**c**) UMAP plots showing unbiased clustering of mucosa-submucosa spots in all and each of 4 ESCC patients. (**d**) Stacked histogram showing the composition of 3 tissue regions in 4 disease stage samples from 4 ESCC patients. (**e**) Heatmap of scaled and normalized RNA levels of the top 10 highly expressed genes for 3 tissue regions. (**f**) Spatial plots showing *EFNB1* (*left panel*) and *EPHB4* (*right panel*) RNA levels in 4 disease stage samples from 4 ESCC patients. Scale bar, 200 μm. (**g**) Dot plots of the average expression levels and the expressed cell percentages of *EFNB1* and *EPHB4* in various cell types from scRNA-seq data sets (HRA000776, *left panel* and GSE160269, *right panel*). (**h**) Bubble plot showing *EFNB1*-*EPHB4* interaction between 8 cell types and epithelial cells from scRNA-seq data (GSE160269). *EFNB1*-*EPHB4* interaction probability between epithelial cells is higher in hESCC than NOR. (**i**) Boxplots of *EFNB1* and *EPHB4* RNA levels of paired NOR and hESCC tissues in bulk transcriptome data (HRA000003). Shown are median and 25th to 75th percentile distribution with 1.5× quantile range represented by whiskers. ****, *P* < 0.0001 of Wilcoxon rank-sum test. (**j**) Spearman correlation between *EFNB1* and *EPHB4* RNA levels in bulk transcriptome data (HRA000003). Shade represents 95% confidence interval.


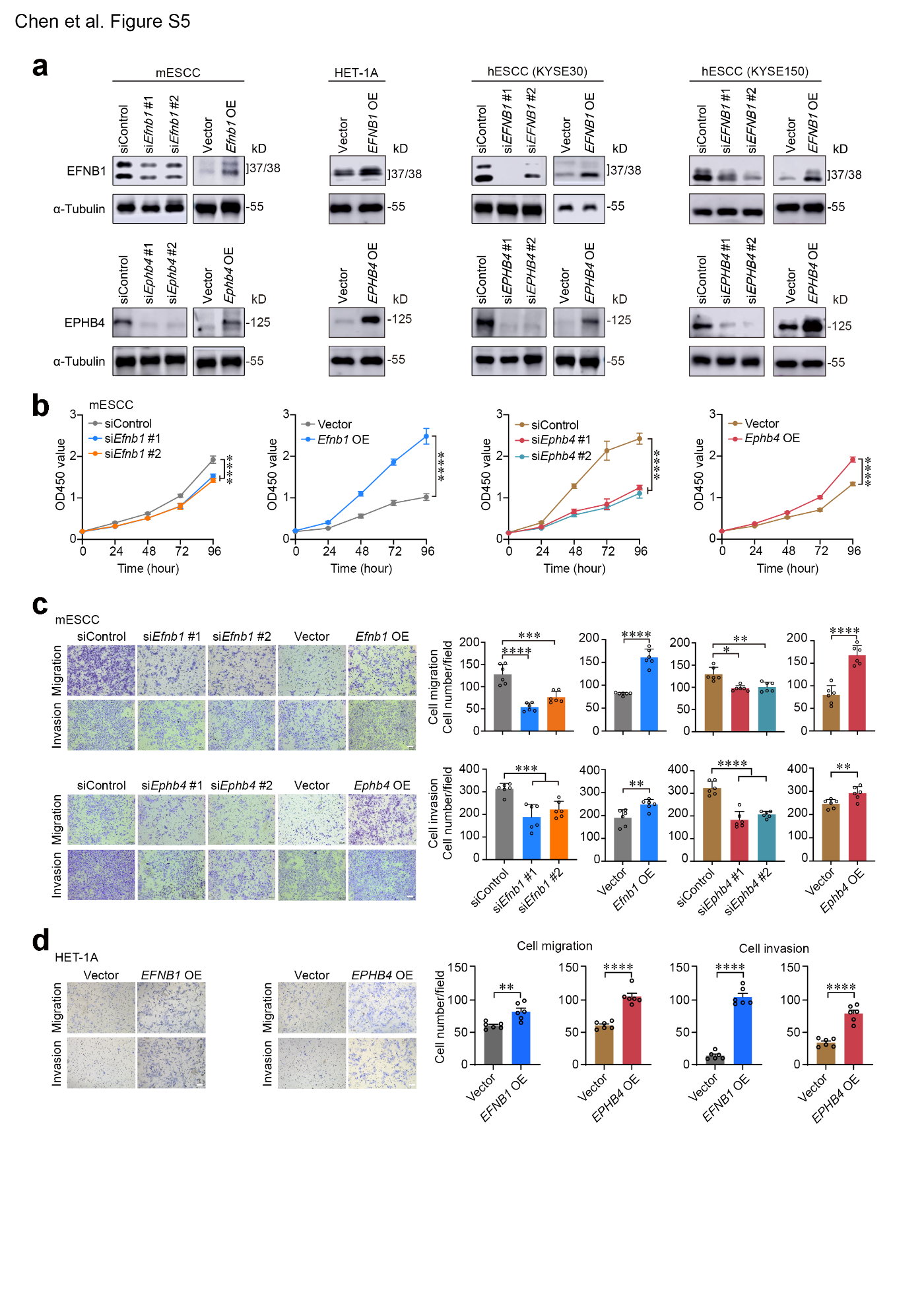


**Figure S5. The effects of EFNB1-EPHB4 interaction alterations on malignant phenotypes in mESCC and human normal esophageal epithelial cells related to Figure 4.** (**a**) Western blotting analysis of EFNB1 and EPHB4 protein levels in *EFNB1* and *EPHB4* knockdown or overexpression mESCC, HET-1A, KYSE30 and KYSE150. (**b**) The effects of forced *Efnb1* and *Ephb4* expression change on mESCC cell proliferation. Each point represents mean ± S.E.M. from 3 independent experiments and each had 6 replications. ****, *P* < 0.0001 of Student’s t-test. (**c**) The effects of forced *Efnb1* and *Ephb4* expression change on mESCC cell migration and invasion. Left panel shows representative transwell images and the right panel shows quantitation statistics. Scale bar, 100 μm. Data are mean ± S.E.M. from 3 independent experiments and each had 2 replications. *, *P* < 0.05; **, *P* < 0.01; ***, *P* < 0.001 and ****, *P* < 0.0001 of Student’s t-test. (**d**) Effects of forced *EFNB1* and *EPHB4* overexpression on HET-1A cell migration and invasion. *Left panel*, representative transwell images; *right panel*, quantitation statistics. Scale bar, 100 μm. Data are mean ± S.E.M. from 3 independent experiments and each had 2 replications. **, *P* < 0.01 and ****, *P* < 0.0001 of Student’s t-test.


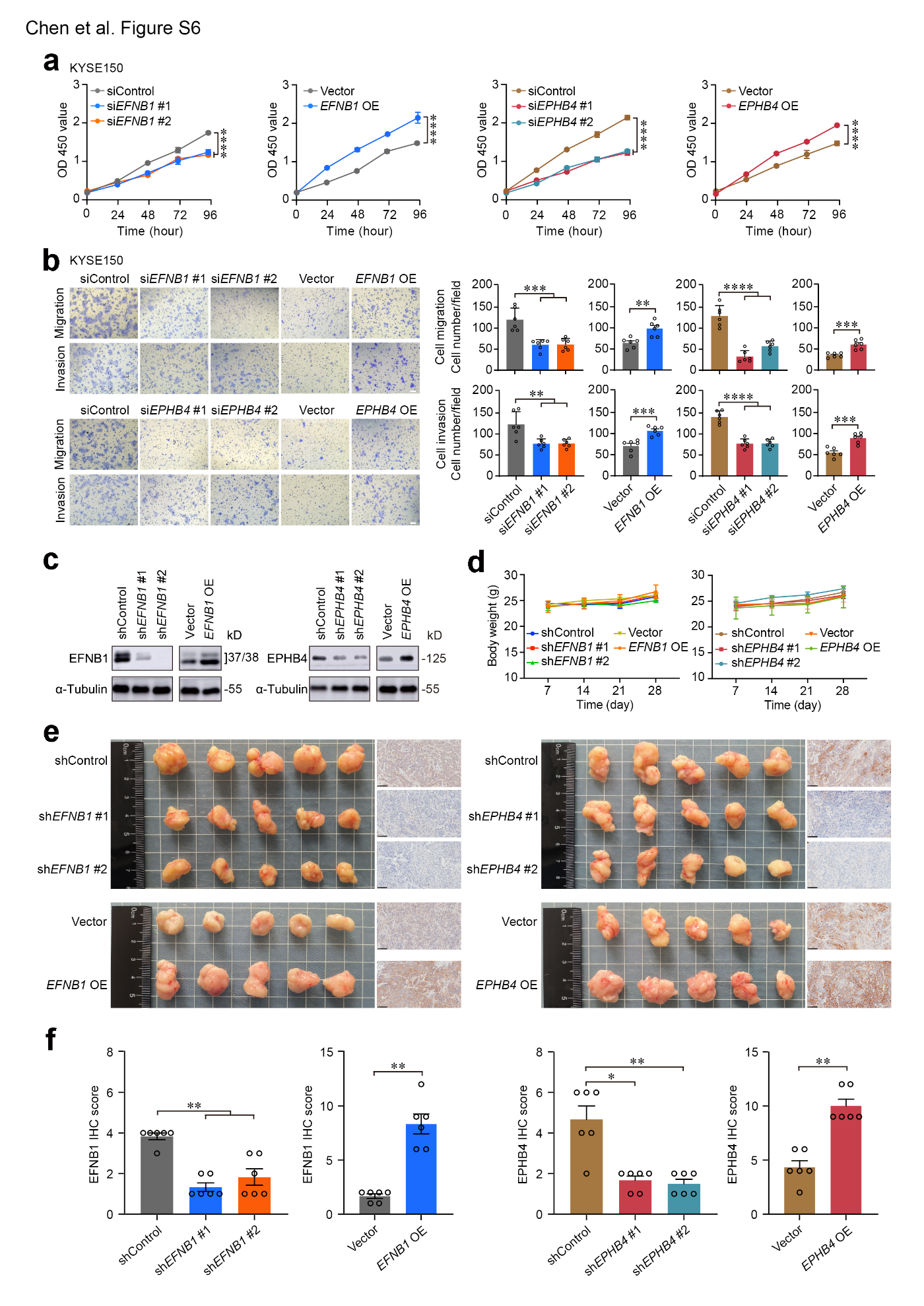


**Figure S6. Effects of aberrant EFNB1-EPHB4 interaction on malignant phenotypes of hESCC related to Figure 4.** (**a**) Effects of forced *EFNB1* and *EPHB4* expression change on hESCC KYSE150 cell proliferation. (**b**) Effects of forced *EFNB1* and *EPHB4* expression change on hESCC KYSE150 cell migration and invasion. *Left panel*, representative transwell images; *right panel*, quantitation statistics. Scale bar, 100 μm. (**c**) Western blot assays of EFNB1 (*left panel*) and EPHB4 (*right panel*) protein levels in *EFNB1* and *EPHB4* stable knockdown and overexpression hESCC cells (KYSE30). (**d**) Body weight curves of NSG mice bearing ESCC xenograft showing no significant difference during the experiment course. Data are mean ± S.E.M. from 5 animals. (**e**) General images and corresponding tissue IHC staning images of hESCC (KYSE30) xenografts with forced *EFNB1* (*left panel*) and *EPHB4* (*right panel*) expression change in NSG mice at the end of the experiment. (**f**) Barplots showing IHC score of EFNB1 and EPHB4 in hESCC (KYSE30) xenograft tissue sections with forced *EFNB1* (*left panel*) and *EPHB4* (*right panel*) expression change. Data in this figure are mean ± S.E.M. *, *P* < 0.05 and **, *P* < 0.01 of Student’s t-test.


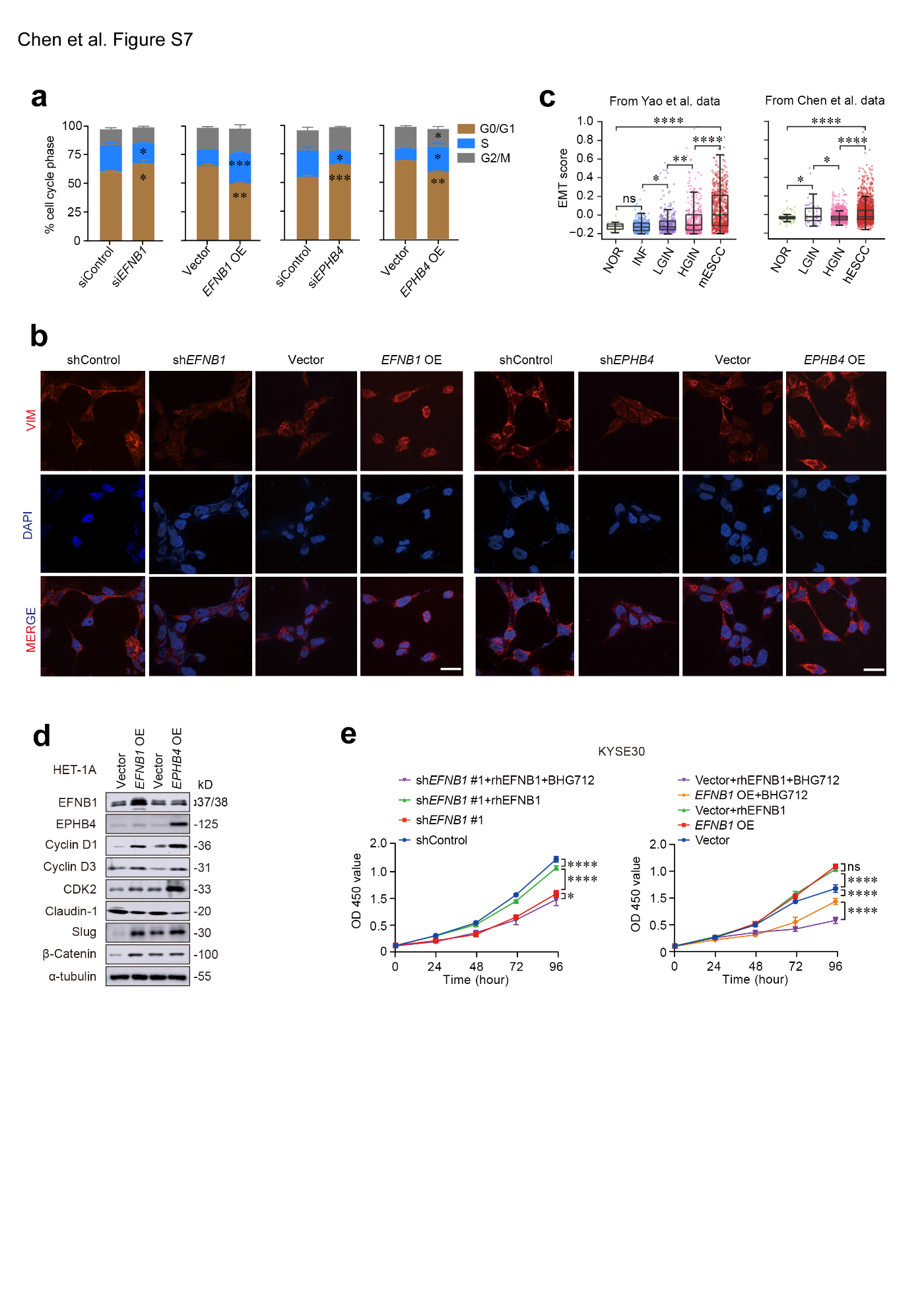


**Figure S7. Aberrant EFNB1-EPHB4 interaction promotes cell cycle and epithelial to mesenchymal transition related to Figure 5.** (**a**) Stacked histogram showing the composition of cell cycle phases of hESCC cells (KYSE30) with *EFNB1* (*left panel*) and *EPHB4* (*right panel*) knockdown and overexpression. *, *P* < 0.05; **, *P* < 0.01; ***, *P* < 0.001 and ns, not significant of Student’s t-test. (**b**) Images of VIM immunostaining in hESCC cells (KYSE30) with *EFNB1* and *EPHB4* knockdown and overexpression. The nuclei were stained with DAPI. Scale bar, 30 μm. (**c**) EMT score in epithelial cells from scRNA-seq data of mouse (CRA002118, *left panel*) and human (HRA000776, *right panel*). Shown are median and 25th to 75th percentile distribution with 1.5× quantile range represented by whiskers. Wilcoxon rank-sum test, *, *P* < 0.05; **, *P* < 0.01; ****, *P* < 0.0001; and ns, not significant. (**d**) Western blotting analysis of cell cycle markers (Cyclin D1, Cyclin D3, and CDK2) and EMT markers (Claudin-1, SLUG, and β-Catenin) in HET-1A cells with *EFNB1* or *EPHB4* overexpression. Each Western blot assay had 3 biological repeats. (**e**) Effects of recombinant human EFNB1 protein (rhEFNB1) and the EPHB4 inhibitor (BHG712) on KYSE30 cell proliferation. Each data point represents mean ± S.E.M. from 3 independent experiments and each had 6 replications. ****, *P* < 0.0001 and ns, not significant of Student’s t-test.


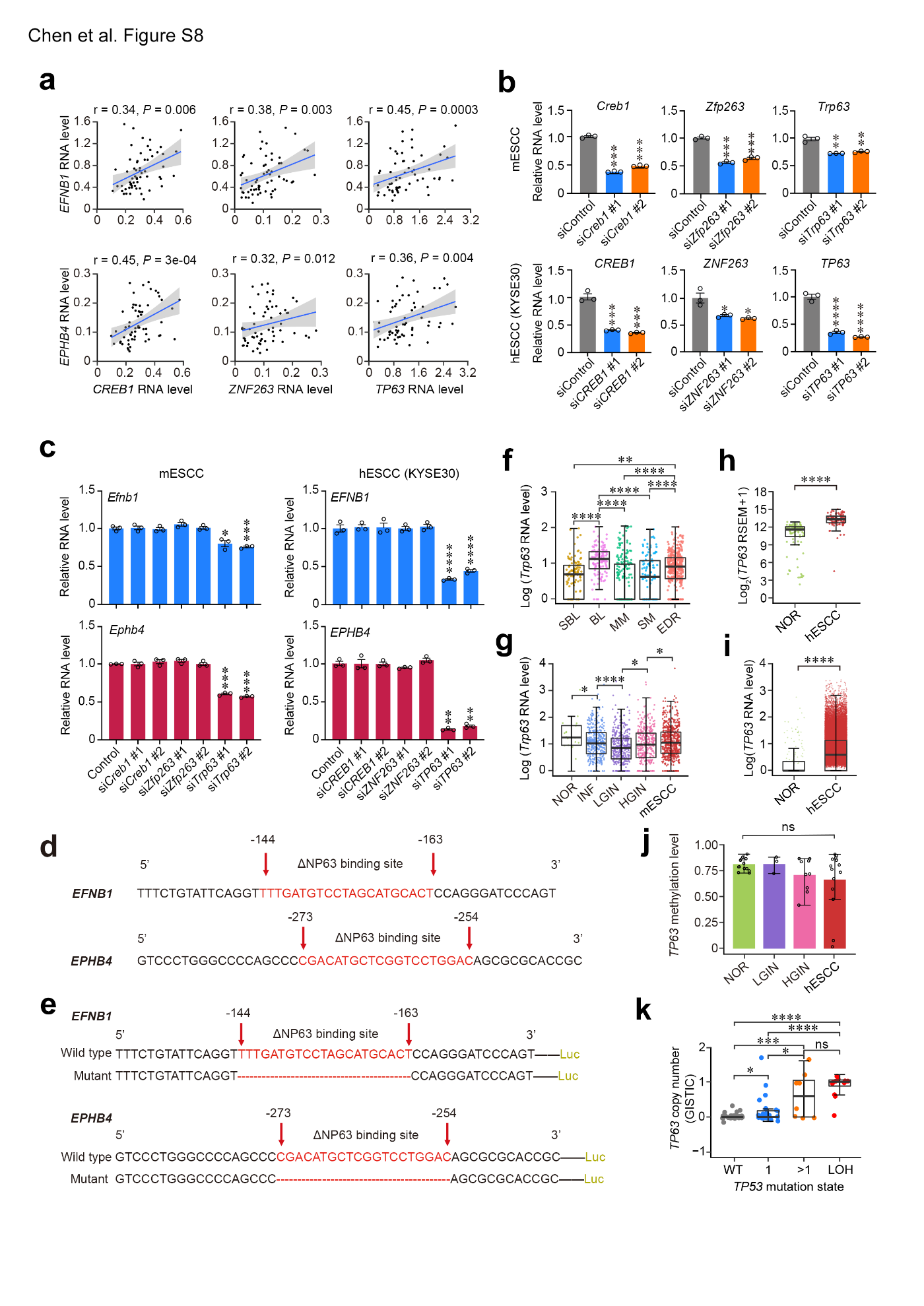


**Figure S8. Overexpression of ∆NP63 due to TP53 dysfunction during ESCC development causes aberrant EFNB1-EPHB4 interaction related to Figure 6.** (**a**) Spearman correlations between the RNA levels of *CREB1*/*ZNF263*/*TP63* and *EFNB1*/*EPHB4* in epithelial cells from scRNA-seq data (GSE160269). (**b**) Histograms showing real time quantitative PCR results verified the mRNA levels of *CREB1*, *ZNF263*, and *TP63* in mESCC and hESCC (KYSE30) treated with siRNAs of corresponding genes. Data are mean ± S.E.M., *, *P* < 0.05; **, *P* < 0.01; ***, *P* < 0.001; and ****, *P* < 0.0001 of Student’s t-test. (**c**) The effects of knockdown of 3 interested TFs on *EFNB1* and *EPHB4* RNA levels in hESCC cells (KYSE30). Data are mean ± S.E.M. from 3 biological replications. *, *P* < 0.05; **, *P* < 0.01; ***, *P* < 0.001; and ****, *P* < 0.0001 of Wilcoxon rank-sum test. (**d**) Promoter sequences of human *EFNB1* (*upper panel*) and *EPHB4* (*lower panel*) with transcription factor ΔNP63 binding site (indicated by arrows). (**e**) The sequences of truncated wild-type or TP63-binding-motif deletion mutant promoter regions of human *EFNB1* (*upper panel*) and *EPHB4* (*lower panel*) for the pGL4 reporter plasmids constructions. (**f**) Boxplot of RNA level of *TP63* in 5 tissue regions in mouse spatial transcriptome data. Shown are median and 25th to 75th percentile distribution with 1.5× quantile range represented by whiskers. (**g**) The RNA level of *TP63* in epithelial cells from mouse scRNA-seq data (CRA002118). Shown are median and 25th to 75th percentile distribution with 1.5× quantile range represented by whiskers. (**h**) The difference of *TP63* RNA levels in epithelial cells of normal and hESCC samples analyzed by bulk RNA-seq (HRA000003 data). (**i**) The difference of *TP63* RNA levels in epithelial cells of normal and hESCC samples analyzed by scRNA-seq (GSE160269 data). Data in **f–i** are median and 25th to 75th percentile distribution with 1.5 × quantile range represented by whiskers. *, *P* < 0.05; **, *P* < 0.01; and ****, *P* < 0.0001 of Wilcoxon rank-sum test. (**j**) Histogram showing DNA methylation level of *TP63* promoter in NOR (n = 14), LGIN (n = 3), HGIN (n = 9), and hESCC (n = 14). Data are mean ± S.E.M. ns, not significant of Wilcoxon rank-sum test. (**k**) Boxplot showing GISTIC copy number of *TP63* in clones with different *TP53* mutation states of wild type (WT; n = 35), 1 (n = 24), >1 (n = 8), and loss of heterozygosity (LOH; n = 12). *, *P* < 0.05; ***, *P* < 0.001; ****, *P* < 0.0001; and ns, not significant of Wilcoxon rank-sum test.

| **Table S1. Spot counts of different tissue regions in mouse spatial transcriptome data** | | | | | | | | |
| --- | --- | --- | --- | --- | --- | --- | --- | --- |
| *Disease  stage | Mucosa/  submucosa | Suprabasal layer | Basal layer | Muscularis mucosa | Submucosa | ESCC development region | Muscularis | Total |
| NOR | 87 | 32 | 19 | 35 | 1 | 0 | 69 | 156 |
| INF | 225 | 50 | 40 | 94 | 36 | 5 | 278 | 503 |
| LGIN | 185 | 17 | 42 | 52 | 35 | 39 | 157 | 342 |
| HGIN | 94 | 4 | 24 | 3 | 17 | 46 | 209 | 303 |
| ESCC | 386 | 16 | 7 | 31 | 15 | 317 | 332 | 718 |
| *NOR, normal; INF, inflammation; LGIN, low-grade intraepithelial neoplasia; HGIN, high-grade intraepithelial neoplasia; ESCC, esophageal squamous cell carcinoma. | | | | | | | | |

| **Table S2. Differentially expressed genes of 5 tissue regions of mouse spatial transcriptome data** | | | |
| --- | --- | --- | --- |
| Tissue region | Gene | Log2 (fold change) | *P*-value |
| Suprabasal layer | *Rptn* | 2.33 | 7.06E-57 |
| Suprabasal layer | *Lce3f* | 2.33 | 2.58E-51 |
| Suprabasal layer | *Lce3c* | 1.84 | 3.58E-50 |
| Suprabasal layer | *Fam25c* | 1.53 | 9.08E-50 |
| Suprabasal layer | *Crct1* | 1.57 | 1.61E-49 |
| Suprabasal layer | *Lor* | 2.17 | 2.33E-48 |
| Suprabasal layer | *Lce3a* | 1.72 | 1.53E-46 |
| Suprabasal layer | *Krt78* | 2.10 | 9.92E-46 |
| Suprabasal layer | *Lce3e* | 2.00 | 9.98E-46 |
| Suprabasal layer | *Lce3b* | 1.81 | 1.34E-44 |
| Suprabasal layer | *Slpi* | 1.55 | 1.44E-44 |
| Suprabasal layer | *Cdsn* | 1.66 | 4.52E-43 |
| Suprabasal layer | *Asprv1* | 2.34 | 1.01E-42 |
| Suprabasal layer | *Lce1a2* | 1.64 | 5.65E-42 |
| Suprabasal layer | *Fetub* | 2.07 | 5.88E-42 |
| Suprabasal layer | *Crisp1* | 1.07 | 3.89E-40 |
| Suprabasal layer | *Lce1a1* | 1.76 | 8.09E-40 |
| Suprabasal layer | *Lce1c* | 1.64 | 1.36E-38 |
| Suprabasal layer | *Kprp* | 1.94 | 8.98E-38 |
| Suprabasal layer | *Lce1d* | 1.49 | 1.30E-37 |
| Suprabasal layer | *Aif1l* | 0.71 | 1.56E-37 |
| Suprabasal layer | *Cnfn* | 0.95 | 1.68E-37 |
| Suprabasal layer | *Lce1e* | 1.27 | 1.10E-36 |
| Suprabasal layer | *Lce1b* | 1.63 | 7.56E-36 |
| Suprabasal layer | *Hrnr* | 1.42 | 7.98E-35 |
| Suprabasal layer | *Lypd5* | 1.31 | 2.13E-34 |
| Suprabasal layer | *Cysrt1* | 1.27 | 6.78E-34 |
| Suprabasal layer | *Lce1k* | 1.02 | 2.56E-33 |
| Suprabasal layer | *Klk14* | 1.11 | 3.23E-33 |
| Suprabasal layer | *Sprr3* | 1.19 | 7.80E-33 |
| Suprabasal layer | *2210017I01Rik* | 0.40 | 2.62E-32 |
| Suprabasal layer | *Gpld1* | 1.17 | 2.99E-32 |
| Suprabasal layer | *Krt13* | 0.79 | 3.00E-32 |
| Suprabasal layer | *Krt4* | 1.05 | 1.98E-31 |
| Suprabasal layer | *Acp7* | 1.20 | 5.12E-31 |
| Suprabasal layer | *Lce1j* | 0.69 | 2.14E-30 |
| Suprabasal layer | *Lce1i* | 1.39 | 2.17E-30 |
| Suprabasal layer | *Cpa4* | 1.17 | 1.85E-29 |
| Suprabasal layer | *Defb4* | 1.16 | 1.88E-29 |
| Suprabasal layer | *Tmem45a2* | 0.66 | 4.25E-29 |
| Suprabasal layer | *Lce1g* | 1.18 | 1.01E-28 |
| Suprabasal layer | *Rnf227* | 0.58 | 2.09E-28 |
| Suprabasal layer | *2310050C09Rik* | 1.16 | 3.96E-28 |
| Suprabasal layer | *Lce1f* | 1.21 | 6.59E-28 |
| Suprabasal layer | *Ecm1* | 1.17 | 6.60E-28 |
| Suprabasal layer | *2300002M23Rik* | 1.26 | 9.12E-27 |
| Suprabasal layer | *Krtdap* | 0.57 | 1.42E-26 |
| Suprabasal layer | *Il18* | 0.91 | 2.30E-26 |
| Suprabasal layer | *Nccrp1* | 0.95 | 5.63E-26 |
| Suprabasal layer | *Sbsn* | 0.67 | 3.22E-25 |
| Suprabasal layer | *Klk9* | 1.09 | 3.53E-25 |
| Suprabasal layer | *Eps8l1* | 1.15 | 3.69E-25 |
| Suprabasal layer | *Anxa9* | 0.90 | 3.90E-25 |
| Suprabasal layer | *Slc46a2* | 0.76 | 4.05E-25 |
| Suprabasal layer | *Smpd3* | 1.15 | 7.98E-25 |
| Suprabasal layer | *Krt80* | 1.18 | 8.64E-25 |
| Suprabasal layer | *Tgm3* | 1.05 | 1.13E-24 |
| Suprabasal layer | *Ide* | 1.03 | 1.20E-24 |
| Suprabasal layer | *Klk7* | 1.13 | 2.77E-24 |
| Suprabasal layer | *Gm2a* | 1.04 | 4.89E-24 |
| Suprabasal layer | *Ppl* | 1.01 | 8.82E-24 |
| Suprabasal layer | *Hopx* | 0.98 | 1.33E-23 |
| Suprabasal layer | *Ada* | 2.30 | 1.35E-23 |
| Suprabasal layer | *Il1f5* | 0.74 | 4.92E-23 |
| Suprabasal layer | *Ctsd* | 0.56 | 5.74E-23 |
| Suprabasal layer | *Dgat2* | 0.84 | 8.71E-23 |
| Suprabasal layer | *Fam57a* | 1.01 | 1.61E-22 |
| Suprabasal layer | *Aloxe3* | 1.04 | 1.78E-22 |
| Suprabasal layer | *Calml3* | 1.22 | 2.10E-22 |
| Suprabasal layer | *Ereg* | 0.56 | 2.46E-22 |
| Suprabasal layer | *Serpina9* | 0.71 | 4.42E-22 |
| Suprabasal layer | *Yod1* | 0.74 | 5.58E-22 |
| Suprabasal layer | *Lce1h* | 0.71 | 1.02E-21 |
| Suprabasal layer | *Spink5* | 0.76 | 1.24E-21 |
| Suprabasal layer | *Lce1l* | 0.62 | 1.93E-21 |
| Suprabasal layer | *Alox12b* | 0.92 | 1.94E-21 |
| Suprabasal layer | *Bglap3* | 1.02 | 3.64E-21 |
| Suprabasal layer | *P4hb* | 0.65 | 4.92E-21 |
| Suprabasal layer | *Esyt3* | 0.60 | 1.03E-20 |
| Suprabasal layer | *Calm4* | 0.80 | 1.12E-20 |
| Suprabasal layer | *Flg* | 0.48 | 1.58E-20 |
| Suprabasal layer | *Glrx* | 0.91 | 2.22E-20 |
| Suprabasal layer | *Psapl1* | 0.90 | 4.99E-20 |
| Suprabasal layer | *Xkrx* | 0.40 | 7.90E-20 |
| Suprabasal layer | *Rnf225* | 0.59 | 2.79E-19 |
| Suprabasal layer | *Ces2f* | 0.76 | 3.39E-19 |
| Suprabasal layer | *Serpinb12* | 0.81 | 4.27E-19 |
| Suprabasal layer | *Dnase1l2* | 0.74 | 8.18E-19 |
| Suprabasal layer | *Dkkl1* | 0.70 | 1.79E-18 |
| Suprabasal layer | *Tmprss13* | 0.81 | 2.11E-18 |
| Suprabasal layer | *Padi1* | 0.53 | 2.75E-18 |
| Suprabasal layer | *Ripor3* | 0.48 | 2.83E-18 |
| Suprabasal layer | *Serpina12* | 0.54 | 3.03E-18 |
| Suprabasal layer | *Rnf222* | 0.40 | 3.59E-18 |
| Suprabasal layer | *Nkpd1* | 0.80 | 4.13E-18 |
| Suprabasal layer | *Rnf223* | 0.31 | 7.76E-18 |
| Suprabasal layer | *Elovl4* | 0.63 | 2.37E-17 |
| Suprabasal layer | *Defb6* | 0.40 | 2.82E-17 |
| Suprabasal layer | *Slc37a2* | 0.63 | 3.11E-17 |
| Suprabasal layer | *Dusp14* | 0.90 | 4.09E-17 |
| Suprabasal layer | *Gsdma* | 0.82 | 5.49E-17 |
| Suprabasal layer | *Gpsm1* | 0.62 | 4.17E-16 |
| Suprabasal layer | *Dennd1b* | 0.47 | 4.42E-16 |
| Suprabasal layer | *Wfdc12* | 0.89 | 4.77E-16 |
| Suprabasal layer | *Trex2* | 0.89 | 6.87E-16 |
| Suprabasal layer | *Rnf208* | 0.75 | 1.33E-15 |
| Suprabasal layer | *Cst6* | 0.73 | 1.40E-15 |
| Suprabasal layer | *Gramd1c* | 0.48 | 1.48E-15 |
| Suprabasal layer | *Prss8* | 0.60 | 2.71E-15 |
| Suprabasal layer | *Sorbs1* | 0.32 | 4.37E-15 |
| Suprabasal layer | *Lnx1* | 0.28 | 7.72E-15 |
| Suprabasal layer | *Aldh3a1* | 0.85 | 7.88E-15 |
| Suprabasal layer | *Dsg1a* | 0.76 | 1.09E-14 |
| Suprabasal layer | *Fndc9* | 0.38 | 1.23E-14 |
| Suprabasal layer | *Nipal4* | 0.76 | 1.49E-14 |
| Suprabasal layer | *Gramd3* | 0.52 | 1.60E-14 |
| Suprabasal layer | *Fmo2* | 0.57 | 2.00E-14 |
| Suprabasal layer | *Cyp2s1* | 0.92 | 2.18E-14 |
| Suprabasal layer | *Prdm1* | 0.43 | 2.24E-14 |
| Suprabasal layer | *Fcho1* | 0.32 | 3.25E-14 |
| Suprabasal layer | *Serpinb3a* | 0.63 | 3.43E-14 |
| Suprabasal layer | *Klk8* | 0.67 | 3.70E-14 |
| Suprabasal layer | *Atg9b* | 0.68 | 4.79E-14 |
| Suprabasal layer | *Gm94* | 0.66 | 4.87E-14 |
| Suprabasal layer | *Serpinb8* | 0.51 | 4.91E-14 |
| Suprabasal layer | *Tmem62* | 0.33 | 9.80E-14 |
| Suprabasal layer | *Porcn* | 0.62 | 1.09E-13 |
| Suprabasal layer | *Ces2g* | 0.67 | 1.15E-13 |
| Suprabasal layer | *Serpinb11* | 0.67 | 1.64E-13 |
| Suprabasal layer | *Acsbg1* | 0.68 | 3.12E-13 |
| Suprabasal layer | *Bpifa5* | 1.50 | 3.14E-13 |
| Suprabasal layer | *Otub2* | 0.49 | 3.90E-13 |
| Suprabasal layer | *Lce3d* | 0.29 | 3.91E-13 |
| Suprabasal layer | *Nrd1* | 0.65 | 4.67E-13 |
| Suprabasal layer | *Slc6a19* | 0.61 | 4.89E-13 |
| Suprabasal layer | *Clmn* | 0.36 | 7.20E-13 |
| Suprabasal layer | *Dmkn* | 0.34 | 9.48E-13 |
| Suprabasal layer | *Nlrp10* | 0.34 | 1.07E-12 |
| Suprabasal layer | *Lce1m* | 0.48 | 1.47E-12 |
| Suprabasal layer | *Lgalsl* | 0.63 | 1.58E-12 |
| Suprabasal layer | *Cited4* | 0.40 | 2.40E-12 |
| Suprabasal layer | *Eps8l2* | 0.73 | 2.47E-12 |
| Suprabasal layer | *Pcdh1* | 0.67 | 4.19E-12 |
| Suprabasal layer | *Pla2g3* | 0.48 | 6.71E-12 |
| Suprabasal layer | *Ankrd24* | 0.27 | 7.05E-12 |
| Suprabasal layer | *Slc26a9* | 0.44 | 1.00E-11 |
| Suprabasal layer | *Smpd1* | 0.66 | 1.04E-11 |
| Suprabasal layer | *Evpl* | 0.60 | 1.09E-11 |
| Suprabasal layer | *Lpin2* | 0.42 | 1.16E-11 |
| Suprabasal layer | *Lpin3* | 0.53 | 2.10E-11 |
| Suprabasal layer | *Srpk1* | 0.56 | 2.38E-11 |
| Suprabasal layer | *Cobl* | 0.53 | 2.61E-11 |
| Suprabasal layer | *9930012K11Rik* | 0.37 | 2.85E-11 |
| Suprabasal layer | *Hmox1* | 0.63 | 2.96E-11 |
| Suprabasal layer | *S100a14* | 0.56 | 3.33E-11 |
| Suprabasal layer | *Il1f8* | 0.44 | 5.88E-11 |
| Suprabasal layer | *Ugcg* | 0.58 | 7.97E-11 |
| Suprabasal layer | *Abca12* | 0.63 | 8.48E-11 |
| Suprabasal layer | *Cgref1* | 0.40 | 8.55E-11 |
| Suprabasal layer | *Prkch* | 0.53 | 9.42E-11 |
| Suprabasal layer | *Endod1* | 0.65 | 9.43E-11 |
| Suprabasal layer | *Pla2g4d* | 0.67 | 9.58E-11 |
| Suprabasal layer | *Pla2g4b* | 0.54 | 1.08E-10 |
| Suprabasal layer | *Clic3* | 0.71 | 1.22E-10 |
| Suprabasal layer | *Slc15a1* | 0.62 | 1.32E-10 |
| Suprabasal layer | *Abcg1* | 0.26 | 1.36E-10 |
| Suprabasal layer | *Usp54* | 0.40 | 1.57E-10 |
| Suprabasal layer | *Sptssb* | 0.69 | 1.74E-10 |
| Suprabasal layer | *Mab21l4* | 0.34 | 2.00E-10 |
| Suprabasal layer | *Prss27* | 0.66 | 2.03E-10 |
| Suprabasal layer | *Shb* | 0.60 | 2.35E-10 |
| Suprabasal layer | *Tmcc3* | 0.33 | 3.51E-10 |
| Suprabasal layer | *AA986860* | 0.43 | 3.51E-10 |
| Suprabasal layer | *2310043M15Rik* | 0.54 | 4.15E-10 |
| Suprabasal layer | *Hpse* | 0.47 | 4.16E-10 |
| Suprabasal layer | *S100a7a* | 0.66 | 5.13E-10 |
| Suprabasal layer | *Elmod1* | 0.32 | 5.26E-10 |
| Suprabasal layer | *Dhrs9* | 0.36 | 5.78E-10 |
| Suprabasal layer | *Rab11fip1* | 0.48 | 6.05E-10 |
| Suprabasal layer | *Gba2* | 0.44 | 6.13E-10 |
| Suprabasal layer | *Pim1* | 0.53 | 6.62E-10 |
| Suprabasal layer | *Cidea* | 0.32 | 8.04E-10 |
| Suprabasal layer | *Dlx3* | 0.35 | 8.14E-10 |
| Suprabasal layer | *Elovl1* | 0.47 | 9.24E-10 |
| Suprabasal layer | *Pax9* | 0.51 | 1.55E-09 |
| Suprabasal layer | *Tbc1d24* | 0.26 | 2.00E-09 |
| Suprabasal layer | *Dsc1* | 0.49 | 2.10E-09 |
| Suprabasal layer | *Gltp* | 0.47 | 2.25E-09 |
| Suprabasal layer | *Zfand6* | 0.57 | 2.56E-09 |
| Suprabasal layer | *Nt5c3* | 0.59 | 2.57E-09 |
| Suprabasal layer | *Gas7* | 0.42 | 4.10E-09 |
| Suprabasal layer | *Dbil5* | 0.51 | 5.18E-09 |
| Suprabasal layer | *Ptrh1* | 0.33 | 7.36E-09 |
| Suprabasal layer | *Ly6g6c* | 0.45 | 7.74E-09 |
| Suprabasal layer | *Ly6m* | 0.35 | 8.95E-09 |
| Suprabasal layer | *Il33* | 0.43 | 1.26E-08 |
| Suprabasal layer | *Casz1* | 0.51 | 1.28E-08 |
| Suprabasal layer | *Itprid2* | 0.51 | 1.77E-08 |
| Suprabasal layer | *Ank* | 0.52 | 2.47E-08 |
| Suprabasal layer | *Krt32* | 0.41 | 2.64E-08 |
| Suprabasal layer | *Klk11* | 0.48 | 3.03E-08 |
| Suprabasal layer | *Mapk13* | 0.57 | 3.61E-08 |
| Suprabasal layer | *Proser2* | 0.49 | 4.17E-08 |
| Suprabasal layer | *Cgn* | 0.33 | 4.65E-08 |
| Suprabasal layer | *Asah2* | 0.47 | 4.95E-08 |
| Suprabasal layer | *Slc19a2* | 0.44 | 5.52E-08 |
| Suprabasal layer | *Spns2* | 0.46 | 6.24E-08 |
| Suprabasal layer | *Nudt4* | 0.46 | 1.03E-07 |
| Suprabasal layer | *Oser1* | 0.46 | 1.22E-07 |
| Suprabasal layer | *Dusp5* | 0.36 | 1.65E-07 |
| Suprabasal layer | *Zranb1* | 0.40 | 2.22E-07 |
| Suprabasal layer | *Ggh* | 0.67 | 2.60E-07 |
| Suprabasal layer | *Wnt7b* | 0.46 | 2.99E-07 |
| Suprabasal layer | *Qsox1* | 0.48 | 3.61E-07 |
| Suprabasal layer | *Vsig10l* | 0.46 | 3.91E-07 |
| Suprabasal layer | *Crybg1* | 0.48 | 4.12E-07 |
| Suprabasal layer | *Teddm3* | 0.50 | 4.18E-07 |
| Suprabasal layer | *Cyfip2* | 0.27 | 4.24E-07 |
| Suprabasal layer | *Tex264* | 0.42 | 4.44E-07 |
| Suprabasal layer | *Kyat1* | 0.57 | 5.51E-07 |
| Suprabasal layer | *Mreg* | 0.27 | 6.22E-07 |
| Suprabasal layer | *Fam83d* | 0.32 | 7.33E-07 |
| Suprabasal layer | *Thop1* | 0.49 | 8.44E-07 |
| Suprabasal layer | *Spint1* | 0.46 | 8.68E-07 |
| Suprabasal layer | *Gm34455* | 0.30 | 1.01E-06 |
| Suprabasal layer | *Mxd1* | 0.47 | 1.14E-06 |
| Suprabasal layer | *Rec8* | 0.27 | 1.24E-06 |
| Suprabasal layer | *Hip1r* | 0.44 | 1.52E-06 |
| Suprabasal layer | *Tuft1* | 0.42 | 1.62E-06 |
| Suprabasal layer | *Atp6v1c2* | 0.35 | 1.82E-06 |
| Suprabasal layer | *Cldn4* | 0.32 | 2.47E-06 |
| Suprabasal layer | *Tpp2* | 0.48 | 2.51E-06 |
| Suprabasal layer | *Slc27a4* | 0.50 | 2.75E-06 |
| Suprabasal layer | *Plk3* | 0.36 | 2.98E-06 |
| Suprabasal layer | *Nucb2* | 0.38 | 3.24E-06 |
| Suprabasal layer | *Gba* | 0.46 | 3.34E-06 |
| Suprabasal layer | *AI661453* | 0.48 | 3.52E-06 |
| Suprabasal layer | *Slurp1* | 0.34 | 3.85E-06 |
| Suprabasal layer | *Tprg* | 0.33 | 3.91E-06 |
| Suprabasal layer | *Sgpp2* | 0.49 | 5.59E-06 |
| Suprabasal layer | *Ube2r2* | 0.49 | 5.66E-06 |
| Suprabasal layer | *Aldh1a7* | 0.37 | 5.84E-06 |
| Suprabasal layer | *Epn3* | 0.46 | 5.88E-06 |
| Suprabasal layer | *Plxdc2* | 0.45 | 6.68E-06 |
| Suprabasal layer | *Camsap3* | 0.38 | 8.05E-06 |
| Suprabasal layer | *Osbpl1a* | 0.30 | 9.57E-06 |
| Suprabasal layer | *Fut2* | 0.27 | 1.31E-05 |
| Suprabasal layer | *Ypel3* | 0.46 | 1.52E-05 |
| Suprabasal layer | *Dsg1b* | 0.39 | 1.63E-05 |
| Suprabasal layer | *Calcoco1* | 0.26 | 1.66E-05 |
| Suprabasal layer | *Creg1* | 0.43 | 1.85E-05 |
| Suprabasal layer | *Hexb* | 0.40 | 1.94E-05 |
| Suprabasal layer | *Gstm2* | 0.53 | 2.11E-05 |
| Suprabasal layer | *Tent5b* | 0.39 | 2.37E-05 |
| Suprabasal layer | *Slc10a6* | 0.41 | 2.40E-05 |
| Suprabasal layer | *Ctnnbip1* | 0.52 | 2.58E-05 |
| Suprabasal layer | *4833423E24Rik* | 0.45 | 2.78E-05 |
| Suprabasal layer | *Tmem54* | 0.49 | 2.94E-05 |
| Suprabasal layer | *Sdr42e1* | 0.43 | 3.12E-05 |
| Suprabasal layer | *Metrnl* | 0.41 | 3.13E-05 |
| Suprabasal layer | *Pof1b* | 0.41 | 3.36E-05 |
| Suprabasal layer | *Hebp2* | 0.50 | 3.66E-05 |
| Suprabasal layer | *Peli1* | 0.34 | 3.80E-05 |
| Suprabasal layer | *Wfdc5* | 0.45 | 3.90E-05 |
| Suprabasal layer | *Fdps* | 0.49 | 3.92E-05 |
| Suprabasal layer | *Adgrl2* | 0.30 | 5.48E-05 |
| Suprabasal layer | *Hsd17b2* | 0.38 | 5.50E-05 |
| Suprabasal layer | *Kctd11* | 0.40 | 6.13E-05 |
| Suprabasal layer | *Tsc22d2* | 0.50 | 6.30E-05 |
| Suprabasal layer | *Zfp706* | 0.34 | 6.33E-05 |
| Suprabasal layer | *Lrrc28* | 0.33 | 6.82E-05 |
| Suprabasal layer | *Rhof* | 0.31 | 7.34E-05 |
| Suprabasal layer | *Kifc3* | 0.28 | 8.24E-05 |
| Suprabasal layer | *Churc1* | 0.49 | 8.52E-05 |
| Suprabasal layer | *Foxo3* | 0.43 | 8.76E-05 |
| Suprabasal layer | *Klk10* | 0.43 | 9.42E-05 |
| Suprabasal layer | *Gdpd3* | 0.54 | 9.46E-05 |
| Suprabasal layer | *Srgap2* | 0.35 | 9.63E-05 |
| Suprabasal layer | *Rab5a* | 0.39 | 1.04E-04 |
| Suprabasal layer | *Sptlc3* | 0.26 | 1.05E-04 |
| Suprabasal layer | *Cnot6l* | 0.47 | 1.09E-04 |
| Suprabasal layer | *Polb* | 0.36 | 1.14E-04 |
| Suprabasal layer | *Slc28a3* | 0.32 | 1.17E-04 |
| Suprabasal layer | *Zdhhc9* | 0.42 | 1.18E-04 |
| Suprabasal layer | *Pfkfb4* | 0.28 | 1.25E-04 |
| Suprabasal layer | *Map2k3* | 0.37 | 1.26E-04 |
| Suprabasal layer | *Cln8* | 0.38 | 1.29E-04 |
| Suprabasal layer | *Lipm* | 0.40 | 1.30E-04 |
| Suprabasal layer | *Capn15* | 0.43 | 1.41E-04 |
| Suprabasal layer | *Daam1* | 0.48 | 1.60E-04 |
| Suprabasal layer | *Elovl7* | 0.53 | 1.60E-04 |
| Suprabasal layer | *Gdpd1* | 0.32 | 1.83E-04 |
| Suprabasal layer | *Ahnak* | 0.32 | 1.90E-04 |
| Suprabasal layer | *Sytl1* | 0.43 | 1.99E-04 |
| Suprabasal layer | *Ripk2* | 0.32 | 2.25E-04 |
| Suprabasal layer | *Paqr5* | 0.28 | 2.37E-04 |
| Suprabasal layer | *Lmtk2* | 0.28 | 2.47E-04 |
| Suprabasal layer | *Dexi* | 0.35 | 2.57E-04 |
| Suprabasal layer | *Fam135a* | 0.39 | 2.63E-04 |
| Suprabasal layer | *Sh3bp5l* | 0.41 | 2.72E-04 |
| Suprabasal layer | *Chl1* | 0.43 | 3.49E-04 |
| Suprabasal layer | *Mfap3l* | 0.47 | 3.71E-04 |
| Suprabasal layer | *Lipk* | 0.26 | 4.38E-04 |
| Suprabasal layer | *Snx9* | 0.38 | 4.72E-04 |
| Suprabasal layer | *Trim25* | 0.35 | 5.80E-04 |
| Suprabasal layer | *Phactr4* | 0.30 | 6.03E-04 |
| Suprabasal layer | *Gjb6* | 0.46 | 6.58E-04 |
| Suprabasal layer | *Itpkc* | 0.33 | 8.80E-04 |
| Suprabasal layer | *Castor2* | 0.27 | 1.15E-03 |
| Suprabasal layer | *Ace2* | 0.30 | 1.18E-03 |
| Suprabasal layer | *Arhgef10l* | 0.43 | 1.26E-03 |
| Suprabasal layer | *Tmem45a* | 0.41 | 1.39E-03 |
| Suprabasal layer | *Pla2g4f* | 0.43 | 1.40E-03 |
| Suprabasal layer | *Ano9* | 0.30 | 1.46E-03 |
| Suprabasal layer | *Spink12* | 0.37 | 1.71E-03 |
| Suprabasal layer | *Dapl1* | 0.41 | 1.75E-03 |
| Suprabasal layer | *Ndufa4l2* | 0.68 | 2.00E-03 |
| Suprabasal layer | *Ephx3* | 0.33 | 2.05E-03 |
| Suprabasal layer | *Rab27b* | 0.34 | 2.10E-03 |
| Suprabasal layer | *Myo6* | 0.38 | 2.29E-03 |
| Suprabasal layer | *Mpzl3* | 0.37 | 2.34E-03 |
| Suprabasal layer | *Syt8* | 0.30 | 2.45E-03 |
| Suprabasal layer | *Ube2d3* | 0.29 | 2.46E-03 |
| Suprabasal layer | *Il1f6* | 0.45 | 2.66E-03 |
| Suprabasal layer | *Ly6g6e* | 0.34 | 2.79E-03 |
| Suprabasal layer | *Fhdc1* | 0.38 | 2.99E-03 |
| Suprabasal layer | *Dedd2* | 0.36 | 3.26E-03 |
| Suprabasal layer | *Vasn* | 0.43 | 3.29E-03 |
| Suprabasal layer | *Ptgr1* | 0.36 | 3.44E-03 |
| Suprabasal layer | *Egln3* | 0.57 | 3.61E-03 |
| Suprabasal layer | *Ppfia3* | 0.34 | 3.82E-03 |
| Suprabasal layer | *Fbp2* | 0.28 | 3.90E-03 |
| Suprabasal layer | *Plekhn1* | 0.36 | 4.02E-03 |
| Suprabasal layer | *Rora* | 0.39 | 4.91E-03 |
| Suprabasal layer | *Cnbp* | 0.33 | 5.65E-03 |
| Suprabasal layer | *Slurp2* | 0.37 | 5.67E-03 |
| Suprabasal layer | *Ces1h* | 0.32 | 6.57E-03 |
| Suprabasal layer | *Serpinb3c* | 0.64 | 6.66E-03 |
| Suprabasal layer | *Malat1* | 1.03 | 7.16E-03 |
| Suprabasal layer | *Vps13d* | 0.27 | 7.30E-03 |
| Suprabasal layer | *Natd1* | 0.31 | 7.40E-03 |
| Suprabasal layer | *Tmprss11a* | 0.41 | 8.21E-03 |
| Suprabasal layer | *Pnpla1* | 0.26 | 8.23E-03 |
| Suprabasal layer | *Acot1* | 0.27 | 8.27E-03 |
| Suprabasal layer | *Iqsec2* | 0.31 | 8.44E-03 |
| Suprabasal layer | *Them5* | 0.32 | 8.80E-03 |
| Suprabasal layer | *Krt23* | 0.31 | 9.11E-03 |
| Suprabasal layer | *Scpep1* | 0.29 | 9.98E-03 |
| Suprabasal layer | *Numb* | 0.25 | 1.15E-02 |
| Suprabasal layer | *Cyp4f39* | 0.31 | 1.19E-02 |
| Suprabasal layer | *Sptlc2* | 0.39 | 1.29E-02 |
| Suprabasal layer | *Crybg2* | 0.27 | 1.29E-02 |
| Suprabasal layer | *Usp53* | 0.26 | 1.30E-02 |
| Suprabasal layer | *Trim62* | 0.30 | 1.38E-02 |
| Suprabasal layer | *1810037I17Rik* | 0.33 | 1.42E-02 |
| Suprabasal layer | *Pdlim2* | 0.35 | 1.42E-02 |
| Suprabasal layer | *Blzf1* | 0.27 | 1.60E-02 |
| Suprabasal layer | *Crabp2* | 0.40 | 1.71E-02 |
| Suprabasal layer | *Arhgap27* | 0.29 | 1.80E-02 |
| Suprabasal layer | *Uaca* | 0.33 | 1.82E-02 |
| Suprabasal layer | *Osbpl2* | 0.27 | 1.85E-02 |
| Suprabasal layer | *Scnn1a* | 0.33 | 1.97E-02 |
| Suprabasal layer | *Sdr16c5* | 0.35 | 2.11E-02 |
| Suprabasal layer | *Tmem40* | 0.34 | 2.18E-02 |
| Suprabasal layer | *Fntb* | 0.25 | 2.33E-02 |
| Suprabasal layer | *Sdcbp2* | 0.29 | 2.40E-02 |
| Suprabasal layer | *Dhcr24* | 0.33 | 2.52E-02 |
| Suprabasal layer | *Snrnp25* | 0.27 | 2.61E-02 |
| Suprabasal layer | *Rpia* | 0.25 | 2.73E-02 |
| Suprabasal layer | *Slc9a3r1* | 0.29 | 3.45E-02 |
| Suprabasal layer | *Tspan5* | 0.30 | 3.67E-02 |
| Suprabasal layer | *Phkg2* | 0.31 | 4.05E-02 |
| Suprabasal layer | *Atp6v0a2* | 0.30 | 4.19E-02 |
| Suprabasal layer | *Pld2* | 0.31 | 4.39E-02 |
| Suprabasal layer | *Cds1* | 0.32 | 4.57E-02 |
| Suprabasal layer | *Pik3c2a* | 0.27 | 4.73E-02 |
| Basal layer | *Mt2* | 0.73 | 2.86E-37 |
| Basal layer | *Eef1a1* | 0.69 | 6.57E-37 |
| Basal layer | *Gstp1* | 0.83 | 4.07E-33 |
| Basal layer | *Mt4* | 0.72 | 2.96E-32 |
| Basal layer | *Ftl1* | 0.96 | 1.04E-29 |
| Basal layer | *Adh7* | 0.95 | 1.66E-26 |
| Basal layer | *Ptma* | 0.58 | 2.83E-26 |
| Basal layer | *Uba52* | 0.61 | 2.74E-25 |
| Basal layer | *Tpt1* | 0.47 | 7.84E-25 |
| Basal layer | *Gstm1* | 0.98 | 2.39E-23 |
| Basal layer | *Krt15* | 1.12 | 3.22E-23 |
| Basal layer | *Ppia* | 0.59 | 1.77E-20 |
| Basal layer | *Eef1g* | 0.52 | 3.07E-20 |
| Basal layer | *Ckap2l* | 0.45 | 1.07E-19 |
| Basal layer | *Gm11808* | 0.55 | 3.54E-19 |
| Basal layer | *Hoxc5* | 0.32 | 9.95E-19 |
| Basal layer | *Igfbp2* | 0.83 | 3.16E-18 |
| Basal layer | *Slc1a4* | 0.60 | 5.06E-18 |
| Basal layer | *Gas5* | 0.57 | 1.32E-17 |
| Basal layer | *Wfdc2* | 0.63 | 1.40E-17 |
| Basal layer | *Wnt10a* | 0.47 | 2.25E-17 |
| Basal layer | *Ces1h* | 0.85 | 3.90E-17 |
| Basal layer | *Gm47283* | 0.59 | 8.91E-17 |
| Basal layer | *Slc25a5* | 0.60 | 9.31E-17 |
| Basal layer | *Fxyd3* | 0.62 | 1.10E-16 |
| Basal layer | *Krt5* | 0.51 | 2.25E-16 |
| Basal layer | *Lmna* | 0.47 | 3.16E-16 |
| Basal layer | *Tuba1b* | 0.51 | 5.76E-16 |
| Basal layer | *Ggta1* | 0.35 | 7.36E-16 |
| Basal layer | *Atpif1* | 0.56 | 1.57E-15 |
| Basal layer | *Il1r2* | 0.88 | 1.64E-15 |
| Basal layer | *Atp1b3* | 0.52 | 4.34E-15 |
| Basal layer | *Mgst2* | 0.53 | 1.20E-14 |
| Basal layer | *Ces1b* | 0.60 | 4.28E-14 |
| Basal layer | *Tmem132a* | 0.53 | 4.33E-14 |
| Basal layer | *Stmn1* | 0.54 | 5.37E-14 |
| Basal layer | *St6galnac2* | 0.36 | 5.54E-14 |
| Basal layer | *Fgfr2* | 0.48 | 4.36E-13 |
| Basal layer | *Nqo1* | 0.51 | 6.28E-13 |
| Basal layer | *Ccnd2* | 0.41 | 8.92E-13 |
| Basal layer | *Plk1* | 0.26 | 1.50E-12 |
| Basal layer | *Itgb4* | 0.45 | 1.55E-12 |
| Basal layer | *Eif4a1* | 0.43 | 1.57E-12 |
| Basal layer | *Rack1* | 0.32 | 2.00E-12 |
| Basal layer | *Antxr1* | 0.34 | 2.30E-12 |
| Basal layer | *Atp1a1* | 0.51 | 2.68E-12 |
| Basal layer | *H2afz* | 0.50 | 2.82E-12 |
| Basal layer | *Hspa8* | 0.49 | 3.48E-12 |
| Basal layer | *Sfrp2* | 0.29 | 3.91E-12 |
| Basal layer | *Eef1b2* | 0.39 | 4.17E-12 |
| Basal layer | *Hmgb2* | 0.52 | 4.60E-12 |
| Basal layer | *Npm1* | 0.46 | 4.68E-12 |
| Basal layer | *Gclm* | 0.53 | 5.11E-12 |
| Basal layer | *Gtf2i* | 0.39 | 5.13E-12 |
| Basal layer | *Tgif1* | 0.29 | 6.43E-12 |
| Basal layer | *Gpsm2* | 0.31 | 9.04E-12 |
| Basal layer | *Cyp2f2* | 0.84 | 1.19E-11 |
| Basal layer | *Mt3* | 0.55 | 1.28E-11 |
| Basal layer | *Nme2* | 0.39 | 1.34E-11 |
| Basal layer | *Paics* | 0.35 | 1.62E-11 |
| Basal layer | *Nol9* | 0.33 | 1.70E-11 |
| Basal layer | *Tpx2* | 0.29 | 1.71E-11 |
| Basal layer | *Sod1* | 0.49 | 1.84E-11 |
| Basal layer | *Aldh3a1* | 0.67 | 2.11E-11 |
| Basal layer | *Hmgn2* | 0.28 | 3.31E-11 |
| Basal layer | *Gstm2* | 0.56 | 4.79E-11 |
| Basal layer | *Sox2* | 0.46 | 4.93E-11 |
| Basal layer | *Mplkip* | 0.27 | 5.55E-11 |
| Basal layer | *H2afv* | 0.42 | 6.23E-11 |
| Basal layer | *Nudt21* | 0.36 | 6.48E-11 |
| Basal layer | *Snrpg* | 0.47 | 7.22E-11 |
| Basal layer | *Hnrnpa3* | 0.47 | 7.43E-11 |
| Basal layer | *Nsa2* | 0.39 | 9.41E-11 |
| Basal layer | *Pebp1* | 0.44 | 1.02E-10 |
| Basal layer | *Notch1* | 0.44 | 1.19E-10 |
| Basal layer | *Naca* | 0.36 | 1.19E-10 |
| Basal layer | *Ddb1* | 0.43 | 1.25E-10 |
| Basal layer | *Pcbp2* | 0.45 | 1.39E-10 |
| Basal layer | *Rnaset2b* | 0.25 | 1.42E-10 |
| Basal layer | *Cbr1* | 0.44 | 1.47E-10 |
| Basal layer | *Fau* | 0.31 | 1.75E-10 |
| Basal layer | *Tkt* | 0.45 | 1.97E-10 |
| Basal layer | *Ran* | 0.43 | 2.08E-10 |
| Basal layer | *Eef2* | 0.31 | 2.42E-10 |
| Basal layer | *Incenp* | 0.31 | 2.53E-10 |
| Basal layer | *Snrpf* | 0.48 | 2.76E-10 |
| Basal layer | *Rxra* | 0.44 | 3.25E-10 |
| Basal layer | *Col17a1* | 0.41 | 3.41E-10 |
| Basal layer | *Trp53* | 0.35 | 3.51E-10 |
| Basal layer | *Tmsb4x* | 0.37 | 4.04E-10 |
| Basal layer | *Cdca7* | 0.27 | 4.57E-10 |
| Basal layer | *Hsp90aa1* | 0.48 | 5.50E-10 |
| Basal layer | *Vsnl1* | 0.35 | 5.55E-10 |
| Basal layer | *Aldh2* | 0.45 | 8.26E-10 |
| Basal layer | *Psat1* | 0.40 | 8.31E-10 |
| Basal layer | *Il20rb* | 0.30 | 8.41E-10 |
| Basal layer | *Wdr48* | 0.39 | 8.44E-10 |
| Basal layer | *S100a10* | 0.39 | 1.08E-09 |
| Basal layer | *Ugdh* | 0.39 | 1.36E-09 |
| Basal layer | *Plp2* | 0.27 | 1.40E-09 |
| Basal layer | *Jag2* | 0.38 | 1.50E-09 |
| Basal layer | *Mau2* | 0.26 | 1.78E-09 |
| Basal layer | *Dlk2* | 0.28 | 1.87E-09 |
| Basal layer | *Sox15* | 0.26 | 1.98E-09 |
| Basal layer | *Impdh2* | 0.38 | 2.63E-09 |
| Basal layer | *Hmgb1* | 0.41 | 3.32E-09 |
| Basal layer | *Ube2c* | 0.46 | 3.43E-09 |
| Basal layer | *Tomm7* | 0.41 | 3.84E-09 |
| Basal layer | *Nudt14* | 0.28 | 4.28E-09 |
| Basal layer | *Gpnmb* | 0.54 | 4.62E-09 |
| Basal layer | *Gsta4* | 0.37 | 4.72E-09 |
| Basal layer | *Prdx6* | 0.45 | 5.00E-09 |
| Basal layer | *Aldh3b2* | 0.47 | 5.37E-09 |
| Basal layer | *Zfp36l2* | 0.43 | 6.51E-09 |
| Basal layer | *Trp63* | 0.45 | 7.44E-09 |
| Basal layer | *Thumpd3* | 0.37 | 7.96E-09 |
| Basal layer | *Ints3* | 0.28 | 8.39E-09 |
| Basal layer | *Zfp579* | 0.35 | 8.85E-09 |
| Basal layer | *Cks2* | 0.37 | 8.96E-09 |
| Basal layer | *Moxd1* | 0.26 | 9.89E-09 |
| Basal layer | *Cbr3* | 0.52 | 1.02E-08 |
| Basal layer | *Dut* | 0.28 | 1.08E-08 |
| Basal layer | *Ccdc3* | 0.27 | 1.41E-08 |
| Basal layer | *Mapkap1* | 0.27 | 1.42E-08 |
| Basal layer | *Trmt112* | 0.35 | 1.51E-08 |
| Basal layer | *Snrpb2* | 0.27 | 1.53E-08 |
| Basal layer | *Ociad2* | 0.39 | 1.83E-08 |
| Basal layer | *Ccnd1* | 0.42 | 1.85E-08 |
| Basal layer | *Hint1* | 0.40 | 1.87E-08 |
| Basal layer | *Mt1* | 0.33 | 1.89E-08 |
| Basal layer | *Snrpd1* | 0.36 | 1.93E-08 |
| Basal layer | *Ccna2* | 0.28 | 1.94E-08 |
| Basal layer | *Maz* | 0.29 | 2.05E-08 |
| Basal layer | *Zbtb7c* | 0.31 | 2.49E-08 |
| Basal layer | *Car12* | 0.48 | 2.67E-08 |
| Basal layer | *Ajuba* | 0.38 | 2.84E-08 |
| Basal layer | *Dgka* | 0.32 | 3.56E-08 |
| Basal layer | *Mak16* | 0.27 | 3.71E-08 |
| Basal layer | *Mrpl52* | 0.46 | 5.38E-08 |
| Basal layer | *Acp5* | 0.38 | 5.41E-08 |
| Basal layer | *Naa38* | 0.32 | 1.01E-07 |
| Basal layer | *Prpf19* | 0.49 | 1.39E-07 |
| Basal layer | *Eno1* | 0.33 | 1.43E-07 |
| Basal layer | *Itm2b* | 0.32 | 1.48E-07 |
| Basal layer | *Ptprf* | 0.37 | 1.75E-07 |
| Basal layer | *Commd4* | 0.32 | 1.80E-07 |
| Basal layer | *Cat* | 0.52 | 1.97E-07 |
| Basal layer | *Eif3k* | 0.37 | 2.14E-07 |
| Basal layer | *Sdhd* | 0.28 | 2.36E-07 |
| Basal layer | *Snrpe* | 0.42 | 2.45E-07 |
| Basal layer | *Smc4* | 0.27 | 2.46E-07 |
| Basal layer | *Imp3* | 0.28 | 2.60E-07 |
| Basal layer | *Tomm5* | 0.38 | 3.20E-07 |
| Basal layer | *Plxnb1* | 0.37 | 3.47E-07 |
| Basal layer | *Plch2* | 0.37 | 3.73E-07 |
| Basal layer | *Oxct1* | 0.38 | 3.76E-07 |
| Basal layer | *Ahcyl2* | 0.25 | 5.60E-07 |
| Basal layer | *Ptbp1* | 0.36 | 5.63E-07 |
| Basal layer | *Cd109* | 0.32 | 6.41E-07 |
| Basal layer | *Ptgr1* | 0.45 | 6.84E-07 |
| Basal layer | *Hspe1* | 0.39 | 7.22E-07 |
| Basal layer | *Sptssb* | 0.46 | 7.63E-07 |
| Basal layer | *Smoc2* | 0.27 | 7.67E-07 |
| Basal layer | *Hspd1* | 0.40 | 7.85E-07 |
| Basal layer | *Hnrnpab* | 0.31 | 8.26E-07 |
| Basal layer | *Ewsr1* | 0.38 | 9.38E-07 |
| Basal layer | *Eif5a* | 0.27 | 9.74E-07 |
| Basal layer | *H3f3a* | 0.29 | 1.04E-06 |
| Basal layer | *Blvrb* | 0.41 | 1.09E-06 |
| Basal layer | *Plek2* | 0.26 | 1.11E-06 |
| Basal layer | *Entpd2* | 0.34 | 1.17E-06 |
| Basal layer | *Hsph1* | 0.36 | 1.35E-06 |
| Basal layer | *Dst* | 0.42 | 1.44E-06 |
| Basal layer | *Gpt* | 0.26 | 1.45E-06 |
| Basal layer | *Dgcr6* | 0.29 | 1.53E-06 |
| Basal layer | *Esd* | 0.46 | 1.76E-06 |
| Basal layer | *Bax* | 0.30 | 1.96E-06 |
| Basal layer | *Cisd3* | 0.27 | 2.00E-06 |
| Basal layer | *Selenbp1* | 0.60 | 2.34E-06 |
| Basal layer | *Set* | 0.36 | 2.43E-06 |
| Basal layer | *Itga3* | 0.40 | 2.52E-06 |
| Basal layer | *Ube2s* | 0.36 | 2.64E-06 |
| Basal layer | *Psma4* | 0.27 | 2.84E-06 |
| Basal layer | *Ola1* | 0.32 | 2.90E-06 |
| Basal layer | *Nono* | 0.31 | 2.99E-06 |
| Basal layer | *Sgpp1* | 0.28 | 3.19E-06 |
| Basal layer | *Dnmt1* | 0.28 | 3.77E-06 |
| Basal layer | *Pmm1* | 0.28 | 3.82E-06 |
| Basal layer | *Lmnb1* | 0.26 | 3.98E-06 |
| Basal layer | *Plxna1* | 0.31 | 4.36E-06 |
| Basal layer | *Eif4b* | 0.33 | 4.59E-06 |
| Basal layer | *Eva1c* | 0.34 | 4.65E-06 |
| Basal layer | *Ncl* | 0.32 | 4.93E-06 |
| Basal layer | *Serpinb5* | 0.35 | 5.79E-06 |
| Basal layer | *Ckap2* | 0.33 | 6.16E-06 |
| Basal layer | *Akr1b8* | 0.45 | 6.21E-06 |
| Basal layer | *Ifitm3* | 0.36 | 6.25E-06 |
| Basal layer | *Osgin1* | 0.29 | 6.26E-06 |
| Basal layer | *Mcm7* | 0.26 | 6.29E-06 |
| Basal layer | *Apoc1* | 0.48 | 8.14E-06 |
| Basal layer | *Sfpq* | 0.31 | 8.37E-06 |
| Basal layer | *G3bp1* | 0.34 | 8.73E-06 |
| Basal layer | *Cdk2ap2* | 0.30 | 1.00E-05 |
| Basal layer | *2410006H16Rik* | 0.37 | 1.01E-05 |
| Basal layer | *Gsta2* | 0.30 | 1.09E-05 |
| Basal layer | *Gclc* | 0.46 | 1.11E-05 |
| Basal layer | *Cdc20* | 0.32 | 1.17E-05 |
| Basal layer | *Pdcd4* | 0.32 | 1.42E-05 |
| Basal layer | *Cpt2* | 0.28 | 1.43E-05 |
| Basal layer | *Wnk4* | 0.27 | 1.47E-05 |
| Basal layer | *Psma5* | 0.31 | 1.50E-05 |
| Basal layer | *Gcdh* | 0.32 | 1.53E-05 |
| Basal layer | *Hpgd* | 0.56 | 1.62E-05 |
| Basal layer | *Hspa1b* | 0.42 | 1.67E-05 |
| Basal layer | *Efs* | 0.27 | 1.74E-05 |
| Basal layer | *Cavin1* | 0.27 | 1.85E-05 |
| Basal layer | *Tubb4b* | 0.29 | 2.13E-05 |
| Basal layer | *Hsd17b2* | 0.29 | 2.30E-05 |
| Basal layer | *Birc5* | 0.25 | 2.42E-05 |
| Basal layer | *Entpd5* | 0.38 | 2.47E-05 |
| Basal layer | *Top2a* | 0.29 | 2.49E-05 |
| Basal layer | *Tspan9* | 0.28 | 2.86E-05 |
| Basal layer | *Aprt* | 0.29 | 3.07E-05 |
| Basal layer | *Acadvl* | 0.31 | 3.20E-05 |
| Basal layer | *Anxa5* | 0.31 | 3.29E-05 |
| Basal layer | *Mir205hg* | 0.44 | 3.35E-05 |
| Basal layer | *Serpinh1* | 0.27 | 3.71E-05 |
| Basal layer | *Usp10* | 0.34 | 3.72E-05 |
| Basal layer | *Rnpep* | 0.29 | 3.78E-05 |
| Basal layer | *Ephx1* | 0.28 | 3.82E-05 |
| Basal layer | *Prxl2a* | 0.27 | 3.99E-05 |
| Basal layer | *Nap1l1* | 0.32 | 4.18E-05 |
| Basal layer | *Tpp1* | 0.27 | 4.31E-05 |
| Basal layer | *Nop53* | 0.31 | 4.33E-05 |
| Basal layer | *Eps15l1* | 0.39 | 4.77E-05 |
| Basal layer | *Kctd15* | 0.27 | 4.93E-05 |
| Basal layer | *Ckmt1* | 0.34 | 4.97E-05 |
| Basal layer | *Agrn* | 0.28 | 5.00E-05 |
| Basal layer | *Edf1* | 0.33 | 5.54E-05 |
| Basal layer | *Ucp2* | 0.32 | 5.57E-05 |
| Basal layer | *Tbca* | 0.34 | 5.79E-05 |
| Basal layer | *Snhg15* | 0.32 | 5.90E-05 |
| Basal layer | *Ssrp1* | 0.28 | 5.97E-05 |
| Basal layer | *Tma7* | 0.34 | 6.05E-05 |
| Basal layer | *Nuf2* | 0.34 | 6.15E-05 |
| Basal layer | *Slc38a2* | 0.36 | 6.42E-05 |
| Basal layer | *Srsf7* | 0.30 | 6.48E-05 |
| Basal layer | *Krt32* | 0.25 | 6.61E-05 |
| Basal layer | *Phb2* | 0.32 | 6.90E-05 |
| Basal layer | *Anp32b* | 0.32 | 7.23E-05 |
| Basal layer | *Vars* | 0.31 | 7.23E-05 |
| Basal layer | *Pcna* | 0.30 | 7.47E-05 |
| Basal layer | *Shisa5* | 0.30 | 7.88E-05 |
| Basal layer | *Cct4* | 0.32 | 7.96E-05 |
| Basal layer | *Pnp* | 0.40 | 8.24E-05 |
| Basal layer | *Abcd3* | 0.27 | 8.26E-05 |
| Basal layer | *Cntnap1* | 0.31 | 8.67E-05 |
| Basal layer | *Tufm* | 0.28 | 9.43E-05 |
| Basal layer | *Oat* | 0.33 | 9.49E-05 |
| Basal layer | *Hnrnpa1* | 0.30 | 9.73E-05 |
| Basal layer | *Crip2* | 0.27 | 1.00E-04 |
| Basal layer | *Cfl1* | 0.31 | 1.04E-04 |
| Basal layer | *Srsf1* | 0.29 | 1.09E-04 |
| Basal layer | *Mgst3* | 0.28 | 1.11E-04 |
| Basal layer | *Fkbp4* | 0.31 | 1.21E-04 |
| Basal layer | *Prdx1* | 0.28 | 1.24E-04 |
| Basal layer | *Efnb1* | 0.30 | 1.24E-04 |
| Basal layer | *Tubb5* | 0.30 | 1.25E-04 |
| Basal layer | *Pcbp1* | 0.36 | 1.28E-04 |
| Basal layer | *Mif* | 0.29 | 1.32E-04 |
| Basal layer | *Eif3f* | 0.33 | 1.37E-04 |
| Basal layer | *Foxq1* | 0.25 | 1.41E-04 |
| Basal layer | *Procr* | 0.25 | 1.44E-04 |
| Basal layer | *Morf4l1* | 0.27 | 1.49E-04 |
| Basal layer | *Tacc3* | 0.35 | 1.51E-04 |
| Basal layer | *Ppib* | 0.27 | 1.72E-04 |
| Basal layer | *Naa10* | 0.25 | 1.75E-04 |
| Basal layer | *Ifi27* | 0.31 | 1.90E-04 |
| Basal layer | *Fbl* | 0.39 | 1.91E-04 |
| Basal layer | *Cdk1* | 0.38 | 1.92E-04 |
| Basal layer | *Aup1* | 0.26 | 2.04E-04 |
| Basal layer | *Rin2* | 0.28 | 2.15E-04 |
| Basal layer | *Hnrnpa0* | 0.31 | 2.21E-04 |
| Basal layer | *Fbln1* | 0.25 | 2.22E-04 |
| Basal layer | *Slc48a1* | 0.29 | 2.23E-04 |
| Basal layer | *Lima1* | 0.28 | 2.43E-04 |
| Basal layer | *Cyb5r3* | 0.30 | 2.44E-04 |
| Basal layer | *Gsta1* | 0.37 | 2.44E-04 |
| Basal layer | *Ndufa7* | 0.25 | 2.61E-04 |
| Basal layer | *Fam83h* | 0.26 | 2.81E-04 |
| Basal layer | *Ddx5* | 0.27 | 2.95E-04 |
| Basal layer | *Tfdp1* | 0.32 | 2.96E-04 |
| Basal layer | *Dapl1* | 0.29 | 2.97E-04 |
| Basal layer | *Mmp2* | 0.29 | 3.05E-04 |
| Basal layer | *Pgrmc1* | 0.36 | 3.17E-04 |
| Basal layer | *Ptgfrn* | 0.28 | 3.79E-04 |
| Basal layer | *Fgfr3* | 0.38 | 3.89E-04 |
| Basal layer | *Ap2m1* | 0.29 | 3.90E-04 |
| Basal layer | *Phgdh* | 0.34 | 3.93E-04 |
| Basal layer | *Ptprs* | 0.28 | 3.94E-04 |
| Basal layer | *Necap2* | 0.33 | 4.13E-04 |
| Basal layer | *Slc39a6* | 0.30 | 4.42E-04 |
| Basal layer | *Mybbp1a* | 0.26 | 4.47E-04 |
| Basal layer | *Fkbp2* | 0.26 | 4.51E-04 |
| Basal layer | *Psmd14* | 0.27 | 5.13E-04 |
| Basal layer | *Mettl17* | 0.30 | 5.81E-04 |
| Basal layer | *Prelid1* | 0.27 | 6.06E-04 |
| Basal layer | *Npm3* | 0.28 | 6.94E-04 |
| Basal layer | *Tra2b* | 0.28 | 7.12E-04 |
| Basal layer | *Reep5* | 0.30 | 7.51E-04 |
| Basal layer | *Tmem258* | 0.26 | 8.41E-04 |
| Basal layer | *Hnrnpu* | 0.26 | 9.06E-04 |
| Basal layer | *Pex6* | 0.34 | 9.44E-04 |
| Basal layer | *Uqcrc1* | 0.32 | 1.00E-03 |
| Basal layer | *Dap3* | 0.33 | 1.06E-03 |
| Basal layer | *Mrfap1* | 0.30 | 1.14E-03 |
| Basal layer | *Tns4* | 0.27 | 1.18E-03 |
| Basal layer | *Fzd6* | 0.26 | 1.20E-03 |
| Basal layer | *Hras* | 0.27 | 1.21E-03 |
| Basal layer | *Slc25a17* | 0.28 | 1.23E-03 |
| Basal layer | *Clta* | 0.29 | 1.27E-03 |
| Basal layer | *Ssna1* | 0.29 | 1.46E-03 |
| Basal layer | *Trappc5* | 0.25 | 1.52E-03 |
| Basal layer | *Barx2* | 0.30 | 1.66E-03 |
| Basal layer | *Arfgap3* | 0.31 | 1.78E-03 |
| Basal layer | *Dennd1a* | 0.30 | 1.80E-03 |
| Basal layer | *Mgst1* | 0.33 | 1.86E-03 |
| Basal layer | *Hnrnpa2b1* | 0.29 | 1.87E-03 |
| Basal layer | *Sec61b* | 0.31 | 1.97E-03 |
| Basal layer | *Fam83g* | 0.32 | 2.19E-03 |
| Basal layer | *Ssr2* | 0.35 | 2.22E-03 |
| Basal layer | *G6pdx* | 0.29 | 2.57E-03 |
| Basal layer | *Cox8a* | 0.25 | 2.59E-03 |
| Basal layer | *Hnrnpc* | 0.27 | 3.01E-03 |
| Basal layer | *Naa50* | 0.27 | 3.08E-03 |
| Basal layer | *Tmpo* | 0.30 | 3.53E-03 |
| Basal layer | *Txn1* | 0.29 | 3.59E-03 |
| Basal layer | *Ost4* | 0.29 | 3.61E-03 |
| Basal layer | *Dnaja1* | 0.26 | 3.88E-03 |
| Basal layer | *Dsc3* | 0.26 | 3.88E-03 |
| Basal layer | *Atp5g3* | 0.26 | 3.94E-03 |
| Basal layer | *Ring1* | 0.28 | 4.03E-03 |
| Basal layer | *Zfp524* | 0.35 | 4.17E-03 |
| Basal layer | *C77080* | 0.26 | 4.19E-03 |
| Basal layer | *Sf3b2* | 0.26 | 4.19E-03 |
| Basal layer | *Eif3i* | 0.30 | 4.22E-03 |
| Basal layer | *Fbxl14* | 0.26 | 5.11E-03 |
| Basal layer | *Wnt4* | 0.29 | 5.23E-03 |
| Basal layer | *Gdi2* | 0.25 | 5.75E-03 |
| Basal layer | *2610528A11Rik* | 0.40 | 6.03E-03 |
| Basal layer | *Skp1a* | 0.29 | 6.26E-03 |
| Basal layer | *U2af1* | 0.29 | 6.43E-03 |
| Basal layer | *Ctnnb1* | 0.26 | 6.48E-03 |
| Basal layer | *Shkbp1* | 0.31 | 6.53E-03 |
| Basal layer | *Dtymk* | 0.25 | 6.70E-03 |
| Basal layer | *Psma2* | 0.26 | 7.86E-03 |
| Basal layer | *Slc10a7* | 0.28 | 7.97E-03 |
| Basal layer | *Rcc2* | 0.26 | 8.38E-03 |
| Basal layer | *Pycard* | 0.27 | 8.84E-03 |
| Basal layer | *Rapgefl1* | 0.28 | 8.94E-03 |
| Basal layer | *Prrc2b* | 0.32 | 9.36E-03 |
| Basal layer | *Lama5* | 0.33 | 9.96E-03 |
| Basal layer | *Mxd4* | 0.29 | 9.97E-03 |
| Basal layer | *Eif3l* | 0.26 | 1.00E-02 |
| Basal layer | *Hadha* | 0.33 | 1.06E-02 |
| Basal layer | *Eif3h* | 0.26 | 1.14E-02 |
| Basal layer | *Cluh* | 0.27 | 1.14E-02 |
| Basal layer | *Tomm40* | 0.35 | 1.22E-02 |
| Basal layer | *Eif1a* | 0.26 | 1.32E-02 |
| Basal layer | *Mrpl23* | 0.25 | 1.47E-02 |
| Basal layer | *Psmb10* | 0.31 | 1.59E-02 |
| Basal layer | *Gstm5* | 0.28 | 1.72E-02 |
| Basal layer | *Map11* | 0.34 | 1.76E-02 |
| Basal layer | *Anapc2* | 0.33 | 2.35E-02 |
| Basal layer | *Aamp* | 0.34 | 2.68E-02 |
| Basal layer | *Ramac* | 0.32 | 2.92E-02 |
| Basal layer | *Cacybp* | 0.29 | 2.98E-02 |
| Basal layer | *Bola2* | 0.26 | 3.22E-02 |
| Basal layer | *Pfkp* | 0.31 | 3.25E-02 |
| Basal layer | *Nop10* | 0.34 | 3.31E-02 |
| Basal layer | *Irf3* | 0.31 | 3.49E-02 |
| Basal layer | *Pigt* | 0.31 | 3.51E-02 |
| Basal layer | *Pdik1l* | 0.32 | 3.61E-02 |
| Basal layer | *Ppp4r3a* | 0.30 | 4.32E-02 |
| Basal layer | *Gss* | 0.25 | 4.43E-02 |
| Muscularis mucosa | *Acta1* | 0.81 | 2.99E-54 |
| Muscularis mucosa | *Mylpf* | 0.93 | 7.19E-48 |
| Muscularis mucosa | *Tnnc2* | 1.02 | 2.45E-47 |
| Muscularis mucosa | *Tnnt3* | 0.92 | 3.87E-47 |
| Muscularis mucosa | *Gm42418* | 1.76 | 7.84E-47 |
| Muscularis mucosa | *Tnni2* | 1.05 | 6.72E-42 |
| Muscularis mucosa | *Ckm* | 0.86 | 8.75E-41 |
| Muscularis mucosa | *Myl3* | 0.91 | 2.43E-40 |
| Muscularis mucosa | *Myh8* | 0.99 | 8.59E-39 |
| Muscularis mucosa | *Aldoa* | 0.69 | 6.82E-38 |
| Muscularis mucosa | *Myh1* | 0.94 | 2.22E-34 |
| Muscularis mucosa | *Malat1* | 1.21 | 7.95E-31 |
| Muscularis mucosa | *Myl1* | 1.04 | 2.39E-30 |
| Muscularis mucosa | *Mb* | 0.70 | 1.28E-24 |
| Muscularis mucosa | *Tpm1* | 0.60 | 2.22E-21 |
| Muscularis mucosa | *Gapdh* | 0.55 | 5.17E-21 |
| Muscularis mucosa | *Bc1* | 1.17 | 2.37E-20 |
| Muscularis mucosa | *H19* | 0.80 | 1.96E-18 |
| Muscularis mucosa | *Lars2* | 1.49 | 2.38E-18 |
| Muscularis mucosa | *Cryab* | 0.82 | 2.47E-18 |
| Muscularis mucosa | *Eno3* | 0.88 | 2.75E-18 |
| Muscularis mucosa | *Tpm2* | 0.47 | 5.32E-18 |
| Muscularis mucosa | *Fhl1* | 0.67 | 6.68E-16 |
| Muscularis mucosa | *Pygm* | 0.84 | 1.09E-15 |
| Muscularis mucosa | *Ttn* | 0.72 | 2.52E-15 |
| Muscularis mucosa | *Sprr2j-ps* | 0.80 | 4.92E-15 |
| Muscularis mucosa | *Serpinb6a* | 0.59 | 1.42E-13 |
| Muscularis mucosa | *Atp2a1* | 0.64 | 2.00E-12 |
| Muscularis mucosa | *Actc1* | 0.93 | 8.69E-12 |
| Muscularis mucosa | *Tcap* | 0.61 | 1.69E-11 |
| Muscularis mucosa | *Sln* | 0.51 | 4.76E-11 |
| Muscularis mucosa | *Pfkm* | 0.84 | 1.75E-10 |
| Muscularis mucosa | *Lor* | 0.32 | 3.22E-10 |
| Muscularis mucosa | *Neb* | 0.81 | 3.66E-09 |
| Muscularis mucosa | *Slc25a4* | 0.61 | 4.26E-09 |
| Muscularis mucosa | *Hspb6* | 0.74 | 6.69E-09 |
| Muscularis mucosa | *Des* | 0.48 | 3.64E-07 |
| Muscularis mucosa | *Cox6a2* | 0.62 | 4.19E-07 |
| Muscularis mucosa | *Igfbp5* | 0.72 | 6.00E-07 |
| Muscularis mucosa | *Csrp3* | 0.75 | 7.09E-07 |
| Muscularis mucosa | *Tgm3* | 0.41 | 9.59E-07 |
| Muscularis mucosa | *Asprv1* | 0.49 | 6.83E-06 |
| Muscularis mucosa | *Aldh3a1* | 0.38 | 1.03E-04 |
| Muscularis mucosa | *Krt4* | 0.30 | 1.03E-04 |
| Muscularis mucosa | *Sparcl1* | 0.60 | 1.58E-04 |
| Muscularis mucosa | *Ada* | 0.43 | 1.95E-04 |
| Muscularis mucosa | *Clptm1* | 0.57 | 2.68E-04 |
| Muscularis mucosa | *Car3* | 0.59 | 4.22E-04 |
| Muscularis mucosa | *Ddx49* | 1.19 | 5.08E-04 |
| Muscularis mucosa | *Psme4* | 0.76 | 1.13E-03 |
| Muscularis mucosa | *Ptgr1* | 0.34 | 1.55E-03 |
| Muscularis mucosa | *Kmt2b* | 0.78 | 2.59E-03 |
| Muscularis mucosa | *Sphk2* | 0.75 | 4.06E-03 |
| Muscularis mucosa | *Gnb1* | 0.54 | 4.61E-03 |
| Muscularis mucosa | *Serf2* | 0.34 | 4.94E-03 |
| Muscularis mucosa | *Pnn* | 0.69 | 7.23E-03 |
| Muscularis mucosa | *Ldha* | 0.28 | 9.44E-03 |
| Muscularis mucosa | *2310022B05Rik* | 0.63 | 9.45E-03 |
| Muscularis mucosa | *Atp5e* | 0.26 | 1.24E-02 |
| Muscularis mucosa | *Ak1* | 0.64 | 1.89E-02 |
| Muscularis mucosa | *Col3a1* | 0.28 | 3.85E-02 |
| Muscularis mucosa | *Reps1* | 1.36 | 4.12E-02 |
| Submucosa | *Mgp* | 1.50 | 2.43E-37 |
| Submucosa | *Fn1* | 1.98 | 5.32E-36 |
| Submucosa | *Crip1* | 1.34 | 1.20E-34 |
| Submucosa | *Col1a2* | 2.04 | 1.02E-33 |
| Submucosa | *Dcn* | 1.45 | 1.09E-32 |
| Submucosa | *Gsn* | 1.21 | 8.60E-32 |
| Submucosa | *C4b* | 1.78 | 1.24E-31 |
| Submucosa | *Pi16* | 1.67 | 7.36E-30 |
| Submucosa | *Col3a1* | 1.71 | 8.76E-30 |
| Submucosa | *Col1a1* | 2.15 | 3.56E-28 |
| Submucosa | *Sparc* | 1.63 | 9.21E-28 |
| Submucosa | *C3* | 1.30 | 9.10E-27 |
| Submucosa | *Fbn1* | 1.63 | 7.74E-26 |
| Submucosa | *Ly6a* | 0.86 | 1.04E-23 |
| Submucosa | *C1qa* | 1.48 | 1.08E-23 |
| Submucosa | *Igfbp6* | 1.24 | 1.34E-23 |
| Submucosa | *Ly6c1* | 1.22 | 1.68E-23 |
| Submucosa | *Postn* | 1.85 | 6.15E-23 |
| Submucosa | *Bgn* | 1.32 | 2.95E-22 |
| Submucosa | *Vim* | 1.28 | 1.04E-20 |
| Submucosa | *Fstl1* | 1.45 | 5.11E-20 |
| Submucosa | *C1ra* | 1.14 | 6.90E-20 |
| Submucosa | *Loxl1* | 1.23 | 1.22E-19 |
| Submucosa | *Lum* | 1.16 | 4.82E-19 |
| Submucosa | *Tnxb* | 1.17 | 5.17E-19 |
| Submucosa | *Cd74* | 1.26 | 2.36E-18 |
| Submucosa | *Col5a1* | 1.06 | 1.67E-17 |
| Submucosa | *Apoe* | 0.86 | 1.00E-16 |
| Submucosa | *Ccl6* | 1.54 | 1.06E-16 |
| Submucosa | *Igfbp4* | 1.09 | 1.53E-16 |
| Submucosa | *Serpinh1* | 1.04 | 1.32E-15 |
| Submucosa | *C1qc* | 1.16 | 1.46E-15 |
| Submucosa | *Calu* | 0.93 | 1.93E-15 |
| Submucosa | *Col14a1* | 1.05 | 3.66E-15 |
| Submucosa | *Fbln1* | 1.13 | 9.35E-15 |
| Submucosa | *Mfap5* | 1.00 | 1.02E-14 |
| Submucosa | *Lgals1* | 0.82 | 1.11E-14 |
| Submucosa | *Aspn* | 1.11 | 1.41E-14 |
| Submucosa | *Retnla* | 2.05 | 1.94E-14 |
| Submucosa | *Sfrp4* | 0.51 | 2.50E-14 |
| Submucosa | *Prelp* | 1.01 | 5.01E-14 |
| Submucosa | *Itih5* | 0.99 | 7.95E-14 |
| Submucosa | *Cavin1* | 0.88 | 2.10E-13 |
| Submucosa | *Cst3* | 0.61 | 2.14E-13 |
| Submucosa | *Tmsb4x* | 0.60 | 2.28E-13 |
| Submucosa | *B2m* | 0.56 | 2.62E-13 |
| Submucosa | *Rcn3* | 1.02 | 4.19E-13 |
| Submucosa | *Col5a2* | 1.17 | 1.02E-12 |
| Submucosa | *H2-Ab1* | 0.86 | 1.62E-12 |
| Submucosa | *Ctsz* | 0.91 | 2.58E-12 |
| Submucosa | *Clec3b* | 0.97 | 2.92E-12 |
| Submucosa | *Igfbp7* | 0.81 | 4.58E-12 |
| Submucosa | *Ccn3* | 0.79 | 5.06E-12 |
| Submucosa | *Lyz2* | 1.08 | 5.63E-12 |
| Submucosa | *Hspg2* | 0.87 | 7.92E-12 |
| Submucosa | *Ltbp4* | 0.67 | 8.44E-12 |
| Submucosa | *Arhgdia* | 0.55 | 1.02E-11 |
| Submucosa | *C1qb* | 1.19 | 1.41E-11 |
| Submucosa | *Dpt* | 1.00 | 3.03E-11 |
| Submucosa | *Col6a1* | 0.85 | 4.49E-11 |
| Submucosa | *Htra3* | 0.97 | 6.33E-11 |
| Submucosa | *Col6a2* | 0.87 | 8.94E-11 |
| Submucosa | *Sod3* | 0.90 | 1.10E-10 |
| Submucosa | *Timp3* | 0.99 | 1.26E-10 |
| Submucosa | *Flna* | 0.83 | 3.01E-10 |
| Submucosa | *Fxyd5* | 0.83 | 4.51E-10 |
| Submucosa | *Mrc1* | 0.90 | 4.79E-10 |
| Submucosa | *Eln* | 0.96 | 6.80E-10 |
| Submucosa | *F13a1* | 0.80 | 1.15E-09 |
| Submucosa | *Timp2* | 0.81 | 1.15E-09 |
| Submucosa | *Psap* | 0.51 | 1.31E-09 |
| Submucosa | *Heg1* | 0.86 | 1.44E-09 |
| Submucosa | *Ccl8* | 0.74 | 3.00E-09 |
| Submucosa | *Dpysl2* | 0.77 | 4.13E-09 |
| Submucosa | *Serinc3* | 0.63 | 7.10E-09 |
| Submucosa | *Cd248* | 1.04 | 9.23E-09 |
| Submucosa | *Zcchc24* | 0.73 | 1.01E-08 |
| Submucosa | *Tm4sf1* | 0.71 | 2.02E-08 |
| Submucosa | *Pcolce* | 0.85 | 2.23E-08 |
| Submucosa | *Ifitm3* | 0.52 | 2.43E-08 |
| Submucosa | *Acta2* | 0.95 | 2.52E-08 |
| Submucosa | *H2-Aa* | 0.84 | 3.39E-08 |
| Submucosa | *Fth1* | 0.40 | 4.26E-08 |
| Submucosa | *Timp1* | 0.80 | 6.07E-08 |
| Submucosa | *Spon2* | 0.90 | 7.20E-08 |
| Submucosa | *Ccn5* | 1.17 | 7.31E-08 |
| Submucosa | *Sulf2* | 0.76 | 7.90E-08 |
| Submucosa | *Thbs1* | 0.81 | 8.05E-08 |
| Submucosa | *Lamc1* | 0.90 | 8.14E-08 |
| Submucosa | *Aebp1* | 0.69 | 8.25E-08 |
| Submucosa | *Cd34* | 0.86 | 1.03E-07 |
| Submucosa | *Cfh* | 0.71 | 1.22E-07 |
| Submucosa | *Tmsb10* | 0.48 | 1.23E-07 |
| Submucosa | *Tgfbr2* | 0.74 | 1.24E-07 |
| Submucosa | *Tagln* | 1.21 | 1.25E-07 |
| Submucosa | *Ifi27l2a* | 0.61 | 1.72E-07 |
| Submucosa | *Fhl1* | 0.47 | 2.33E-07 |
| Submucosa | *Mustn1* | 0.77 | 3.12E-07 |
| Submucosa | *Lsp1* | 0.71 | 3.63E-07 |
| Submucosa | *Adamts2* | 0.53 | 4.51E-07 |
| Submucosa | *Col6a3* | 0.74 | 4.65E-07 |
| Submucosa | *Serpinf1* | 0.70 | 5.06E-07 |
| Submucosa | *Sparcl1* | 0.66 | 5.25E-07 |
| Submucosa | *Olfml2b* | 0.53 | 5.45E-07 |
| Submucosa | *Islr* | 0.60 | 5.62E-07 |
| Submucosa | *Ddr2* | 0.49 | 6.80E-07 |
| Submucosa | *Ctsb* | 0.57 | 7.89E-07 |
| Submucosa | *Tpm1* | 0.35 | 8.79E-07 |
| Submucosa | *Loxl2* | 0.83 | 9.43E-07 |
| Submucosa | *Thy1* | 0.73 | 1.01E-06 |
| Submucosa | *Adgrd1* | 0.68 | 1.42E-06 |
| Submucosa | *Rarres2* | 0.71 | 1.99E-06 |
| Submucosa | *Des* | 0.55 | 2.15E-06 |
| Submucosa | *Emp3* | 0.73 | 2.81E-06 |
| Submucosa | *Tle5* | 0.48 | 3.82E-06 |
| Submucosa | *Ifitm2* | 0.56 | 4.19E-06 |
| Submucosa | *Rhoj* | 0.71 | 4.53E-06 |
| Submucosa | *Vsir* | 0.55 | 5.44E-06 |
| Submucosa | *Ccl7* | 0.46 | 6.34E-06 |
| Submucosa | *Col8a1* | 0.71 | 8.03E-06 |
| Submucosa | *Tsc22d3* | 0.69 | 8.21E-06 |
| Submucosa | *Lox* | 0.75 | 9.22E-06 |
| Submucosa | *Ly6e* | 0.53 | 9.36E-06 |
| Submucosa | *Ckb* | 0.65 | 9.74E-06 |
| Submucosa | *Laptm5* | 0.75 | 1.35E-05 |
| Submucosa | *Tgm2* | 0.55 | 1.47E-05 |
| Submucosa | *Mxra7* | 0.45 | 1.52E-05 |
| Submucosa | *Ccl9* | 0.89 | 1.70E-05 |
| Submucosa | *Cd302* | 0.75 | 1.77E-05 |
| Submucosa | *Ly86* | 0.44 | 1.84E-05 |
| Submucosa | *Col4a1* | 0.69 | 1.91E-05 |
| Submucosa | *Rbms3* | 0.44 | 1.99E-05 |
| Submucosa | *Axl* | 0.63 | 2.01E-05 |
| Submucosa | *Scara3* | 0.63 | 2.99E-05 |
| Submucosa | *Anxa5* | 0.60 | 3.27E-05 |
| Submucosa | *Ackr3* | 0.76 | 3.50E-05 |
| Submucosa | *Itgam* | 0.61 | 3.73E-05 |
| Submucosa | *Zbtb20* | 0.62 | 3.82E-05 |
| Submucosa | *Klf2* | 0.75 | 4.07E-05 |
| Submucosa | *Fxyd1* | 0.57 | 4.41E-05 |
| Submucosa | *Serpinb6a* | 0.37 | 4.73E-05 |
| Submucosa | *H2-Eb1* | 0.84 | 4.91E-05 |
| Submucosa | *Nfatc4* | 0.51 | 5.19E-05 |
| Submucosa | *Ptms* | 0.43 | 5.93E-05 |
| Submucosa | *Efemp1* | 0.70 | 6.24E-05 |
| Submucosa | *Tppp3* | 0.47 | 7.82E-05 |
| Submucosa | *S100a13* | 0.55 | 8.56E-05 |
| Submucosa | *Capns1* | 0.49 | 9.71E-05 |
| Submucosa | *Atp5e* | 0.38 | 1.33E-04 |
| Submucosa | *Cilp* | 0.71 | 1.44E-04 |
| Submucosa | *Fkbp1a* | 0.55 | 1.55E-04 |
| Submucosa | *Tpm2* | 0.34 | 1.64E-04 |
| Submucosa | *C1s1* | 0.80 | 1.66E-04 |
| Submucosa | *Sfrp1* | 0.71 | 1.73E-04 |
| Submucosa | *Sulf1* | 0.48 | 2.12E-04 |
| Submucosa | *Cfb* | 0.57 | 2.17E-04 |
| Submucosa | *Sptbn1* | 0.53 | 2.21E-04 |
| Submucosa | *Nid1* | 0.62 | 2.38E-04 |
| Submucosa | *Adamts5* | 0.52 | 2.66E-04 |
| Submucosa | *Myl6* | 0.40 | 3.21E-04 |
| Submucosa | *Pam* | 0.73 | 3.66E-04 |
| Submucosa | *Ntn1* | 0.58 | 3.96E-04 |
| Submucosa | *Scara5* | 0.65 | 4.68E-04 |
| Submucosa | *H2-K1* | 0.36 | 5.45E-04 |
| Submucosa | *Tnni2* | 0.36 | 5.87E-04 |
| Submucosa | *Efemp2* | 0.58 | 6.63E-04 |
| Submucosa | *Ubb* | 0.31 | 6.83E-04 |
| Submucosa | *Plekho2* | 0.47 | 7.24E-04 |
| Submucosa | *Rian* | 0.27 | 7.25E-04 |
| Submucosa | *Pid1* | 0.39 | 9.25E-04 |
| Submucosa | *Emilin2* | 0.52 | 1.02E-03 |
| Submucosa | *Lbh* | 0.61 | 1.05E-03 |
| Submucosa | *Pmp22* | 0.68 | 1.07E-03 |
| Submucosa | *Tnnt3* | 0.35 | 1.09E-03 |
| Submucosa | *9530068E07Rik* | 0.46 | 1.09E-03 |
| Submucosa | *Adgre1* | 0.43 | 1.10E-03 |
| Submucosa | *Ccr2* | 0.46 | 1.21E-03 |
| Submucosa | *Calm3* | 0.47 | 1.25E-03 |
| Submucosa | *Rnase4* | 0.63 | 1.33E-03 |
| Submucosa | *Myl3* | 0.43 | 1.55E-03 |
| Submucosa | *Cd93* | 0.45 | 1.64E-03 |
| Submucosa | *Lamp1* | 0.34 | 1.82E-03 |
| Submucosa | *Myh11* | 0.85 | 1.84E-03 |
| Submucosa | *Cfp* | 0.55 | 2.03E-03 |
| Submucosa | *Plpp3* | 0.60 | 2.14E-03 |
| Submucosa | *Ttn* | 0.58 | 2.33E-03 |
| Submucosa | *Cyb5r3* | 0.51 | 2.87E-03 |
| Submucosa | *Dio2* | 0.52 | 3.29E-03 |
| Submucosa | *Mylpf* | 0.40 | 3.35E-03 |
| Submucosa | *Grcc10* | 0.41 | 3.61E-03 |
| Submucosa | *Stard8* | 0.34 | 4.07E-03 |
| Submucosa | *Myl9* | 0.84 | 5.21E-03 |
| Submucosa | *Maged1* | 0.56 | 5.48E-03 |
| Submucosa | *Rhoc* | 0.52 | 5.53E-03 |
| Submucosa | *Ckm* | 0.29 | 5.62E-03 |
| Submucosa | *Hspa8* | 0.28 | 5.87E-03 |
| Submucosa | *Cox4i1* | 0.29 | 5.90E-03 |
| Submucosa | *Ncf1* | 0.44 | 5.95E-03 |
| Submucosa | *Dkk2* | 0.54 | 6.30E-03 |
| Submucosa | *Pltp* | 0.65 | 6.59E-03 |
| Submucosa | *Tspo* | 0.48 | 6.98E-03 |
| Submucosa | *Colec12* | 0.47 | 8.75E-03 |
| Submucosa | *Sdc2* | 0.53 | 8.77E-03 |
| Submucosa | *Ptgis* | 0.68 | 9.13E-03 |
| Submucosa | *Tmem176a* | 0.61 | 9.88E-03 |
| Submucosa | *Col12a1* | 0.59 | 9.90E-03 |
| Submucosa | *S100a4* | 0.67 | 1.01E-02 |
| Submucosa | *Eid1* | 0.49 | 1.02E-02 |
| Submucosa | *Tuba1a* | 0.56 | 1.07E-02 |
| Submucosa | *Sh3bgrl* | 0.61 | 1.24E-02 |
| Submucosa | *Cd55* | 0.55 | 1.27E-02 |
| Submucosa | *Pcolce2* | 0.39 | 1.42E-02 |
| Submucosa | *Calm2* | 0.57 | 1.44E-02 |
| Submucosa | *Txnip* | 0.42 | 1.56E-02 |
| Submucosa | *Ackr4* | 0.47 | 1.61E-02 |
| Submucosa | *Col4a2* | 0.67 | 1.67E-02 |
| Submucosa | *Ugp2* | 0.38 | 1.68E-02 |
| Submucosa | *H2-DMb1* | 0.56 | 1.91E-02 |
| Submucosa | *Acta1* | 0.30 | 1.91E-02 |
| Submucosa | *Mbnl1* | 0.45 | 2.16E-02 |
| Submucosa | *Ssc5d* | 0.34 | 2.32E-02 |
| Submucosa | *Pbxip1* | 0.63 | 2.49E-02 |
| Submucosa | *Gm13889* | 0.52 | 2.61E-02 |
| Submucosa | *Ptafr* | 0.42 | 2.76E-02 |
| Submucosa | *Vcan* | 0.41 | 2.81E-02 |
| Submucosa | *Cpq* | 0.47 | 2.98E-02 |
| Submucosa | *Fbln2* | 0.69 | 3.09E-02 |
| Submucosa | *Tgfbr3* | 0.62 | 3.27E-02 |
| Submucosa | *Slit3* | 0.49 | 3.32E-02 |
| Submucosa | *Cyba* | 0.69 | 3.94E-02 |
| Submucosa | *Ctss* | 0.58 | 4.09E-02 |
| Submucosa | *Itm2c* | 0.58 | 4.48E-02 |
| ESCC development region | *S100a8* | 1.81 | 1.23E-115 |
| ESCC development region | *S100a9* | 2.14 | 1.28E-115 |
| ESCC development region | *Cstdc5* | 2.23 | 1.97E-107 |
| ESCC development region | *Sprr2d* | 1.90 | 3.40E-106 |
| ESCC development region | *Krt6b* | 1.26 | 6.79E-97 |
| ESCC development region | *Sprr2i* | 1.66 | 1.94E-95 |
| ESCC development region | *Krt6a* | 1.00 | 2.61E-87 |
| ESCC development region | *Krt16* | 1.52 | 2.97E-87 |
| ESCC development region | *Dsg3* | 1.00 | 1.66E-82 |
| ESCC development region | *Sprr2h* | 1.49 | 3.87E-82 |
| ESCC development region | *Dsp* | 0.98 | 9.01E-82 |
| ESCC development region | *Anxa1* | 1.22 | 1.10E-81 |
| ESCC development region | *Sprr2f* | 1.21 | 1.68E-71 |
| ESCC development region | *Pdzk1ip1* | 0.86 | 2.25E-66 |
| ESCC development region | *Klk13* | 0.99 | 1.16E-64 |
| ESCC development region | *Krt14* | 0.88 | 1.02E-63 |
| ESCC development region | *Sprr2b* | 0.98 | 7.79E-61 |
| ESCC development region | *Chil4* | 1.20 | 2.19E-60 |
| ESCC development region | *Gm49980* | 0.76 | 4.12E-59 |
| ESCC development region | *Uox* | 0.90 | 1.03E-58 |
| ESCC development region | *Rbm3* | 0.84 | 1.38E-58 |
| ESCC development region | *Sprr1b* | 1.01 | 1.56E-58 |
| ESCC development region | *Tmprss11b* | 1.01 | 3.90E-58 |
| ESCC development region | *Slc5a1* | 0.94 | 1.23E-57 |
| ESCC development region | *Gk* | 0.94 | 8.68E-56 |
| ESCC development region | *Krt17* | 1.58 | 3.96E-55 |
| ESCC development region | *Urah* | 0.91 | 6.83E-54 |
| ESCC development region | *Sprr1a* | 0.87 | 9.02E-54 |
| ESCC development region | *Cfd* | 1.09 | 2.47E-53 |
| ESCC development region | *Ceacam1* | 0.99 | 3.50E-53 |
| ESCC development region | *Cyp51* | 0.87 | 4.14E-53 |
| ESCC development region | *S100a11* | 0.63 | 1.14E-52 |
| ESCC development region | *Tmprss11g* | 0.84 | 1.10E-51 |
| ESCC development region | *Defb3* | 0.99 | 1.92E-51 |
| ESCC development region | *Nat8l* | 0.86 | 1.00E-50 |
| ESCC development region | *Atf4* | 0.73 | 1.36E-50 |
| ESCC development region | *Fabp4* | 0.83 | 1.70E-50 |
| ESCC development region | *Ltf* | 1.56 | 2.02E-50 |
| ESCC development region | *Scd2* | 0.76 | 1.10E-48 |
| ESCC development region | *Ly6d* | 0.60 | 3.13E-47 |
| ESCC development region | *Rab11a* | 0.64 | 8.87E-47 |
| ESCC development region | *Lypd3* | 0.57 | 7.14E-46 |
| ESCC development region | *Slc6a14* | 0.80 | 3.10E-45 |
| ESCC development region | *Sprr2e* | 0.76 | 1.96E-44 |
| ESCC development region | *Ighm* | 0.80 | 8.46E-44 |
| ESCC development region | *Tnfaip2* | 0.79 | 3.75E-43 |
| ESCC development region | *Il1rn* | 0.80 | 7.65E-43 |
| ESCC development region | *Stom* | 0.73 | 1.82E-41 |
| ESCC development region | *Cyp2e1* | 0.59 | 9.87E-41 |
| ESCC development region | *Blmh* | 0.71 | 2.60E-40 |
| ESCC development region | *Dsc2* | 0.67 | 5.73E-40 |
| ESCC development region | *Tuba1c* | 0.59 | 1.77E-39 |
| ESCC development region | *Sprr2a3* | 0.75 | 2.28E-39 |
| ESCC development region | *Cartpt* | 0.60 | 2.91E-39 |
| ESCC development region | *Rnase2b* | 0.67 | 4.52E-39 |
| ESCC development region | *Glul* | 0.70 | 4.50E-38 |
| ESCC development region | *Igkc* | 0.68 | 2.31E-37 |
| ESCC development region | *Gjb2* | 0.65 | 1.05E-35 |
| ESCC development region | *Lcn2* | 0.57 | 1.23E-35 |
| ESCC development region | *Hif1a* | 0.66 | 2.01E-35 |
| ESCC development region | *AA467197* | 0.78 | 1.65E-34 |
| ESCC development region | *Klf5* | 0.38 | 3.80E-34 |
| ESCC development region | *Retn* | 0.61 | 5.81E-34 |
| ESCC development region | *Junb* | 0.70 | 1.02E-33 |
| ESCC development region | *Csta1* | 0.69 | 6.27E-33 |
| ESCC development region | *Scd1* | 0.63 | 1.00E-32 |
| ESCC development region | *Pglyrp1* | 0.55 | 1.92E-32 |
| ESCC development region | *Mal* | 0.68 | 8.54E-32 |
| ESCC development region | *Cdh1* | 0.58 | 2.75E-31 |
| ESCC development region | *Sbno2* | 0.63 | 5.42E-31 |
| ESCC development region | *Nectin4* | 0.58 | 8.70E-30 |
| ESCC development region | *Klk10* | 0.53 | 1.03E-29 |
| ESCC development region | *Gal* | 0.43 | 2.27E-29 |
| ESCC development region | *Cidec* | 0.46 | 3.31E-28 |
| ESCC development region | *Upp1* | 0.54 | 4.04E-28 |
| ESCC development region | *Fasn* | 0.53 | 1.48E-27 |
| ESCC development region | *Stfa3* | 0.63 | 2.76E-27 |
| ESCC development region | *Cd24a* | 0.55 | 1.33E-26 |
| ESCC development region | *Vip* | 0.43 | 1.44E-26 |
| ESCC development region | *Ndufa4* | 0.47 | 1.72E-26 |
| ESCC development region | *Il1f6* | 0.62 | 2.37E-26 |
| ESCC development region | *Tgm1* | 0.56 | 3.48E-26 |
| ESCC development region | *Myh2* | 0.41 | 3.51E-26 |
| ESCC development region | *Npy* | 0.33 | 6.12E-26 |
| ESCC development region | *Srsf2* | 0.48 | 1.35E-25 |
| ESCC development region | *Klf4* | 0.53 | 3.55E-25 |
| ESCC development region | *Lamc2* | 1.41 | 5.30E-25 |
| ESCC development region | *Anxa8* | 0.61 | 5.34E-25 |
| ESCC development region | *Tgfa* | 0.54 | 1.33E-24 |
| ESCC development region | *Adam28* | 0.53 | 1.58E-24 |
| ESCC development region | *Mmp9* | 1.29 | 2.40E-24 |
| ESCC development region | *Bpifc* | 0.54 | 5.13E-24 |
| ESCC development region | *Anxa2* | 0.51 | 6.64E-24 |
| ESCC development region | *Pglyrp4* | 0.54 | 6.79E-24 |
| ESCC development region | *Ankk1* | 0.30 | 8.50E-24 |
| ESCC development region | *Mmp13* | 0.88 | 1.28E-23 |
| ESCC development region | *Psca* | 0.74 | 1.44E-23 |
| ESCC development region | *Id1* | 0.53 | 1.79E-23 |
| ESCC development region | *Vmp1* | 0.51 | 2.97E-23 |
| ESCC development region | *Plet1* | 0.55 | 5.25E-23 |
| ESCC development region | *Chchd2* | 0.35 | 1.14E-22 |
| ESCC development region | *1810037I17Rik* | 0.44 | 2.22E-22 |
| ESCC development region | *Cpe* | 0.50 | 2.30E-22 |
| ESCC development region | *Epha2* | 0.47 | 2.52E-22 |
| ESCC development region | *Pmvk* | 0.44 | 3.92E-22 |
| ESCC development region | *Myl9* | 0.39 | 7.34E-22 |
| ESCC development region | *Msmo1* | 0.45 | 8.66E-22 |
| ESCC development region | *Jup* | 0.44 | 1.05E-21 |
| ESCC development region | *Idi1* | 0.53 | 1.55E-21 |
| ESCC development region | *Cxcl3* | 0.51 | 2.19E-21 |
| ESCC development region | *Ldlr* | 0.46 | 2.39E-21 |
| ESCC development region | *Dstn* | 0.34 | 2.56E-21 |
| ESCC development region | *Psors1c2* | 0.61 | 3.01E-21 |
| ESCC development region | *Nectin1* | 0.44 | 3.30E-21 |
| ESCC development region | *Hmgcs1* | 0.48 | 4.55E-21 |
| ESCC development region | *Tmem45b* | 0.42 | 1.33E-20 |
| ESCC development region | *Mapk6* | 0.51 | 1.63E-20 |
| ESCC development region | *Iffo2* | 0.47 | 2.53E-20 |
| ESCC development region | *Hdgf* | 0.43 | 2.60E-20 |
| ESCC development region | *Vamp8* | 0.46 | 4.27E-20 |
| ESCC development region | *Nrarp* | 0.43 | 4.76E-20 |
| ESCC development region | *S100a16* | 0.31 | 4.99E-20 |
| ESCC development region | *Oit1* | 0.48 | 6.54E-20 |
| ESCC development region | *Fam162a* | 0.45 | 8.08E-20 |
| ESCC development region | *Thrsp* | 0.35 | 9.46E-20 |
| ESCC development region | *Adipoq* | 0.33 | 1.08E-19 |
| ESCC development region | *Gm26532* | 0.38 | 1.18E-19 |
| ESCC development region | *Cd44* | 0.47 | 1.51E-19 |
| ESCC development region | *Defb14* | 0.48 | 2.27E-19 |
| ESCC development region | *2310046K23Rik* | 0.51 | 3.07E-19 |
| ESCC development region | *Tmbim6* | 0.36 | 5.37E-19 |
| ESCC development region | *Hbegf* | 0.50 | 5.83E-19 |
| ESCC development region | *Mindy1* | 0.45 | 6.44E-19 |
| ESCC development region | *Spint2* | 0.41 | 7.71E-19 |
| ESCC development region | *Arg1* | 1.00 | 1.66E-18 |
| ESCC development region | *Slc5a8* | 0.30 | 1.84E-18 |
| ESCC development region | *Eppk1* | 0.46 | 3.61E-18 |
| ESCC development region | *Actg2* | 0.38 | 3.90E-18 |
| ESCC development region | *Klk12* | 0.43 | 4.20E-18 |
| ESCC development region | *Pnpla2* | 0.38 | 4.52E-18 |
| ESCC development region | *Txn1* | 0.39 | 4.59E-18 |
| ESCC development region | *Atp6v1a* | 0.38 | 6.42E-18 |
| ESCC development region | *Fabp5* | 0.27 | 7.34E-18 |
| ESCC development region | *Slc25a1* | 0.38 | 8.16E-18 |
| ESCC development region | *Myh11* | 0.35 | 1.20E-17 |
| ESCC development region | *Acta2* | 0.29 | 1.21E-17 |
| ESCC development region | *Sema3f* | 0.45 | 1.40E-17 |
| ESCC development region | *Prr13* | 0.42 | 3.29E-17 |
| ESCC development region | *Slc2a1* | 0.50 | 3.53E-17 |
| ESCC development region | *S100a6* | 0.58 | 3.58E-17 |
| ESCC development region | *Arf5* | 0.42 | 3.83E-17 |
| ESCC development region | *Mvk* | 0.43 | 6.85E-17 |
| ESCC development region | *Furin* | 0.39 | 8.89E-17 |
| ESCC development region | *Il34* | 0.41 | 9.78E-17 |
| ESCC development region | *Ptbp3* | 0.41 | 1.35E-16 |
| ESCC development region | *Tmprss11d* | 0.52 | 1.39E-16 |
| ESCC development region | *Mal2* | 0.49 | 1.40E-16 |
| ESCC development region | *Higd1a* | 0.40 | 1.58E-16 |
| ESCC development region | *Lgi2* | 0.33 | 1.61E-16 |
| ESCC development region | *Ube2i* | 0.37 | 1.68E-16 |
| ESCC development region | *Cxcl5* | 0.41 | 2.21E-16 |
| ESCC development region | *Serpinb1a* | 0.40 | 2.23E-16 |
| ESCC development region | *Dip2b* | 0.38 | 3.74E-16 |
| ESCC development region | *Ppbp* | 0.63 | 4.80E-16 |
| ESCC development region | *Adig* | 0.32 | 5.43E-16 |
| ESCC development region | *Sppl3* | 0.36 | 6.76E-16 |
| ESCC development region | *Acly* | 0.37 | 6.78E-16 |
| ESCC development region | *Neat1* | 0.32 | 1.16E-15 |
| ESCC development region | *Cast* | 0.43 | 1.31E-15 |
| ESCC development region | *Ighg3* | 0.27 | 1.91E-15 |
| ESCC development region | *Trf* | 0.32 | 2.69E-15 |
| ESCC development region | *Dgat1* | 0.34 | 5.83E-15 |
| ESCC development region | *Teddm3* | 0.47 | 6.77E-15 |
| ESCC development region | *Ptges* | 0.39 | 6.79E-15 |
| ESCC development region | *Slc6a20a* | 0.28 | 9.51E-15 |
| ESCC development region | *Ckap4* | 0.44 | 9.52E-15 |
| ESCC development region | *Wdr77* | 0.37 | 1.36E-14 |
| ESCC development region | *Plbd1* | 0.45 | 1.36E-14 |
| ESCC development region | *Stfa1* | 0.33 | 1.40E-14 |
| ESCC development region | *Hsd17b7* | 0.34 | 1.46E-14 |
| ESCC development region | *Cstb* | 0.41 | 1.63E-14 |
| ESCC development region | *Cdh3* | 0.34 | 1.79E-14 |
| ESCC development region | *Rab31* | 0.33 | 1.80E-14 |
| ESCC development region | *Serpinb2* | 0.35 | 1.80E-14 |
| ESCC development region | *Ppard* | 0.41 | 2.01E-14 |
| ESCC development region | *Cnn1* | 0.33 | 2.30E-14 |
| ESCC development region | *Sdc4* | 0.32 | 2.44E-14 |
| ESCC development region | *Hmgn1* | 0.33 | 2.48E-14 |
| ESCC development region | *Vsig8* | 0.42 | 2.49E-14 |
| ESCC development region | *Rcn1* | 0.39 | 2.82E-14 |
| ESCC development region | *Bhlhe40* | 0.41 | 3.66E-14 |
| ESCC development region | *Tent5a* | 0.34 | 4.11E-14 |
| ESCC development region | *Duoxa2* | 0.26 | 4.33E-14 |
| ESCC development region | *Sqle* | 0.36 | 4.45E-14 |
| ESCC development region | *Atp2c2* | 0.36 | 4.54E-14 |
| ESCC development region | *Lamb3* | 0.98 | 6.43E-14 |
| ESCC development region | *H2-K1* | 0.30 | 8.74E-14 |
| ESCC development region | *Ppp1r13l* | 0.39 | 8.76E-14 |
| ESCC development region | *Endou* | 0.42 | 9.62E-14 |
| ESCC development region | *Sprr2k* | 0.29 | 1.07E-13 |
| ESCC development region | *Mboat1* | 0.32 | 1.08E-13 |
| ESCC development region | *Defb1* | 0.33 | 1.10E-13 |
| ESCC development region | *Alas1* | 0.36 | 1.27E-13 |
| ESCC development region | *Elf3* | 0.39 | 1.33E-13 |
| ESCC development region | *H1f0* | 0.39 | 2.01E-13 |
| ESCC development region | *Pxdc1* | 0.42 | 2.22E-13 |
| ESCC development region | *Erg28* | 0.38 | 2.62E-13 |
| ESCC development region | *Sdc1* | 0.32 | 3.30E-13 |
| ESCC development region | *Hmgcr* | 0.36 | 3.51E-13 |
| ESCC development region | *Acat2* | 0.34 | 4.93E-13 |
| ESCC development region | *Arpc5* | 0.37 | 5.93E-13 |
| ESCC development region | *Tubb2a* | 0.38 | 7.25E-13 |
| ESCC development region | *R3hdm4* | 0.36 | 7.38E-13 |
| ESCC development region | *Abracl* | 0.41 | 8.89E-13 |
| ESCC development region | *Slc1a5* | 0.28 | 1.02E-12 |
| ESCC development region | *Fgfbp1* | 0.39 | 1.21E-12 |
| ESCC development region | *Cxcl2* | 0.40 | 1.26E-12 |
| ESCC development region | *Fam167a* | 0.35 | 1.33E-12 |
| ESCC development region | *Hnrnpd* | 0.34 | 1.61E-12 |
| ESCC development region | *Tfap2a* | 0.34 | 1.69E-12 |
| ESCC development region | *Zc3h12a* | 0.33 | 2.43E-12 |
| ESCC development region | *Krt5* | 0.36 | 2.76E-12 |
| ESCC development region | *Sh3pxd2a* | 0.34 | 2.79E-12 |
| ESCC development region | *Ier3* | 0.44 | 2.87E-12 |
| ESCC development region | *Chil1* | 0.29 | 3.11E-12 |
| ESCC development region | *Aqp3* | 0.34 | 3.68E-12 |
| ESCC development region | *Ifi202b* | 0.40 | 5.71E-12 |
| ESCC development region | *Trafd1* | 0.30 | 6.36E-12 |
| ESCC development region | *Psmd2* | 0.34 | 6.39E-12 |
| ESCC development region | *Gsdmc* | 0.36 | 6.67E-12 |
| ESCC development region | *Marcksl1* | 0.38 | 9.77E-12 |
| ESCC development region | *Btf3* | 0.31 | 9.91E-12 |
| ESCC development region | *Calm4* | 0.34 | 1.05E-11 |
| ESCC development region | *Prdx5* | 0.34 | 1.13E-11 |
| ESCC development region | *Tmem265* | 0.28 | 1.22E-11 |
| ESCC development region | *Arf1* | 0.32 | 1.36E-11 |
| ESCC development region | *Igsf8* | 0.31 | 1.37E-11 |
| ESCC development region | *Ids* | 0.35 | 1.48E-11 |
| ESCC development region | *Myo1b* | 0.26 | 1.57E-11 |
| ESCC development region | *Cwh43* | 0.33 | 1.70E-11 |
| ESCC development region | *Galnt6* | 0.31 | 2.17E-11 |
| ESCC development region | *Mid1ip1* | 0.31 | 2.53E-11 |
| ESCC development region | *Lama3* | 0.83 | 3.04E-11 |
| ESCC development region | *Snhg3* | 0.34 | 3.87E-11 |
| ESCC development region | *Prss27* | 0.46 | 4.19E-11 |
| ESCC development region | *Klk6* | 0.28 | 5.87E-11 |
| ESCC development region | *Pthlh* | 0.31 | 7.01E-11 |
| ESCC development region | *Slc30a4* | 0.27 | 7.08E-11 |
| ESCC development region | *Degs1* | 0.32 | 1.00E-10 |
| ESCC development region | *Itga6* | 0.51 | 1.08E-10 |
| ESCC development region | *Sat1* | 0.33 | 1.19E-10 |
| ESCC development region | *Ovol1* | 0.31 | 1.47E-10 |
| ESCC development region | *Sema4d* | 0.30 | 1.55E-10 |
| ESCC development region | *Sh3kbp1* | 0.26 | 1.70E-10 |
| ESCC development region | *Runx1* | 0.26 | 1.91E-10 |
| ESCC development region | *Mvd* | 0.30 | 2.99E-10 |
| ESCC development region | *Hsd17b12* | 0.31 | 3.85E-10 |
| ESCC development region | *Fdft1* | 0.30 | 5.41E-10 |
| ESCC development region | *Rnf144b* | 0.27 | 6.26E-10 |
| ESCC development region | *Lss* | 0.33 | 7.23E-10 |
| ESCC development region | *Aacs* | 0.31 | 7.39E-10 |
| ESCC development region | *Cap1* | 0.34 | 8.58E-10 |
| ESCC development region | *Ddx3x* | 0.31 | 1.23E-09 |
| ESCC development region | *Ykt6* | 0.33 | 1.25E-09 |
| ESCC development region | *Plcd1* | 0.33 | 1.26E-09 |
| ESCC development region | *Snhg9* | 0.30 | 1.27E-09 |
| ESCC development region | *Tpm4* | 0.30 | 1.27E-09 |
| ESCC development region | *Cyp7b1* | 0.28 | 1.51E-09 |
| ESCC development region | *Atp6v0e* | 0.26 | 1.63E-09 |
| ESCC development region | *Ripk4* | 0.27 | 1.74E-09 |
| ESCC development region | *Guk1* | 0.29 | 2.81E-09 |
| ESCC development region | *Wsb1* | 0.29 | 3.07E-09 |
| ESCC development region | *Scrib* | 0.32 | 3.28E-09 |
| ESCC development region | *Hnrnpk* | 0.29 | 3.34E-09 |
| ESCC development region | *Mapk3* | 0.32 | 3.69E-09 |
| ESCC development region | *Degs2* | 0.27 | 4.00E-09 |
| ESCC development region | *Serpinb3a* | 0.30 | 4.48E-09 |
| ESCC development region | *Il1f9* | 0.32 | 6.04E-09 |
| ESCC development region | *Gjb6* | 0.34 | 6.93E-09 |
| ESCC development region | *Plau* | 0.29 | 7.33E-09 |
| ESCC development region | *Api5* | 0.30 | 8.13E-09 |
| ESCC development region | *Lipe* | 0.27 | 8.42E-09 |
| ESCC development region | *Nsdhl* | 0.28 | 8.52E-09 |
| ESCC development region | *Abhd17c* | 0.27 | 8.71E-09 |
| ESCC development region | *Clic1* | 0.31 | 9.84E-09 |
| ESCC development region | *Fat2* | 0.30 | 1.04E-08 |
| ESCC development region | *Mafb* | 0.28 | 1.07E-08 |
| ESCC development region | *Snrpd3* | 0.32 | 1.11E-08 |
| ESCC development region | *Fam43a* | 0.33 | 1.12E-08 |
| ESCC development region | *Wdr1* | 0.31 | 1.34E-08 |
| ESCC development region | *Chit1* | 0.37 | 1.56E-08 |
| ESCC development region | *Nme1* | 0.30 | 1.60E-08 |
| ESCC development region | *Ly6g6c* | 0.32 | 2.08E-08 |
| ESCC development region | *Tagln2* | 0.31 | 2.58E-08 |
| ESCC development region | *Borcs6* | 0.27 | 2.91E-08 |
| ESCC development region | *Nfkbia* | 0.40 | 3.71E-08 |
| ESCC development region | *Fam83f* | 0.27 | 3.79E-08 |
| ESCC development region | *Acadl* | 0.26 | 3.83E-08 |
| ESCC development region | *Dnajb6* | 0.26 | 4.56E-08 |
| ESCC development region | *BC005537* | 0.28 | 5.28E-08 |
| ESCC development region | *Dhrs1* | 0.30 | 6.74E-08 |
| ESCC development region | *Dynap* | 0.29 | 7.38E-08 |
| ESCC development region | *Pfn1* | 0.28 | 7.68E-08 |
| ESCC development region | *Actn1* | 0.33 | 1.23E-07 |
| ESCC development region | *F11r* | 0.25 | 1.28E-07 |
| ESCC development region | *Psmb3* | 0.31 | 1.65E-07 |
| ESCC development region | *Rgs12* | 0.28 | 1.93E-07 |
| ESCC development region | *Sptlc1* | 0.27 | 1.96E-07 |
| ESCC development region | *Krt1* | 0.39 | 2.07E-07 |
| ESCC development region | *Dhcr7* | 0.28 | 2.18E-07 |
| ESCC development region | *Fosl2* | 0.27 | 5.46E-07 |
| ESCC development region | *Pmepa1* | 0.29 | 6.49E-07 |
| ESCC development region | *Etv3* | 0.25 | 8.37E-07 |
| ESCC development region | *6430548M08Rik* | 0.29 | 8.45E-07 |
| ESCC development region | *Aplp2* | 0.28 | 8.83E-07 |
| ESCC development region | *Serpinb3b* | 0.26 | 9.16E-07 |
| ESCC development region | *Tnfrsf12a* | 0.25 | 9.47E-07 |
| ESCC development region | *Abi1* | 0.26 | 9.71E-07 |
| ESCC development region | *Tollip* | 0.26 | 1.09E-06 |
| ESCC development region | *Eif1ad* | 0.30 | 1.32E-06 |
| ESCC development region | *Inhba* | 0.31 | 1.33E-06 |
| ESCC development region | *Wfdc5* | 0.27 | 1.61E-06 |
| ESCC development region | *Cab39* | 0.28 | 1.68E-06 |
| ESCC development region | *Rbms1* | 0.28 | 2.97E-06 |
| ESCC development region | *Cxcl1* | 0.28 | 3.30E-06 |
| ESCC development region | *Psmd4* | 0.26 | 3.97E-06 |
| ESCC development region | *Irf6* | 0.27 | 3.97E-06 |
| ESCC development region | *Bdh1* | 0.25 | 4.47E-06 |
| ESCC development region | *Efhd2* | 0.31 | 4.62E-06 |
| ESCC development region | *2200002D01Rik* | 0.30 | 5.36E-06 |
| ESCC development region | *Prss22* | 0.35 | 5.57E-06 |
| ESCC development region | *Bzw1* | 0.25 | 1.03E-05 |
| ESCC development region | *Sc5d* | 0.27 | 1.20E-05 |
| ESCC development region | *Atp6v0a1* | 0.27 | 1.22E-05 |
| ESCC development region | *Cct8* | 0.26 | 1.25E-05 |
| ESCC development region | *Aldh1a3* | 0.27 | 1.66E-05 |
| ESCC development region | *Serp1* | 0.27 | 2.27E-05 |
| ESCC development region | *Ubxn1* | 0.28 | 2.46E-05 |
| ESCC development region | *Agap3* | 0.26 | 3.31E-05 |
| ESCC development region | *Ndrg1* | 0.32 | 6.05E-05 |
| ESCC development region | *Actn4* | 0.26 | 1.62E-04 |
| ESCC development region | *Igfbp3* | 0.39 | 3.22E-04 |
| ESCC development region | *Flnb* | 0.28 | 1.45E-03 |

| **Table S3. Ratios of ligand-receptor (LR) gene pairs enriched in 5 tissue regions of mouse spatial transcriptome data** | | | | | |
| --- | --- | --- | --- | --- | --- |
| LR gene pair | Ratio of SBL | Ratio of BL | Ratio of MM | Ratio of SM | Ratio of EDR |
| *Btc-Erbb3* | 0.13 | 0.07 | 0.03 | 0.03 | 0.07 |
| *Ereg-Egfr* | 0.13 | 0.02 | 0.05 | 0.02 | 0.08 |
| *Slpi-Plscr1* | 0.47 | 0.18 | 0.18 | 0.12 | 0.14 |
| *Slpi-Plscr4* | 0.54 | 0.26 | 0.27 | 0.14 | 0.16 |
| *Tnfsf13-Tnfrsf1a* | 0.15 | 0.07 | 0.06 | 0.05 | 0.04 |
| *Dlk2-Notch1* | 0.07 | 0.18 | 0.01 | 0.04 | 0.05 |
| *Dll1-Notch1* | 0.05 | 0.14 | 0.03 | 0.02 | 0.05 |
| *Efnb1-Ephb2* | 0.07 | 0.16 | 0.04 | 0.01 | 0.03 |
| *Efnb1-Ephb4* | 0.06 | 0.15 | 0.02 | 0.07 | 0.05 |
| *Jag2-Notch1* | 0.05 | 0.20 | 0.02 | 0.09 | 0.03 |
| *Bsg-Slc16a1* | 0.02 | 0.03 | 0.11 | 0.04 | 0.01 |
| *Bsg-Slc16a7* | 0.03 | 0.08 | 0.09 | 0.06 | 0.03 |
| *Dkk2-Lrp5* | 0.02 | 0.05 | 0.09 | 0.05 | 0.05 |
| *Dsc2-Dsg3* | 0.03 | 0.03 | 0.09 | 0.02 | 0.04 |
| *Pdgfb-Art1* | 0.01 | 0.00 | 0.09 | 0.06 | 0.04 |
| *Ccl8-Ackr4* | 0.03 | 0.02 | 0.01 | 0.23 | 0.03 |
| *Ccl8-Ccr2* | 0.03 | 0.05 | 0.02 | 0.28 | 0.02 |
| *Col1a1-Ddr2* | 0.01 | 0.01 | 0.01 | 0.25 | 0.04 |
| *Col3a1-Ddr2* | 0.02 | 0.02 | 0.03 | 0.28 | 0.02 |
| *Fn1-Itgb7* | 0.00 | 0.00 | 0.04 | 0.24 | 0.04 |
| *Afdn-Nectin1* | 0.04 | 0.02 | 0.05 | 0.00 | 0.11 |
| *Areg-Erbb3* | 0.03 | 0.05 | 0.04 | 0.04 | 0.09 |
| *Nectin1-Nectin1* | 0.13 | 0.05 | 0.13 | 0.07 | 0.20 |
| *S100a8-Tlr4* | 0.18 | 0.19 | 0.16 | 0.37 | 0.59 |
| *S100a9-Tlr4* | 0.20 | 0.17 | 0.16 | 0.32 | 0.59 |
| *SBL, suprabasal layer; BL, basal layer; MM, muscularis mucosa; SM, submucosa; EDR, ESCC development region. | | | | | |

| **Table S4. Spot counts of different tissue regions in human spatial transcriptome data** | | | |
| --- | --- | --- | --- |
| *Patient ID | *Disease stage | Tissue region | Spot counts |
| Pt #22 | NOR | Mucosa-submucosa | 569 |
|  |  | Muscularis | 810 |
|  | HGIN | Mucosa-submucosa | 210 |
|  |  | Muscularis | 341 |
|  | ESCC | Mucosa-submucosa | 515 |
|  |  | Muscularis | 0 |
| Pt #27 | LGIN | Mucosa-submucosa | 441 |
|  |  | Muscularis | 841 |
|  | HGIN | Mucosa-submucosa | 268 |
|  |  | Muscularis | 24 |
|  | ESCC | Mucosa-submucosa | 69 |
|  |  | Muscularis | 274 |
| Pt #28 | NOR | Mucosa-submucosa | 333 |
|  |  | Muscularis | 417 |
|  | HGIN | Mucosa-submucosa | 303 |
|  |  | Muscularis | 637 |
|  | ESCC | Mucosa-submucosa | 609 |
|  |  | Muscularis | 0 |
| Pt #29 | NOR | Mucosa-submucosa | 92 |
|  |  | Muscularis | 330 |
|  | HGIN | Mucosa-submucosa | 107 |
|  |  | Muscularis | 30 |
|  | ESCC | Mucosa-submucosa | 236 |
|  |  | Muscularis | 129 |
| *NOR, normal; LGIN, low-grade intraepithelial neoplasia; HGIN, high-grade intraepithelial neoplasia; ESCC, esophageal squamous cell carcinoma. Pt, Patient. | | | |

| **Table S5. *EFNB1* and *EPHB4* correlated genes in scRNA-seq data (GSE160269)** | | | |
| --- | --- | --- | --- |
| Gene1 | Gene2 | r | *P*-value |
| *EFNB1* | *AAMDC* | -0.53 | 1.06E-05 |
| *EFNB1* | *AARS* | 0.30 | 1.63E-02 |
| *EFNB1* | *ABCA1* | 0.46 | 1.98E-04 |
| *EFNB1* | *ABCB7* | 0.32 | 1.12E-02 |
| *EFNB1* | *ABCC1* | 0.40 | 1.27E-03 |
| *EFNB1* | *ABCC4* | 0.35 | 4.97E-03 |
| *EFNB1* | *ABCC5* | 0.36 | 4.58E-03 |
| *EFNB1* | *ABHD11-AS1* | -0.35 | 5.42E-03 |
| *EFNB1* | *ABHD6* | 0.31 | 1.51E-02 |
| *EFNB1* | *AC005944.2* | 0.35 | 5.50E-03 |
| *EFNB1* | *AC008074.1* | 0.33 | 7.86E-03 |
| *EFNB1* | *AC017002.2* | -0.33 | 7.96E-03 |
| *EFNB1* | *AC083843.2* | 0.41 | 1.08E-03 |
| *EFNB1* | *AC090498.1* | -0.36 | 3.83E-03 |
| *EFNB1* | *AC104088.1* | 0.35 | 4.94E-03 |
| *EFNB1* | *AC107081.5* | 0.33 | 8.56E-03 |
| *EFNB1* | *AC253572.1* | -0.33 | 8.37E-03 |
| *EFNB1* | *ACAA2* | -0.30 | 1.62E-02 |
| *EFNB1* | *ACKR3* | 0.42 | 7.20E-04 |
| *EFNB1* | *ACTL10* | -0.35 | 4.74E-03 |
| *EFNB1* | *ACVR1* | 0.33 | 8.49E-03 |
| *EFNB1* | *ADAM17* | 0.31 | 1.44E-02 |
| *EFNB1* | *ADGRL3* | 0.40 | 1.42E-03 |
| *EFNB1* | *ADI1* | -0.33 | 9.51E-03 |
| *EFNB1* | *ADIPOR2* | 0.33 | 8.17E-03 |
| *EFNB1* | *ADPGK-AS1* | 0.31 | 1.52E-02 |
| *EFNB1* | *ADRBK2* | 0.39 | 1.88E-03 |
| *EFNB1* | *AGBL2* | 0.39 | 1.68E-03 |
| *EFNB1* | *AHNAK2* | 0.31 | 1.36E-02 |
| *EFNB1* | *AIDA* | 0.33 | 7.98E-03 |
| *EFNB1* | *AL022476.2* | 0.34 | 6.57E-03 |
| *EFNB1* | *ALG10* | 0.32 | 1.12E-02 |
| *EFNB1* | *ALKBH4* | 0.31 | 1.53E-02 |
| *EFNB1* | *AMER1* | 0.42 | 7.39E-04 |
| *EFNB1* | *AMN1* | 0.39 | 1.91E-03 |
| *EFNB1* | *ANKRD42* | -0.41 | 9.86E-04 |
| *EFNB1* | *ANKS3* | 0.35 | 5.55E-03 |
| *EFNB1* | *ANO1* | 0.34 | 6.05E-03 |
| *EFNB1* | *ANO1-AS1* | 0.38 | 2.48E-03 |
| *EFNB1* | *ANO5* | -0.38 | 2.21E-03 |
| *EFNB1* | *ANO7* | -0.36 | 4.21E-03 |
| *EFNB1* | *ANXA3* | -0.37 | 2.99E-03 |
| *EFNB1* | *AOC2* | 0.31 | 1.51E-02 |
| *EFNB1* | *AP1S1* | 0.36 | 4.50E-03 |
| *EFNB1* | *AP2A1* | 0.33 | 9.08E-03 |
| *EFNB1* | *APBA1* | 0.33 | 9.39E-03 |
| *EFNB1* | *APBA3* | 0.31 | 1.40E-02 |
| *EFNB1* | *APC* | 0.31 | 1.29E-02 |
| *EFNB1* | *APMAP* | 0.33 | 9.52E-03 |
| *EFNB1* | *APOD* | -0.31 | 1.37E-02 |
| *EFNB1* | *ARAP3* | -0.30 | 1.70E-02 |
| *EFNB1* | *ARHGDIB* | -0.31 | 1.48E-02 |
| *EFNB1* | *ARHGEF39* | -0.31 | 1.41E-02 |
| *EFNB1* | *ARL4C* | 0.35 | 5.24E-03 |
| *EFNB1* | *ARMC7* | 0.39 | 1.99E-03 |
| *EFNB1* | *ARMCX5* | 0.31 | 1.57E-02 |
| *EFNB1* | *ARNTL* | 0.31 | 1.52E-02 |
| *EFNB1* | *ARSJ* | 0.37 | 2.81E-03 |
| *EFNB1* | *ASRGL1* | -0.32 | 1.15E-02 |
| *EFNB1* | *ATG2B* | 0.36 | 4.53E-03 |
| *EFNB1* | *ATP1B3* | 0.37 | 2.94E-03 |
| *EFNB1* | *ATP2B1* | 0.30 | 1.72E-02 |
| *EFNB1* | *ATP2C1* | 0.31 | 1.33E-02 |
| *EFNB1* | *ATP5G2* | -0.32 | 1.26E-02 |
| *EFNB1* | *ATPIF1* | -0.30 | 1.65E-02 |
| *EFNB1* | *ATRN* | 0.32 | 1.20E-02 |
| *EFNB1* | *ATXN1L* | 0.36 | 3.67E-03 |
| *EFNB1* | *ATXN3* | 0.31 | 1.43E-02 |
| *EFNB1* | *B3GALNT1* | -0.30 | 1.76E-02 |
| *EFNB1* | *B4GALNT1* | 0.34 | 6.20E-03 |
| *EFNB1* | *B4GALT7* | 0.30 | 1.67E-02 |
| *EFNB1* | *BAG5* | 0.35 | 5.05E-03 |
| *EFNB1* | *BAX* | 0.40 | 1.15E-03 |
| *EFNB1* | *BBOX1* | -0.31 | 1.36E-02 |
| *EFNB1* | *BCAS3* | 0.32 | 9.97E-03 |
| *EFNB1* | *BCKDHA* | 0.33 | 8.48E-03 |
| *EFNB1* | *BCL11B* | 0.44 | 2.94E-04 |
| *EFNB1* | *BDKRB1* | 0.36 | 3.81E-03 |
| *EFNB1* | *BDKRB2* | 0.34 | 6.71E-03 |
| *EFNB1* | *BEND7* | -0.40 | 1.25E-03 |
| *EFNB1* | *BICD1* | 0.32 | 1.11E-02 |
| *EFNB1* | *BICD2* | 0.34 | 7.61E-03 |
| *EFNB1* | *BRICD5* | 0.30 | 1.78E-02 |
| *EFNB1* | *BRWD3* | 0.32 | 1.03E-02 |
| *EFNB1* | *BSPRY* | -0.47 | 1.05E-04 |
| *EFNB1* | *BTG1* | 0.31 | 1.32E-02 |
| *EFNB1* | *C11orf70* | -0.31 | 1.33E-02 |
| *EFNB1* | *C12orf49* | -0.30 | 1.64E-02 |
| *EFNB1* | *C16orf70* | 0.32 | 1.12E-02 |
| *EFNB1* | *C16orf72* | 0.36 | 3.92E-03 |
| *EFNB1* | *C16orf91* | 0.39 | 1.52E-03 |
| *EFNB1* | *C19orf33* | -0.36 | 4.59E-03 |
| *EFNB1* | *C1orf210* | -0.32 | 1.16E-02 |
| *EFNB1* | *C1QTNF1* | 0.34 | 6.05E-03 |
| *EFNB1* | *C22orf31* | 0.32 | 1.01E-02 |
| *EFNB1* | *C5* | 0.30 | 1.71E-02 |
| *EFNB1* | *C5orf30* | -0.31 | 1.49E-02 |
| *EFNB1* | *C8orf37* | 0.34 | 7.43E-03 |
| *EFNB1* | *C9orf72* | 0.30 | 1.65E-02 |
| *EFNB1* | *CA12* | 0.39 | 1.54E-03 |
| *EFNB1* | *CA13* | -0.38 | 2.60E-03 |
| *EFNB1* | *CALM1* | 0.36 | 3.62E-03 |
| *EFNB1* | *CAMK1* | -0.30 | 1.70E-02 |
| *EFNB1* | *CAPS* | -0.31 | 1.42E-02 |
| *EFNB1* | *CAPZA1* | 0.35 | 5.05E-03 |
| *EFNB1* | *CAPZA2* | 0.35 | 5.00E-03 |
| *EFNB1* | *CAV2* | 0.32 | 1.19E-02 |
| *EFNB1* | *CCDC3* | 0.32 | 1.05E-02 |
| *EFNB1* | *CCDC85C* | 0.33 | 9.27E-03 |
| *EFNB1* | *CCDC90B* | -0.35 | 5.57E-03 |
| *EFNB1* | *CCNI2* | -0.33 | 8.46E-03 |
| *EFNB1* | *CCNK* | 0.33 | 8.03E-03 |
| *EFNB1* | *CD109* | 0.30 | 1.62E-02 |
| *EFNB1* | *CD44* | 0.38 | 2.03E-03 |
| *EFNB1* | *CD70* | -0.31 | 1.31E-02 |
| *EFNB1* | *CD9* | 0.36 | 4.44E-03 |
| *EFNB1* | *CDC42EP4* | 0.35 | 5.46E-03 |
| *EFNB1* | *CDH13* | 0.30 | 1.70E-02 |
| *EFNB1* | *CDH3* | 0.33 | 7.89E-03 |
| *EFNB1* | *CDHR1* | 0.36 | 3.60E-03 |
| *EFNB1* | *CDKN1B* | 0.30 | 1.72E-02 |
| *EFNB1* | *CELSR1* | 0.35 | 4.72E-03 |
| *EFNB1* | *CENPV* | -0.40 | 1.29E-03 |
| *EFNB1* | *CEP170B* | 0.34 | 7.41E-03 |
| *EFNB1* | *CEP295* | -0.44 | 3.61E-04 |
| *EFNB1* | *CEP57* | -0.37 | 3.48E-03 |
| *EFNB1* | *CEP68* | 0.31 | 1.39E-02 |
| *EFNB1* | *CHD9* | 0.30 | 1.61E-02 |
| *EFNB1* | *CHDH* | -0.30 | 1.73E-02 |
| *EFNB1* | *CHORDC1* | -0.30 | 1.76E-02 |
| *EFNB1* | *CLCN3* | 0.31 | 1.55E-02 |
| *EFNB1* | *CLDN7* | -0.35 | 5.13E-03 |
| *EFNB1* | *CLDND2* | 0.35 | 5.94E-03 |
| *EFNB1* | *CLEC16A* | 0.31 | 1.43E-02 |
| *EFNB1* | *CLEC2D* | 0.35 | 5.64E-03 |
| *EFNB1* | *CLK3* | -0.31 | 1.58E-02 |
| *EFNB1* | *CLSTN1* | 0.39 | 1.53E-03 |
| *EFNB1* | *CMTM7* | -0.38 | 2.19E-03 |
| *EFNB1* | *COG5* | 0.34 | 7.11E-03 |
| *EFNB1* | *COL12A1* | 0.40 | 1.10E-03 |
| *EFNB1* | *COL17A1* | 0.32 | 1.09E-02 |
| *EFNB1* | *COL18A1* | 0.32 | 1.03E-02 |
| *EFNB1* | *COL27A1* | 0.35 | 6.02E-03 |
| *EFNB1* | *COL4A5* | 0.39 | 1.81E-03 |
| *EFNB1* | *COL4A6* | 0.31 | 1.57E-02 |
| *EFNB1* | *COL7A1* | 0.40 | 1.28E-03 |
| *EFNB1* | *COX14* | -0.32 | 1.24E-02 |
| *EFNB1* | *COX17* | -0.31 | 1.44E-02 |
| *EFNB1* | *CPSF2* | 0.37 | 3.39E-03 |
| *EFNB1* | *CRACR2B* | -0.34 | 6.51E-03 |
| *EFNB1* | *CREB1* | 0.34 | 6.18E-03 |
| *EFNB1* | *CRYGS* | 0.30 | 1.65E-02 |
| *EFNB1* | *CSF3R* | -0.30 | 1.66E-02 |
| *EFNB1* | *CSK* | -0.32 | 1.09E-02 |
| *EFNB1* | *CSNK1G2* | 0.31 | 1.56E-02 |
| *EFNB1* | *CTA-276F8.1* | 0.31 | 1.54E-02 |
| *EFNB1* | *CTA-384D8.34* | 0.32 | 1.09E-02 |
| *EFNB1* | *CTB-193M12.5* | 0.35 | 5.18E-03 |
| *EFNB1* | *CTC-490E21.14* | 0.30 | 1.76E-02 |
| *EFNB1* | *CTD-2231E14.8* | 0.31 | 1.48E-02 |
| *EFNB1* | *CTD-2341M24.1* | 0.32 | 1.26E-02 |
| *EFNB1* | *CTD-2555O16.2* | 0.54 | 6.65E-06 |
| *EFNB1* | *CUL2* | 0.30 | 1.78E-02 |
| *EFNB1* | *CUX1* | 0.40 | 1.37E-03 |
| *EFNB1* | *CWC15* | -0.50 | 3.41E-05 |
| *EFNB1* | *CYP51A1* | 0.34 | 7.16E-03 |
| *EFNB1* | *DAB2IP* | 0.32 | 1.15E-02 |
| *EFNB1* | *DACT1* | 0.30 | 1.63E-02 |
| *EFNB1* | *DBNDD1* | -0.34 | 6.28E-03 |
| *EFNB1* | *DCK* | 0.33 | 9.23E-03 |
| *EFNB1* | *DDAH1* | -0.35 | 5.49E-03 |
| *EFNB1* | *DDB2* | 0.37 | 2.77E-03 |
| *EFNB1* | *DDR1* | 0.31 | 1.34E-02 |
| *EFNB1* | *DEPDC7* | 0.30 | 1.66E-02 |
| *EFNB1* | *DGKI* | 0.34 | 6.31E-03 |
| *EFNB1* | *DHX16* | -0.33 | 9.56E-03 |
| *EFNB1* | *DIAPH2-AS1* | 0.31 | 1.26E-02 |
| *EFNB1* | *DICER1* | 0.41 | 1.08E-03 |
| *EFNB1* | *DIP2B* | 0.42 | 7.12E-04 |
| *EFNB1* | *DKK3* | 0.30 | 1.68E-02 |
| *EFNB1* | *DLGAP1-AS1* | -0.31 | 1.42E-02 |
| *EFNB1* | *DNAH17* | 0.50 | 3.61E-05 |
| *EFNB1* | *DNAJA3* | 0.33 | 9.54E-03 |
| *EFNB1* | *DNAJB1* | 0.30 | 1.70E-02 |
| *EFNB1* | *DNAJC12* | -0.33 | 9.55E-03 |
| *EFNB1* | *DNALI1* | -0.32 | 1.24E-02 |
| *EFNB1* | *DPCD* | -0.43 | 4.50E-04 |
| *EFNB1* | *DRAM2* | 0.33 | 8.83E-03 |
| *EFNB1* | *DYX1C1* | -0.31 | 1.37E-02 |
| *EFNB1* | *ECHS1* | -0.35 | 5.35E-03 |
| *EFNB1* | *EIF2B4* | -0.36 | 4.35E-03 |
| *EFNB1* | *EIF2S3* | -0.31 | 1.40E-02 |
| *EFNB1* | *EIF4G2* | 0.32 | 9.99E-03 |
| *EFNB1* | *EIF5* | 0.37 | 3.14E-03 |
| *EFNB1* | *EIF5A2* | 0.31 | 1.32E-02 |
| *EFNB1* | *ELMO2* | 0.32 | 1.22E-02 |
| *EFNB1* | *ELMSAN1* | 0.31 | 1.42E-02 |
| *EFNB1* | *EMC7* | 0.36 | 3.65E-03 |
| *EFNB1* | *EMP2* | 0.45 | 2.62E-04 |
| *EFNB1* | *EPHB4* | 0.34 | 6.04E-03 |
| *EFNB1* | *ERCC4* | 0.31 | 1.56E-02 |
| *EFNB1* | *ERGIC2* | 0.45 | 2.67E-04 |
| *EFNB1* | *ERMAP* | 0.36 | 4.26E-03 |
| *EFNB1* | *ERVK3-1* | 0.31 | 1.42E-02 |
| *EFNB1* | *FAH* | -0.32 | 1.15E-02 |
| *EFNB1* | *FAM104A* | 0.35 | 5.71E-03 |
| *EFNB1* | *FAM149A* | -0.39 | 1.92E-03 |
| *EFNB1* | *FAM159A* | -0.33 | 9.43E-03 |
| *EFNB1* | *FAM160A1* | 0.34 | 6.16E-03 |
| *EFNB1* | *FAM160A2* | 0.40 | 1.31E-03 |
| *EFNB1* | *FAM3B* | -0.31 | 1.46E-02 |
| *EFNB1* | *FAM3C* | 0.31 | 1.33E-02 |
| *EFNB1* | *FASTKD2* | 0.30 | 1.64E-02 |
| *EFNB1* | *FAT2* | 0.34 | 6.12E-03 |
| *EFNB1* | *FBP1* | -0.37 | 2.84E-03 |
| *EFNB1* | *FBXL12* | 0.37 | 3.00E-03 |
| *EFNB1* | *FBXL16* | 0.31 | 1.45E-02 |
| *EFNB1* | *FBXO16* | -0.42 | 5.89E-04 |
| *EFNB1* | *FBXO8* | 0.32 | 1.20E-02 |
| *EFNB1* | *FCHO1* | -0.33 | 8.44E-03 |
| *EFNB1* | *FERMT1* | 0.33 | 8.08E-03 |
| *EFNB1* | *FGD6* | 0.38 | 2.11E-03 |
| *EFNB1* | *FGFR1OP* | -0.34 | 6.69E-03 |
| *EFNB1* | *FGFR3* | 0.37 | 2.88E-03 |
| *EFNB1* | *FLRT2* | 0.33 | 7.93E-03 |
| *EFNB1* | *FMNL2* | 0.31 | 1.50E-02 |
| *EFNB1* | *FMO5* | -0.40 | 1.28E-03 |
| *EFNB1* | *FN3K* | -0.30 | 1.59E-02 |
| *EFNB1* | *FNDC3B* | 0.32 | 1.18E-02 |
| *EFNB1* | *FNTB* | 0.37 | 2.91E-03 |
| *EFNB1* | *FOLR1* | -0.33 | 8.81E-03 |
| *EFNB1* | *FOXF2* | 0.35 | 5.42E-03 |
| *EFNB1* | *FOXJ2* | 0.33 | 8.88E-03 |
| *EFNB1* | *FOXN2* | 0.37 | 2.94E-03 |
| *EFNB1* | *FREM1* | 0.31 | 1.30E-02 |
| *EFNB1* | *FSCN1* | 0.31 | 1.37E-02 |
| *EFNB1* | *FST* | 0.41 | 8.98E-04 |
| *EFNB1* | *FTX* | 0.30 | 1.68E-02 |
| *EFNB1* | *FUCA2* | -0.33 | 8.60E-03 |
| *EFNB1* | *GAA* | 0.31 | 1.51E-02 |
| *EFNB1* | *GABRE* | 0.34 | 7.35E-03 |
| *EFNB1* | *GAL3ST4* | 0.36 | 4.62E-03 |
| *EFNB1* | *GALC* | 0.33 | 9.80E-03 |
| *EFNB1* | *GALE* | -0.40 | 1.13E-03 |
| *EFNB1* | *GALNT11* | 0.34 | 7.76E-03 |
| *EFNB1* | *GALNT18* | 0.42 | 5.88E-04 |
| *EFNB1* | *GAPVD1* | 0.31 | 1.39E-02 |
| *EFNB1* | *GCA* | -0.37 | 3.21E-03 |
| *EFNB1* | *GCK* | -0.33 | 9.14E-03 |
| *EFNB1* | *GCNT1* | -0.37 | 2.73E-03 |
| *EFNB1* | *GDF15* | -0.34 | 7.29E-03 |
| *EFNB1* | *GINS3* | 0.33 | 9.45E-03 |
| *EFNB1* | *GIPC2* | -0.45 | 2.44E-04 |
| *EFNB1* | *GJA3* | 0.37 | 2.74E-03 |
| *EFNB1* | *GJB2* | 0.41 | 9.32E-04 |
| *EFNB1* | *GJB5* | 0.36 | 4.32E-03 |
| *EFNB1* | *GJB6* | 0.36 | 4.17E-03 |
| *EFNB1* | *GLE1* | 0.31 | 1.40E-02 |
| *EFNB1* | *GM2A* | 0.50 | 3.05E-05 |
| *EFNB1* | *GMEB2* | 0.35 | 5.96E-03 |
| *EFNB1* | *GMPPB* | -0.37 | 2.95E-03 |
| *EFNB1* | *GNAI1* | 0.36 | 3.75E-03 |
| *EFNB1* | *GNL2* | -0.36 | 3.59E-03 |
| *EFNB1* | *GNRH1* | 0.32 | 1.17E-02 |
| *EFNB1* | *GOLGA5* | 0.32 | 1.08E-02 |
| *EFNB1* | *GOLGA7B* | 0.33 | 8.11E-03 |
| *EFNB1* | *GOLM1* | -0.46 | 1.65E-04 |
| *EFNB1* | *GOLPH3L* | -0.32 | 1.18E-02 |
| *EFNB1* | *GOLT1A* | -0.43 | 4.24E-04 |
| *EFNB1* | *GOLT1B* | 0.30 | 1.74E-02 |
| *EFNB1* | *GPC1* | 0.46 | 1.54E-04 |
| *EFNB1* | *GPR153* | 0.32 | 1.08E-02 |
| *EFNB1* | *GPR160* | -0.35 | 5.34E-03 |
| *EFNB1* | *GPS2* | -0.32 | 1.01E-02 |
| *EFNB1* | *GPSM3* | -0.36 | 3.88E-03 |
| *EFNB1* | *GRIP1* | 0.39 | 1.93E-03 |
| *EFNB1* | *GS1-393G12.14* | 0.32 | 1.24E-02 |
| *EFNB1* | *GSK3B* | 0.38 | 2.49E-03 |
| *EFNB1* | *GSKIP* | 0.36 | 4.25E-03 |
| *EFNB1* | *GTF2E1* | 0.34 | 7.02E-03 |
| *EFNB1* | *GTPBP10* | 0.33 | 9.75E-03 |
| *EFNB1* | *H2AFY2* | 0.32 | 1.02E-02 |
| *EFNB1* | *HAS3* | 0.43 | 4.89E-04 |
| *EFNB1* | *HBP1* | 0.38 | 2.42E-03 |
| *EFNB1* | *HERC3* | 0.34 | 6.47E-03 |
| *EFNB1* | *HIC1* | -0.31 | 1.45E-02 |
| *EFNB1* | *HIF1A* | 0.40 | 1.24E-03 |
| *EFNB1* | *HIP1* | 0.47 | 1.19E-04 |
| *EFNB1* | *HOMER2* | -0.33 | 9.36E-03 |
| *EFNB1* | *HOXD10* | 0.32 | 1.16E-02 |
| *EFNB1* | *HPN* | -0.34 | 6.28E-03 |
| *EFNB1* | *HRCT1* | -0.31 | 1.30E-02 |
| *EFNB1* | *HSP90AA1* | 0.39 | 1.70E-03 |
| *EFNB1* | *HSPH1* | 0.44 | 3.10E-04 |
| *EFNB1* | *ICAM3* | -0.33 | 9.58E-03 |
| *EFNB1* | *IFI27L2* | 0.33 | 8.64E-03 |
| *EFNB1* | *IFITM2* | -0.31 | 1.31E-02 |
| *EFNB1* | *IFRD1* | 0.35 | 5.02E-03 |
| *EFNB1* | *IGFL1* | 0.33 | 8.32E-03 |
| *EFNB1* | *IGSF3* | 0.38 | 2.32E-03 |
| *EFNB1* | *IL20RB* | 0.37 | 2.74E-03 |
| *EFNB1* | *IL32* | -0.32 | 1.25E-02 |
| *EFNB1* | *IMMP2L* | 0.38 | 2.39E-03 |
| *EFNB1* | *IMPDH2* | -0.33 | 9.14E-03 |
| *EFNB1* | *INHBA* | 0.30 | 1.70E-02 |
| *EFNB1* | *INPP5E* | 0.33 | 8.98E-03 |
| *EFNB1* | *INTS4* | -0.33 | 9.51E-03 |
| *EFNB1* | *IPO8* | 0.33 | 8.35E-03 |
| *EFNB1* | *IPPK* | 0.36 | 4.08E-03 |
| *EFNB1* | *IRX4* | 0.31 | 1.56E-02 |
| *EFNB1* | *ISLR2* | 0.43 | 4.58E-04 |
| *EFNB1* | *ITGA2* | 0.36 | 3.81E-03 |
| *EFNB1* | *ITGB3* | -0.33 | 8.55E-03 |
| *EFNB1* | *ITPK1* | 0.30 | 1.68E-02 |
| *EFNB1* | *JAG1* | 0.47 | 1.12E-04 |
| *EFNB1* | *JAG2* | 0.36 | 4.61E-03 |
| *EFNB1* | *JMJD7-PLA2G4B* | 0.34 | 7.17E-03 |
| *EFNB1* | *JTB* | -0.35 | 5.78E-03 |
| *EFNB1* | *KAT7* | 0.31 | 1.54E-02 |
| *EFNB1* | *KB-1460A1.5* | 0.31 | 1.40E-02 |
| *EFNB1* | *KCND3* | 0.34 | 7.32E-03 |
| *EFNB1* | *KCNE3* | -0.32 | 1.14E-02 |
| *EFNB1* | *KCNQ5* | 0.39 | 1.88E-03 |
| *EFNB1* | *KCTD15* | 0.35 | 5.94E-03 |
| *EFNB1* | *KCTD7* | 0.38 | 2.05E-03 |
| *EFNB1* | *KDM5A* | 0.30 | 1.67E-02 |
| *EFNB1* | *KIAA0930* | 0.40 | 1.30E-03 |
| *EFNB1* | *KIF16B* | 0.34 | 6.16E-03 |
| *EFNB1* | *KLF10* | 0.34 | 7.62E-03 |
| *EFNB1* | *KLF13* | 0.35 | 6.03E-03 |
| *EFNB1* | *KLF7* | 0.41 | 1.01E-03 |
| *EFNB1* | *KMT2E* | 0.33 | 8.75E-03 |
| *EFNB1* | *KMT2E-AS1* | 0.30 | 1.72E-02 |
| *EFNB1* | *KPNA1* | 0.36 | 4.44E-03 |
| *EFNB1* | *KPNA3* | 0.35 | 5.42E-03 |
| *EFNB1* | *KPTN* | 0.34 | 6.80E-03 |
| *EFNB1* | *KRT15* | 0.34 | 6.89E-03 |
| *EFNB1* | *KRT16* | 0.33 | 8.08E-03 |
| *EFNB1* | *KRT18* | -0.44 | 3.55E-04 |
| *EFNB1* | *KRT7* | -0.37 | 3.11E-03 |
| *EFNB1* | *KRT8* | -0.38 | 2.67E-03 |
| *EFNB1* | *KRTDAP* | 0.33 | 8.88E-03 |
| *EFNB1* | *LA16c-306E5.2* | 0.36 | 4.18E-03 |
| *EFNB1* | *LAMB3* | 0.33 | 9.91E-03 |
| *EFNB1* | *LAMP2* | 0.33 | 8.64E-03 |
| *EFNB1* | *LINC00519* | 0.32 | 1.21E-02 |
| *EFNB1* | *LINC00640* | 0.34 | 6.04E-03 |
| *EFNB1* | *LINC01004* | 0.36 | 3.65E-03 |
| *EFNB1* | *LINC01010* | 0.32 | 1.21E-02 |
| *EFNB1* | *LINC01094* | 0.34 | 7.18E-03 |
| *EFNB1* | *LINC01322* | 0.33 | 9.76E-03 |
| *EFNB1* | *LINC01376* | 0.30 | 1.72E-02 |
| *EFNB1* | *LINC01572* | 0.44 | 3.09E-04 |
| *EFNB1* | *LINC01605* | 0.42 | 6.54E-04 |
| *EFNB1* | *LIPA* | 0.31 | 1.36E-02 |
| *EFNB1* | *LMO1* | 0.30 | 1.63E-02 |
| *EFNB1* | *LNX2* | 0.31 | 1.48E-02 |
| *EFNB1* | *LPCAT3* | 0.42 | 7.28E-04 |
| *EFNB1* | *LRFN3* | 0.36 | 3.60E-03 |
| *EFNB1* | *LRP12* | 0.42 | 7.16E-04 |
| *EFNB1* | *LRRC29* | 0.30 | 1.61E-02 |
| *EFNB1* | *LRRC38* | 0.36 | 3.59E-03 |
| *EFNB1* | *LRRC58* | 0.34 | 6.08E-03 |
| *EFNB1* | *LRRC70* | 0.31 | 1.46E-02 |
| *EFNB1* | *LRRK1* | -0.32 | 1.10E-02 |
| *EFNB1* | *LRRTM2* | 0.34 | 7.19E-03 |
| *EFNB1* | *LSG1* | 0.32 | 1.11E-02 |
| *EFNB1* | *LSMEM1* | 0.31 | 1.34E-02 |
| *EFNB1* | *LTB4R* | 0.43 | 4.99E-04 |
| *EFNB1* | *LTB4R2* | 0.42 | 5.84E-04 |
| *EFNB1* | *LTBR* | 0.30 | 1.61E-02 |
| *EFNB1* | *LURAP1L* | -0.31 | 1.35E-02 |
| *EFNB1* | *LYL1* | -0.33 | 8.42E-03 |
| *EFNB1* | *LYZ* | -0.44 | 3.88E-04 |
| *EFNB1* | *M6PR* | 0.46 | 1.74E-04 |
| *EFNB1* | *MAN1B1-AS1* | 0.41 | 9.77E-04 |
| *EFNB1* | *MAP7* | -0.34 | 6.75E-03 |
| *EFNB1* | *MAPKBP1* | 0.46 | 1.55E-04 |
| *EFNB1* | *1-Mar* | -0.43 | 5.42E-04 |
| *EFNB1* | *MARK3* | 0.31 | 1.29E-02 |
| *EFNB1* | *MAST1* | -0.30 | 1.71E-02 |
| *EFNB1* | *MBD1* | 0.31 | 1.31E-02 |
| *EFNB1* | *MDGA1* | 0.35 | 5.33E-03 |
| *EFNB1* | *MEX3C* | 0.40 | 1.23E-03 |
| *EFNB1* | *MFHAS1* | 0.46 | 1.43E-04 |
| *EFNB1* | *MIER3* | 0.40 | 1.34E-03 |
| *EFNB1* | *MN1* | 0.32 | 1.03E-02 |
| *EFNB1* | *MPC2* | -0.31 | 1.55E-02 |
| *EFNB1* | *MRE11A* | -0.39 | 1.90E-03 |
| *EFNB1* | *MRPL53* | -0.30 | 1.78E-02 |
| *EFNB1* | *MSN* | 0.31 | 1.53E-02 |
| *EFNB1* | *MTERF1* | 0.35 | 5.71E-03 |
| *EFNB1* | *MTSS1* | 0.31 | 1.55E-02 |
| *EFNB1* | *MYL6* | -0.36 | 4.45E-03 |
| *EFNB1* | *MYO5B* | -0.32 | 1.24E-02 |
| *EFNB1* | *MYO5C* | -0.39 | 1.85E-03 |
| *EFNB1* | *MZF1-AS1* | 0.31 | 1.51E-02 |
| *EFNB1* | *N4BP1* | 0.37 | 2.79E-03 |
| *EFNB1* | *NAA20* | 0.31 | 1.59E-02 |
| *EFNB1* | *NACA* | -0.30 | 1.68E-02 |
| *EFNB1* | *NALT1* | 0.34 | 6.45E-03 |
| *EFNB1* | *NAPB* | 0.36 | 3.64E-03 |
| *EFNB1* | *NAV3* | 0.31 | 1.29E-02 |
| *EFNB1* | *NDE1* | 0.43 | 4.46E-04 |
| *EFNB1* | *NDRG4* | 0.39 | 1.74E-03 |
| *EFNB1* | *NDUFAF3* | -0.31 | 1.56E-02 |
| *EFNB1* | *NDUFS5* | -0.31 | 1.50E-02 |
| *EFNB1* | *NEK3* | -0.37 | 2.83E-03 |
| *EFNB1* | *NFATC4* | -0.32 | 1.24E-02 |
| *EFNB1* | *NFE2L1* | 0.34 | 6.82E-03 |
| *EFNB1* | *NFE2L2* | 0.35 | 4.90E-03 |
| *EFNB1* | *NGEF* | 0.37 | 2.85E-03 |
| *EFNB1* | *NIT1* | -0.35 | 4.89E-03 |
| *EFNB1* | *NME6* | -0.33 | 8.86E-03 |
| *EFNB1* | *NONO* | 0.33 | 8.17E-03 |
| *EFNB1* | *NOSTRIN* | -0.30 | 1.65E-02 |
| *EFNB1* | *NR1D1* | 0.31 | 1.49E-02 |
| *EFNB1* | *NR2C1* | -0.32 | 1.22E-02 |
| *EFNB1* | *NSMCE4A* | -0.31 | 1.34E-02 |
| *EFNB1* | *NT5C* | 0.38 | 2.18E-03 |
| *EFNB1* | *NTAN1* | 0.35 | 5.65E-03 |
| *EFNB1* | *NUP50-AS1* | 0.32 | 1.23E-02 |
| *EFNB1* | *NUP58* | 0.32 | 1.14E-02 |
| *EFNB1* | *NUPR2* | -0.38 | 2.57E-03 |
| *EFNB1* | *ODC1* | 0.30 | 1.71E-02 |
| *EFNB1* | *OGT* | 0.34 | 7.22E-03 |
| *EFNB1* | *OLFML2B* | 0.34 | 7.53E-03 |
| *EFNB1* | *ORC5* | 0.37 | 2.89E-03 |
| *EFNB1* | *PALB2* | 0.36 | 3.60E-03 |
| *EFNB1* | *PANX2* | 0.38 | 2.19E-03 |
| *EFNB1* | *PAPOLA* | 0.37 | 2.80E-03 |
| *EFNB1* | *PAQR4* | 0.38 | 2.39E-03 |
| *EFNB1* | *PARD6A* | -0.49 | 4.41E-05 |
| *EFNB1* | *PARVG* | -0.32 | 1.11E-02 |
| *EFNB1* | *PAX8* | 0.33 | 7.96E-03 |
| *EFNB1* | *PCBD1* | -0.30 | 1.70E-02 |
| *EFNB1* | *PCDH17* | 0.36 | 3.76E-03 |
| *EFNB1* | *PCDHB15* | 0.44 | 3.82E-04 |
| *EFNB1* | *PCDHB16* | 0.33 | 9.88E-03 |
| *EFNB1* | *PCDHB7* | 0.30 | 1.72E-02 |
| *EFNB1* | *PCDHGA8* | 0.36 | 3.59E-03 |
| *EFNB1* | *PCDHGB2* | 0.34 | 6.10E-03 |
| *EFNB1* | *PCIF1* | 0.33 | 7.91E-03 |
| *EFNB1* | *PCM1* | -0.37 | 2.97E-03 |
| *EFNB1* | *PCYOX1L* | 0.35 | 5.50E-03 |
| *EFNB1* | *PCYT1A* | 0.36 | 3.54E-03 |
| *EFNB1* | *PDCD4* | -0.31 | 1.39E-02 |
| *EFNB1* | *PDE4D* | -0.34 | 6.70E-03 |
| *EFNB1* | *PDE7A* | 0.34 | 6.93E-03 |
| *EFNB1* | *PECR* | -0.44 | 4.05E-04 |
| *EFNB1* | *PEX13* | 0.34 | 6.07E-03 |
| *EFNB1* | *PGBD2* | 0.30 | 1.67E-02 |
| *EFNB1* | *PGM2L1* | -0.35 | 5.72E-03 |
| *EFNB1* | *PGRMC1* | 0.33 | 9.72E-03 |
| *EFNB1* | *PHF21A* | 0.31 | 1.53E-02 |
| *EFNB1* | *PHYHD1* | -0.33 | 8.40E-03 |
| *EFNB1* | *PI15* | 0.30 | 1.77E-02 |
| *EFNB1* | *PIFO* | -0.31 | 1.46E-02 |
| *EFNB1* | *PIGK* | 0.35 | 5.89E-03 |
| *EFNB1* | *PIM3* | 0.31 | 1.31E-02 |
| *EFNB1* | *PJA1* | 0.51 | 2.34E-05 |
| *EFNB1* | *PKN1* | -0.35 | 5.48E-03 |
| *EFNB1* | *PKN3* | -0.39 | 1.85E-03 |
| *EFNB1* | *PLA2R1* | 0.38 | 2.12E-03 |
| *EFNB1* | *PLEKHA6* | -0.35 | 5.96E-03 |
| *EFNB1* | *PLEKHG3* | 0.40 | 1.30E-03 |
| *EFNB1* | *PLOD3* | 0.37 | 2.99E-03 |
| *EFNB1* | *PNPLA8* | 0.32 | 1.20E-02 |
| *EFNB1* | *POLL* | -0.30 | 1.73E-02 |
| *EFNB1* | *POLR3C* | -0.40 | 1.27E-03 |
| *EFNB1* | *PORCN* | 0.31 | 1.35E-02 |
| *EFNB1* | *PPA1* | -0.32 | 1.19E-02 |
| *EFNB1* | *PPAT* | 0.33 | 8.24E-03 |
| *EFNB1* | *PPIL6* | -0.45 | 2.05E-04 |
| *EFNB1* | *PPM1H* | -0.39 | 1.71E-03 |
| *EFNB1* | *PPM1J* | 0.34 | 6.64E-03 |
| *EFNB1* | *PPP1R21* | 0.32 | 1.24E-02 |
| *EFNB1* | *PRCD* | -0.33 | 9.49E-03 |
| *EFNB1* | *PRICKLE1* | 0.38 | 2.04E-03 |
| *EFNB1* | *PRKD2* | 0.31 | 1.32E-02 |
| *EFNB1* | *PRKRIP1* | 0.31 | 1.50E-02 |
| *EFNB1* | *PRNCR1* | 0.33 | 8.54E-03 |
| *EFNB1* | *PRNP* | 0.42 | 6.72E-04 |
| *EFNB1* | *PRR13* | -0.34 | 6.92E-03 |
| *EFNB1* | *PRSS16* | -0.43 | 4.64E-04 |
| *EFNB1* | *PTCD1* | 0.40 | 1.43E-03 |
| *EFNB1* | *PTGFRN* | 0.39 | 1.59E-03 |
| *EFNB1* | *PTH2R* | 0.35 | 5.79E-03 |
| *EFNB1* | *PTPRCAP* | -0.32 | 1.23E-02 |
| *EFNB1* | *PTPRZ1* | 0.39 | 1.88E-03 |
| *EFNB1* | *PVRL1* | 0.37 | 3.17E-03 |
| *EFNB1* | *PVT1* | 0.33 | 7.84E-03 |
| *EFNB1* | *PYCARD-AS1* | 0.32 | 1.09E-02 |
| *EFNB1* | *PYGB* | 0.36 | 3.78E-03 |
| *EFNB1* | *QPRT* | -0.41 | 9.65E-04 |
| *EFNB1* | *QSER1* | 0.43 | 5.29E-04 |
| *EFNB1* | *R3HDM4* | 0.31 | 1.38E-02 |
| *EFNB1* | *RAB30-AS1* | -0.40 | 1.19E-03 |
| *EFNB1* | *RAB36* | -0.32 | 1.02E-02 |
| *EFNB1* | *RAB42* | -0.32 | 1.09E-02 |
| *EFNB1* | *RABGEF1* | 0.32 | 1.07E-02 |
| *EFNB1* | *RAD51B* | 0.35 | 4.79E-03 |
| *EFNB1* | *RAMP1* | -0.33 | 8.48E-03 |
| *EFNB1* | *RAP2C-AS1* | 0.31 | 1.32E-02 |
| *EFNB1* | *RARG* | 0.38 | 2.26E-03 |
| *EFNB1* | *RASAL2-AS1* | -0.31 | 1.34E-02 |
| *EFNB1* | *RBM5-AS1* | 0.31 | 1.32E-02 |
| *EFNB1* | *RBP7* | -0.33 | 9.56E-03 |
| *EFNB1* | *RENBP* | -0.30 | 1.78E-02 |
| *EFNB1* | *REST* | 0.35 | 4.95E-03 |
| *EFNB1* | *RFWD3* | 0.41 | 1.03E-03 |
| *EFNB1* | *RGL3* | -0.32 | 1.07E-02 |
| *EFNB1* | *RHBDD2* | 0.37 | 3.26E-03 |
| *EFNB1* | *RHBDF2* | 0.33 | 9.07E-03 |
| *EFNB1* | *RHPN2* | -0.31 | 1.33E-02 |
| *EFNB1* | *RINT1* | 0.35 | 5.67E-03 |
| *EFNB1* | *RNF125* | -0.33 | 8.82E-03 |
| *EFNB1* | *RNF168* | 0.36 | 4.19E-03 |
| *EFNB1* | *RNF217-AS1* | 0.38 | 2.09E-03 |
| *EFNB1* | *RNPS1* | 0.37 | 2.88E-03 |
| *EFNB1* | *ROGDI* | 0.37 | 3.08E-03 |
| *EFNB1* | *RP11-1000B6.8* | 0.35 | 5.46E-03 |
| *EFNB1* | *RP11-1246C19.1* | 0.33 | 8.81E-03 |
| *EFNB1* | *RP11-128M1.1* | 0.31 | 1.49E-02 |
| *EFNB1* | *RP11-15B17.1* | 0.33 | 9.16E-03 |
| *EFNB1* | *RP11-174G6.1* | 0.36 | 4.12E-03 |
| *EFNB1* | *RP11-191L17.1* | 0.36 | 3.95E-03 |
| *EFNB1* | *RP11-191L9.4* | 0.34 | 7.63E-03 |
| *EFNB1* | *RP11-212I21.4* | 0.31 | 1.32E-02 |
| *EFNB1* | *RP11-225B17.2* | 0.30 | 1.76E-02 |
| *EFNB1* | *RP11-231D20.2* | 0.31 | 1.51E-02 |
| *EFNB1* | *RP11-279O9.4* | -0.36 | 4.44E-03 |
| *EFNB1* | *RP11-27M24.1* | 0.41 | 1.02E-03 |
| *EFNB1* | *RP11-283G6.3* | 0.35 | 4.98E-03 |
| *EFNB1* | *RP11-2B6.2* | 0.30 | 1.62E-02 |
| *EFNB1* | *RP11-307N16.6* | 0.32 | 1.22E-02 |
| *EFNB1* | *RP11-327F22.4* | 0.46 | 1.61E-04 |
| *EFNB1* | *RP11-328C8.4* | 0.39 | 1.75E-03 |
| *EFNB1* | *RP11-334J6.7* | 0.40 | 1.16E-03 |
| *EFNB1* | *RP11-35G9.5* | 0.41 | 9.83E-04 |
| *EFNB1* | *RP11-362K14.5* | 0.32 | 1.12E-02 |
| *EFNB1* | *RP11-379K17.12* | 0.38 | 2.67E-03 |
| *EFNB1* | *RP11-397A16.1* | 0.37 | 3.14E-03 |
| *EFNB1* | *RP11-42I10.1* | 0.36 | 3.99E-03 |
| *EFNB1* | *RP11-434D9.2* | 0.39 | 1.97E-03 |
| *EFNB1* | *RP11-44N21.1* | 0.35 | 5.34E-03 |
| *EFNB1* | *RP11-455F5.3* | 0.33 | 9.32E-03 |
| *EFNB1* | *RP11-473O4.4* | 0.30 | 1.67E-02 |
| *EFNB1* | *RP11-486L19.2* | 0.32 | 1.08E-02 |
| *EFNB1* | *RP11-497E19.1* | 0.34 | 6.66E-03 |
| *EFNB1* | *RP11-497E19.2* | 0.33 | 8.86E-03 |
| *EFNB1* | *RP11-498C9.2* | 0.31 | 1.40E-02 |
| *EFNB1* | *RP11-498C9.3* | 0.31 | 1.50E-02 |
| *EFNB1* | *RP11-49I11.1* | -0.31 | 1.29E-02 |
| *EFNB1* | *RP11-552F3.9* | 0.38 | 2.07E-03 |
| *EFNB1* | *RP11-575L7.8* | 0.41 | 9.15E-04 |
| *EFNB1* | *RP11-57H14.2* | 0.32 | 1.16E-02 |
| *EFNB1* | *RP11-5C23.1* | 0.30 | 1.77E-02 |
| *EFNB1* | *RP11-600F24.7* | 0.39 | 1.59E-03 |
| *EFNB1* | *RP11-615I2.2* | 0.31 | 1.49E-02 |
| *EFNB1* | *RP11-649A18.12* | 0.32 | 1.07E-02 |
| *EFNB1* | *RP11-65I12.1* | 0.34 | 6.97E-03 |
| *EFNB1* | *RP11-666A8.8* | 0.34 | 7.39E-03 |
| *EFNB1* | *RP11-692D12.1* | 0.37 | 3.42E-03 |
| *EFNB1* | *RP11-729L2.2* | 0.39 | 1.78E-03 |
| *EFNB1* | *RP11-776H12.1* | 0.34 | 7.51E-03 |
| *EFNB1* | *RP11-780K2.1* | 0.33 | 7.96E-03 |
| *EFNB1* | *RP11-894P9.1* | 0.38 | 2.12E-03 |
| *EFNB1* | *RP11-902B17.1* | 0.33 | 8.03E-03 |
| *EFNB1* | *RP11-93H24.3* | 0.30 | 1.64E-02 |
| *EFNB1* | *RP11-973N13.4* | 0.38 | 2.62E-03 |
| *EFNB1* | *RP11-98D18.9* | -0.31 | 1.34E-02 |
| *EFNB1* | *RP13-766D20.4* | 0.38 | 2.22E-03 |
| *EFNB1* | *RP4-734G22.3* | 0.32 | 1.18E-02 |
| *EFNB1* | *RP5-884C9.2* | 0.32 | 1.12E-02 |
| *EFNB1* | *RP6-114E22.1* | 0.31 | 1.42E-02 |
| *EFNB1* | *RPL26* | -0.34 | 6.62E-03 |
| *EFNB1* | *RPL31* | -0.31 | 1.27E-02 |
| *EFNB1* | *RPL32* | -0.39 | 1.77E-03 |
| *EFNB1* | *RPL34* | -0.31 | 1.32E-02 |
| *EFNB1* | *RPL37* | -0.31 | 1.40E-02 |
| *EFNB1* | *RPL41* | -0.32 | 1.23E-02 |
| *EFNB1* | *RPL6* | -0.33 | 8.82E-03 |
| *EFNB1* | *RPS12* | -0.41 | 8.90E-04 |
| *EFNB1* | *RPS24* | -0.39 | 1.74E-03 |
| *EFNB1* | *RPS27* | -0.32 | 1.17E-02 |
| *EFNB1* | *RPS27L* | -0.37 | 3.41E-03 |
| *EFNB1* | *RPS3* | -0.36 | 3.98E-03 |
| *EFNB1* | *RPS6KC1* | 0.31 | 1.40E-02 |
| *EFNB1* | *RRAD* | -0.31 | 1.55E-02 |
| *EFNB1* | *RSPRY1* | 0.32 | 1.07E-02 |
| *EFNB1* | *RTN4* | 0.43 | 5.32E-04 |
| *EFNB1* | *S100A13* | -0.40 | 1.33E-03 |
| *EFNB1* | *S100A6* | -0.39 | 1.90E-03 |
| *EFNB1* | *SAMD8* | 0.37 | 3.51E-03 |
| *EFNB1* | *SCIN* | -0.47 | 1.03E-04 |
| *EFNB1* | *SCN2A* | 0.34 | 6.54E-03 |
| *EFNB1* | *SCRN3* | -0.31 | 1.42E-02 |
| *EFNB1* | *SDC1* | 0.39 | 1.76E-03 |
| *EFNB1* | *SDK2* | 0.34 | 6.38E-03 |
| *EFNB1* | *SDSL* | -0.31 | 1.29E-02 |
| *EFNB1* | *SEL1L* | 0.43 | 5.12E-04 |
| *EFNB1* | *SELT* | 0.30 | 1.74E-02 |
| *EFNB1* | *SENP5* | 0.35 | 4.82E-03 |
| *EFNB1* | *SEPW1* | 0.36 | 4.39E-03 |
| *EFNB1* | *SERINC3* | 0.33 | 8.31E-03 |
| *EFNB1* | *SGK223* | 0.31 | 1.36E-02 |
| *EFNB1* | *SH2D4A* | -0.32 | 1.03E-02 |
| *EFNB1* | *SH3BGRL2* | -0.33 | 7.93E-03 |
| *EFNB1* | *SH3RF3* | 0.32 | 1.02E-02 |
| *EFNB1* | *SHKBP1* | 0.31 | 1.52E-02 |
| *EFNB1* | *SHTN1* | -0.34 | 7.09E-03 |
| *EFNB1* | *SIGIRR* | -0.38 | 2.33E-03 |
| *EFNB1* | *SIRPA* | 0.34 | 6.68E-03 |
| *EFNB1* | *SIRT6* | 0.35 | 4.78E-03 |
| *EFNB1* | *SLAIN1* | -0.37 | 2.70E-03 |
| *EFNB1* | *SLC16A1* | 0.37 | 3.52E-03 |
| *EFNB1* | *SLC1A4* | 0.48 | 7.97E-05 |
| *EFNB1* | *SLC22A18* | 0.30 | 1.63E-02 |
| *EFNB1* | *SLC30A6* | 0.33 | 8.30E-03 |
| *EFNB1* | *SLC36A4* | -0.45 | 2.70E-04 |
| *EFNB1* | *SLC37A3* | 0.31 | 1.52E-02 |
| *EFNB1* | *SLC38A6* | 0.35 | 5.33E-03 |
| *EFNB1* | *SLC39A11* | 0.37 | 3.43E-03 |
| *EFNB1* | *SLC39A9* | 0.33 | 9.43E-03 |
| *EFNB1* | *SLC3A2* | 0.34 | 7.76E-03 |
| *EFNB1* | *SLC40A1* | -0.31 | 1.50E-02 |
| *EFNB1* | *SLC44A1* | 0.35 | 5.15E-03 |
| *EFNB1* | *SLC44A3* | -0.48 | 8.90E-05 |
| *EFNB1* | *SLC46A1* | -0.43 | 4.59E-04 |
| *EFNB1* | *SLC47A2* | 0.36 | 4.27E-03 |
| *EFNB1* | *SLC5A12* | 0.36 | 4.30E-03 |
| *EFNB1* | *SLC7A4* | -0.35 | 6.03E-03 |
| *EFNB1* | *SLC7A8* | 0.30 | 1.68E-02 |
| *EFNB1* | *SLC9A3R1* | 0.31 | 1.40E-02 |
| *EFNB1* | *SLC9A6* | 0.41 | 1.05E-03 |
| *EFNB1* | *SLC9A9* | 0.42 | 7.08E-04 |
| *EFNB1* | *SMC6* | 0.34 | 6.55E-03 |
| *EFNB1* | *SMCO4* | -0.32 | 1.16E-02 |
| *EFNB1* | *SMIM24* | -0.32 | 1.15E-02 |
| *EFNB1* | *SMPDL3B* | -0.58 | 8.00E-07 |
| *EFNB1* | *SNAI2* | 0.31 | 1.39E-02 |
| *EFNB1* | *SNAPC1* | 0.34 | 6.49E-03 |
| *EFNB1* | *SNX11* | 0.31 | 1.52E-02 |
| *EFNB1* | *SOCS1* | 0.50 | 2.85E-05 |
| *EFNB1* | *SORBS2* | -0.32 | 1.26E-02 |
| *EFNB1* | *SOX12* | 0.31 | 1.57E-02 |
| *EFNB1* | *SPDYE16* | 0.33 | 8.92E-03 |
| *EFNB1* | *SPDYE2B* | 0.31 | 1.37E-02 |
| *EFNB1* | *SPN* | -0.36 | 4.22E-03 |
| *EFNB1* | *SPRY4-IT1* | 0.31 | 1.45E-02 |
| *EFNB1* | *SPTLC2* | 0.32 | 1.01E-02 |
| *EFNB1* | *SRD5A1* | 0.32 | 1.21E-02 |
| *EFNB1* | *SRGAP2C* | 0.35 | 4.81E-03 |
| *EFNB1* | *SRPK3* | -0.30 | 1.64E-02 |
| *EFNB1* | *SRRM3* | 0.32 | 1.02E-02 |
| *EFNB1* | *ST3GAL1* | 0.38 | 2.01E-03 |
| *EFNB1* | *ST3GAL5* | 0.31 | 1.33E-02 |
| *EFNB1* | *ST6GALNAC2* | 0.35 | 5.69E-03 |
| *EFNB1* | *ST7* | 0.40 | 1.14E-03 |
| *EFNB1* | *ST7-AS1* | 0.32 | 1.09E-02 |
| *EFNB1* | *STRIP2* | 0.32 | 1.06E-02 |
| *EFNB1* | *STX16* | 0.33 | 9.75E-03 |
| *EFNB1* | *STX7* | -0.36 | 3.66E-03 |
| *EFNB1* | *SULF2* | 0.30 | 1.74E-02 |
| *EFNB1* | *SVIP* | -0.36 | 4.13E-03 |
| *EFNB1* | *SYNE4* | -0.46 | 1.75E-04 |
| *EFNB1* | *SYNJ2BP* | 0.30 | 1.76E-02 |
| *EFNB1* | *SYPL1* | 0.45 | 2.58E-04 |
| *EFNB1* | *SYT14* | 0.30 | 1.74E-02 |
| *EFNB1* | *SYTL2* | -0.35 | 4.77E-03 |
| *EFNB1* | *TAF1D* | -0.41 | 8.08E-04 |
| *EFNB1* | *TANGO6* | 0.30 | 1.69E-02 |
| *EFNB1* | *TARSL2* | -0.42 | 7.85E-04 |
| *EFNB1* | *TBC1D10B* | 0.33 | 8.04E-03 |
| *EFNB1* | *TCEA3* | -0.36 | 3.71E-03 |
| *EFNB1* | *TCF20* | 0.38 | 2.44E-03 |
| *EFNB1* | *TCFL5* | 0.31 | 1.40E-02 |
| *EFNB1* | *TCN2* | -0.39 | 1.61E-03 |
| *EFNB1* | *TCTEX1D2* | 0.35 | 5.24E-03 |
| *EFNB1* | *TDRKH* | -0.46 | 1.73E-04 |
| *EFNB1* | *TEAD2* | 0.33 | 7.97E-03 |
| *EFNB1* | *TECR* | 0.42 | 6.93E-04 |
| *EFNB1* | *TEF* | 0.36 | 4.48E-03 |
| *EFNB1* | *TEX41* | 0.32 | 1.15E-02 |
| *EFNB1* | *TEX9* | -0.35 | 4.91E-03 |
| *EFNB1* | *THRA* | 0.33 | 9.90E-03 |
| *EFNB1* | *TINCR* | 0.35 | 4.67E-03 |
| *EFNB1* | *TMEM104* | 0.37 | 2.76E-03 |
| *EFNB1* | *TMEM109* | 0.42 | 6.49E-04 |
| *EFNB1* | *TMEM125* | -0.38 | 2.40E-03 |
| *EFNB1* | *TMEM126A* | -0.33 | 9.33E-03 |
| *EFNB1* | *TMEM126B* | -0.41 | 9.15E-04 |
| *EFNB1* | *TMEM132A* | 0.42 | 6.12E-04 |
| *EFNB1* | *TMEM154* | 0.33 | 8.27E-03 |
| *EFNB1* | *TMEM161A* | 0.39 | 1.55E-03 |
| *EFNB1* | *TMEM165* | 0.31 | 1.57E-02 |
| *EFNB1* | *TMEM168* | 0.33 | 9.84E-03 |
| *EFNB1* | *TMEM184B* | 0.38 | 2.45E-03 |
| *EFNB1* | *TMEM186* | 0.38 | 2.18E-03 |
| *EFNB1* | *TMEM189* | 0.37 | 3.26E-03 |
| *EFNB1* | *TMEM208* | 0.30 | 1.62E-02 |
| *EFNB1* | *TMEM220* | -0.39 | 1.74E-03 |
| *EFNB1* | *TMEM256* | -0.35 | 5.29E-03 |
| *EFNB1* | *TMEM33* | 0.36 | 4.56E-03 |
| *EFNB1* | *TMEM38B* | 0.30 | 1.67E-02 |
| *EFNB1* | *TMEM63C* | 0.34 | 7.37E-03 |
| *EFNB1* | *TMX1* | 0.38 | 2.49E-03 |
| *EFNB1* | *TNC* | 0.44 | 3.78E-04 |
| *EFNB1* | *TNFRSF1A* | 0.45 | 2.59E-04 |
| *EFNB1* | *TNFSF13* | -0.44 | 3.92E-04 |
| *EFNB1* | *TOPORS-AS1* | -0.39 | 1.52E-03 |
| *EFNB1* | *TOX2* | 0.40 | 1.14E-03 |
| *EFNB1* | *TP53AIP1* | 0.33 | 8.93E-03 |
| *EFNB1* | *TP53I3* | -0.33 | 9.20E-03 |
| *EFNB1* | *TP63* | 0.45 | 2.88E-04 |
| *EFNB1* | *TP73* | 0.30 | 1.78E-02 |
| *EFNB1* | *TPBG* | 0.39 | 1.96E-03 |
| *EFNB1* | *TPST1* | 0.35 | 5.32E-03 |
| *EFNB1* | *TRABD2B* | 0.34 | 6.46E-03 |
| *EFNB1* | *TRAF3* | 0.53 | 1.17E-05 |
| *EFNB1* | *TRAF7* | 0.39 | 1.51E-03 |
| *EFNB1* | *TRIP11* | 0.34 | 6.18E-03 |
| *EFNB1* | *TSEN2* | -0.39 | 1.97E-03 |
| *EFNB1* | *TSLP* | 0.32 | 1.02E-02 |
| *EFNB1* | *TSPAN1* | -0.46 | 1.92E-04 |
| *EFNB1* | *TSPAN14* | 0.33 | 9.46E-03 |
| *EFNB1* | *TSPAN9* | 0.30 | 1.73E-02 |
| *EFNB1* | *TTC39A* | -0.31 | 1.42E-02 |
| *EFNB1* | *TTLL12* | 0.35 | 5.73E-03 |
| *EFNB1* | *TTPAL* | 0.30 | 1.63E-02 |
| *EFNB1* | *TYW1* | 0.33 | 8.04E-03 |
| *EFNB1* | *UAP1L1* | 0.33 | 8.15E-03 |
| *EFNB1* | *UBA6* | 0.38 | 2.22E-03 |
| *EFNB1* | *UBE2Q2L* | 0.38 | 2.42E-03 |
| *EFNB1* | *UBE2QL1* | 0.37 | 3.41E-03 |
| *EFNB1* | *UBR5* | 0.30 | 1.72E-02 |
| *EFNB1* | *UBR5-AS1* | -0.36 | 3.77E-03 |
| *EFNB1* | *UBXN10* | -0.33 | 9.17E-03 |
| *EFNB1* | *UBXN7* | 0.34 | 7.45E-03 |
| *EFNB1* | *UCP2* | -0.34 | 6.93E-03 |
| *EFNB1* | *UGT1A7* | 0.31 | 1.47E-02 |
| *EFNB1* | *UQCRQ* | -0.31 | 1.35E-02 |
| *EFNB1* | *URGCP-MRPS24* | 0.30 | 1.63E-02 |
| *EFNB1* | *USMG5* | -0.36 | 4.31E-03 |
| *EFNB1* | *USP42* | 0.32 | 1.01E-02 |
| *EFNB1* | *USP5* | 0.31 | 1.32E-02 |
| *EFNB1* | *USP7* | 0.35 | 5.58E-03 |
| *EFNB1* | *VKORC1L1* | 0.32 | 1.04E-02 |
| *EFNB1* | *VWA1* | -0.31 | 1.30E-02 |
| *EFNB1* | *WDFY2* | 0.45 | 2.47E-04 |
| *EFNB1* | *WDR44* | 0.43 | 4.12E-04 |
| *EFNB1* | *WDR47* | 0.35 | 5.15E-03 |
| *EFNB1* | *WDR91* | 0.32 | 1.20E-02 |
| *EFNB1* | *WNT11* | -0.30 | 1.69E-02 |
| *EFNB1* | *WNT7B* | 0.51 | 2.83E-05 |
| *EFNB1* | *WNT9A* | 0.31 | 1.34E-02 |
| *EFNB1* | *XK* | -0.43 | 5.22E-04 |
| *EFNB1* | *XRN2* | 0.31 | 1.31E-02 |
| *EFNB1* | *XXbac-BPG181M17.6* | 0.36 | 3.93E-03 |
| *EFNB1* | *XXbac-BPG32J3.22* | -0.34 | 6.32E-03 |
| *EFNB1* | *XYLT2* | 0.34 | 7.60E-03 |
| *EFNB1* | *YAP1* | -0.30 | 1.63E-02 |
| *EFNB1* | *YWHAG* | 0.33 | 8.57E-03 |
| *EFNB1* | *YY1* | 0.38 | 2.20E-03 |
| *EFNB1* | *ZBTB25* | 0.35 | 4.66E-03 |
| *EFNB1* | *ZC2HC1C* | -0.32 | 1.13E-02 |
| *EFNB1* | *ZFP90* | 0.35 | 5.02E-03 |
| *EFNB1* | *ZFYVE1* | 0.32 | 1.21E-02 |
| *EFNB1* | *ZMPSTE24* | 0.33 | 8.15E-03 |
| *EFNB1* | *ZNF107* | 0.32 | 1.04E-02 |
| *EFNB1* | *ZNF205* | 0.36 | 3.94E-03 |
| *EFNB1* | *ZNF213* | 0.32 | 1.22E-02 |
| *EFNB1* | *ZNF236* | 0.30 | 1.62E-02 |
| *EFNB1* | *ZNF25* | 0.30 | 1.62E-02 |
| *EFNB1* | *ZNF263* | 0.38 | 2.55E-03 |
| *EFNB1* | *ZNF267* | 0.43 | 5.10E-04 |
| *EFNB1* | *ZNF277* | 0.39 | 1.51E-03 |
| *EFNB1* | *ZNF280C* | 0.31 | 1.44E-02 |
| *EFNB1* | *ZNF384* | 0.34 | 7.65E-03 |
| *EFNB1* | *ZNF500* | 0.32 | 1.10E-02 |
| *EFNB1* | *ZNF532* | 0.32 | 1.01E-02 |
| *EFNB1* | *ZNF680* | 0.31 | 1.31E-02 |
| *EFNB1* | *ZNF69* | -0.36 | 4.10E-03 |
| *EFNB1* | *ZNF771* | 0.30 | 1.73E-02 |
| *EFNB1* | *ZNF830* | 0.36 | 4.59E-03 |
| *EFNB1* | *ZNF843* | 0.32 | 1.18E-02 |
| *EFNB1* | *ZNF92* | 0.34 | 6.79E-03 |
| *EFNB1* | *ZSCAN21* | 0.31 | 1.52E-02 |
| *EFNB1* | *ZSCAN32* | 0.39 | 1.70E-03 |
| *EFNB1* | *ZSWIM1* | 0.32 | 1.16E-02 |
| *EPHB4* | *A1BG-AS1* | 0.32 | 1.22E-02 |
| *EPHB4* | *A2M-AS1* | 0.38 | 2.43E-03 |
| *EPHB4* | *AACS* | 0.33 | 7.78E-03 |
| *EPHB4* | *AASDHPPT* | -0.33 | 9.10E-03 |
| *EPHB4* | *AASS* | 0.32 | 9.96E-03 |
| *EPHB4* | *AATBC* | 0.47 | 1.08E-04 |
| *EPHB4* | *ABAT* | 0.31 | 1.58E-02 |
| *EPHB4* | *ABCA1* | 0.31 | 1.31E-02 |
| *EPHB4* | *ABCA10* | 0.32 | 1.06E-02 |
| *EPHB4* | *ABCA2* | 0.33 | 8.77E-03 |
| *EPHB4* | *ABCA7* | 0.33 | 8.10E-03 |
| *EPHB4* | *ABCB10* | 0.31 | 1.42E-02 |
| *EPHB4* | *ABCB6* | 0.35 | 4.79E-03 |
| *EPHB4* | *ABCB9* | 0.42 | 7.46E-04 |
| *EPHB4* | *ABCC1* | 0.31 | 1.53E-02 |
| *EPHB4* | *ABCC10* | 0.41 | 1.00E-03 |
| *EPHB4* | *ABCC4* | 0.35 | 5.48E-03 |
| *EPHB4* | *ABHD15* | 0.39 | 1.56E-03 |
| *EPHB4* | *ABL1* | 0.44 | 3.73E-04 |
| *EPHB4* | *ABTB2* | 0.30 | 1.68E-02 |
| *EPHB4* | *AC000068.5* | 0.39 | 1.85E-03 |
| *EPHB4* | *AC004540.5* | 0.31 | 1.56E-02 |
| *EPHB4* | *AC004893.11* | 0.41 | 8.95E-04 |
| *EPHB4* | *AC004951.6* | 0.38 | 2.54E-03 |
| *EPHB4* | *AC005076.5* | 0.32 | 1.22E-02 |
| *EPHB4* | *AC005363.11* | 0.30 | 1.78E-02 |
| *EPHB4* | *AC005537.2* | 0.42 | 7.46E-04 |
| *EPHB4* | *AC005932.1* | 0.43 | 5.41E-04 |
| *EPHB4* | *AC006116.17* | 0.56 | 2.82E-06 |
| *EPHB4* | *AC007383.3* | 0.30 | 1.67E-02 |
| *EPHB4* | *AC007773.2* | 0.34 | 6.53E-03 |
| *EPHB4* | *AC007950.2* | 0.35 | 5.89E-03 |
| *EPHB4* | *AC008522.1* | 0.39 | 1.60E-03 |
| *EPHB4* | *AC010226.4* | 0.34 | 6.15E-03 |
| *EPHB4* | *AC015971.2* | 0.38 | 2.42E-03 |
| *EPHB4* | *AC018766.4* | 0.37 | 2.89E-03 |
| *EPHB4* | *AC034243.1* | 0.32 | 1.14E-02 |
| *EPHB4* | *AC068499.10* | 0.39 | 1.99E-03 |
| *EPHB4* | *AC073254.1* | 0.35 | 5.50E-03 |
| *EPHB4* | *AC073283.4* | 0.35 | 5.11E-03 |
| *EPHB4* | *AC083843.2* | 0.31 | 1.59E-02 |
| *EPHB4* | *AC084219.4* | 0.47 | 1.03E-04 |
| *EPHB4* | *AC087294.2* | 0.33 | 8.86E-03 |
| *EPHB4* | *AC093375.1* | 0.39 | 1.63E-03 |
| *EPHB4* | *AC093673.5* | 0.40 | 1.30E-03 |
| *EPHB4* | *AC093901.1* | 0.33 | 7.87E-03 |
| *EPHB4* | *AC096772.6* | 0.33 | 9.44E-03 |
| *EPHB4* | *AC099850.1* | 0.31 | 1.45E-02 |
| *EPHB4* | *AC105760.2* | 0.42 | 7.01E-04 |
| *EPHB4* | *AC114271.2* | 0.38 | 2.61E-03 |
| *EPHB4* | *AC114730.8* | 0.31 | 1.40E-02 |
| *EPHB4* | *AC138969.4* | 0.37 | 3.05E-03 |
| *EPHB4* | *AC142472.6* | 0.46 | 1.47E-04 |
| *EPHB4* | *AC142528.1* | 0.48 | 9.08E-05 |
| *EPHB4* | *AC144652.1* | 0.31 | 1.32E-02 |
| *EPHB4* | *AC240274.1* | 0.41 | 8.07E-04 |
| *EPHB4* | *ACACB* | 0.39 | 1.98E-03 |
| *EPHB4* | *ACAD10* | 0.49 | 6.37E-05 |
| *EPHB4* | *ACAD11* | 0.34 | 7.59E-03 |
| *EPHB4* | *ACAD9* | 0.34 | 7.33E-03 |
| *EPHB4* | *ACAP3* | 0.31 | 1.41E-02 |
| *EPHB4* | *ACCS* | 0.33 | 8.74E-03 |
| *EPHB4* | *ACKR1* | 0.35 | 5.37E-03 |
| *EPHB4* | *ACRBP* | 0.34 | 7.47E-03 |
| *EPHB4* | *ACSF2* | 0.30 | 1.74E-02 |
| *EPHB4* | *ACSF3* | 0.32 | 1.26E-02 |
| *EPHB4* | *ACSS1* | 0.39 | 1.80E-03 |
| *EPHB4* | *ACTN1-AS1* | 0.31 | 1.59E-02 |
| *EPHB4* | *ACTN2* | 0.31 | 1.38E-02 |
| *EPHB4* | *ACTR5* | 0.34 | 7.56E-03 |
| *EPHB4* | *ACVR1* | 0.34 | 6.91E-03 |
| *EPHB4* | *ACVR1B* | 0.39 | 1.77E-03 |
| *EPHB4* | *ACVR2B-AS1* | 0.39 | 1.89E-03 |
| *EPHB4* | *ADAMTS18* | 0.32 | 1.04E-02 |
| *EPHB4* | *ADAMTS4* | 0.42 | 7.39E-04 |
| *EPHB4* | *ADAMTS6* | 0.33 | 9.56E-03 |
| *EPHB4* | *ADAMTS9-AS2* | 0.33 | 8.39E-03 |
| *EPHB4* | *ADARB1* | 0.33 | 8.97E-03 |
| *EPHB4* | *ADCK1* | 0.33 | 9.89E-03 |
| *EPHB4* | *ADCK4* | 0.45 | 2.52E-04 |
| *EPHB4* | *ADCY9* | 0.32 | 1.06E-02 |
| *EPHB4* | *ADD1* | 0.30 | 1.77E-02 |
| *EPHB4* | *ADGRA3* | 0.41 | 8.26E-04 |
| *EPHB4* | *ADGRB2* | 0.35 | 5.22E-03 |
| *EPHB4* | *ADGRG1* | 0.37 | 3.29E-03 |
| *EPHB4* | *ADGRG3* | 0.32 | 1.17E-02 |
| *EPHB4* | *ADGRL1* | 0.38 | 2.67E-03 |
| *EPHB4* | *ADGRV1* | 0.31 | 1.43E-02 |
| *EPHB4* | *ADM5* | 0.38 | 2.04E-03 |
| *EPHB4* | *ADPGK* | 0.36 | 4.23E-03 |
| *EPHB4* | *ADPGK-AS1* | 0.41 | 1.02E-03 |
| *EPHB4* | *ADRA2C* | 0.33 | 8.47E-03 |
| *EPHB4* | *ADRBK2* | 0.37 | 3.26E-03 |
| *EPHB4* | *AF011889.5* | 0.44 | 3.62E-04 |
| *EPHB4* | *AFTPH* | 0.31 | 1.35E-02 |
| *EPHB4* | *AGAP6* | 0.35 | 5.37E-03 |
| *EPHB4* | *AGBL2* | 0.31 | 1.27E-02 |
| *EPHB4* | *AGER* | 0.35 | 5.70E-03 |
| *EPHB4* | *AGO1* | 0.43 | 4.86E-04 |
| *EPHB4* | *AGO3* | 0.46 | 1.61E-04 |
| *EPHB4* | *AGO4* | 0.45 | 2.41E-04 |
| *EPHB4* | *AGRN* | 0.47 | 1.07E-04 |
| *EPHB4* | *AHCYL2* | 0.36 | 4.26E-03 |
| *EPHB4* | *AHDC1* | 0.36 | 3.93E-03 |
| *EPHB4* | *AHI1* | 0.34 | 6.32E-03 |
| *EPHB4* | *AHRR* | 0.31 | 1.47E-02 |
| *EPHB4* | *AHSA2* | 0.32 | 1.12E-02 |
| *EPHB4* | *AIM1* | 0.36 | 4.55E-03 |
| *EPHB4* | *AJUBA* | 0.37 | 2.76E-03 |
| *EPHB4* | *AKAP10* | 0.40 | 1.22E-03 |
| *EPHB4* | *AKAP9* | 0.33 | 8.79E-03 |
| *EPHB4* | *AKIRIN1* | 0.31 | 1.45E-02 |
| *EPHB4* | *AKT1* | 0.39 | 1.83E-03 |
| *EPHB4* | *AL132709.8* | 0.35 | 5.96E-03 |
| *EPHB4* | *AL513523.2* | 0.32 | 1.25E-02 |
| *EPHB4* | *ALDH16A1* | 0.33 | 9.88E-03 |
| *EPHB4* | *ALKBH4* | 0.35 | 5.46E-03 |
| *EPHB4* | *ALMS1* | 0.33 | 8.09E-03 |
| *EPHB4* | *ALOX12B* | 0.34 | 7.19E-03 |
| *EPHB4* | *ALS2* | 0.48 | 9.24E-05 |
| *EPHB4* | *AMBRA1* | 0.35 | 4.65E-03 |
| *EPHB4* | *AMER1* | 0.34 | 6.06E-03 |
| *EPHB4* | *AMH* | 0.31 | 1.51E-02 |
| *EPHB4* | *AMMECR1L* | 0.34 | 7.29E-03 |
| *EPHB4* | *AMT* | 0.33 | 8.84E-03 |
| *EPHB4* | *ANAPC7* | 0.31 | 1.32E-02 |
| *EPHB4* | *ANGEL1* | 0.41 | 9.23E-04 |
| *EPHB4* | *ANGEL2* | 0.31 | 1.32E-02 |
| *EPHB4* | *ANKDD1B* | 0.34 | 7.48E-03 |
| *EPHB4* | *ANKFY1* | 0.34 | 6.60E-03 |
| *EPHB4* | *ANKIB1* | 0.35 | 4.77E-03 |
| *EPHB4* | *ANKMY1* | 0.38 | 2.56E-03 |
| *EPHB4* | *ANKMY2* | 0.30 | 1.62E-02 |
| *EPHB4* | *ANKRD11* | 0.32 | 1.14E-02 |
| *EPHB4* | *ANKRD26* | 0.33 | 8.10E-03 |
| *EPHB4* | *ANKRD36B* | 0.33 | 8.50E-03 |
| *EPHB4* | *ANKRD50* | 0.35 | 4.75E-03 |
| *EPHB4* | *ANKRD52* | 0.45 | 2.10E-04 |
| *EPHB4* | *ANKRD54* | 0.38 | 2.64E-03 |
| *EPHB4* | *ANKS3* | 0.44 | 3.82E-04 |
| *EPHB4* | *ANKZF1* | 0.35 | 5.41E-03 |
| *EPHB4* | *ANO1* | 0.41 | 1.04E-03 |
| *EPHB4* | *ANO1-AS1* | 0.40 | 1.12E-03 |
| *EPHB4* | *AP000487.5* | 0.39 | 1.63E-03 |
| *EPHB4* | *AP001046.5* | 0.32 | 1.09E-02 |
| *EPHB4* | *AP1S1* | 0.33 | 9.66E-03 |
| *EPHB4* | *AP2A1* | 0.30 | 1.60E-02 |
| *EPHB4* | *AP2A2* | 0.38 | 2.24E-03 |
| *EPHB4* | *AP4M1* | 0.43 | 4.12E-04 |
| *EPHB4* | *AP5Z1* | 0.38 | 2.56E-03 |
| *EPHB4* | *APAF1* | 0.36 | 3.73E-03 |
| *EPHB4* | *APBB2* | 0.34 | 7.24E-03 |
| *EPHB4* | *APBB3* | 0.45 | 2.79E-04 |
| *EPHB4* | *APC* | 0.51 | 1.95E-05 |
| *EPHB4* | *APH1B* | 0.35 | 4.87E-03 |
| *EPHB4* | *APLNR* | 0.37 | 3.37E-03 |
| *EPHB4* | *APMAP* | 0.37 | 3.37E-03 |
| *EPHB4* | *APOBEC3F* | 0.36 | 3.70E-03 |
| *EPHB4* | *APOPT1* | 0.31 | 1.43E-02 |
| *EPHB4* | *APPBP2* | 0.33 | 9.51E-03 |
| *EPHB4* | *APPL2* | 0.35 | 4.82E-03 |
| *EPHB4* | *APTR* | 0.34 | 7.66E-03 |
| *EPHB4* | *ARHGAP12* | 0.34 | 7.23E-03 |
| *EPHB4* | *ARHGAP22* | 0.33 | 8.92E-03 |
| *EPHB4* | *ARHGAP25* | 0.42 | 7.89E-04 |
| *EPHB4* | *ARHGAP31* | 0.32 | 1.06E-02 |
| *EPHB4* | *ARHGAP35* | 0.52 | 1.31E-05 |
| *EPHB4* | *ARHGEF1* | 0.34 | 7.10E-03 |
| *EPHB4* | *ARHGEF10L* | 0.35 | 5.81E-03 |
| *EPHB4* | *ARHGEF11* | 0.31 | 1.44E-02 |
| *EPHB4* | *ARHGEF16* | 0.31 | 1.40E-02 |
| *EPHB4* | *ARHGEF17* | 0.35 | 5.20E-03 |
| *EPHB4* | *ARHGEF19* | 0.59 | 5.61E-07 |
| *EPHB4* | *ARHGEF2* | 0.31 | 1.59E-02 |
| *EPHB4* | *ARHGEF26* | 0.33 | 9.82E-03 |
| *EPHB4* | *ARHGEF28* | 0.37 | 3.18E-03 |
| *EPHB4* | *ARHGEF40* | 0.34 | 6.42E-03 |
| *EPHB4* | *ARID1A* | 0.31 | 1.40E-02 |
| *EPHB4* | *ARID1B* | 0.35 | 4.65E-03 |
| *EPHB4* | *ARL10* | 0.31 | 1.51E-02 |
| *EPHB4* | *ARL16* | 0.37 | 2.91E-03 |
| *EPHB4* | *ARL17A* | 0.31 | 1.47E-02 |
| *EPHB4* | *ARL4C* | 0.34 | 7.32E-03 |
| *EPHB4* | *ARMC2* | 0.31 | 1.48E-02 |
| *EPHB4* | *ARMC5* | 0.34 | 6.38E-03 |
| *EPHB4* | *ARMC7* | 0.37 | 3.42E-03 |
| *EPHB4* | *ARMC8* | 0.37 | 3.32E-03 |
| *EPHB4* | *ARSD* | 0.30 | 1.78E-02 |
| *EPHB4* | *ASAP3* | 0.33 | 7.98E-03 |
| *EPHB4* | *ASB3* | 0.33 | 8.24E-03 |
| *EPHB4* | *ASCL2* | 0.36 | 3.67E-03 |
| *EPHB4* | *ASH1L* | 0.34 | 6.05E-03 |
| *EPHB4* | *ASMTL-AS1* | 0.36 | 4.57E-03 |
| *EPHB4* | *ASPSCR1* | 0.37 | 3.01E-03 |
| *EPHB4* | *ASTE1* | 0.40 | 1.43E-03 |
| *EPHB4* | *ASXL2* | 0.33 | 8.71E-03 |
| *EPHB4* | *ATE1-AS1* | 0.37 | 3.11E-03 |
| *EPHB4* | *ATF5* | 0.31 | 1.28E-02 |
| *EPHB4* | *ATF7IP* | 0.36 | 4.21E-03 |
| *EPHB4* | *ATG101* | 0.32 | 1.07E-02 |
| *EPHB4* | *ATG14* | 0.32 | 1.22E-02 |
| *EPHB4* | *ATG2A* | 0.33 | 9.05E-03 |
| *EPHB4* | *ATG2B* | 0.36 | 3.98E-03 |
| *EPHB4* | *ATG4B* | 0.42 | 7.52E-04 |
| *EPHB4* | *ATG9A* | 0.41 | 1.09E-03 |
| *EPHB4* | *ATN1* | 0.46 | 1.75E-04 |
| *EPHB4* | *ATP13A1* | 0.43 | 4.99E-04 |
| *EPHB4* | *ATP1B2* | 0.37 | 3.48E-03 |
| *EPHB4* | *ATP1B3* | 0.36 | 3.75E-03 |
| *EPHB4* | *ATP2A2* | 0.38 | 2.47E-03 |
| *EPHB4* | *ATP2C1* | 0.46 | 1.87E-04 |
| *EPHB4* | *ATP5L* | -0.35 | 5.39E-03 |
| *EPHB4* | *ATP6V0A2* | 0.45 | 2.45E-04 |
| *EPHB4* | *ATP8A2* | 0.36 | 4.17E-03 |
| *EPHB4* | *ATR* | 0.42 | 6.88E-04 |
| *EPHB4* | *ATRN* | 0.31 | 1.42E-02 |
| *EPHB4* | *ATXN2* | 0.34 | 7.47E-03 |
| *EPHB4* | *ATXN7L1* | 0.52 | 1.73E-05 |
| *EPHB4* | *ATXN7L2* | 0.32 | 1.09E-02 |
| *EPHB4* | *ATXN7L3* | 0.47 | 1.34E-04 |
| *EPHB4* | *AUTS2* | 0.38 | 2.37E-03 |
| *EPHB4* | *AVIL* | 0.31 | 1.46E-02 |
| *EPHB4* | *AVL9* | 0.37 | 3.17E-03 |
| *EPHB4* | *AXIN1* | 0.34 | 7.27E-03 |
| *EPHB4* | *AXIN2* | 0.32 | 1.17E-02 |
| *EPHB4* | *AZIN2* | 0.38 | 2.20E-03 |
| *EPHB4* | *B3GLCT* | 0.40 | 1.27E-03 |
| *EPHB4* | *B3GNT2* | 0.32 | 1.11E-02 |
| *EPHB4* | *B4GALNT4* | 0.42 | 6.40E-04 |
| *EPHB4* | *BAHCC1* | 0.30 | 1.75E-02 |
| *EPHB4* | *BAHD1* | 0.37 | 3.29E-03 |
| *EPHB4* | *BAIAP2-AS1* | 0.49 | 6.43E-05 |
| *EPHB4* | *BAIAP2L1* | 0.35 | 5.03E-03 |
| *EPHB4* | *BANK1* | 0.33 | 7.89E-03 |
| *EPHB4* | *BAZ1B* | 0.45 | 2.65E-04 |
| *EPHB4* | *BAZ2A* | 0.32 | 1.15E-02 |
| *EPHB4* | *BAZ2B* | 0.37 | 3.51E-03 |
| *EPHB4* | *BBOX1-AS1* | 0.31 | 1.31E-02 |
| *EPHB4* | *BBS9* | 0.41 | 8.45E-04 |
| *EPHB4* | *BCAM* | 0.32 | 1.26E-02 |
| *EPHB4* | *BCAS3* | 0.40 | 1.14E-03 |
| *EPHB4* | *BCL7A* | 0.50 | 4.21E-05 |
| *EPHB4* | *BCL9* | 0.43 | 4.43E-04 |
| *EPHB4* | *BCL9L* | 0.32 | 1.13E-02 |
| *EPHB4* | *BCOR* | 0.36 | 4.33E-03 |
| *EPHB4* | *BEND3* | 0.44 | 3.01E-04 |
| *EPHB4* | *BGLAP* | 0.35 | 4.71E-03 |
| *EPHB4* | *BHLHE41* | 0.34 | 7.75E-03 |
| *EPHB4* | *BICD1* | 0.39 | 1.96E-03 |
| *EPHB4* | *BIRC6* | 0.37 | 2.98E-03 |
| *EPHB4* | *BLCAP* | 0.30 | 1.69E-02 |
| *EPHB4* | *BMF* | 0.32 | 1.25E-02 |
| *EPHB4* | *BMP1* | 0.45 | 2.25E-04 |
| *EPHB4* | *BMPER* | 0.36 | 3.86E-03 |
| *EPHB4* | *BMPR1A* | 0.34 | 7.05E-03 |
| *EPHB4* | *BMPR2* | 0.40 | 1.33E-03 |
| *EPHB4* | *BOC* | 0.40 | 1.23E-03 |
| *EPHB4* | *BOD1L1* | 0.36 | 4.55E-03 |
| *EPHB4* | *BOLA1* | 0.35 | 4.87E-03 |
| *EPHB4* | *BORCS5* | 0.31 | 1.54E-02 |
| *EPHB4* | *BORCS8-MEF2B* | 0.42 | 6.99E-04 |
| *EPHB4* | *BPTF* | 0.36 | 3.80E-03 |
| *EPHB4* | *BRAP* | 0.41 | 9.16E-04 |
| *EPHB4* | *BRAT1* | 0.42 | 6.41E-04 |
| *EPHB4* | *BRD2* | 0.30 | 1.68E-02 |
| *EPHB4* | *BRD9* | 0.43 | 4.63E-04 |
| *EPHB4* | *BRF1* | 0.32 | 9.96E-03 |
| *EPHB4* | *BRICD5* | 0.44 | 3.64E-04 |
| *EPHB4* | *BRPF1* | 0.31 | 1.59E-02 |
| *EPHB4* | *BRPF3* | 0.41 | 8.96E-04 |
| *EPHB4* | *BRSK1* | 0.42 | 7.72E-04 |
| *EPHB4* | *BSDC1* | 0.31 | 1.53E-02 |
| *EPHB4* | *BTBD6* | 0.31 | 1.57E-02 |
| *EPHB4* | *BTG1* | 0.33 | 9.59E-03 |
| *EPHB4* | *BTN2A2* | 0.45 | 2.88E-04 |
| *EPHB4* | *BUD31* | 0.34 | 7.55E-03 |
| *EPHB4* | *BZRAP1* | 0.31 | 1.28E-02 |
| *EPHB4* | *BZRAP1-AS1* | 0.40 | 1.11E-03 |
| *EPHB4* | *C10orf111* | 0.30 | 1.66E-02 |
| *EPHB4* | *C10orf12* | 0.41 | 1.05E-03 |
| *EPHB4* | *C10orf67* | 0.31 | 1.36E-02 |
| *EPHB4* | *C12orf65* | 0.53 | 9.27E-06 |
| *EPHB4* | *C12orf76* | 0.38 | 2.20E-03 |
| *EPHB4* | *C16orf71* | 0.39 | 1.50E-03 |
| *EPHB4* | *C17orf107* | 0.39 | 1.61E-03 |
| *EPHB4* | *C17orf51* | 0.42 | 6.24E-04 |
| *EPHB4* | *C17orf62* | 0.33 | 9.94E-03 |
| *EPHB4* | *C17orf80* | 0.34 | 6.67E-03 |
| *EPHB4* | *C19orf44* | 0.33 | 8.42E-03 |
| *EPHB4* | *C19orf68* | 0.37 | 3.35E-03 |
| *EPHB4* | *C1orf159* | 0.37 | 2.95E-03 |
| *EPHB4* | *C1orf228* | 0.50 | 3.98E-05 |
| *EPHB4* | *C1QTNF1* | 0.34 | 7.29E-03 |
| *EPHB4* | *C1RL* | 0.42 | 5.77E-04 |
| *EPHB4* | *C1RL-AS1* | 0.46 | 1.97E-04 |
| *EPHB4* | *C20orf195* | 0.33 | 7.81E-03 |
| *EPHB4* | *C2CD4B* | 0.31 | 1.51E-02 |
| *EPHB4* | *C2CD5* | 0.39 | 1.53E-03 |
| *EPHB4* | *C2orf40* | 0.30 | 1.69E-02 |
| *EPHB4* | *C3orf33* | 0.30 | 1.77E-02 |
| *EPHB4* | *C3orf58* | 0.30 | 1.69E-02 |
| *EPHB4* | *C3orf62* | 0.35 | 4.80E-03 |
| *EPHB4* | *C4orf47* | 0.37 | 2.84E-03 |
| *EPHB4* | *C5* | 0.44 | 3.87E-04 |
| *EPHB4* | *C5AR1* | 0.37 | 3.22E-03 |
| *EPHB4* | *C5orf45* | 0.46 | 1.79E-04 |
| *EPHB4* | *C6orf25* | 0.39 | 1.56E-03 |
| *EPHB4* | *C6orf62* | 0.32 | 1.14E-02 |
| *EPHB4* | *C7orf26* | 0.35 | 5.74E-03 |
| *EPHB4* | *C7orf61* | 0.42 | 7.38E-04 |
| *EPHB4* | *C8orf59* | -0.33 | 9.07E-03 |
| *EPHB4* | *C9orf47* | 0.32 | 1.22E-02 |
| *EPHB4* | *CA11* | 0.35 | 5.15E-03 |
| *EPHB4* | *CABLES1* | 0.40 | 1.11E-03 |
| *EPHB4* | *CABP1* | 0.30 | 1.72E-02 |
| *EPHB4* | *CACHD1* | 0.35 | 5.17E-03 |
| *EPHB4* | *CACNA1C* | 0.37 | 2.94E-03 |
| *EPHB4* | *CACNA2D2* | 0.32 | 1.23E-02 |
| *EPHB4* | *CACNA2D4* | 0.32 | 1.25E-02 |
| *EPHB4* | *CACNB1* | 0.36 | 4.09E-03 |
| *EPHB4* | *CACTIN* | 0.32 | 1.11E-02 |
| *EPHB4* | *CAD* | 0.52 | 1.39E-05 |
| *EPHB4* | *CALCOCO1* | 0.34 | 7.28E-03 |
| *EPHB4* | *CAMK2B* | 0.32 | 1.00E-02 |
| *EPHB4* | *CAMKK2* | 0.35 | 5.13E-03 |
| *EPHB4* | *CAND1* | 0.39 | 1.92E-03 |
| *EPHB4* | *CAND2* | 0.31 | 1.44E-02 |
| *EPHB4* | *CANT1* | 0.36 | 4.19E-03 |
| *EPHB4* | *CAPN10-AS1* | 0.33 | 9.65E-03 |
| *EPHB4* | *CAPN12* | 0.38 | 2.58E-03 |
| *EPHB4* | *CAPN13* | 0.31 | 1.32E-02 |
| *EPHB4* | *CAPN15* | 0.44 | 2.99E-04 |
| *EPHB4* | *CAPN3* | 0.42 | 7.94E-04 |
| *EPHB4* | *CAPRIN2* | 0.51 | 2.24E-05 |
| *EPHB4* | *CAPS2* | 0.32 | 1.11E-02 |
| *EPHB4* | *CARD10* | 0.32 | 1.11E-02 |
| *EPHB4* | *CARD8* | 0.33 | 8.47E-03 |
| *EPHB4* | *CARF* | 0.45 | 2.57E-04 |
| *EPHB4* | *CARMN* | 0.35 | 5.06E-03 |
| *EPHB4* | *CASC15* | 0.41 | 1.02E-03 |
| *EPHB4* | *CASD1* | 0.43 | 4.16E-04 |
| *EPHB4* | *CASS4* | 0.38 | 2.02E-03 |
| *EPHB4* | *CASZ1* | 0.35 | 4.89E-03 |
| *EPHB4* | *CATSPER2* | 0.38 | 2.45E-03 |
| *EPHB4* | *CATSPERG* | 0.32 | 1.03E-02 |
| *EPHB4* | *CBX4* | 0.41 | 1.00E-03 |
| *EPHB4* | *CC2D1A* | 0.41 | 8.81E-04 |
| *EPHB4* | *CC2D1B* | 0.41 | 8.48E-04 |
| *EPHB4* | *CCAR2* | 0.34 | 6.82E-03 |
| *EPHB4* | *CCDC106* | 0.31 | 1.42E-02 |
| *EPHB4* | *CCDC130* | 0.31 | 1.53E-02 |
| *EPHB4* | *CCDC14* | 0.45 | 2.89E-04 |
| *EPHB4* | *CCDC142* | 0.40 | 1.31E-03 |
| *EPHB4* | *CCDC144A* | 0.35 | 5.61E-03 |
| *EPHB4* | *CCDC146* | 0.38 | 2.23E-03 |
| *EPHB4* | *CCDC149* | 0.40 | 1.48E-03 |
| *EPHB4* | *CCDC154* | 0.31 | 1.31E-02 |
| *EPHB4* | *CCDC157* | 0.38 | 2.26E-03 |
| *EPHB4* | *CCDC163P* | 0.32 | 1.00E-02 |
| *EPHB4* | *CCDC3* | 0.32 | 1.02E-02 |
| *EPHB4* | *CCDC30* | 0.31 | 1.52E-02 |
| *EPHB4* | *CCDC40* | 0.53 | 8.35E-06 |
| *EPHB4* | *CCDC7.1* | 0.31 | 1.41E-02 |
| *EPHB4* | *CCDC85C* | 0.44 | 3.76E-04 |
| *EPHB4* | *CCDC91* | 0.31 | 1.50E-02 |
| *EPHB4* | *CCM2* | 0.32 | 1.02E-02 |
| *EPHB4* | *CCNK* | 0.34 | 6.97E-03 |
| *EPHB4* | *CCNL2* | 0.45 | 2.58E-04 |
| *EPHB4* | *CCNT1* | 0.30 | 1.76E-02 |
| *EPHB4* | *CCNT2* | 0.34 | 7.55E-03 |
| *EPHB4* | *CCSAP* | 0.31 | 1.49E-02 |
| *EPHB4* | *CD200* | 0.33 | 8.72E-03 |
| *EPHB4* | *CD37* | 0.42 | 6.05E-04 |
| *EPHB4* | *CD81-AS1* | 0.35 | 5.93E-03 |
| *EPHB4* | *CD96* | 0.32 | 1.19E-02 |
| *EPHB4* | *CDAN1* | 0.33 | 8.74E-03 |
| *EPHB4* | *CDC14A* | 0.43 | 4.47E-04 |
| *EPHB4* | *CDC40* | 0.34 | 7.43E-03 |
| *EPHB4* | *CDC42BPG* | 0.33 | 7.82E-03 |
| *EPHB4* | *CDH11* | 0.32 | 1.02E-02 |
| *EPHB4* | *CDH23* | 0.46 | 1.65E-04 |
| *EPHB4* | *CDHR3* | 0.30 | 1.75E-02 |
| *EPHB4* | *CDK10* | 0.35 | 5.89E-03 |
| *EPHB4* | *CDK11A* | 0.49 | 6.25E-05 |
| *EPHB4* | *CDK13* | 0.33 | 9.31E-03 |
| *EPHB4* | *CDK18* | 0.49 | 6.01E-05 |
| *EPHB4* | *CDK5RAP3* | 0.33 | 8.90E-03 |
| *EPHB4* | *CDK6* | 0.36 | 4.17E-03 |
| *EPHB4* | *CDKL2* | 0.32 | 1.02E-02 |
| *EPHB4* | *CDKL3* | 0.37 | 2.89E-03 |
| *EPHB4* | *CEACAM21* | 0.34 | 7.10E-03 |
| *EPHB4* | *CECR5-AS1* | 0.31 | 1.34E-02 |
| *EPHB4* | *CELF1* | 0.32 | 1.26E-02 |
| *EPHB4* | *CELSR1* | 0.40 | 1.31E-03 |
| *EPHB4* | *CELSR2* | 0.35 | 5.58E-03 |
| *EPHB4* | *CENPB* | 0.42 | 5.82E-04 |
| *EPHB4* | *CENPBD1* | 0.36 | 4.33E-03 |
| *EPHB4* | *CEP131* | 0.40 | 1.31E-03 |
| *EPHB4* | *CEP135* | 0.35 | 5.65E-03 |
| *EPHB4* | *CEP162* | 0.33 | 7.80E-03 |
| *EPHB4* | *CEP170B* | 0.39 | 1.57E-03 |
| *EPHB4* | *CEP250* | 0.37 | 2.79E-03 |
| *EPHB4* | *CEP68* | 0.35 | 5.70E-03 |
| *EPHB4* | *CEP83-AS1* | 0.34 | 7.08E-03 |
| *EPHB4* | *CEP95* | 0.35 | 4.89E-03 |
| *EPHB4* | *CERK* | 0.41 | 9.81E-04 |
| *EPHB4* | *CFAP161* | 0.35 | 5.14E-03 |
| *EPHB4* | *CFAP43* | 0.35 | 4.90E-03 |
| *EPHB4* | *CFAP69* | 0.40 | 1.19E-03 |
| *EPHB4* | *CGB7* | 0.40 | 1.13E-03 |
| *EPHB4* | *CH507-254M2.3* | 0.35 | 5.02E-03 |
| *EPHB4* | *CHCHD4* | -0.33 | 8.92E-03 |
| *EPHB4* | *CHCHD6* | 0.32 | 1.13E-02 |
| *EPHB4* | *CHD3* | 0.50 | 3.27E-05 |
| *EPHB4* | *CHD6* | 0.37 | 2.83E-03 |
| *EPHB4* | *CHD8* | 0.40 | 1.36E-03 |
| *EPHB4* | *CHI3L2* | 0.31 | 1.54E-02 |
| *EPHB4* | *CHKB* | 0.33 | 7.94E-03 |
| *EPHB4* | *CHMP1B* | -0.39 | 1.95E-03 |
| *EPHB4* | *CHPF2* | 0.38 | 2.65E-03 |
| *EPHB4* | *CHRM3* | 0.37 | 2.76E-03 |
| *EPHB4* | *CHRNA10* | 0.38 | 2.09E-03 |
| *EPHB4* | *CHRNA7* | 0.33 | 8.47E-03 |
| *EPHB4* | *CHRNE* | 0.32 | 1.16E-02 |
| *EPHB4* | *CHST12* | 0.31 | 1.48E-02 |
| *EPHB4* | *CHST3* | 0.36 | 4.44E-03 |
| *EPHB4* | *CIC* | 0.55 | 3.73E-06 |
| *EPHB4* | *CIITA* | 0.38 | 2.42E-03 |
| *EPHB4* | *CILP2* | 0.36 | 3.69E-03 |
| *EPHB4* | *CIZ1* | 0.36 | 4.23E-03 |
| *EPHB4* | *CKAP4* | 0.46 | 1.91E-04 |
| *EPHB4* | *CKMT2-AS1* | 0.37 | 2.81E-03 |
| *EPHB4* | *CLASRP* | 0.42 | 5.96E-04 |
| *EPHB4* | *CLCN2* | 0.43 | 4.28E-04 |
| *EPHB4* | *CLCN3* | 0.31 | 1.40E-02 |
| *EPHB4* | *CLCN6* | 0.33 | 8.57E-03 |
| *EPHB4* | *CLCN7* | 0.41 | 8.93E-04 |
| *EPHB4* | *CLDN11* | 0.34 | 6.21E-03 |
| *EPHB4* | *CLDN15* | 0.52 | 1.44E-05 |
| *EPHB4* | *CLEC2D* | 0.36 | 3.78E-03 |
| *EPHB4* | *CLHC1* | 0.31 | 1.28E-02 |
| *EPHB4* | *CLPTM1L* | 0.35 | 5.45E-03 |
| *EPHB4* | *CLSTN1* | 0.51 | 2.78E-05 |
| *EPHB4* | *CLSTN3* | 0.57 | 1.70E-06 |
| *EPHB4* | *CLTCL1* | 0.37 | 2.73E-03 |
| *EPHB4* | *CMKLR1* | 0.34 | 7.18E-03 |
| *EPHB4* | *CNIH3* | 0.33 | 9.63E-03 |
| *EPHB4* | *CNNM3* | 0.42 | 7.45E-04 |
| *EPHB4* | *CNOT3* | 0.37 | 3.22E-03 |
| *EPHB4* | *CNOT6* | 0.31 | 1.52E-02 |
| *EPHB4* | *CNPY3* | 0.33 | 9.83E-03 |
| *EPHB4* | *CNPY4* | 0.46 | 1.46E-04 |
| *EPHB4* | *CNTLN* | 0.46 | 1.90E-04 |
| *EPHB4* | *CNTRL* | 0.39 | 1.87E-03 |
| *EPHB4* | *CNTROB* | 0.41 | 1.02E-03 |
| *EPHB4* | *COASY* | 0.33 | 7.94E-03 |
| *EPHB4* | *COG5* | 0.35 | 5.74E-03 |
| *EPHB4* | *COL13A1* | 0.31 | 1.47E-02 |
| *EPHB4* | *COL16A1* | 0.42 | 5.89E-04 |
| *EPHB4* | *COL18A1* | 0.45 | 2.78E-04 |
| *EPHB4* | *COL23A1* | 0.42 | 7.24E-04 |
| *EPHB4* | *COL27A1* | 0.38 | 2.11E-03 |
| *EPHB4* | *COL4A5* | 0.52 | 1.23E-05 |
| *EPHB4* | *COL4A6* | 0.38 | 2.42E-03 |
| *EPHB4* | *COL7A1* | 0.52 | 1.52E-05 |
| *EPHB4* | *COL9A2* | 0.44 | 4.01E-04 |
| *EPHB4* | *COPS7B* | 0.32 | 1.15E-02 |
| *EPHB4* | *COX18* | 0.40 | 1.46E-03 |
| *EPHB4* | *CPD* | 0.35 | 5.67E-03 |
| *EPHB4* | *CPNE5* | 0.35 | 4.71E-03 |
| *EPHB4* | *CPSF4* | 0.37 | 3.14E-03 |
| *EPHB4* | *CPT1A* | 0.30 | 1.67E-02 |
| *EPHB4* | *CPT1B* | 0.44 | 3.23E-04 |
| *EPHB4* | *CRACR2A* | 0.35 | 4.85E-03 |
| *EPHB4* | *CRAMP1* | 0.35 | 4.74E-03 |
| *EPHB4* | *CRCP* | 0.35 | 4.79E-03 |
| *EPHB4* | *CREB1* | 0.45 | 2.60E-04 |
| *EPHB4* | *CREBBP* | 0.35 | 5.40E-03 |
| *EPHB4* | *CREBL2* | 0.34 | 6.19E-03 |
| *EPHB4* | *CRIM1* | 0.36 | 3.71E-03 |
| *EPHB4* | *CRISPLD2* | 0.30 | 1.60E-02 |
| *EPHB4* | *CRTC1* | 0.42 | 6.05E-04 |
| *EPHB4* | *CRYGN* | 0.38 | 2.41E-03 |
| *EPHB4* | *CSAD* | 0.38 | 2.25E-03 |
| *EPHB4* | *CSMD1* | 0.43 | 4.71E-04 |
| *EPHB4* | *CSMD2* | 0.35 | 5.16E-03 |
| *EPHB4* | *CSNK1G2* | 0.35 | 5.88E-03 |
| *EPHB4* | *CSNK2A3* | 0.37 | 2.73E-03 |
| *EPHB4* | *CSRNP2* | 0.48 | 9.23E-05 |
| *EPHB4* | *CTA-204B4.2* | 0.37 | 3.27E-03 |
| *EPHB4* | *CTA-29F11.1* | 0.32 | 1.01E-02 |
| *EPHB4* | *CTA-384D8.36* | 0.37 | 2.78E-03 |
| *EPHB4* | *CTB-113I20.2* | 0.32 | 1.08E-02 |
| *EPHB4* | *CTB-119C2.1* | 0.45 | 2.29E-04 |
| *EPHB4* | *CTB-193M12.5* | 0.43 | 4.88E-04 |
| *EPHB4* | *CTB-25B13.12* | 0.39 | 1.76E-03 |
| *EPHB4* | *CTB-50L17.16* | 0.36 | 4.02E-03 |
| *EPHB4* | *CTB-58E17.3* | 0.32 | 1.13E-02 |
| *EPHB4* | *CTBP1-AS2* | 0.38 | 2.04E-03 |
| *EPHB4* | *CTBS* | 0.35 | 5.33E-03 |
| *EPHB4* | *CTC-241N9.1* | 0.43 | 4.42E-04 |
| *EPHB4* | *CTC-250I14.6* | 0.33 | 9.44E-03 |
| *EPHB4* | *CTC-301O7.4* | 0.35 | 5.95E-03 |
| *EPHB4* | *CTC-321K16.1* | 0.31 | 1.53E-02 |
| *EPHB4* | *CTC-444N24.8* | 0.38 | 2.10E-03 |
| *EPHB4* | *CTC-471J1.11* | 0.40 | 1.44E-03 |
| *EPHB4* | *CTC-490E21.14* | 0.31 | 1.51E-02 |
| *EPHB4* | *CTC-497E21.3* | 0.49 | 5.05E-05 |
| *EPHB4* | *CTC-499B15.5* | 0.34 | 7.28E-03 |
| *EPHB4* | *CTC-559E9.1* | 0.43 | 4.53E-04 |
| *EPHB4* | *CTC1* | 0.34 | 6.55E-03 |
| *EPHB4* | *CTD-2008L17.1* | 0.36 | 3.87E-03 |
| *EPHB4* | *CTD-2020K17.3* | 0.40 | 1.38E-03 |
| *EPHB4* | *CTD-2047H16.4* | 0.34 | 7.41E-03 |
| *EPHB4* | *CTD-2095E4.5* | 0.42 | 6.10E-04 |
| *EPHB4* | *CTD-2105E13.6* | 0.31 | 1.34E-02 |
| *EPHB4* | *CTD-2194D22.3* | 0.32 | 1.24E-02 |
| *EPHB4* | *CTD-2231H16.1* | 0.44 | 3.37E-04 |
| *EPHB4* | *CTD-2270P14.5* | 0.34 | 7.46E-03 |
| *EPHB4* | *CTD-2287O16.5* | 0.33 | 8.99E-03 |
| *EPHB4* | *CTD-2302E22.4* | 0.37 | 2.80E-03 |
| *EPHB4* | *CTD-2302E22.6* | 0.31 | 1.57E-02 |
| *EPHB4* | *CTD-2410N18.3* | 0.51 | 2.18E-05 |
| *EPHB4* | *CTD-2506P8.6* | 0.40 | 1.33E-03 |
| *EPHB4* | *CTD-2517O10.6* | 0.38 | 2.39E-03 |
| *EPHB4* | *CTD-2541J13.2* | 0.34 | 7.39E-03 |
| *EPHB4* | *CTD-2547G23.4* | 0.39 | 1.99E-03 |
| *EPHB4* | *CTD-2555O16.2* | 0.41 | 8.43E-04 |
| *EPHB4* | *CTD-2562J15.6* | 0.34 | 6.81E-03 |
| *EPHB4* | *CTD-2583A14.10* | 0.33 | 8.29E-03 |
| *EPHB4* | *CTD-2619J13.3* | 0.41 | 1.09E-03 |
| *EPHB4* | *CTD-2630F21.1* | 0.33 | 9.81E-03 |
| *EPHB4* | *CTD-3001H11.2* | 0.43 | 5.18E-04 |
| *EPHB4* | *CTD-3113P16.11* | 0.36 | 4.13E-03 |
| *EPHB4* | *CTD-3203P2.3* | 0.38 | 2.05E-03 |
| *EPHB4* | *CTD-3214H19.6* | 0.30 | 1.74E-02 |
| *EPHB4* | *CTDSP1* | 0.37 | 3.50E-03 |
| *EPHB4* | *CTU2* | 0.31 | 1.28E-02 |
| *EPHB4* | *CUBN* | 0.39 | 1.97E-03 |
| *EPHB4* | *CUEDC1* | 0.31 | 1.38E-02 |
| *EPHB4* | *CUEDC2* | 0.31 | 1.58E-02 |
| *EPHB4* | *CUL9* | 0.38 | 2.43E-03 |
| *EPHB4* | *CUX1* | 0.42 | 7.45E-04 |
| *EPHB4* | *CUZD1* | 0.30 | 1.64E-02 |
| *EPHB4* | *CXCR4* | 0.35 | 4.89E-03 |
| *EPHB4* | *CXXC4* | 0.36 | 3.90E-03 |
| *EPHB4* | *CYB561D2.1* | 0.34 | 6.63E-03 |
| *EPHB4* | *CYB5D1* | 0.38 | 2.38E-03 |
| *EPHB4* | *CYP20A1* | 0.35 | 5.35E-03 |
| *EPHB4* | *CYP26B1* | 0.31 | 1.49E-02 |
| *EPHB4* | *CYP27C1* | 0.37 | 2.99E-03 |
| *EPHB4* | *CYP2R1* | 0.30 | 1.64E-02 |
| *EPHB4* | *CYP2W1* | 0.35 | 4.80E-03 |
| *EPHB4* | *CYP4X1* | 0.41 | 9.12E-04 |
| *EPHB4* | *CYP51A1-AS1* | 0.46 | 1.80E-04 |
| *EPHB4* | *CYS1* | 0.35 | 5.39E-03 |
| *EPHB4* | *CYTH1* | 0.42 | 5.96E-04 |
| *EPHB4* | *D2HGDH* | 0.31 | 1.32E-02 |
| *EPHB4* | *DAB2IP* | 0.33 | 9.14E-03 |
| *EPHB4* | *DACT3-AS1* | 0.38 | 2.30E-03 |
| *EPHB4* | *DAPK2* | 0.34 | 7.54E-03 |
| *EPHB4* | *DBT* | 0.36 | 4.45E-03 |
| *EPHB4* | *DCAF10* | 0.32 | 1.13E-02 |
| *EPHB4* | *DCAF5* | 0.33 | 8.72E-03 |
| *EPHB4* | *DCAF8* | 0.33 | 9.85E-03 |
| *EPHB4* | *DCHS2* | 0.39 | 1.72E-03 |
| *EPHB4* | *DDHD2* | 0.35 | 5.08E-03 |
| *EPHB4* | *DDIT4L* | 0.35 | 5.09E-03 |
| *EPHB4* | *DDR1* | 0.42 | 7.25E-04 |
| *EPHB4* | *DDX11* | 0.48 | 6.65E-05 |
| *EPHB4* | *DDX51* | 0.53 | 8.62E-06 |
| *EPHB4* | *DECR2* | 0.31 | 1.28E-02 |
| *EPHB4* | *DEF6* | 0.42 | 6.82E-04 |
| *EPHB4* | *DENND4B* | 0.46 | 1.91E-04 |
| *EPHB4* | *DENND5A* | 0.33 | 8.04E-03 |
| *EPHB4* | *DENND6B* | 0.32 | 1.08E-02 |
| *EPHB4* | *DEPDC5* | 0.39 | 1.88E-03 |
| *EPHB4* | *DFFB* | 0.34 | 7.55E-03 |
| *EPHB4* | *DFNB59* | 0.36 | 3.56E-03 |
| *EPHB4* | *DGCR2* | 0.34 | 6.47E-03 |
| *EPHB4* | *DGKD* | 0.37 | 3.51E-03 |
| *EPHB4* | *DGKE* | 0.30 | 1.75E-02 |
| *EPHB4* | *DGKZ* | 0.35 | 5.51E-03 |
| *EPHB4* | *DHCR7* | 0.31 | 1.51E-02 |
| *EPHB4* | *DHDH* | 0.31 | 1.42E-02 |
| *EPHB4* | *DHH* | 0.35 | 5.15E-03 |
| *EPHB4* | *DHRS13* | 0.44 | 3.88E-04 |
| *EPHB4* | *DHRSX* | 0.33 | 8.70E-03 |
| *EPHB4* | *DHX15* | 0.31 | 1.59E-02 |
| *EPHB4* | *DHX32* | 0.30 | 1.69E-02 |
| *EPHB4* | *DHX34* | 0.42 | 7.02E-04 |
| *EPHB4* | *DHX35* | 0.31 | 1.53E-02 |
| *EPHB4* | *DHX37* | 0.44 | 3.82E-04 |
| *EPHB4* | *DHX38* | 0.30 | 1.59E-02 |
| *EPHB4* | *DHX57* | 0.50 | 3.55E-05 |
| *EPHB4* | *DICER1* | 0.38 | 2.03E-03 |
| *EPHB4* | *DICER1-AS1* | 0.44 | 3.98E-04 |
| *EPHB4* | *DIO3* | 0.37 | 3.29E-03 |
| *EPHB4* | *DIS3L* | 0.37 | 2.73E-03 |
| *EPHB4* | *DIS3L2* | 0.42 | 6.58E-04 |
| *EPHB4* | *DISC1* | 0.44 | 3.11E-04 |
| *EPHB4* | *DKK3* | 0.35 | 5.57E-03 |
| *EPHB4* | *DKKL1* | 0.38 | 2.19E-03 |
| *EPHB4* | *DLAT* | -0.32 | 1.00E-02 |
| *EPHB4* | *DLG5* | 0.38 | 2.18E-03 |
| *EPHB4* | *DLL1* | 0.57 | 1.05E-06 |
| *EPHB4* | *DLX3* | 0.38 | 2.30E-03 |
| *EPHB4* | *DLX4* | 0.46 | 1.62E-04 |
| *EPHB4* | *DMAP1* | 0.44 | 3.55E-04 |
| *EPHB4* | *DMPK* | 0.41 | 9.40E-04 |
| *EPHB4* | *DMTF1* | 0.45 | 2.41E-04 |
| *EPHB4* | *DMWD* | 0.50 | 3.22E-05 |
| *EPHB4* | *DNAH10* | 0.36 | 3.93E-03 |
| *EPHB4* | *DNAH17* | 0.36 | 3.65E-03 |
| *EPHB4* | *DNAH2* | 0.34 | 6.04E-03 |
| *EPHB4* | *DNAH6* | 0.39 | 1.58E-03 |
| *EPHB4* | *DNAJB5-AS1* | 0.42 | 7.61E-04 |
| *EPHB4* | *DNAJC16* | 0.40 | 1.30E-03 |
| *EPHB4* | *DNAJC27* | 0.36 | 4.50E-03 |
| *EPHB4* | *DNAJC28* | 0.34 | 6.16E-03 |
| *EPHB4* | *DNASE1* | 0.33 | 7.81E-03 |
| *EPHB4* | *DNASE2* | 0.40 | 1.15E-03 |
| *EPHB4* | *DNM1L* | 0.36 | 4.29E-03 |
| *EPHB4* | *DNM1P35* | 0.33 | 7.89E-03 |
| *EPHB4* | *DOCK1* | 0.48 | 6.77E-05 |
| *EPHB4* | *DOCK7* | 0.41 | 9.47E-04 |
| *EPHB4* | *DOPEY1* | 0.32 | 1.12E-02 |
| *EPHB4* | *DPH3* | -0.38 | 2.42E-03 |
| *EPHB4* | *DPP9* | 0.35 | 4.73E-03 |
| *EPHB4* | *DPY19L1* | 0.42 | 6.61E-04 |
| *EPHB4* | *DPYSL2* | 0.30 | 1.68E-02 |
| *EPHB4* | *DRC3* | 0.32 | 1.20E-02 |
| *EPHB4* | *DRD4* | 0.38 | 2.67E-03 |
| *EPHB4* | *DROSHA* | 0.33 | 7.90E-03 |
| *EPHB4* | *DTX4* | 0.33 | 8.87E-03 |
| *EPHB4* | *DUSP16* | 0.31 | 1.30E-02 |
| *EPHB4* | *DVL2* | 0.30 | 1.72E-02 |
| *EPHB4* | *DYNC1H1* | 0.37 | 2.75E-03 |
| *EPHB4* | *DYNC1I1* | 0.41 | 9.93E-04 |
| *EPHB4* | *DYRK2* | 0.50 | 3.69E-05 |
| *EPHB4* | *DZANK1* | 0.43 | 4.73E-04 |
| *EPHB4* | *E2F3* | 0.32 | 1.09E-02 |
| *EPHB4* | *E4F1* | 0.41 | 1.09E-03 |
| *EPHB4* | *ECE1* | 0.45 | 2.19E-04 |
| *EPHB4* | *ECHDC2* | 0.32 | 1.18E-02 |
| *EPHB4* | *EDARADD* | 0.33 | 7.98E-03 |
| *EPHB4* | *EDC4* | 0.34 | 6.28E-03 |
| *EPHB4* | *EDNRA* | 0.40 | 1.47E-03 |
| *EPHB4* | *EEFSEC* | 0.36 | 4.34E-03 |
| *EPHB4* | *EFNA4* | 0.36 | 3.99E-03 |
| *EPHB4* | *EFNB1* | 0.34 | 6.04E-03 |
| *EPHB4* | *EFS* | 0.36 | 4.20E-03 |
| *EPHB4* | *EGFL8* | 0.31 | 1.34E-02 |
| *EPHB4* | *EGFR* | 0.32 | 1.21E-02 |
| *EPHB4* | *EGR2* | 0.34 | 7.45E-03 |
| *EPHB4* | *EHD3* | 0.34 | 7.13E-03 |
| *EPHB4* | *EHHADH* | 0.34 | 6.94E-03 |
| *EPHB4* | *EHMT1* | 0.36 | 4.20E-03 |
| *EPHB4* | *EHMT2* | 0.47 | 1.02E-04 |
| *EPHB4* | *EIF2AK4* | 0.31 | 1.52E-02 |
| *EPHB4* | *EIF3E* | -0.37 | 3.44E-03 |
| *EPHB4* | *EIF4ENIF1* | 0.38 | 2.19E-03 |
| *EPHB4* | *EIF4G3* | 0.41 | 8.83E-04 |
| *EPHB4* | *ELMO2* | 0.41 | 9.13E-04 |
| *EPHB4* | *ELMSAN1* | 0.49 | 5.33E-05 |
| *EPHB4* | *EMC1* | 0.51 | 2.59E-05 |
| *EPHB4* | *EMC10* | 0.32 | 1.25E-02 |
| *EPHB4* | *EMC2* | -0.37 | 3.13E-03 |
| *EPHB4* | *EML2* | 0.31 | 1.47E-02 |
| *EPHB4* | *ENDOG* | 0.32 | 1.26E-02 |
| *EPHB4* | *ENDOV* | 0.40 | 1.33E-03 |
| *EPHB4* | *ENGASE* | 0.49 | 4.85E-05 |
| *EPHB4* | *ENKUR* | 0.34 | 7.00E-03 |
| *EPHB4* | *ENO1-AS1* | 0.35 | 5.78E-03 |
| *EPHB4* | *ENO3* | 0.33 | 7.87E-03 |
| *EPHB4* | *ENPP2* | 0.31 | 1.45E-02 |
| *EPHB4* | *ENPP5* | 0.33 | 8.38E-03 |
| *EPHB4* | *ENTHD2* | 0.33 | 7.86E-03 |
| *EPHB4* | *ENTPD5* | 0.36 | 4.17E-03 |
| *EPHB4* | *ENTPD6* | 0.42 | 6.61E-04 |
| *EPHB4* | *EP300* | 0.40 | 1.33E-03 |
| *EPHB4* | *EP400* | 0.49 | 4.52E-05 |
| *EPHB4* | *EP400NL* | 0.40 | 1.25E-03 |
| *EPHB4* | *EPB41* | 0.39 | 1.89E-03 |
| *EPHB4* | *EPB41L4A* | 0.32 | 1.22E-02 |
| *EPHB4* | *EPB41L4A-AS2* | 0.38 | 2.41E-03 |
| *EPHB4* | *EPC1* | 0.31 | 1.43E-02 |
| *EPHB4* | *EPHA7* | 0.38 | 2.46E-03 |
| *EPHB4* | *EPHB3* | 0.31 | 1.44E-02 |
| *EPHB4* | *EPHB6* | 0.31 | 1.52E-02 |
| *EPHB4* | *ERC1* | 0.42 | 7.27E-04 |
| *EPHB4* | *ERCC2* | 0.39 | 1.61E-03 |
| *EPHB4* | *ERCC6* | 0.30 | 1.63E-02 |
| *EPHB4* | *ERCC6L2* | 0.32 | 1.10E-02 |
| *EPHB4* | *ERF* | 0.44 | 3.64E-04 |
| *EPHB4* | *ERLIN2* | 0.30 | 1.70E-02 |
| *EPHB4* | *ERN1* | 0.32 | 1.23E-02 |
| *EPHB4* | *ERP29* | 0.37 | 2.79E-03 |
| *EPHB4* | *ESPN* | 0.30 | 1.68E-02 |
| *EPHB4* | *ETV6* | 0.40 | 1.46E-03 |
| *EPHB4* | *EVC2* | 0.42 | 6.75E-04 |
| *EPHB4* | *EVPLL* | 0.32 | 1.10E-02 |
| *EPHB4* | *EXD3* | 0.32 | 1.20E-02 |
| *EPHB4* | *EXO5* | 0.38 | 2.36E-03 |
| *EPHB4* | *EXOC4* | 0.31 | 1.41E-02 |
| *EPHB4* | *EXOC7* | 0.42 | 5.83E-04 |
| *EPHB4* | *EXOSC10* | 0.31 | 1.31E-02 |
| *EPHB4* | *EXTL3* | 0.35 | 4.78E-03 |
| *EPHB4* | *FAAH* | 0.33 | 9.08E-03 |
| *EPHB4* | *FAAP100* | 0.45 | 2.86E-04 |
| *EPHB4* | *FAAP20* | 0.40 | 1.11E-03 |
| *EPHB4* | *FABP3* | -0.33 | 8.46E-03 |
| *EPHB4* | *FADD* | 0.31 | 1.43E-02 |
| *EPHB4* | *FAM102B* | 0.44 | 3.27E-04 |
| *EPHB4* | *FAM109A* | 0.40 | 1.47E-03 |
| *EPHB4* | *FAM110D* | 0.37 | 3.17E-03 |
| *EPHB4* | *FAM111A* | 0.38 | 2.21E-03 |
| *EPHB4* | *FAM117A* | 0.46 | 1.99E-04 |
| *EPHB4* | *FAM120B* | 0.37 | 3.34E-03 |
| *EPHB4* | *FAM122C* | 0.33 | 8.14E-03 |
| *EPHB4* | *FAM131A* | 0.37 | 3.13E-03 |
| *EPHB4* | *FAM133B* | 0.35 | 5.82E-03 |
| *EPHB4* | *FAM134C* | 0.41 | 8.86E-04 |
| *EPHB4* | *FAM157C* | 0.41 | 9.16E-04 |
| *EPHB4* | *FAM160A1* | 0.37 | 2.89E-03 |
| *EPHB4* | *FAM160A2* | 0.43 | 4.70E-04 |
| *EPHB4* | *FAM160B2* | 0.41 | 9.37E-04 |
| *EPHB4* | *FAM167A* | 0.40 | 1.45E-03 |
| *EPHB4* | *FAM172A* | 0.30 | 1.60E-02 |
| *EPHB4* | *FAM183A* | 0.32 | 1.12E-02 |
| *EPHB4* | *FAM185A* | 0.46 | 1.48E-04 |
| *EPHB4* | *FAM19A5* | 0.31 | 1.32E-02 |
| *EPHB4* | *FAM20C* | 0.32 | 1.24E-02 |
| *EPHB4* | *FAM215B* | 0.39 | 1.60E-03 |
| *EPHB4* | *FAM217B* | 0.36 | 3.85E-03 |
| *EPHB4* | *FAM21A* | 0.36 | 4.24E-03 |
| *EPHB4* | *FAM21C* | 0.31 | 1.37E-02 |
| *EPHB4* | *FAM222B* | 0.49 | 6.23E-05 |
| *EPHB4* | *FAM227A* | 0.50 | 4.00E-05 |
| *EPHB4* | *FAM3C* | 0.33 | 8.98E-03 |
| *EPHB4* | *FAM53A* | 0.52 | 1.42E-05 |
| *EPHB4* | *FAM53B* | 0.41 | 9.23E-04 |
| *EPHB4* | *FAM60A* | 0.31 | 1.45E-02 |
| *EPHB4* | *FAM65C* | 0.33 | 9.67E-03 |
| *EPHB4* | *FAM66A* | 0.46 | 1.80E-04 |
| *EPHB4* | *FAM66B* | 0.42 | 7.51E-04 |
| *EPHB4* | *FAM66C* | 0.36 | 3.99E-03 |
| *EPHB4* | *FAM71E1* | 0.37 | 2.78E-03 |
| *EPHB4* | *FAM71F2* | 0.32 | 1.14E-02 |
| *EPHB4* | *FAM72D* | 0.31 | 1.29E-02 |
| *EPHB4* | *FAM73B* | 0.33 | 9.79E-03 |
| *EPHB4* | *FAM76A* | 0.30 | 1.72E-02 |
| *EPHB4* | *FAM83G* | 0.31 | 1.35E-02 |
| *EPHB4* | *FANCA* | 0.33 | 9.40E-03 |
| *EPHB4* | *FARP2* | 0.31 | 1.30E-02 |
| *EPHB4* | *FASTKD2* | 0.36 | 4.23E-03 |
| *EPHB4* | *FATE1* | 0.34 | 6.79E-03 |
| *EPHB4* | *FAXDC2* | 0.30 | 1.63E-02 |
| *EPHB4* | *FBF1* | 0.49 | 5.21E-05 |
| *EPHB4* | *FBLIM1* | 0.33 | 8.97E-03 |
| *EPHB4* | *FBRS* | 0.38 | 2.38E-03 |
| *EPHB4* | *FBRSL1* | 0.40 | 1.26E-03 |
| *EPHB4* | *FBXL18* | 0.31 | 1.39E-02 |
| *EPHB4* | *FBXL19* | 0.38 | 2.11E-03 |
| *EPHB4* | *FBXL19-AS1* | 0.44 | 3.29E-04 |
| *EPHB4* | *FBXO21* | 0.31 | 1.37E-02 |
| *EPHB4* | *FBXO31* | 0.34 | 7.65E-03 |
| *EPHB4* | *FBXO36* | 0.40 | 1.38E-03 |
| *EPHB4* | *FBXO42* | 0.36 | 3.59E-03 |
| *EPHB4* | *FBXO44* | 0.44 | 3.26E-04 |
| *EPHB4* | *FBXW4* | 0.34 | 6.40E-03 |
| *EPHB4* | *FBXW7* | 0.43 | 4.28E-04 |
| *EPHB4* | *FBXW8* | 0.35 | 5.20E-03 |
| *EPHB4* | *FCGBP* | 0.48 | 7.53E-05 |
| *EPHB4* | *FDX1L* | 0.35 | 5.10E-03 |
| *EPHB4* | *FDXR* | 0.36 | 4.48E-03 |
| *EPHB4* | *FGD1* | 0.30 | 1.67E-02 |
| *EPHB4* | *FGD5* | 0.43 | 4.32E-04 |
| *EPHB4* | *FGD6* | 0.46 | 1.55E-04 |
| *EPHB4* | *FGFBP3* | 0.33 | 8.25E-03 |
| *EPHB4* | *FGFR2* | 0.36 | 3.99E-03 |
| *EPHB4* | *FGFR3* | 0.52 | 1.67E-05 |
| *EPHB4* | *FGFRL1* | 0.31 | 1.56E-02 |
| *EPHB4* | *FHDC1* | 0.45 | 2.45E-04 |
| *EPHB4* | *FHOD1* | 0.33 | 8.99E-03 |
| *EPHB4* | *FKBP14* | 0.40 | 1.20E-03 |
| *EPHB4* | *FKBP9* | 0.42 | 5.81E-04 |
| *EPHB4* | *FLJ31104* | 0.52 | 1.68E-05 |
| *EPHB4* | *FLJ35934* | 0.44 | 3.83E-04 |
| *EPHB4* | *FLNA* | 0.36 | 3.87E-03 |
| *EPHB4* | *FLOT2* | 0.40 | 1.19E-03 |
| *EPHB4* | *FMNL2* | 0.32 | 1.17E-02 |
| *EPHB4* | *FMNL3* | 0.49 | 5.63E-05 |
| *EPHB4* | *FMO1* | 0.30 | 1.60E-02 |
| *EPHB4* | *FNBP4* | 0.38 | 2.40E-03 |
| *EPHB4* | *FNDC3B* | 0.46 | 1.58E-04 |
| *EPHB4* | *FNTB* | 0.34 | 7.27E-03 |
| *EPHB4* | *FOXD3-AS1* | 0.34 | 7.59E-03 |
| *EPHB4* | *FOXJ3* | 0.39 | 1.72E-03 |
| *EPHB4* | *FOXK2* | 0.35 | 5.29E-03 |
| *EPHB4* | *FOXO3* | 0.30 | 1.66E-02 |
| *EPHB4* | *FOXO6* | 0.45 | 2.78E-04 |
| *EPHB4* | *FOXRED2* | 0.36 | 3.54E-03 |
| *EPHB4* | *FRAS1* | 0.33 | 8.95E-03 |
| *EPHB4* | *FRMD3* | 0.34 | 6.56E-03 |
| *EPHB4* | *FRMD4A* | 0.33 | 8.55E-03 |
| *EPHB4* | *FRS2* | 0.31 | 1.50E-02 |
| *EPHB4* | *FST* | 0.39 | 1.65E-03 |
| *EPHB4* | *FTX* | 0.33 | 8.94E-03 |
| *EPHB4* | *FUK* | 0.32 | 1.11E-02 |
| *EPHB4* | *FUT1* | 0.31 | 1.47E-02 |
| *EPHB4* | *FUZ* | 0.31 | 1.38E-02 |
| *EPHB4* | *FXR2* | 0.32 | 1.10E-02 |
| *EPHB4* | *FZD1* | 0.43 | 5.06E-04 |
| *EPHB4* | *FZD2* | 0.41 | 9.25E-04 |
| *EPHB4* | *FZD8* | 0.30 | 1.70E-02 |
| *EPHB4* | *GAA* | 0.42 | 6.88E-04 |
| *EPHB4* | *GAL3ST4* | 0.46 | 1.99E-04 |
| *EPHB4* | *GALNT14* | 0.34 | 6.80E-03 |
| *EPHB4* | *GALNT18* | 0.43 | 4.88E-04 |
| *EPHB4* | *GALNT6* | 0.31 | 1.27E-02 |
| *EPHB4* | *GANAB* | 0.32 | 1.08E-02 |
| *EPHB4* | *GARNL3* | 0.50 | 4.16E-05 |
| *EPHB4* | *GATAD2B* | 0.43 | 5.04E-04 |
| *EPHB4* | *GATS* | 0.30 | 1.76E-02 |
| *EPHB4* | *GATSL3* | 0.33 | 9.78E-03 |
| *EPHB4* | *GBF1* | 0.35 | 4.68E-03 |
| *EPHB4* | *GCDH* | 0.32 | 1.07E-02 |
| *EPHB4* | *GCN1* | 0.44 | 3.58E-04 |
| *EPHB4* | *GCSAM* | 0.33 | 8.32E-03 |
| *EPHB4* | *GDF11* | 0.35 | 4.73E-03 |
| *EPHB4* | *GEN1* | 0.32 | 1.18E-02 |
| *EPHB4* | *GGA1* | 0.36 | 4.18E-03 |
| *EPHB4* | *GGA2* | 0.31 | 1.32E-02 |
| *EPHB4* | *GGA3* | 0.32 | 1.09E-02 |
| *EPHB4* | *GIGYF1* | 0.59 | 4.07E-07 |
| *EPHB4* | *GIT1* | 0.45 | 2.43E-04 |
| *EPHB4* | *GIT2* | 0.33 | 7.98E-03 |
| *EPHB4* | *GK5* | 0.46 | 1.93E-04 |
| *EPHB4* | *GLDN* | 0.32 | 1.07E-02 |
| *EPHB4* | *GLI1* | 0.33 | 8.00E-03 |
| *EPHB4* | *GLI3* | 0.41 | 9.21E-04 |
| *EPHB4* | *GLIPR1L2* | 0.36 | 4.59E-03 |
| *EPHB4* | *GLRX2* | -0.32 | 1.13E-02 |
| *EPHB4* | *GLTSCR1* | 0.30 | 1.61E-02 |
| *EPHB4* | *GLTSCR1L* | 0.37 | 3.51E-03 |
| *EPHB4* | *GNA11* | 0.35 | 5.54E-03 |
| *EPHB4* | *GNAZ* | 0.45 | 2.83E-04 |
| *EPHB4* | *GNB2* | 0.35 | 5.79E-03 |
| *EPHB4* | *GNRH1* | 0.36 | 3.63E-03 |
| *EPHB4* | *GNS* | 0.34 | 6.84E-03 |
| *EPHB4* | *GOLGA3* | 0.39 | 1.82E-03 |
| *EPHB4* | *GOLGA6L4* | 0.32 | 1.08E-02 |
| *EPHB4* | *GOLGA8A* | 0.40 | 1.28E-03 |
| *EPHB4* | *GOLGA8B* | 0.31 | 1.52E-02 |
| *EPHB4* | *GOLGB1* | 0.38 | 2.24E-03 |
| *EPHB4* | *GOPC* | 0.39 | 1.61E-03 |
| *EPHB4* | *GPAT4* | 0.36 | 4.12E-03 |
| *EPHB4* | *GPATCH2* | 0.32 | 1.13E-02 |
| *EPHB4* | *GPATCH2L* | 0.30 | 1.76E-02 |
| *EPHB4* | *GPATCH8* | 0.39 | 1.88E-03 |
| *EPHB4* | *GPC1* | 0.33 | 8.49E-03 |
| *EPHB4* | *GPC2* | 0.46 | 1.85E-04 |
| *EPHB4* | *GPR135* | 0.40 | 1.31E-03 |
| *EPHB4* | *GPR158* | 0.32 | 1.09E-02 |
| *EPHB4* | *GPR161* | 0.35 | 4.76E-03 |
| *EPHB4* | *GPR3* | 0.35 | 5.03E-03 |
| *EPHB4* | *GPRASP1* | 0.39 | 1.84E-03 |
| *EPHB4* | *GPRIN2* | 0.30 | 1.72E-02 |
| *EPHB4* | *GPSM1* | 0.38 | 2.29E-03 |
| *EPHB4* | *GRAMD2* | 0.32 | 1.09E-02 |
| *EPHB4* | *GRAMD4* | 0.36 | 3.92E-03 |
| *EPHB4* | *GRASP* | 0.36 | 3.77E-03 |
| *EPHB4* | *GRB10* | 0.36 | 3.54E-03 |
| *EPHB4* | *GRIP1* | 0.42 | 7.58E-04 |
| *EPHB4* | *GRN* | 0.30 | 1.78E-02 |
| *EPHB4* | *GS1-124K5.11* | 0.34 | 6.28E-03 |
| *EPHB4* | *GS1-124K5.3* | 0.40 | 1.44E-03 |
| *EPHB4* | *GS1-124K5.4* | 0.33 | 7.94E-03 |
| *EPHB4* | *GSK3A* | 0.40 | 1.18E-03 |
| *EPHB4* | *GSK3B* | 0.35 | 5.32E-03 |
| *EPHB4* | *GSPT2* | 0.36 | 3.73E-03 |
| *EPHB4* | *GTF2I* | 0.41 | 9.79E-04 |
| *EPHB4* | *GTF2IRD1* | 0.39 | 1.92E-03 |
| *EPHB4* | *GTF2IRD2* | 0.35 | 5.65E-03 |
| *EPHB4* | *GTF3C1* | 0.34 | 7.06E-03 |
| *EPHB4* | *GTF3C2* | 0.40 | 1.14E-03 |
| *EPHB4* | *GUCY1B3* | 0.33 | 9.07E-03 |
| *EPHB4* | *GUCY2D* | 0.33 | 8.63E-03 |
| *EPHB4* | *GYG2* | 0.38 | 2.35E-03 |
| *EPHB4* | *GYLTL1B* | 0.33 | 9.56E-03 |
| *EPHB4* | *GYS1* | 0.40 | 1.10E-03 |
| *EPHB4* | *H2AFY2* | 0.32 | 1.07E-02 |
| *EPHB4* | *H6PD* | 0.42 | 6.89E-04 |
| *EPHB4* | *HABP4* | 0.31 | 1.45E-02 |
| *EPHB4* | *HAPLN1* | 0.33 | 9.06E-03 |
| *EPHB4* | *HBP1* | 0.34 | 7.13E-03 |
| *EPHB4* | *HCAR3* | 0.36 | 3.76E-03 |
| *EPHB4* | *HCFC1* | 0.41 | 9.81E-04 |
| *EPHB4* | *HCFC2* | 0.44 | 3.02E-04 |
| *EPHB4* | *HCG11* | 0.33 | 8.02E-03 |
| *EPHB4* | *HCN3* | 0.40 | 1.39E-03 |
| *EPHB4* | *HDAC4* | 0.42 | 7.01E-04 |
| *EPHB4* | *HDAC5* | 0.30 | 1.61E-02 |
| *EPHB4* | *HDAC6* | 0.37 | 2.85E-03 |
| *EPHB4* | *HDAC7* | 0.46 | 1.52E-04 |
| *EPHB4* | *HEATR5B* | 0.34 | 7.32E-03 |
| *EPHB4* | *HECTD2* | 0.33 | 8.18E-03 |
| *EPHB4* | *HECTD3* | 0.31 | 1.54E-02 |
| *EPHB4* | *HECW2* | 0.31 | 1.27E-02 |
| *EPHB4* | *HELB* | 0.32 | 1.09E-02 |
| *EPHB4* | *HEXIM1* | 0.33 | 8.00E-03 |
| *EPHB4* | *HGS* | 0.33 | 8.80E-03 |
| *EPHB4* | *HGSNAT* | 0.31 | 1.50E-02 |
| *EPHB4* | *HIP1* | 0.47 | 1.33E-04 |
| *EPHB4* | *HIPK2* | 0.30 | 1.64E-02 |
| *EPHB4* | *HIST1H2AB* | 0.36 | 4.26E-03 |
| *EPHB4* | *HIST1H2AK* | 0.37 | 2.84E-03 |
| *EPHB4* | *HIST1H2AM* | 0.34 | 7.17E-03 |
| *EPHB4* | *HIST1H2BB* | 0.43 | 4.75E-04 |
| *EPHB4* | *HIST1H2BE* | 0.40 | 1.33E-03 |
| *EPHB4* | *HIST1H2BF* | 0.34 | 7.37E-03 |
| *EPHB4* | *HIST1H2BG* | 0.33 | 8.24E-03 |
| *EPHB4* | *HIST1H2BH* | 0.38 | 2.02E-03 |
| *EPHB4* | *HIST1H2BI* | 0.31 | 1.35E-02 |
| *EPHB4* | *HIST1H2BL* | 0.45 | 2.13E-04 |
| *EPHB4* | *HIST1H2BN* | 0.40 | 1.20E-03 |
| *EPHB4* | *HIST1H3A* | 0.37 | 3.44E-03 |
| *EPHB4* | *HIST1H3E* | 0.46 | 1.65E-04 |
| *EPHB4* | *HIST1H3F* | 0.35 | 5.87E-03 |
| *EPHB4* | *HIST1H3J* | 0.51 | 2.24E-05 |
| *EPHB4* | *HIST1H4B* | 0.33 | 8.96E-03 |
| *EPHB4* | *HIST1H4D* | 0.33 | 8.86E-03 |
| *EPHB4* | *HIST1H4E* | 0.43 | 4.34E-04 |
| *EPHB4* | *HIST1H4J* | 0.37 | 2.99E-03 |
| *EPHB4* | *HIST1H4L* | 0.30 | 1.71E-02 |
| *EPHB4* | *HIST2H2BF* | 0.32 | 1.22E-02 |
| *EPHB4* | *HIST2H3D* | 0.31 | 1.48E-02 |
| *EPHB4* | *HIST4H4* | 0.52 | 1.26E-05 |
| *EPHB4* | *HIVEP1* | 0.35 | 4.99E-03 |
| *EPHB4* | *HIVEP3* | 0.44 | 3.93E-04 |
| *EPHB4* | *HLA-DOA* | 0.34 | 6.68E-03 |
| *EPHB4* | *HMCN1* | 0.36 | 4.16E-03 |
| *EPHB4* | *HMGXB4* | 0.32 | 1.21E-02 |
| *EPHB4* | *HNRNPA1L2* | 0.42 | 6.32E-04 |
| *EPHB4* | *HNRNPH1* | 0.31 | 1.50E-02 |
| *EPHB4* | *HNRNPLL* | 0.33 | 8.95E-03 |
| *EPHB4* | *HOOK2* | 0.30 | 1.68E-02 |
| *EPHB4* | *HOXA6* | 0.40 | 1.22E-03 |
| *EPHB4* | *HOXC-AS1* | 0.42 | 7.44E-04 |
| *EPHB4* | *HOXC11* | 0.39 | 1.91E-03 |
| *EPHB4* | *HOXC13* | 0.31 | 1.34E-02 |
| *EPHB4* | *HOXC13-AS* | 0.34 | 7.58E-03 |
| *EPHB4* | *HOXC6* | 0.37 | 2.81E-03 |
| *EPHB4* | *HOXD9* | 0.33 | 8.85E-03 |
| *EPHB4* | *HP1BP3* | 0.37 | 2.74E-03 |
| *EPHB4* | *HR* | 0.34 | 7.59E-03 |
| *EPHB4* | *HRC* | 0.32 | 1.07E-02 |
| *EPHB4* | *HS6ST1* | 0.47 | 1.20E-04 |
| *EPHB4* | *HSF2* | 0.32 | 1.00E-02 |
| *EPHB4* | *HSPA2* | 0.34 | 7.39E-03 |
| *EPHB4* | *HSPBAP1* | 0.34 | 6.89E-03 |
| *EPHB4* | *HSPG2* | 0.39 | 1.90E-03 |
| *EPHB4* | *HUNK* | 0.31 | 1.36E-02 |
| *EPHB4* | *HUWE1* | 0.33 | 7.83E-03 |
| *EPHB4* | *HYDIN* | 0.34 | 7.01E-03 |
| *EPHB4* | *IER5L* | 0.35 | 4.88E-03 |
| *EPHB4* | *IFFO1* | 0.33 | 8.76E-03 |
| *EPHB4* | *IFITM10* | 0.47 | 9.91E-05 |
| *EPHB4* | *IFNLR1* | 0.36 | 4.05E-03 |
| *EPHB4* | *IFT122* | 0.32 | 1.10E-02 |
| *EPHB4* | *IFT140* | 0.39 | 1.94E-03 |
| *EPHB4* | *IFT43* | 0.33 | 9.17E-03 |
| *EPHB4* | *IFT81* | 0.42 | 6.62E-04 |
| *EPHB4* | *IFT88* | 0.32 | 1.07E-02 |
| *EPHB4* | *IGF2R* | 0.37 | 3.07E-03 |
| *EPHB4* | *IGFBP4* | 0.32 | 1.11E-02 |
| *EPHB4* | *IGHMBP2* | 0.32 | 1.03E-02 |
| *EPHB4* | *IGSF9* | 0.36 | 3.62E-03 |
| *EPHB4* | *IKBKAP* | 0.34 | 6.52E-03 |
| *EPHB4* | *IKBKB* | 0.35 | 4.69E-03 |
| *EPHB4* | *IL16* | 0.33 | 8.89E-03 |
| *EPHB4* | *IL17RD* | 0.34 | 6.67E-03 |
| *EPHB4* | *IL20* | 0.44 | 3.23E-04 |
| *EPHB4* | *IL27RA* | 0.38 | 2.29E-03 |
| *EPHB4* | *IL2RB* | 0.35 | 5.15E-03 |
| *EPHB4* | *ILF3* | 0.35 | 6.01E-03 |
| *EPHB4* | *INADL* | 0.34 | 7.14E-03 |
| *EPHB4* | *INF2* | 0.34 | 6.14E-03 |
| *EPHB4* | *ING4* | 0.42 | 6.56E-04 |
| *EPHB4* | *ING5* | 0.36 | 3.67E-03 |
| *EPHB4* | *INMT* | 0.34 | 7.61E-03 |
| *EPHB4* | *INO80D* | 0.33 | 9.15E-03 |
| *EPHB4* | *INPP5E* | 0.48 | 6.48E-05 |
| *EPHB4* | *INTS1* | 0.52 | 1.23E-05 |
| *EPHB4* | *INTS2* | 0.34 | 6.14E-03 |
| *EPHB4* | *INTS6* | 0.38 | 2.64E-03 |
| *EPHB4* | *INVS* | 0.30 | 1.63E-02 |
| *EPHB4* | *IPO13* | 0.33 | 8.69E-03 |
| *EPHB4* | *IQCA1* | 0.31 | 1.34E-02 |
| *EPHB4* | *IQCE* | 0.44 | 3.64E-04 |
| *EPHB4* | *IRF2BP1* | 0.37 | 3.44E-03 |
| *EPHB4* | *IRF2BPL* | 0.45 | 2.67E-04 |
| *EPHB4* | *IRF3* | 0.44 | 3.13E-04 |
| *EPHB4* | *IRGQ* | 0.42 | 6.08E-04 |
| *EPHB4* | *IRX2* | 0.31 | 1.56E-02 |
| *EPHB4* | *IRX4* | 0.31 | 1.31E-02 |
| *EPHB4* | *IRX6* | 0.32 | 1.16E-02 |
| *EPHB4* | *ISLR2* | 0.30 | 1.65E-02 |
| *EPHB4* | *ISM1* | 0.42 | 6.89E-04 |
| *EPHB4* | *ITFG2* | 0.42 | 6.48E-04 |
| *EPHB4* | *ITGA10* | 0.38 | 2.25E-03 |
| *EPHB4* | *ITGA3* | 0.31 | 1.39E-02 |
| *EPHB4* | *ITGA7* | 0.53 | 8.70E-06 |
| *EPHB4* | *ITGA8* | 0.37 | 3.01E-03 |
| *EPHB4* | *ITGA9* | 0.32 | 1.00E-02 |
| *EPHB4* | *ITGAM* | 0.33 | 8.21E-03 |
| *EPHB4* | *ITGB4* | 0.43 | 5.47E-04 |
| *EPHB4* | *ITGB5* | 0.38 | 2.14E-03 |
| *EPHB4* | *ITPK1* | 0.35 | 5.83E-03 |
| *EPHB4* | *ITPR1-AS1* | 0.52 | 1.77E-05 |
| *EPHB4* | *ITPR3* | 0.34 | 6.49E-03 |
| *EPHB4* | *IZUMO1* | 0.46 | 1.72E-04 |
| *EPHB4* | *JAG2* | 0.57 | 1.64E-06 |
| *EPHB4* | *JDP2* | 0.43 | 5.70E-04 |
| *EPHB4* | *JMJD6* | 0.32 | 1.06E-02 |
| *EPHB4* | *JMJD7-PLA2G4B* | 0.42 | 5.86E-04 |
| *EPHB4* | *JMJD8* | 0.36 | 3.83E-03 |
| *EPHB4* | *JUNB* | 0.31 | 1.49E-02 |
| *EPHB4* | *KANSL1* | 0.39 | 1.88E-03 |
| *EPHB4* | *KANSL1L* | 0.38 | 2.37E-03 |
| *EPHB4* | *KANTR* | 0.32 | 1.13E-02 |
| *EPHB4* | *KAT2A* | 0.32 | 1.21E-02 |
| *EPHB4* | *KAT7* | 0.37 | 3.39E-03 |
| *EPHB4* | *KAT8* | 0.32 | 1.19E-02 |
| *EPHB4* | *KATNA1* | 0.35 | 5.73E-03 |
| *EPHB4* | *KB-1410C5.5* | 0.32 | 1.12E-02 |
| *EPHB4* | *KBTBD2* | 0.38 | 2.32E-03 |
| *EPHB4* | *KBTBD4* | 0.35 | 6.01E-03 |
| *EPHB4* | *KBTBD6* | 0.30 | 1.66E-02 |
| *EPHB4* | *KCNAB3* | 0.40 | 1.42E-03 |
| *EPHB4* | *KCNC3* | 0.41 | 8.90E-04 |
| *EPHB4* | *KCNC4* | 0.34 | 6.23E-03 |
| *EPHB4* | *KCNH8* | 0.37 | 2.85E-03 |
| *EPHB4* | *KCNQ5* | 0.32 | 1.20E-02 |
| *EPHB4* | *KCTD7* | 0.41 | 8.25E-04 |
| *EPHB4* | *KDF1* | 0.30 | 1.64E-02 |
| *EPHB4* | *KDM2B* | 0.62 | 8.28E-08 |
| *EPHB4* | *KDM4A* | 0.45 | 2.63E-04 |
| *EPHB4* | *KDM5A* | 0.40 | 1.45E-03 |
| *EPHB4* | *KDM5C* | 0.38 | 2.18E-03 |
| *EPHB4* | *KDM6B* | 0.31 | 1.40E-02 |
| *EPHB4* | *KDM8* | 0.30 | 1.72E-02 |
| *EPHB4* | *KIAA0100* | 0.47 | 1.08E-04 |
| *EPHB4* | *KIAA0226L* | 0.37 | 3.50E-03 |
| *EPHB4* | *KIAA0232* | 0.36 | 3.73E-03 |
| *EPHB4* | *KIAA0319L* | 0.36 | 4.20E-03 |
| *EPHB4* | *KIAA0930* | 0.32 | 1.25E-02 |
| *EPHB4* | *KIAA1109* | 0.33 | 8.98E-03 |
| *EPHB4* | *KIAA1217* | 0.31 | 1.42E-02 |
| *EPHB4* | *KIAA1456* | 0.31 | 1.48E-02 |
| *EPHB4* | *KIAA1462* | 0.34 | 7.57E-03 |
| *EPHB4* | *KIAA1549L* | 0.31 | 1.41E-02 |
| *EPHB4* | *KIAA1614* | 0.35 | 5.98E-03 |
| *EPHB4* | *KIAA1671* | 0.40 | 1.28E-03 |
| *EPHB4* | *KIAA1683* | 0.46 | 1.57E-04 |
| *EPHB4* | *KIAA1755* | 0.32 | 1.07E-02 |
| *EPHB4* | *KIAA1919* | 0.37 | 3.08E-03 |
| *EPHB4* | *KIAA2013* | 0.36 | 3.58E-03 |
| *EPHB4* | *KIF1B* | 0.44 | 3.20E-04 |
| *EPHB4* | *KIF26A* | 0.32 | 1.00E-02 |
| *EPHB4* | *KIF26B* | 0.33 | 8.95E-03 |
| *EPHB4* | *KIF27* | 0.39 | 1.86E-03 |
| *EPHB4* | *KL* | 0.36 | 4.58E-03 |
| *EPHB4* | *KLC4* | 0.31 | 1.47E-02 |
| *EPHB4* | *KLHL11* | 0.35 | 4.77E-03 |
| *EPHB4* | *KLHL15* | 0.34 | 6.38E-03 |
| *EPHB4* | *KLHL21* | 0.32 | 1.25E-02 |
| *EPHB4* | *KLHL26* | 0.42 | 6.31E-04 |
| *EPHB4* | *KLHL29* | 0.32 | 1.06E-02 |
| *EPHB4* | *KLLN* | 0.40 | 1.40E-03 |
| *EPHB4* | *KMT2D* | 0.36 | 3.72E-03 |
| *EPHB4* | *KMT2E* | 0.41 | 8.25E-04 |
| *EPHB4* | *KMT2E-AS1* | 0.48 | 7.69E-05 |
| *EPHB4* | *KMT5A* | 0.30 | 1.60E-02 |
| *EPHB4* | *KMT5C* | 0.34 | 6.67E-03 |
| *EPHB4* | *KNOP1* | 0.32 | 1.22E-02 |
| *EPHB4* | *KNTC1* | 0.39 | 1.60E-03 |
| *EPHB4* | *KPNA1* | 0.35 | 5.51E-03 |
| *EPHB4* | *KPTN* | 0.34 | 7.45E-03 |
| *EPHB4* | *KRBA2* | 0.31 | 1.42E-02 |
| *EPHB4* | *KRCC1* | 0.33 | 7.78E-03 |
| *EPHB4* | *KRIT1* | 0.43 | 4.51E-04 |
| *EPHB4* | *KRT9* | 0.32 | 1.24E-02 |
| *EPHB4* | *L3MBTL1* | 0.31 | 1.33E-02 |
| *EPHB4* | *L3MBTL2* | 0.36 | 4.07E-03 |
| *EPHB4* | *LA16c-306E5.2* | 0.35 | 4.90E-03 |
| *EPHB4* | *LAG3* | 0.59 | 3.82E-07 |
| *EPHB4* | *LAMA5* | 0.44 | 3.72E-04 |
| *EPHB4* | *LAMB1* | 0.31 | 1.42E-02 |
| *EPHB4* | *LAMB3* | 0.30 | 1.65E-02 |
| *EPHB4* | *LAMTOR4* | 0.35 | 4.98E-03 |
| *EPHB4* | *LANCL1* | 0.30 | 1.73E-02 |
| *EPHB4* | *LARP7* | 0.33 | 8.02E-03 |
| *EPHB4* | *LAT* | 0.44 | 3.09E-04 |
| *EPHB4* | *LATS1* | 0.36 | 4.60E-03 |
| *EPHB4* | *LBH* | 0.36 | 3.86E-03 |
| *EPHB4* | *LCAT* | 0.37 | 2.76E-03 |
| *EPHB4* | *LCLAT1* | 0.41 | 8.07E-04 |
| *EPHB4* | *LCMT1-AS1* | 0.36 | 4.47E-03 |
| *EPHB4* | *LDAH* | 0.30 | 1.69E-02 |
| *EPHB4* | *LDB1* | 0.33 | 8.61E-03 |
| *EPHB4* | *LDLRAP1* | 0.40 | 1.16E-03 |
| *EPHB4* | *LDOC1L* | 0.37 | 3.33E-03 |
| *EPHB4* | *LENG8* | 0.45 | 2.77E-04 |
| *EPHB4* | *LFNG* | 0.31 | 1.28E-02 |
| *EPHB4* | *LGALS9B* | 0.30 | 1.67E-02 |
| *EPHB4* | *LGALS9C* | 0.43 | 4.43E-04 |
| *EPHB4* | *LGR4* | 0.34 | 6.38E-03 |
| *EPHB4* | *LGR6* | 0.40 | 1.31E-03 |
| *EPHB4* | *LIFR* | 0.31 | 1.30E-02 |
| *EPHB4* | *LIG3* | 0.36 | 4.27E-03 |
| *EPHB4* | *LIMK2* | 0.31 | 1.42E-02 |
| *EPHB4* | *LIN7B* | 0.32 | 1.16E-02 |
| *EPHB4* | *LINC00174* | 0.40 | 1.46E-03 |
| *EPHB4* | *LINC00240* | 0.32 | 1.04E-02 |
| *EPHB4* | *LINC00243* | 0.30 | 1.67E-02 |
| *EPHB4* | *LINC00309* | 0.31 | 1.49E-02 |
| *EPHB4* | *LINC00310* | -0.34 | 6.48E-03 |
| *EPHB4* | *LINC00511* | 0.31 | 1.49E-02 |
| *EPHB4* | *LINC00526* | 0.34 | 7.70E-03 |
| *EPHB4* | *LINC00540* | 0.32 | 1.06E-02 |
| *EPHB4* | *LINC00853* | 0.33 | 8.25E-03 |
| *EPHB4* | *LINC00863* | 0.37 | 2.70E-03 |
| *EPHB4* | *LINC00894* | 0.32 | 1.20E-02 |
| *EPHB4* | *LINC00909* | 0.45 | 2.31E-04 |
| *EPHB4* | *LINC00959* | 0.31 | 1.56E-02 |
| *EPHB4* | *LINC00987* | 0.42 | 6.36E-04 |
| *EPHB4* | *LINC01001* | 0.48 | 7.83E-05 |
| *EPHB4* | *LINC01004* | 0.55 | 4.46E-06 |
| *EPHB4* | *LINC01089* | 0.37 | 2.73E-03 |
| *EPHB4* | *LINC01125* | 0.32 | 1.02E-02 |
| *EPHB4* | *LINC01136* | 0.35 | 5.01E-03 |
| *EPHB4* | *LINC01139* | 0.34 | 6.94E-03 |
| *EPHB4* | *LINC01176* | 0.39 | 1.64E-03 |
| *EPHB4* | *LINC01197* | 0.34 | 6.57E-03 |
| *EPHB4* | *LINC01270* | 0.31 | 1.43E-02 |
| *EPHB4* | *LINC01271* | 0.40 | 1.28E-03 |
| *EPHB4* | *LINC01376* | 0.43 | 4.42E-04 |
| *EPHB4* | *LINC01521* | 0.47 | 1.30E-04 |
| *EPHB4* | *LINC01550* | 0.32 | 1.03E-02 |
| *EPHB4* | *LINC01558* | 0.39 | 1.60E-03 |
| *EPHB4* | *LINC01572* | 0.33 | 8.33E-03 |
| *EPHB4* | *LIPE-AS1* | 0.36 | 4.12E-03 |
| *EPHB4* | *LLGL1* | 0.35 | 5.88E-03 |
| *EPHB4* | *LMF2* | 0.38 | 2.04E-03 |
| *EPHB4* | *LMLN* | 0.35 | 4.87E-03 |
| *EPHB4* | *LMNTD2* | 0.39 | 1.68E-03 |
| *EPHB4* | *LMO1* | 0.35 | 5.39E-03 |
| *EPHB4* | *LNP1* | 0.34 | 6.79E-03 |
| *EPHB4* | *LNPEP* | 0.41 | 8.43E-04 |
| *EPHB4* | *LOH12CR2* | 0.44 | 3.97E-04 |
| *EPHB4* | *LPCAT2* | 0.32 | 1.05E-02 |
| *EPHB4* | *LPCAT3* | 0.34 | 6.42E-03 |
| *EPHB4* | *LPIN3* | 0.30 | 1.60E-02 |
| *EPHB4* | *LRCH3* | 0.36 | 3.71E-03 |
| *EPHB4* | *LRCH4* | 0.47 | 1.14E-04 |
| *EPHB4* | *LRFN1* | 0.32 | 1.22E-02 |
| *EPHB4* | *LRFN3* | 0.43 | 4.14E-04 |
| *EPHB4* | *LRIG2* | 0.46 | 1.40E-04 |
| *EPHB4* | *LRIG3* | 0.45 | 2.21E-04 |
| *EPHB4* | *LRP1* | 0.40 | 1.21E-03 |
| *EPHB4* | *LRP11* | 0.34 | 6.32E-03 |
| *EPHB4* | *LRP2BP* | 0.41 | 9.68E-04 |
| *EPHB4* | *LRP5L* | 0.48 | 8.20E-05 |
| *EPHB4* | *LRP6* | 0.44 | 3.26E-04 |
| *EPHB4* | *LRRC16B* | 0.41 | 1.06E-03 |
| *EPHB4* | *LRRC23* | 0.34 | 6.58E-03 |
| *EPHB4* | *LRRC29* | 0.40 | 1.44E-03 |
| *EPHB4* | *LRRC32* | 0.38 | 2.40E-03 |
| *EPHB4* | *LRRC37A2* | 0.32 | 1.05E-02 |
| *EPHB4* | *LRRC37A3* | 0.31 | 1.31E-02 |
| *EPHB4* | *LRRC37B* | 0.47 | 1.39E-04 |
| *EPHB4* | *LRRC45* | 0.41 | 8.83E-04 |
| *EPHB4* | *LRRC58* | 0.34 | 7.48E-03 |
| *EPHB4* | *LRRC75A* | 0.41 | 8.17E-04 |
| *EPHB4* | *LRRC75B* | 0.41 | 1.09E-03 |
| *EPHB4* | *LRRC8D* | 0.37 | 2.88E-03 |
| *EPHB4* | *LRRTM2* | 0.32 | 1.03E-02 |
| *EPHB4* | *LRWD1* | 0.39 | 1.55E-03 |
| *EPHB4* | *LSM14B* | 0.39 | 1.94E-03 |
| *EPHB4* | *LSM3* | -0.52 | 1.62E-05 |
| *EPHB4* | *LSMEM1* | 0.38 | 2.51E-03 |
| *EPHB4* | *LTB4R* | 0.34 | 7.15E-03 |
| *EPHB4* | *LTBR* | 0.33 | 8.45E-03 |
| *EPHB4* | *LUC7L* | 0.36 | 4.52E-03 |
| *EPHB4* | *LUC7L3* | 0.31 | 1.35E-02 |
| *EPHB4* | *LUZP1* | 0.39 | 1.67E-03 |
| *EPHB4* | *LY6G5B* | 0.43 | 5.46E-04 |
| *EPHB4* | *LYVE1* | 0.32 | 1.26E-02 |
| *EPHB4* | *LZTR1* | 0.32 | 1.06E-02 |
| *EPHB4* | *LZTS2* | 0.31 | 1.28E-02 |
| *EPHB4* | *MADD* | 0.37 | 2.81E-03 |
| *EPHB4* | *MAFA* | 0.41 | 9.87E-04 |
| *EPHB4* | *MAFB* | 0.46 | 1.50E-04 |
| *EPHB4* | *MAFG-AS1* | 0.33 | 8.73E-03 |
| *EPHB4* | *MAGI2* | 0.34 | 6.91E-03 |
| *EPHB4* | *MAMLD1* | 0.33 | 8.28E-03 |
| *EPHB4* | *MAN2B2* | 0.35 | 5.59E-03 |
| *EPHB4* | *MANEA-AS1* | 0.33 | 8.88E-03 |
| *EPHB4* | *MAP1LC3B2* | 0.48 | 7.18E-05 |
| *EPHB4* | *MAP3K11* | 0.37 | 2.72E-03 |
| *EPHB4* | *MAP3K14* | 0.38 | 2.16E-03 |
| *EPHB4* | *MAP3K2* | 0.31 | 1.35E-02 |
| *EPHB4* | *MAP3K3* | 0.33 | 9.25E-03 |
| *EPHB4* | *MAP3K6* | 0.39 | 1.73E-03 |
| *EPHB4* | *MAP9* | 0.35 | 5.25E-03 |
| *EPHB4* | *MAPK11* | 0.40 | 1.13E-03 |
| *EPHB4* | *MAPK7* | 0.40 | 1.48E-03 |
| *EPHB4* | *MAPKAPK5* | 0.34 | 6.25E-03 |
| *EPHB4* | *MAPKAPK5-AS1* | 0.34 | 6.70E-03 |
| *EPHB4* | *MAPKBP1* | 0.38 | 2.61E-03 |
| *EPHB4* | *9-Mar* | 0.33 | 7.82E-03 |
| *EPHB4* | *MARCKSL1* | 0.37 | 3.24E-03 |
| *EPHB4* | *MARK4* | 0.34 | 7.21E-03 |
| *EPHB4* | *MAST2* | 0.35 | 4.93E-03 |
| *EPHB4* | *MAST3* | 0.36 | 3.98E-03 |
| *EPHB4* | *MATK* | -0.33 | 9.87E-03 |
| *EPHB4* | *MAU2* | 0.36 | 4.56E-03 |
| *EPHB4* | *MAVS* | 0.37 | 2.80E-03 |
| *EPHB4* | *MAZ* | 0.43 | 5.01E-04 |
| *EPHB4* | *MBD3* | 0.33 | 8.81E-03 |
| *EPHB4* | *MBTD1* | 0.40 | 1.15E-03 |
| *EPHB4* | *MCC* | 0.40 | 1.31E-03 |
| *EPHB4* | *MCF2L* | 0.32 | 1.01E-02 |
| *EPHB4* | *MCM7* | 0.44 | 3.66E-04 |
| *EPHB4* | *MDC1* | 0.38 | 2.64E-03 |
| *EPHB4* | *MDH1B* | 0.37 | 3.09E-03 |
| *EPHB4* | *MDM1* | 0.35 | 5.06E-03 |
| *EPHB4* | *MED11* | 0.31 | 1.53E-02 |
| *EPHB4* | *MED12* | 0.41 | 8.37E-04 |
| *EPHB4* | *MED12L* | 0.34 | 6.15E-03 |
| *EPHB4* | *MED13L* | 0.37 | 3.00E-03 |
| *EPHB4* | *MED23* | 0.36 | 4.15E-03 |
| *EPHB4* | *MED25* | 0.30 | 1.65E-02 |
| *EPHB4* | *MED26* | 0.35 | 5.67E-03 |
| *EPHB4* | *MEGF10* | 0.46 | 1.44E-04 |
| *EPHB4* | *MEGF6* | 0.60 | 2.45E-07 |
| *EPHB4* | *MEGF8* | 0.39 | 1.49E-03 |
| *EPHB4* | *MEOX1* | 0.39 | 1.81E-03 |
| *EPHB4* | *MEPCE* | 0.50 | 3.61E-05 |
| *EPHB4* | *METTL12* | 0.31 | 1.28E-02 |
| *EPHB4* | *METTL16* | 0.36 | 3.57E-03 |
| *EPHB4* | *METTL20* | 0.36 | 4.49E-03 |
| *EPHB4* | *METTL21A* | 0.36 | 3.97E-03 |
| *EPHB4* | *MEX3A* | 0.46 | 1.43E-04 |
| *EPHB4* | *MEX3C* | 0.32 | 1.06E-02 |
| *EPHB4* | *MFAP2* | 0.32 | 1.12E-02 |
| *EPHB4* | *MFHAS1* | 0.40 | 1.11E-03 |
| *EPHB4* | *MFN2* | 0.39 | 1.62E-03 |
| *EPHB4* | *MFSD11* | 0.34 | 6.72E-03 |
| *EPHB4* | *MFSD2A* | 0.38 | 2.03E-03 |
| *EPHB4* | *MGA* | 0.36 | 3.83E-03 |
| *EPHB4* | *MGAT5* | 0.32 | 1.01E-02 |
| *EPHB4* | *MGRN1* | 0.31 | 1.58E-02 |
| *EPHB4* | *MICAL1* | 0.35 | 5.06E-03 |
| *EPHB4* | *MICALL2* | 0.34 | 6.45E-03 |
| *EPHB4* | *MIIP* | 0.37 | 2.82E-03 |
| *EPHB4* | *MINK1* | 0.33 | 9.20E-03 |
| *EPHB4* | *MIR3142HG* | 0.37 | 2.90E-03 |
| *EPHB4* | *MIR762HG* | 0.32 | 1.20E-02 |
| *EPHB4* | *MKL1* | 0.37 | 2.93E-03 |
| *EPHB4* | *MKS1* | 0.39 | 1.68E-03 |
| *EPHB4* | *MLEC* | 0.43 | 4.77E-04 |
| *EPHB4* | *MLH3* | 0.42 | 6.81E-04 |
| *EPHB4* | *MLLT6* | 0.31 | 1.28E-02 |
| *EPHB4* | *MMP14* | 0.38 | 2.13E-03 |
| *EPHB4* | *MMP25-AS1* | 0.31 | 1.56E-02 |
| *EPHB4* | *MMRN2* | 0.41 | 9.53E-04 |
| *EPHB4* | *MOB3B* | 0.33 | 8.25E-03 |
| *EPHB4* | *MORN1* | 0.35 | 5.99E-03 |
| *EPHB4* | *MOSPD3* | 0.33 | 8.58E-03 |
| *EPHB4* | *MOV10* | 0.44 | 3.49E-04 |
| *EPHB4* | *MOV10L1* | 0.38 | 2.44E-03 |
| *EPHB4* | *MPPED2* | 0.36 | 3.93E-03 |
| *EPHB4* | *MR1* | 0.33 | 9.50E-03 |
| *EPHB4* | *MRI1* | 0.36 | 4.44E-03 |
| *EPHB4* | *MSH6* | 0.33 | 8.77E-03 |
| *EPHB4* | *MSL2* | 0.36 | 3.75E-03 |
| *EPHB4* | *MSS51* | 0.35 | 4.92E-03 |
| *EPHB4* | *MST1* | 0.32 | 1.11E-02 |
| *EPHB4* | *MSX1* | 0.33 | 8.74E-03 |
| *EPHB4* | *MTA1* | 0.35 | 5.03E-03 |
| *EPHB4* | *MTERF2* | 0.36 | 4.63E-03 |
| *EPHB4* | *MTHFR* | 0.37 | 3.32E-03 |
| *EPHB4* | *MTMR4* | 0.36 | 4.38E-03 |
| *EPHB4* | *MTOR* | 0.46 | 1.82E-04 |
| *EPHB4* | *MTSS1L* | 0.34 | 6.09E-03 |
| *EPHB4* | *MUM1* | 0.30 | 1.66E-02 |
| *EPHB4* | *MYCN* | 0.33 | 9.27E-03 |
| *EPHB4* | *MYH3* | 0.32 | 1.18E-02 |
| *EPHB4* | *MYO15B* | 0.37 | 2.86E-03 |
| *EPHB4* | *MYO18A* | 0.48 | 7.29E-05 |
| *EPHB4* | *MYO1G* | 0.39 | 1.76E-03 |
| *EPHB4* | *MYO9A* | 0.33 | 8.76E-03 |
| *EPHB4* | *MYOF* | 0.33 | 9.78E-03 |
| *EPHB4* | *MYPOP* | 0.42 | 6.65E-04 |
| *EPHB4* | *MZT1* | -0.32 | 1.19E-02 |
| *EPHB4* | *N4BP2* | 0.36 | 4.63E-03 |
| *EPHB4* | *NAA25* | 0.39 | 1.90E-03 |
| *EPHB4* | *NACAD* | 0.45 | 2.31E-04 |
| *EPHB4* | *NADK* | 0.31 | 1.39E-02 |
| *EPHB4* | *NADSYN1* | 0.31 | 1.53E-02 |
| *EPHB4* | *NAGLU* | 0.35 | 5.13E-03 |
| *EPHB4* | *NAGPA* | 0.33 | 8.59E-03 |
| *EPHB4* | *NAIP* | 0.39 | 1.67E-03 |
| *EPHB4* | *NANOS3* | 0.30 | 1.78E-02 |
| *EPHB4* | *NAPB* | 0.37 | 3.37E-03 |
| *EPHB4* | *NAPEPLD* | 0.40 | 1.11E-03 |
| *EPHB4* | *NARF* | 0.42 | 6.71E-04 |
| *EPHB4* | *NARFL* | 0.30 | 1.61E-02 |
| *EPHB4* | *NAT10* | 0.33 | 9.68E-03 |
| *EPHB4* | *NAT9* | 0.40 | 1.30E-03 |
| *EPHB4* | *NBEAL2* | 0.31 | 1.30E-02 |
| *EPHB4* | *NBPF1* | 0.30 | 1.72E-02 |
| *EPHB4* | *NBPF20* | 0.37 | 2.87E-03 |
| *EPHB4* | *NBPF3* | 0.41 | 9.62E-04 |
| *EPHB4* | *NCKAP5* | 0.31 | 1.31E-02 |
| *EPHB4* | *NCKAP5L* | 0.46 | 1.47E-04 |
| *EPHB4* | *NCOR2* | 0.58 | 9.29E-07 |
| *EPHB4* | *NDE1* | 0.40 | 1.28E-03 |
| *EPHB4* | *NDST1* | 0.30 | 1.59E-02 |
| *EPHB4* | *NDUFAF3* | -0.36 | 3.66E-03 |
| *EPHB4* | *NDUFB2-AS1* | 0.32 | 1.20E-02 |
| *EPHB4* | *NDUFB6* | -0.33 | 9.73E-03 |
| *EPHB4* | *NEB* | 0.32 | 1.20E-02 |
| *EPHB4* | *NEK11* | 0.52 | 1.24E-05 |
| *EPHB4* | *NEK8* | 0.32 | 1.18E-02 |
| *EPHB4* | *NEMP1* | 0.34 | 6.09E-03 |
| *EPHB4* | *NEO1* | 0.39 | 1.63E-03 |
| *EPHB4* | *NEURL2* | 0.33 | 9.32E-03 |
| *EPHB4* | *NF1* | 0.45 | 2.66E-04 |
| *EPHB4* | *NFATC1* | 0.31 | 1.40E-02 |
| *EPHB4* | *NFE2L1* | 0.36 | 4.55E-03 |
| *EPHB4* | *NFIB* | 0.45 | 2.52E-04 |
| *EPHB4* | *NFIC* | 0.34 | 6.85E-03 |
| *EPHB4* | *NFIX* | 0.44 | 3.41E-04 |
| *EPHB4* | *NFKBIA* | 0.34 | 7.25E-03 |
| *EPHB4* | *NFYC-AS1* | 0.32 | 9.99E-03 |
| *EPHB4* | *NGFR* | 0.35 | 5.89E-03 |
| *EPHB4* | *NHSL2* | 0.41 | 9.79E-04 |
| *EPHB4* | *NIN* | 0.34 | 6.71E-03 |
| *EPHB4* | *NINL* | 0.45 | 2.30E-04 |
| *EPHB4* | *NIPBL* | 0.32 | 1.00E-02 |
| *EPHB4* | *NISCH* | 0.33 | 9.59E-03 |
| *EPHB4* | *NKD2* | 0.36 | 3.61E-03 |
| *EPHB4* | *NLGN2* | 0.51 | 2.43E-05 |
| *EPHB4* | *NLRP1* | 0.52 | 1.56E-05 |
| *EPHB4* | *NLRP2* | 0.32 | 1.15E-02 |
| *EPHB4* | *NMRAL1* | 0.33 | 9.15E-03 |
| *EPHB4* | *NNT* | 0.36 | 4.54E-03 |
| *EPHB4* | *NOD1* | 0.38 | 2.52E-03 |
| *EPHB4* | *NOMO1* | 0.48 | 8.13E-05 |
| *EPHB4* | *NOMO2* | 0.34 | 6.11E-03 |
| *EPHB4* | *NOP10* | -0.30 | 1.74E-02 |
| *EPHB4* | *NOP2* | 0.37 | 3.42E-03 |
| *EPHB4* | *NOTCH1* | 0.31 | 1.56E-02 |
| *EPHB4* | *NPHP3* | 0.35 | 5.97E-03 |
| *EPHB4* | *NPHP4* | 0.54 | 7.13E-06 |
| *EPHB4* | *NPIPA1* | 0.35 | 4.98E-03 |
| *EPHB4* | *NPIPA5* | 0.31 | 1.41E-02 |
| *EPHB4* | *NPIPB3* | 0.33 | 8.68E-03 |
| *EPHB4* | *NPIPB5* | 0.35 | 5.68E-03 |
| *EPHB4* | *NPLOC4* | 0.35 | 5.34E-03 |
| *EPHB4* | *NPPC* | 0.42 | 7.49E-04 |
| *EPHB4* | *NPTXR* | 0.36 | 4.13E-03 |
| *EPHB4* | *NRDC* | 0.31 | 1.28E-02 |
| *EPHB4* | *NRSN2* | 0.31 | 1.40E-02 |
| *EPHB4* | *NSUN6* | 0.37 | 3.01E-03 |
| *EPHB4* | *NT5C* | 0.43 | 5.26E-04 |
| *EPHB4* | *NTF3* | 0.38 | 2.39E-03 |
| *EPHB4* | *NTN1* | 0.36 | 4.12E-03 |
| *EPHB4* | *NUAK1* | 0.50 | 3.44E-05 |
| *EPHB4* | *NUDCD3* | 0.32 | 1.08E-02 |
| *EPHB4* | *NUFIP2* | 0.48 | 8.60E-05 |
| *EPHB4* | *NUP133* | 0.44 | 3.33E-04 |
| *EPHB4* | *NUP210L* | 0.35 | 5.05E-03 |
| *EPHB4* | *NUP62* | 0.38 | 2.03E-03 |
| *EPHB4* | *NYAP1* | 0.35 | 5.66E-03 |
| *EPHB4* | *OBSCN* | 0.46 | 1.72E-04 |
| *EPHB4* | *OGDH* | 0.41 | 8.86E-04 |
| *EPHB4* | *OGFOD2* | 0.31 | 1.36E-02 |
| *EPHB4* | *OLFML2A* | 0.32 | 1.24E-02 |
| *EPHB4* | *OPA3* | 0.45 | 2.34E-04 |
| *EPHB4* | *OPHN1* | 0.32 | 1.13E-02 |
| *EPHB4* | *OPRL1* | 0.40 | 1.45E-03 |
| *EPHB4* | *ORAI2* | 0.47 | 1.00E-04 |
| *EPHB4* | *OSBPL5* | 0.34 | 6.27E-03 |
| *EPHB4* | *OSER1-AS1* | 0.41 | 8.55E-04 |
| *EPHB4* | *OTUB2* | 0.33 | 9.05E-03 |
| *EPHB4* | *OTUD3* | 0.37 | 3.35E-03 |
| *EPHB4* | *OTX1* | 0.36 | 4.30E-03 |
| *EPHB4* | *OVGP1* | 0.38 | 2.47E-03 |
| *EPHB4* | *OXCT1* | 0.40 | 1.16E-03 |
| *EPHB4* | *OXER1* | 0.36 | 4.04E-03 |
| *EPHB4* | *P2RX7* | 0.35 | 5.43E-03 |
| *EPHB4* | *P3H1* | 0.42 | 6.01E-04 |
| *EPHB4* | *PABPC1L* | 0.51 | 1.90E-05 |
| *EPHB4* | *PACS2* | 0.31 | 1.57E-02 |
| *EPHB4* | *PADI3* | 0.31 | 1.32E-02 |
| *EPHB4* | *PAK6* | 0.33 | 8.44E-03 |
| *EPHB4* | *PALB2* | 0.35 | 5.30E-03 |
| *EPHB4* | *PALMD* | 0.31 | 1.36E-02 |
| *EPHB4* | *PAN2* | 0.42 | 7.60E-04 |
| *EPHB4* | *PAN3* | 0.33 | 9.84E-03 |
| *EPHB4* | *PAN3-AS1* | 0.37 | 2.77E-03 |
| *EPHB4* | *PANK4* | 0.33 | 7.92E-03 |
| *EPHB4* | *PANO1* | 0.32 | 1.26E-02 |
| *EPHB4* | *PANX2* | 0.30 | 1.73E-02 |
| *EPHB4* | *PAOX* | 0.46 | 1.96E-04 |
| *EPHB4* | *PAPD7* | 0.34 | 7.66E-03 |
| *EPHB4* | *PAPLN* | 0.43 | 5.22E-04 |
| *EPHB4* | *PAPOLG* | 0.37 | 3.35E-03 |
| *EPHB4* | *PAQR4* | 0.36 | 3.71E-03 |
| *EPHB4* | *PARK2* | 0.38 | 2.03E-03 |
| *EPHB4* | *PARN* | 0.38 | 2.50E-03 |
| *EPHB4* | *PARP16* | 0.30 | 1.71E-02 |
| *EPHB4* | *PARP6* | 0.33 | 8.64E-03 |
| *EPHB4* | *PASK* | 0.44 | 3.43E-04 |
| *EPHB4* | *PAXBP1-AS1* | 0.30 | 1.60E-02 |
| *EPHB4* | *PCAT7* | 0.34 | 6.69E-03 |
| *EPHB4* | *PCDHB10* | 0.32 | 1.10E-02 |
| *EPHB4* | *PCDHB2* | 0.32 | 1.16E-02 |
| *EPHB4* | *PCDHB9* | 0.34 | 6.06E-03 |
| *EPHB4* | *PCDHGA4* | 0.36 | 3.88E-03 |
| *EPHB4* | *PCDHGA5* | 0.40 | 1.38E-03 |
| *EPHB4* | *PCNT* | 0.35 | 6.02E-03 |
| *EPHB4* | *PCNX* | 0.33 | 9.70E-03 |
| *EPHB4* | *PCNXL3* | 0.34 | 7.17E-03 |
| *EPHB4* | *PCNXL4* | 0.34 | 7.11E-03 |
| *EPHB4* | *PCOLCE-AS1* | 0.35 | 5.36E-03 |
| *EPHB4* | *PCSK9* | 0.30 | 1.66E-02 |
| *EPHB4* | *PCYOX1* | 0.38 | 2.38E-03 |
| *EPHB4* | *PCYOX1L* | 0.32 | 1.16E-02 |
| *EPHB4* | *PCYT2* | 0.34 | 6.67E-03 |
| *EPHB4* | *PDAP1* | 0.31 | 1.28E-02 |
| *EPHB4* | *PDCD11* | 0.31 | 1.38E-02 |
| *EPHB4* | *PDE2A* | 0.31 | 1.31E-02 |
| *EPHB4* | *PDE7A* | 0.37 | 3.37E-03 |
| *EPHB4* | *PDGFA* | 0.36 | 3.63E-03 |
| *EPHB4* | *PDS5A* | 0.30 | 1.64E-02 |
| *EPHB4* | *PDS5B* | 0.41 | 1.09E-03 |
| *EPHB4* | *PELI3* | 0.44 | 3.56E-04 |
| *EPHB4* | *PER3* | 0.39 | 1.51E-03 |
| *EPHB4* | *PEX10* | 0.36 | 3.70E-03 |
| *EPHB4* | *PGBD2* | 0.39 | 1.86E-03 |
| *EPHB4* | *PGS1* | 0.44 | 3.62E-04 |
| *EPHB4* | *PHACTR4* | 0.32 | 1.08E-02 |
| *EPHB4* | *PHC1* | 0.42 | 6.12E-04 |
| *EPHB4* | *PHC3* | 0.31 | 1.43E-02 |
| *EPHB4* | *PHF1* | 0.35 | 5.82E-03 |
| *EPHB4* | *PHF12* | 0.33 | 8.75E-03 |
| *EPHB4* | *PHF13* | 0.34 | 7.76E-03 |
| *EPHB4* | *PHF21A* | 0.42 | 6.16E-04 |
| *EPHB4* | *PHKG1* | 0.31 | 1.58E-02 |
| *EPHB4* | *PHRF1* | 0.32 | 1.09E-02 |
| *EPHB4* | *PI4KA* | 0.33 | 8.46E-03 |
| *EPHB4* | *PI4KB* | 0.31 | 1.31E-02 |
| *EPHB4* | *PIAS3* | 0.32 | 1.19E-02 |
| *EPHB4* | *PICK1* | 0.39 | 1.91E-03 |
| *EPHB4* | *PIDD1* | 0.39 | 1.65E-03 |
| *EPHB4* | *PIGG* | 0.43 | 5.49E-04 |
| *EPHB4* | *PIGS* | 0.32 | 1.05E-02 |
| *EPHB4* | *PIK3C2A* | 0.34 | 6.11E-03 |
| *EPHB4* | *PIK3CB* | 0.33 | 9.76E-03 |
| *EPHB4* | *PIK3R1* | 0.32 | 1.08E-02 |
| *EPHB4* | *PIK3R2* | 0.47 | 1.33E-04 |
| *EPHB4* | *PIK3R3* | 0.32 | 1.13E-02 |
| *EPHB4* | *PIK3R4* | 0.35 | 5.80E-03 |
| *EPHB4* | *PIKFYVE* | 0.30 | 1.74E-02 |
| *EPHB4* | *PILRB* | 0.45 | 2.79E-04 |
| *EPHB4* | *PIP4K2B* | 0.44 | 2.92E-04 |
| *EPHB4* | *PITPNM2* | 0.35 | 4.87E-03 |
| *EPHB4* | *PKD1* | 0.40 | 1.11E-03 |
| *EPHB4* | *PKI55* | 0.34 | 6.04E-03 |
| *EPHB4* | *PKN2* | 0.36 | 4.11E-03 |
| *EPHB4* | *PKP4* | 0.32 | 1.09E-02 |
| *EPHB4* | *PLA2G15* | 0.31 | 1.48E-02 |
| *EPHB4* | *PLA2G6* | 0.50 | 3.42E-05 |
| *EPHB4* | *PLA2R1* | 0.42 | 6.58E-04 |
| *EPHB4* | *PLBD2* | 0.45 | 2.58E-04 |
| *EPHB4* | *PLCD4* | 0.37 | 3.11E-03 |
| *EPHB4* | *PLCG1* | 0.44 | 2.94E-04 |
| *EPHB4* | *PLCH2* | 0.47 | 1.04E-04 |
| *EPHB4* | *PLCL1* | 0.38 | 2.21E-03 |
| *EPHB4* | *PLEC* | 0.30 | 1.60E-02 |
| *EPHB4* | *PLEKHA4* | 0.43 | 5.18E-04 |
| *EPHB4* | *PLEKHG3* | 0.46 | 1.49E-04 |
| *EPHB4* | *PLEKHG4B* | 0.48 | 7.11E-05 |
| *EPHB4* | *PLEKHH3* | 0.34 | 6.30E-03 |
| *EPHB4* | *PLEKHM2* | 0.31 | 1.34E-02 |
| *EPHB4* | *PLEKHO2* | 0.47 | 1.24E-04 |
| *EPHB4* | *PLGLB1* | 0.36 | 3.90E-03 |
| *EPHB4* | *PLIN5* | 0.42 | 7.76E-04 |
| *EPHB4* | *PLOD3* | 0.43 | 4.28E-04 |
| *EPHB4* | *PLXNA1* | 0.62 | 8.18E-08 |
| *EPHB4* | *PLXNB1* | 0.45 | 2.71E-04 |
| *EPHB4* | *PLXNB2* | 0.32 | 1.04E-02 |
| *EPHB4* | *PLXNB3* | 0.53 | 9.30E-06 |
| *EPHB4* | *PM20D1* | 0.39 | 1.81E-03 |
| *EPHB4* | *PMS2* | 0.36 | 4.07E-03 |
| *EPHB4* | *PNKP* | 0.37 | 3.50E-03 |
| *EPHB4* | *PNPLA6* | 0.37 | 3.13E-03 |
| *EPHB4* | *PNPLA8* | 0.36 | 3.81E-03 |
| *EPHB4* | *POGLUT1* | 0.38 | 2.13E-03 |
| *EPHB4* | *POLD1* | 0.48 | 7.11E-05 |
| *EPHB4* | *POLE* | 0.49 | 6.37E-05 |
| *EPHB4* | *POLG2* | 0.32 | 1.15E-02 |
| *EPHB4* | *POLM* | 0.34 | 6.51E-03 |
| *EPHB4* | *POLR1A* | 0.31 | 1.56E-02 |
| *EPHB4* | *POLR2A* | 0.47 | 1.12E-04 |
| *EPHB4* | *POLR2B* | 0.36 | 4.14E-03 |
| *EPHB4* | *POLR2J2* | 0.50 | 3.30E-05 |
| *EPHB4* | *POLR2K* | -0.31 | 1.30E-02 |
| *EPHB4* | *POLR3A* | 0.43 | 5.22E-04 |
| *EPHB4* | *POLR3B* | 0.34 | 7.44E-03 |
| *EPHB4* | *POMZP3* | 0.33 | 7.80E-03 |
| *EPHB4* | *POR* | 0.36 | 3.61E-03 |
| *EPHB4* | *POU3F1* | 0.39 | 1.68E-03 |
| *EPHB4* | *POU5F1B* | 0.33 | 8.54E-03 |
| *EPHB4* | *POU6F2* | 0.36 | 4.07E-03 |
| *EPHB4* | *PPARGC1A* | 0.33 | 8.47E-03 |
| *EPHB4* | *PPARGC1B* | 0.34 | 7.01E-03 |
| *EPHB4* | *PPFIA1* | 0.34 | 7.22E-03 |
| *EPHB4* | *PPFIBP2* | 0.33 | 9.08E-03 |
| *EPHB4* | *PPHLN1* | 0.34 | 6.86E-03 |
| *EPHB4* | *PPIP5K1* | 0.33 | 8.08E-03 |
| *EPHB4* | *PPM1F* | 0.35 | 5.58E-03 |
| *EPHB4* | *PPP1R12C* | 0.34 | 6.78E-03 |
| *EPHB4* | *PPP1R13B* | 0.37 | 3.31E-03 |
| *EPHB4* | *PPP1R16B* | 0.36 | 3.60E-03 |
| *EPHB4* | *PPP1R35* | 0.33 | 9.76E-03 |
| *EPHB4* | *PPP1R37* | 0.41 | 8.75E-04 |
| *EPHB4* | *PPP1R3D* | 0.33 | 7.91E-03 |
| *EPHB4* | *PPP1R9B* | 0.36 | 4.58E-03 |
| *EPHB4* | *PPP2R3A* | 0.37 | 3.10E-03 |
| *EPHB4* | *PPP2R3B* | 0.36 | 4.11E-03 |
| *EPHB4* | *PPP4R3B* | 0.36 | 4.62E-03 |
| *EPHB4* | *PPP5D1* | 0.30 | 1.62E-02 |
| *EPHB4* | *PPP6R1* | 0.32 | 1.18E-02 |
| *EPHB4* | *PPP6R2* | 0.35 | 5.41E-03 |
| *EPHB4* | *PQLC2* | 0.33 | 9.28E-03 |
| *EPHB4* | *PRDM11* | 0.47 | 1.37E-04 |
| *EPHB4* | *PRDM2* | 0.40 | 1.17E-03 |
| *EPHB4* | *PREX2* | 0.34 | 7.47E-03 |
| *EPHB4* | *PRH1* | 0.37 | 3.35E-03 |
| *EPHB4* | *PRKAB1* | 0.39 | 1.95E-03 |
| *EPHB4* | *PRKCZ* | 0.37 | 2.67E-03 |
| *EPHB4* | *PRKD2* | 0.50 | 3.74E-05 |
| *EPHB4* | *PRKRIP1* | 0.44 | 3.04E-04 |
| *EPHB4* | *PRMT5-AS1* | 0.50 | 3.66E-05 |
| *EPHB4* | *PRNCR1* | 0.40 | 1.40E-03 |
| *EPHB4* | *PROCA1* | 0.31 | 1.33E-02 |
| *EPHB4* | *PRPF3* | 0.31 | 1.48E-02 |
| *EPHB4* | *PRPF40B* | 0.42 | 6.69E-04 |
| *EPHB4* | *PRPF8* | 0.40 | 1.36E-03 |
| *EPHB4* | *PRR12* | 0.33 | 8.52E-03 |
| *EPHB4* | *PRR19* | 0.33 | 8.30E-03 |
| *EPHB4* | *PRR3* | 0.32 | 1.20E-02 |
| *EPHB4* | *PRRC2A* | 0.51 | 2.44E-05 |
| *EPHB4* | *PRRC2B* | 0.35 | 5.35E-03 |
| *EPHB4* | *PRRG2* | 0.38 | 2.43E-03 |
| *EPHB4* | *PRRT1* | 0.45 | 2.89E-04 |
| *EPHB4* | *PRRT2* | 0.36 | 3.71E-03 |
| *EPHB4* | *PRSS53* | 0.45 | 2.60E-04 |
| *EPHB4* | *PRX* | 0.51 | 2.02E-05 |
| *EPHB4* | *PSKH1* | 0.35 | 5.31E-03 |
| *EPHB4* | *PSMA3-AS1* | 0.31 | 1.27E-02 |
| *EPHB4* | *PSMD6* | -0.37 | 3.09E-03 |
| *EPHB4* | *PSMD6-AS2* | 0.33 | 8.37E-03 |
| *EPHB4* | *PSMG2* | -0.33 | 9.42E-03 |
| *EPHB4* | *PSPN* | 0.32 | 1.25E-02 |
| *EPHB4* | *PTCD1* | 0.52 | 1.38E-05 |
| *EPHB4* | *PTCH1* | 0.37 | 2.83E-03 |
| *EPHB4* | *PTK7* | 0.38 | 2.16E-03 |
| *EPHB4* | *PTOV1-AS1* | 0.42 | 6.19E-04 |
| *EPHB4* | *PTOV1-AS2* | 0.39 | 1.58E-03 |
| *EPHB4* | *PTPDC1* | 0.34 | 7.05E-03 |
| *EPHB4* | *PTPN1* | 0.40 | 1.31E-03 |
| *EPHB4* | *PTPN14* | 0.35 | 5.67E-03 |
| *EPHB4* | *PTPN4* | 0.37 | 3.40E-03 |
| *EPHB4* | *PTPRF* | 0.50 | 2.85E-05 |
| *EPHB4* | *PTPRJ* | 0.38 | 2.44E-03 |
| *EPHB4* | *PTPRS* | 0.33 | 9.77E-03 |
| *EPHB4* | *PTS* | -0.36 | 4.23E-03 |
| *EPHB4* | *PTTG1* | -0.32 | 1.25E-02 |
| *EPHB4* | *PUM1* | 0.32 | 1.05E-02 |
| *EPHB4* | *PURB* | 0.38 | 2.10E-03 |
| *EPHB4* | *PUS7* | 0.30 | 1.70E-02 |
| *EPHB4* | *PYCARD-AS1* | 0.41 | 9.05E-04 |
| *EPHB4* | *PYGB* | 0.35 | 4.85E-03 |
| *EPHB4* | *PYROXD2* | 0.34 | 6.81E-03 |
| *EPHB4* | *QPCTL* | 0.49 | 5.53E-05 |
| *EPHB4* | *QRICH2* | 0.38 | 2.03E-03 |
| *EPHB4* | *QSER1* | 0.30 | 1.77E-02 |
| *EPHB4* | *QTRT1* | 0.30 | 1.77E-02 |
| *EPHB4* | *RAB11FIP3* | 0.36 | 4.49E-03 |
| *EPHB4* | *RAB11FIP4* | 0.31 | 1.45E-02 |
| *EPHB4* | *RAB29* | 0.34 | 6.63E-03 |
| *EPHB4* | *RAB40AL* | 0.31 | 1.48E-02 |
| *EPHB4* | *RAB4B* | 0.36 | 4.42E-03 |
| *EPHB4* | *RABEP2* | 0.43 | 4.92E-04 |
| *EPHB4* | *RAD51AP2* | 0.36 | 4.54E-03 |
| *EPHB4* | *RAD52* | 0.37 | 2.89E-03 |
| *EPHB4* | *RAG1* | 0.42 | 7.65E-04 |
| *EPHB4* | *RAI1* | 0.49 | 4.86E-05 |
| *EPHB4* | *RALGAPA1* | 0.33 | 8.08E-03 |
| *EPHB4* | *RALGAPA2* | 0.33 | 8.87E-03 |
| *EPHB4* | *RAP1GAP* | 0.30 | 1.75E-02 |
| *EPHB4* | *RAP1GAP2* | 0.35 | 4.75E-03 |
| *EPHB4* | *RAPGEF4* | 0.31 | 1.42E-02 |
| *EPHB4* | *RAPGEF6* | 0.39 | 1.94E-03 |
| *EPHB4* | *RARA* | 0.35 | 4.70E-03 |
| *EPHB4* | *RARG* | 0.43 | 4.76E-04 |
| *EPHB4* | *RASA1* | 0.44 | 2.95E-04 |
| *EPHB4* | *RASA4* | 0.40 | 1.24E-03 |
| *EPHB4* | *RASGRP1* | 0.35 | 4.74E-03 |
| *EPHB4* | *RASSF10* | 0.38 | 2.23E-03 |
| *EPHB4* | *RAVER2* | 0.32 | 1.14E-02 |
| *EPHB4* | *RBBP8NL* | 0.32 | 1.15E-02 |
| *EPHB4* | *RBFOX2* | 0.37 | 3.28E-03 |
| *EPHB4* | *RBL1* | 0.33 | 8.58E-03 |
| *EPHB4* | *RBM10* | 0.45 | 2.43E-04 |
| *EPHB4* | *RBM27* | 0.33 | 7.98E-03 |
| *EPHB4* | *RBP5* | 0.38 | 2.60E-03 |
| *EPHB4* | *RCAN3* | 0.32 | 1.21E-02 |
| *EPHB4* | *RCC2* | 0.31 | 1.45E-02 |
| *EPHB4* | *RDH16* | 0.44 | 3.62E-04 |
| *EPHB4* | *RECQL5* | 0.45 | 2.88E-04 |
| *EPHB4* | *REEP6* | -0.32 | 1.08E-02 |
| *EPHB4* | *REP15* | 0.33 | 8.52E-03 |
| *EPHB4* | *REPS1* | 0.34 | 7.68E-03 |
| *EPHB4* | *REPS2* | 0.45 | 2.57E-04 |
| *EPHB4* | *REST* | 0.41 | 1.06E-03 |
| *EPHB4* | *REV3L* | 0.43 | 4.94E-04 |
| *EPHB4* | *RFNG* | 0.31 | 1.48E-02 |
| *EPHB4* | *RFWD3* | 0.32 | 1.03E-02 |
| *EPHB4* | *RFX1* | 0.33 | 8.40E-03 |
| *EPHB4* | *RFX5* | 0.37 | 3.38E-03 |
| *EPHB4* | *RFX7* | 0.30 | 1.75E-02 |
| *EPHB4* | *RGAG4* | 0.50 | 3.09E-05 |
| *EPHB4* | *RGMB* | 0.35 | 4.97E-03 |
| *EPHB4* | *RGS12* | 0.39 | 1.51E-03 |
| *EPHB4* | *RGS6* | 0.32 | 1.13E-02 |
| *EPHB4* | *RGS7BP* | 0.38 | 2.33E-03 |
| *EPHB4* | *RHBDD1* | 0.33 | 8.42E-03 |
| *EPHB4* | *RHBDD2* | 0.44 | 3.71E-04 |
| *EPHB4* | *RHBDD3* | 0.32 | 1.17E-02 |
| *EPHB4* | *RHBDF2* | 0.40 | 1.38E-03 |
| *EPHB4* | *RHNO1* | 0.32 | 1.23E-02 |
| *EPHB4* | *RHOA* | -0.34 | 6.69E-03 |
| *EPHB4* | *RHOBTB2* | 0.50 | 3.69E-05 |
| *EPHB4* | *RHOJ* | 0.31 | 1.50E-02 |
| *EPHB4* | *RHOT1* | 0.34 | 7.19E-03 |
| *EPHB4* | *RHOT2* | 0.36 | 4.13E-03 |
| *EPHB4* | *RIC8B* | 0.32 | 1.18E-02 |
| *EPHB4* | *RILPL1* | 0.47 | 1.10E-04 |
| *EPHB4* | *RILPL2* | 0.35 | 5.57E-03 |
| *EPHB4* | *RIMBP2* | 0.30 | 1.64E-02 |
| *EPHB4* | *RIN2* | 0.30 | 1.76E-02 |
| *EPHB4* | *RING1* | 0.35 | 5.31E-03 |
| *EPHB4* | *RMI2* | 0.31 | 1.29E-02 |
| *EPHB4* | *RNF150* | 0.31 | 1.50E-02 |
| *EPHB4* | *RNF165* | 0.36 | 4.12E-03 |
| *EPHB4* | *RNF166* | 0.42 | 6.10E-04 |
| *EPHB4* | *RNF216* | 0.33 | 9.13E-03 |
| *EPHB4* | *RNF217-AS1* | 0.38 | 2.08E-03 |
| *EPHB4* | *RNF31* | 0.31 | 1.56E-02 |
| *EPHB4* | *RNF34* | 0.36 | 3.87E-03 |
| *EPHB4* | *RNF40* | 0.33 | 9.88E-03 |
| *EPHB4* | *RNF43* | 0.36 | 4.31E-03 |
| *EPHB4* | *RNF44* | 0.37 | 3.32E-03 |
| *EPHB4* | *RNU11* | 0.38 | 2.02E-03 |
| *EPHB4* | *RNU12* | 0.38 | 2.61E-03 |
| *EPHB4* | *ROBO2* | 0.34 | 6.55E-03 |
| *EPHB4* | *ROGDI* | 0.39 | 1.99E-03 |
| *EPHB4* | *RP1-101K10.6* | 0.31 | 1.45E-02 |
| *EPHB4* | *RP1-168L15.5* | 0.41 | 9.22E-04 |
| *EPHB4* | *RP1-202O8.3* | 0.41 | 8.39E-04 |
| *EPHB4* | *RP1-221C16.8* | 0.37 | 3.43E-03 |
| *EPHB4* | *RP1-34B20.21* | 0.31 | 1.30E-02 |
| *EPHB4* | *RP1-40E16.12* | 0.36 | 4.43E-03 |
| *EPHB4* | *RP1-43E13.2* | 0.37 | 3.08E-03 |
| *EPHB4* | *RP1-78B3.1* | 0.32 | 1.20E-02 |
| *EPHB4* | *RP11-1000B6.8* | 0.32 | 1.03E-02 |
| *EPHB4* | *RP11-1006G14.1* | 0.35 | 5.33E-03 |
| *EPHB4* | *RP11-1017G21.4* | 0.36 | 3.60E-03 |
| *EPHB4* | *RP11-1017G21.5* | 0.33 | 7.89E-03 |
| *EPHB4* | *RP11-102M11.2* | 0.40 | 1.11E-03 |
| *EPHB4* | *RP11-1038A11.3* | 0.42 | 5.78E-04 |
| *EPHB4* | *RP11-107N15.1* | 0.38 | 2.38E-03 |
| *EPHB4* | *RP11-108M9.6* | 0.44 | 3.24E-04 |
| *EPHB4* | *RP11-1114A5.4* | 0.33 | 8.11E-03 |
| *EPHB4* | *RP11-114F3.4* | 0.34 | 6.74E-03 |
| *EPHB4* | *RP11-114N19.3* | 0.36 | 4.28E-03 |
| *EPHB4* | *RP11-11M20.4* | 0.35 | 5.79E-03 |
| *EPHB4* | *RP11-122K13.12* | 0.35 | 5.06E-03 |
| *EPHB4* | *RP11-1246C19.1* | 0.32 | 1.18E-02 |
| *EPHB4* | *RP11-124N14.3* | 0.32 | 1.13E-02 |
| *EPHB4* | *RP11-1252I4.2* | 0.31 | 1.29E-02 |
| *EPHB4* | *RP11-1277A3.1* | 0.31 | 1.38E-02 |
| *EPHB4* | *RP11-127B20.3* | 0.38 | 2.02E-03 |
| *EPHB4* | *RP11-1299A16.3* | 0.30 | 1.68E-02 |
| *EPHB4* | *RP11-138J23.1* | 0.32 | 1.25E-02 |
| *EPHB4* | *RP11-141B14.1* | 0.45 | 2.77E-04 |
| *EPHB4* | *RP11-147L13.11* | 0.31 | 1.50E-02 |
| *EPHB4* | *RP11-147L13.13* | 0.36 | 4.18E-03 |
| *EPHB4* | *RP11-147L13.2* | 0.35 | 4.66E-03 |
| *EPHB4* | *RP11-152N13.5* | 0.35 | 5.00E-03 |
| *EPHB4* | *RP11-154H23.3* | 0.37 | 2.88E-03 |
| *EPHB4* | *RP11-157P1.4* | 0.33 | 8.05E-03 |
| *EPHB4* | *RP11-159D12.5* | 0.44 | 3.47E-04 |
| *EPHB4* | *RP11-15B17.1* | 0.50 | 4.10E-05 |
| *EPHB4* | *RP11-15K19.2* | 0.43 | 5.59E-04 |
| *EPHB4* | *RP11-160E2.6* | 0.33 | 9.63E-03 |
| *EPHB4* | *RP11-166O4.6* | 0.39 | 1.88E-03 |
| *EPHB4* | *RP11-16N11.2* | 0.32 | 1.05E-02 |
| *EPHB4* | *RP11-178L8.7* | 0.35 | 6.01E-03 |
| *EPHB4* | *RP11-182L21.6* | 0.53 | 9.42E-06 |
| *EPHB4* | *RP11-18H7.1* | 0.52 | 1.72E-05 |
| *EPHB4* | *RP11-191L9.4* | 0.36 | 4.09E-03 |
| *EPHB4* | *RP11-196G18.23* | 0.46 | 1.44E-04 |
| *EPHB4* | *RP11-196G18.24* | 0.32 | 1.04E-02 |
| *EPHB4* | *RP11-206L10.9* | 0.37 | 3.50E-03 |
| *EPHB4* | *RP11-211G3.2* | 0.35 | 5.47E-03 |
| *EPHB4* | *RP11-227G15.12* | 0.35 | 4.82E-03 |
| *EPHB4* | *RP11-22P6.2* | 0.41 | 9.64E-04 |
| *EPHB4* | *RP11-231C14.7* | 0.43 | 4.59E-04 |
| *EPHB4* | *RP11-231D20.2* | 0.44 | 3.04E-04 |
| *EPHB4* | *RP11-242D8.1* | 0.41 | 8.13E-04 |
| *EPHB4* | *RP11-251M1.1* | 0.35 | 5.70E-03 |
| *EPHB4* | *RP11-253E3.3* | 0.36 | 4.15E-03 |
| *EPHB4* | *RP11-254F7.2* | 0.47 | 1.28E-04 |
| *EPHB4* | *RP11-268P4.4* | 0.32 | 1.03E-02 |
| *EPHB4* | *RP11-276M12.1* | 0.31 | 1.46E-02 |
| *EPHB4* | *RP11-283G6.3* | 0.34 | 7.57E-03 |
| *EPHB4* | *RP11-283G6.5* | 0.47 | 1.35E-04 |
| *EPHB4* | *RP11-290D2.6* | 0.32 | 1.05E-02 |
| *EPHB4* | *RP11-295P9.13* | 0.41 | 1.03E-03 |
| *EPHB4* | *RP11-296O14.3* | 0.42 | 6.09E-04 |
| *EPHB4* | *RP11-298I3.4* | 0.41 | 9.51E-04 |
| *EPHB4* | *RP11-299H21.1* | 0.31 | 1.38E-02 |
| *EPHB4* | *RP11-2B6.2* | 0.34 | 6.16E-03 |
| *EPHB4* | *RP11-312B8.1* | 0.37 | 3.41E-03 |
| *EPHB4* | *RP11-314N13.10* | 0.31 | 1.32E-02 |
| *EPHB4* | *RP11-317N8.5* | 0.43 | 4.97E-04 |
| *EPHB4* | *RP11-318A15.2* | 0.35 | 5.06E-03 |
| *EPHB4* | *RP11-319E16.2* | 0.34 | 7.58E-03 |
| *EPHB4* | *RP11-31F15.2* | 0.33 | 9.34E-03 |
| *EPHB4* | *RP11-324E6.6* | 0.35 | 5.85E-03 |
| *EPHB4* | *RP11-333E1.1* | 0.35 | 4.65E-03 |
| *EPHB4* | *RP11-334C17.5* | 0.31 | 1.33E-02 |
| *EPHB4* | *RP11-334J6.7* | 0.39 | 1.55E-03 |
| *EPHB4* | *RP11-343H19.1* | 0.46 | 1.64E-04 |
| *EPHB4* | *RP11-347I19.7* | 0.39 | 1.86E-03 |
| *EPHB4* | *RP11-348N5.9* | 0.41 | 8.10E-04 |
| *EPHB4* | *RP11-350N15.5* | 0.37 | 3.39E-03 |
| *EPHB4* | *RP11-351D16.3* | 0.38 | 2.42E-03 |
| *EPHB4* | *RP11-355F16.1* | 0.35 | 5.92E-03 |
| *EPHB4* | *RP11-356I2.4* | 0.48 | 8.85E-05 |
| *EPHB4* | *RP11-363J20.1* | 0.31 | 1.44E-02 |
| *EPHB4* | *RP11-367J11.3* | 0.36 | 3.63E-03 |
| *EPHB4* | *RP11-367N14.2* | 0.39 | 1.86E-03 |
| *EPHB4* | *RP11-370I10.12* | 0.36 | 3.78E-03 |
| *EPHB4* | *RP11-378J18.8* | 0.51 | 2.56E-05 |
| *EPHB4* | *RP11-379K17.12* | 0.37 | 3.21E-03 |
| *EPHB4* | *RP11-37C7.3* | 0.36 | 3.98E-03 |
| *EPHB4* | *RP11-386G11.5* | 0.37 | 2.97E-03 |
| *EPHB4* | *RP11-386I14.4* | 0.30 | 1.73E-02 |
| *EPHB4* | *RP11-38L15.3* | 0.37 | 3.32E-03 |
| *EPHB4* | *RP11-393I2.4* | 0.37 | 3.46E-03 |
| *EPHB4* | *RP11-395A13.2* | 0.37 | 2.91E-03 |
| *EPHB4* | *RP11-397A16.1* | 0.33 | 7.82E-03 |
| *EPHB4* | *RP11-398C13.6* | 0.43 | 5.03E-04 |
| *EPHB4* | *RP11-398K22.12* | 0.40 | 1.23E-03 |
| *EPHB4* | *RP11-408B11.2* | 0.45 | 2.28E-04 |
| *EPHB4* | *RP11-408H1.3* | 0.37 | 3.32E-03 |
| *EPHB4* | *RP11-418J17.1* | 0.39 | 1.67E-03 |
| *EPHB4* | *RP11-421M1.8* | 0.40 | 1.25E-03 |
| *EPHB4* | *RP11-426C22.4* | 0.31 | 1.51E-02 |
| *EPHB4* | *RP11-426L16.9* | 0.38 | 2.54E-03 |
| *EPHB4* | *RP11-434D9.2* | 0.34 | 7.20E-03 |
| *EPHB4* | *RP11-440L14.1* | 0.40 | 1.34E-03 |
| *EPHB4* | *RP11-440L14.4* | 0.37 | 3.35E-03 |
| *EPHB4* | *RP11-444D3.1* | 0.39 | 1.57E-03 |
| *EPHB4* | *RP11-448A19.1* | 0.30 | 1.77E-02 |
| *EPHB4* | *RP11-44N21.1* | 0.35 | 5.27E-03 |
| *EPHB4* | *RP11-452L6.1* | 0.38 | 2.19E-03 |
| *EPHB4* | *RP11-457M11.5* | 0.34 | 6.59E-03 |
| *EPHB4* | *RP11-458J1.1* | 0.31 | 1.31E-02 |
| *EPHB4* | *RP11-467D6.1* | 0.32 | 1.20E-02 |
| *EPHB4* | *RP11-467L13.7* | 0.33 | 9.24E-03 |
| *EPHB4* | *RP11-46D6.1* | 0.30 | 1.63E-02 |
| *EPHB4* | *RP11-471B22.3* | 0.37 | 3.40E-03 |
| *EPHB4* | *RP11-474I16.8* | 0.33 | 9.83E-03 |
| *EPHB4* | *RP11-474O21.5* | 0.31 | 1.27E-02 |
| *EPHB4* | *RP11-479G22.8* | 0.41 | 8.64E-04 |
| *EPHB4* | *RP11-479O9.4* | 0.43 | 4.49E-04 |
| *EPHB4* | *RP11-480I12.10* | 0.34 | 7.15E-03 |
| *EPHB4* | *RP11-481J2.2* | 0.37 | 3.23E-03 |
| *EPHB4* | *RP11-484K9.4* | 0.36 | 4.20E-03 |
| *EPHB4* | *RP11-486A14.2* | 0.38 | 2.25E-03 |
| *EPHB4* | *RP11-486O12.2* | 0.41 | 9.67E-04 |
| *EPHB4* | *RP11-488C13.5* | 0.40 | 1.48E-03 |
| *EPHB4* | *RP11-488L18.10* | 0.34 | 6.37E-03 |
| *EPHB4* | *RP11-48B3.5* | 0.37 | 2.81E-03 |
| *EPHB4* | *RP11-495P10.1* | 0.32 | 1.10E-02 |
| *EPHB4* | *RP11-498C9.15* | 0.34 | 7.52E-03 |
| *EPHB4* | *RP11-498C9.2* | 0.35 | 5.73E-03 |
| *EPHB4* | *RP11-499F3.2* | 0.31 | 1.29E-02 |
| *EPHB4* | *RP11-517P14.2* | 0.33 | 7.99E-03 |
| *EPHB4* | *RP11-526I2.5* | 0.32 | 1.04E-02 |
| *EPHB4* | *RP11-544I20.2* | 0.35 | 4.99E-03 |
| *EPHB4* | *RP11-545I5.3* | 0.37 | 3.52E-03 |
| *EPHB4* | *RP11-545M17.1* | 0.34 | 6.85E-03 |
| *EPHB4* | *RP11-547D23.1* | 0.49 | 5.79E-05 |
| *EPHB4* | *RP11-548H3.1* | 0.33 | 7.83E-03 |
| *EPHB4* | *RP11-552F3.9* | 0.50 | 4.15E-05 |
| *EPHB4* | *RP11-567M16.3* | 0.38 | 2.09E-03 |
| *EPHB4* | *RP11-572M11.4* | 0.33 | 9.67E-03 |
| *EPHB4* | *RP11-574F21.2* | 0.41 | 9.60E-04 |
| *EPHB4* | *RP11-575F12.3* | 0.31 | 1.59E-02 |
| *EPHB4* | *RP11-582J16.5* | 0.37 | 3.53E-03 |
| *EPHB4* | *RP11-589C21.5* | 0.49 | 4.69E-05 |
| *EPHB4* | *RP11-59C5.3* | 0.36 | 4.08E-03 |
| *EPHB4* | *RP11-5C23.1* | 0.37 | 3.47E-03 |
| *EPHB4* | *RP11-600F24.7* | 0.42 | 7.85E-04 |
| *EPHB4* | *RP11-603J24.5* | 0.30 | 1.74E-02 |
| *EPHB4* | *RP11-610P16.1* | 0.33 | 9.59E-03 |
| *EPHB4* | *RP11-61K9.3* | 0.42 | 7.93E-04 |
| *EPHB4* | *RP11-624M8.1* | 0.40 | 1.24E-03 |
| *EPHB4* | *RP11-649A18.12* | 0.48 | 9.05E-05 |
| *EPHB4* | *RP11-651L5.3* | 0.34 | 6.72E-03 |
| *EPHB4* | *RP11-660L16.2* | 0.38 | 2.18E-03 |
| *EPHB4* | *RP11-666A20.4* | 0.31 | 1.45E-02 |
| *EPHB4* | *RP11-66N24.3* | 0.42 | 6.66E-04 |
| *EPHB4* | *RP11-67L2.2* | 0.49 | 5.90E-05 |
| *EPHB4* | *RP11-686O6.2* | 0.36 | 4.06E-03 |
| *EPHB4* | *RP11-692D12.1* | 0.50 | 3.39E-05 |
| *EPHB4* | *RP11-69E11.4* | 0.42 | 7.06E-04 |
| *EPHB4* | *RP11-707G18.1* | 0.43 | 4.80E-04 |
| *EPHB4* | *RP11-722G7.1* | 0.47 | 1.35E-04 |
| *EPHB4* | *RP11-731C17.2* | 0.39 | 1.90E-03 |
| *EPHB4* | *RP11-734K2.4* | 0.34 | 7.26E-03 |
| *EPHB4* | *RP11-739B23.1* | 0.35 | 4.84E-03 |
| *EPHB4* | *RP11-757F18.5* | 0.31 | 1.53E-02 |
| *EPHB4* | *RP11-758H9.2* | 0.30 | 1.66E-02 |
| *EPHB4* | *RP11-774O3.3* | 0.40 | 1.25E-03 |
| *EPHB4* | *RP11-776H12.1* | 0.52 | 1.45E-05 |
| *EPHB4* | *RP11-77H9.2* | 0.30 | 1.59E-02 |
| *EPHB4* | *RP11-77I22.2* | 0.38 | 2.63E-03 |
| *EPHB4* | *RP11-77K12.9* | 0.38 | 2.14E-03 |
| *EPHB4* | *RP11-77P16.4* | 0.43 | 4.81E-04 |
| *EPHB4* | *RP11-77P6.2* | 0.32 | 1.16E-02 |
| *EPHB4* | *RP11-783K16.14* | 0.36 | 4.35E-03 |
| *EPHB4* | *RP11-793H13.11* | 0.45 | 2.79E-04 |
| *EPHB4* | *RP11-795F19.5* | 0.34 | 6.24E-03 |
| *EPHB4* | *RP11-796E2.4* | 0.36 | 3.86E-03 |
| *EPHB4* | *RP11-79H23.3* | 0.37 | 2.79E-03 |
| *EPHB4* | *RP11-79O8.1* | 0.39 | 1.98E-03 |
| *EPHB4* | *RP11-7F17.8* | 0.32 | 1.01E-02 |
| *EPHB4* | *RP11-806H10.4* | 0.55 | 3.60E-06 |
| *EPHB4* | *RP11-823E8.3* | 0.39 | 1.96E-03 |
| *EPHB4* | *RP11-83A24.2* | 0.34 | 7.32E-03 |
| *EPHB4* | *RP11-849H4.4* | 0.39 | 1.93E-03 |
| *EPHB4* | *RP11-85A1.3* | 0.37 | 3.12E-03 |
| *EPHB4* | *RP11-88H9.2* | 0.35 | 4.72E-03 |
| *EPHB4* | *RP11-932O9.7* | 0.42 | 7.48E-04 |
| *EPHB4* | *RP11-936I5.1* | 0.30 | 1.60E-02 |
| *EPHB4* | *RP11-96D1.11* | 0.32 | 1.15E-02 |
| *EPHB4* | *RP11-983P16.4* | 0.32 | 1.02E-02 |
| *EPHB4* | *RP13-516M14.1* | 0.30 | 1.66E-02 |
| *EPHB4* | *RP13-516M14.4* | 0.47 | 1.26E-04 |
| *EPHB4* | *RP13-766D20.4* | 0.34 | 7.30E-03 |
| *EPHB4* | *RP13-977J11.2* | 0.50 | 3.62E-05 |
| *EPHB4* | *RP3-323P24.3* | 0.32 | 1.16E-02 |
| *EPHB4* | *RP3-399L15.3* | 0.38 | 2.35E-03 |
| *EPHB4* | *RP3-402G11.26* | 0.31 | 1.36E-02 |
| *EPHB4* | *RP3-406P24.3* | 0.34 | 6.13E-03 |
| *EPHB4* | *RP3-439F8.1* | 0.35 | 5.88E-03 |
| *EPHB4* | *RP3-441A12.1* | 0.32 | 1.11E-02 |
| *EPHB4* | *RP3-508I15.9* | 0.32 | 1.09E-02 |
| *EPHB4* | *RP4-535B20.1* | 0.33 | 9.16E-03 |
| *EPHB4* | *RP4-549L20.3* | 0.35 | 5.47E-03 |
| *EPHB4* | *RP4-605O3.4* | 0.30 | 1.74E-02 |
| *EPHB4* | *RP4-613B23.1* | 0.35 | 5.17E-03 |
| *EPHB4* | *RP4-635E18.8* | 0.36 | 3.55E-03 |
| *EPHB4* | *RP4-669L17.10* | 0.32 | 1.25E-02 |
| *EPHB4* | *RP4-734G22.3* | 0.33 | 8.86E-03 |
| *EPHB4* | *RP4-761J14.8* | 0.39 | 1.78E-03 |
| *EPHB4* | *RP4-773N10.4* | 0.35 | 5.11E-03 |
| *EPHB4* | *RP4-798A10.7* | 0.32 | 1.04E-02 |
| *EPHB4* | *RP5-1009E24.8* | 0.33 | 9.85E-03 |
| *EPHB4* | *RP5-1021I20.1* | 0.37 | 3.04E-03 |
| *EPHB4* | *RP5-1024G6.7* | 0.35 | 5.70E-03 |
| *EPHB4* | *RP5-1050D4.5* | 0.34 | 6.50E-03 |
| *EPHB4* | *RP5-1057I20.4* | 0.36 | 3.89E-03 |
| *EPHB4* | *RP5-1065J22.8* | 0.36 | 3.84E-03 |
| *EPHB4* | *RP5-1071N3.1* | 0.30 | 1.72E-02 |
| *EPHB4* | *RP5-1198O20.4* | 0.32 | 1.11E-02 |
| *EPHB4* | *RP5-821D11.7* | 0.30 | 1.63E-02 |
| *EPHB4* | *RP5-823G15.5* | 0.46 | 1.96E-04 |
| *EPHB4* | *RP5-827C21.4* | 0.33 | 7.96E-03 |
| *EPHB4* | *RP5-858L17.1* | 0.36 | 3.81E-03 |
| *EPHB4* | *RP5-864K19.4* | 0.55 | 2.86E-06 |
| *EPHB4* | *RP5-882C2.2* | 0.32 | 1.05E-02 |
| *EPHB4* | *RP5-894A10.2* | 0.31 | 1.55E-02 |
| *EPHB4* | *RP5-908M14.9* | 0.35 | 5.80E-03 |
| *EPHB4* | *RP5-965G21.3* | 0.30 | 1.69E-02 |
| *EPHB4* | *RP5-966M1.6* | 0.30 | 1.71E-02 |
| *EPHB4* | *RP6-109B7.4* | 0.35 | 5.34E-03 |
| *EPHB4* | *RPAP1* | 0.36 | 4.19E-03 |
| *EPHB4* | *RPAP2* | 0.42 | 6.18E-04 |
| *EPHB4* | *RPL29* | -0.32 | 1.10E-02 |
| *EPHB4* | *RPL32* | -0.47 | 1.21E-04 |
| *EPHB4* | *RPL39* | -0.31 | 1.35E-02 |
| *EPHB4* | *RPN1* | 0.31 | 1.52E-02 |
| *EPHB4* | *RPPH1* | 0.36 | 4.17E-03 |
| *EPHB4* | *RPS14* | -0.31 | 1.53E-02 |
| *EPHB4* | *RPS25* | -0.35 | 4.72E-03 |
| *EPHB4* | *RPS6KA1* | 0.34 | 6.11E-03 |
| *EPHB4* | *RPS6KA2* | 0.31 | 1.31E-02 |
| *EPHB4* | *RPSA* | -0.32 | 1.11E-02 |
| *EPHB4* | *RPTOR* | 0.30 | 1.60E-02 |
| *EPHB4* | *RREB1* | 0.36 | 3.77E-03 |
| *EPHB4* | *RSBN1L* | 0.45 | 2.67E-04 |
| *EPHB4* | *RSL24D1* | -0.31 | 1.28E-02 |
| *EPHB4* | *RSRP1* | 0.39 | 1.86E-03 |
| *EPHB4* | *RTEL1* | 0.41 | 9.27E-04 |
| *EPHB4* | *RTF1* | 0.32 | 1.11E-02 |
| *EPHB4* | *RTN4RL1* | 0.44 | 3.95E-04 |
| *EPHB4* | *RUNX3* | 0.35 | 4.84E-03 |
| *EPHB4* | *RXRA* | 0.31 | 1.53E-02 |
| *EPHB4* | *RXRB* | 0.33 | 8.84E-03 |
| *EPHB4* | *RYK* | 0.37 | 2.96E-03 |
| *EPHB4* | *S100PBP* | 0.36 | 4.26E-03 |
| *EPHB4* | *S1PR4* | 0.31 | 1.28E-02 |
| *EPHB4* | *SAA2* | 0.33 | 9.77E-03 |
| *EPHB4* | *SAA2-SAA4* | 0.35 | 5.03E-03 |
| *EPHB4* | *SAC3D1* | 0.31 | 1.45E-02 |
| *EPHB4* | *SALL4* | 0.46 | 1.66E-04 |
| *EPHB4* | *SAMD1* | 0.38 | 2.22E-03 |
| *EPHB4* | *SAP25* | 0.30 | 1.70E-02 |
| *EPHB4* | *SART3* | 0.44 | 3.59E-04 |
| *EPHB4* | *SAT2* | 0.31 | 1.48E-02 |
| *EPHB4* | *SAXO2* | 0.40 | 1.46E-03 |
| *EPHB4* | *SBF1* | 0.34 | 6.46E-03 |
| *EPHB4* | *SBF2* | 0.31 | 1.40E-02 |
| *EPHB4* | *SBK1* | 0.37 | 3.13E-03 |
| *EPHB4* | *SCAF1* | 0.54 | 5.37E-06 |
| *EPHB4* | *SCARF1* | 0.30 | 1.74E-02 |
| *EPHB4* | *SCARNA2* | 0.37 | 3.34E-03 |
| *EPHB4* | *SCLY* | 0.33 | 8.00E-03 |
| *EPHB4* | *SCMH1* | 0.49 | 5.89E-05 |
| *EPHB4* | *SCML2* | 0.44 | 4.01E-04 |
| *EPHB4* | *SCN8A* | 0.49 | 5.66E-05 |
| *EPHB4* | *SCNN1G* | 0.47 | 1.32E-04 |
| *EPHB4* | *SDF2* | 0.44 | 3.04E-04 |
| *EPHB4* | *SDK1* | 0.37 | 2.91E-03 |
| *EPHB4* | *SDK2* | 0.44 | 3.82E-04 |
| *EPHB4* | *SDR42E2* | 0.30 | 1.70E-02 |
| *EPHB4* | *SEC31B* | 0.51 | 2.36E-05 |
| *EPHB4* | *SEL1L* | 0.31 | 1.35E-02 |
| *EPHB4* | *SELE* | 0.33 | 9.37E-03 |
| *EPHB4* | *SELP* | 0.33 | 8.09E-03 |
| *EPHB4* | *SEMA4A* | 0.33 | 8.43E-03 |
| *EPHB4* | *SEMA4C* | 0.43 | 4.66E-04 |
| *EPHB4* | *SEMA4F* | 0.51 | 2.39E-05 |
| *EPHB4* | *SENP3* | 0.42 | 6.32E-04 |
| *EPHB4* | *SENP8* | 0.40 | 1.26E-03 |
| *EPHB4* | *SEPN1* | 0.49 | 6.02E-05 |
| *EPHB4* | *SEPSECS* | 0.37 | 2.68E-03 |
| *EPHB4* | *SEPT7-AS1* | 0.45 | 2.80E-04 |
| *EPHB4* | *9-Sep* | 0.42 | 6.12E-04 |
| *EPHB4* | *SERPINE1* | 0.38 | 2.08E-03 |
| *EPHB4* | *SETD2* | 0.32 | 1.01E-02 |
| *EPHB4* | *SETDB1* | 0.32 | 1.24E-02 |
| *EPHB4* | *SF3B3* | 0.31 | 1.35E-02 |
| *EPHB4* | *SFI1* | 0.34 | 7.41E-03 |
| *EPHB4* | *SFTPD* | 0.41 | 1.07E-03 |
| *EPHB4* | *SGIP1* | 0.38 | 2.16E-03 |
| *EPHB4* | *SGK223* | 0.33 | 8.91E-03 |
| *EPHB4* | *SGK494* | 0.40 | 1.36E-03 |
| *EPHB4* | *SGPL1* | 0.42 | 6.82E-04 |
| *EPHB4* | *SGSH* | 0.52 | 1.50E-05 |
| *EPHB4* | *SGSM2* | 0.43 | 5.56E-04 |
| *EPHB4* | *SH2B3* | 0.35 | 5.29E-03 |
| *EPHB4* | *SH2D3C* | 0.40 | 1.13E-03 |
| *EPHB4* | *SH3BP2* | 0.32 | 1.02E-02 |
| *EPHB4* | *SH3BP5L* | 0.41 | 8.67E-04 |
| *EPHB4* | *SH3PXD2B* | 0.38 | 2.15E-03 |
| *EPHB4* | *SH3RF1* | 0.32 | 1.21E-02 |
| *EPHB4* | *SH3RF3* | 0.37 | 2.83E-03 |
| *EPHB4* | *SHANK2* | 0.40 | 1.10E-03 |
| *EPHB4* | *SHANK3* | 0.47 | 1.12E-04 |
| *EPHB4* | *SHISA2* | 0.45 | 2.54E-04 |
| *EPHB4* | *SHPK* | 0.33 | 8.92E-03 |
| *EPHB4* | *SHPRH* | 0.36 | 4.32E-03 |
| *EPHB4* | *SHROOM1* | 0.31 | 1.56E-02 |
| *EPHB4* | *SHROOM2* | 0.41 | 8.28E-04 |
| *EPHB4* | *SIN3A* | 0.38 | 2.01E-03 |
| *EPHB4* | *SIN3B* | 0.32 | 1.07E-02 |
| *EPHB4* | *SIPA1L3* | 0.46 | 1.67E-04 |
| *EPHB4* | *SIRT1* | 0.31 | 1.56E-02 |
| *EPHB4* | *SIRT3* | 0.30 | 1.78E-02 |
| *EPHB4* | *SIRT4* | 0.33 | 8.22E-03 |
| *EPHB4* | *SIRT6* | 0.34 | 6.77E-03 |
| *EPHB4* | *SIX2* | 0.33 | 9.32E-03 |
| *EPHB4* | *SIX5* | 0.42 | 6.63E-04 |
| *EPHB4* | *SKI* | 0.32 | 1.16E-02 |
| *EPHB4* | *SKIL* | 0.40 | 1.40E-03 |
| *EPHB4* | *SKIV2L* | 0.43 | 5.74E-04 |
| *EPHB4* | *SLC10A7* | 0.43 | 4.39E-04 |
| *EPHB4* | *SLC11A2* | 0.33 | 7.83E-03 |
| *EPHB4* | *SLC12A7* | 0.37 | 3.08E-03 |
| *EPHB4* | *SLC12A9* | 0.64 | 1.57E-08 |
| *EPHB4* | *SLC15A4* | 0.39 | 1.69E-03 |
| *EPHB4* | *SLC16A1-AS1* | 0.39 | 1.99E-03 |
| *EPHB4* | *SLC16A10* | 0.43 | 4.80E-04 |
| *EPHB4* | *SLC17A7* | 0.40 | 1.34E-03 |
| *EPHB4* | *SLC1A4* | 0.35 | 5.99E-03 |
| *EPHB4* | *SLC22A1* | 0.34 | 7.37E-03 |
| *EPHB4* | *SLC23A3* | 0.41 | 8.85E-04 |
| *EPHB4* | *SLC25A13* | 0.37 | 2.69E-03 |
| *EPHB4* | *SLC25A29* | 0.39 | 1.74E-03 |
| *EPHB4* | *SLC25A35* | 0.43 | 4.60E-04 |
| *EPHB4* | *SLC25A40* | 0.40 | 1.14E-03 |
| *EPHB4* | *SLC25A5* | -0.33 | 7.84E-03 |
| *EPHB4* | *SLC26A11* | 0.38 | 2.62E-03 |
| *EPHB4* | *SLC27A1* | 0.33 | 8.18E-03 |
| *EPHB4* | *SLC30A7* | 0.33 | 9.46E-03 |
| *EPHB4* | *SLC35A5* | 0.31 | 1.41E-02 |
| *EPHB4* | *SLC35B4* | 0.35 | 5.35E-03 |
| *EPHB4* | *SLC35E2B* | 0.36 | 4.56E-03 |
| *EPHB4* | *SLC35E3* | 0.32 | 1.18E-02 |
| *EPHB4* | *SLC38A10* | 0.46 | 1.47E-04 |
| *EPHB4* | *SLC38A4* | 0.31 | 1.53E-02 |
| *EPHB4* | *SLC39A11* | 0.35 | 5.90E-03 |
| *EPHB4* | *SLC39A4* | -0.35 | 5.60E-03 |
| *EPHB4* | *SLC41A1* | 0.48 | 7.68E-05 |
| *EPHB4* | *SLC41A3* | 0.32 | 1.07E-02 |
| *EPHB4* | *SLC43A2* | 0.34 | 7.70E-03 |
| *EPHB4* | *SLC47A2* | 0.31 | 1.55E-02 |
| *EPHB4* | *SLC4A3* | 0.41 | 9.54E-04 |
| *EPHB4* | *SLC52A1* | 0.39 | 2.00E-03 |
| *EPHB4* | *SLC5A12* | 0.36 | 4.41E-03 |
| *EPHB4* | *SLC6A16* | 0.46 | 1.88E-04 |
| *EPHB4* | *SLC7A5* | 0.31 | 1.53E-02 |
| *EPHB4* | *SLC8B1* | 0.35 | 5.99E-03 |
| *EPHB4* | *SLC9A6* | 0.39 | 1.76E-03 |
| *EPHB4* | *SLCO1A2* | 0.32 | 1.10E-02 |
| *EPHB4* | *SLCO2A1* | 0.43 | 5.70E-04 |
| *EPHB4* | *SLX1A* | 0.42 | 6.65E-04 |
| *EPHB4* | *SLX4* | 0.34 | 7.20E-03 |
| *EPHB4* | *SMAD1* | 0.35 | 5.32E-03 |
| *EPHB4* | *SMARCA2* | 0.42 | 6.48E-04 |
| *EPHB4* | *SMARCA5* | 0.32 | 1.26E-02 |
| *EPHB4* | *SMARCAL1* | 0.31 | 1.54E-02 |
| *EPHB4* | *SMARCC2* | 0.30 | 1.71E-02 |
| *EPHB4* | *SMC6* | 0.30 | 1.64E-02 |
| *EPHB4* | *SMG1* | 0.33 | 7.90E-03 |
| *EPHB4* | *SMG5* | 0.34 | 7.76E-03 |
| *EPHB4* | *SMG6* | 0.42 | 6.52E-04 |
| *EPHB4* | *SMIM10L2A* | 0.37 | 2.83E-03 |
| *EPHB4* | *SMYD4* | 0.37 | 3.45E-03 |
| *EPHB4* | *SNAI1* | 0.35 | 5.60E-03 |
| *EPHB4* | *SNAI3-AS1* | 0.41 | 8.69E-04 |
| *EPHB4* | *SNAPC4* | 0.41 | 9.12E-04 |
| *EPHB4* | *SNPH* | 0.36 | 4.28E-03 |
| *EPHB4* | *SNRNP200* | 0.32 | 1.23E-02 |
| *EPHB4* | *SNRNP35* | 0.37 | 2.95E-03 |
| *EPHB4* | *SNRNP70* | 0.41 | 9.43E-04 |
| *EPHB4* | *SNTG2* | 0.39 | 1.69E-03 |
| *EPHB4* | *SNX22* | 0.31 | 1.44E-02 |
| *EPHB4* | *SNX32* | 0.37 | 3.44E-03 |
| *EPHB4* | *SOCS1* | 0.48 | 7.81E-05 |
| *EPHB4* | *SOCS7* | 0.48 | 8.60E-05 |
| *EPHB4* | *SOGA1* | 0.45 | 2.87E-04 |
| *EPHB4* | *SORBS1* | 0.43 | 4.86E-04 |
| *EPHB4* | *SORCS2* | 0.30 | 1.66E-02 |
| *EPHB4* | *SOX12* | 0.44 | 3.02E-04 |
| *EPHB4* | *SOX6* | 0.34 | 7.02E-03 |
| *EPHB4* | *SOX8* | 0.33 | 9.32E-03 |
| *EPHB4* | *SOX9* | 0.37 | 3.19E-03 |
| *EPHB4* | *SP2-AS1* | 0.44 | 3.44E-04 |
| *EPHB4* | *SP6* | 0.30 | 1.62E-02 |
| *EPHB4* | *SPAST* | 0.49 | 4.70E-05 |
| *EPHB4* | *SPATA12* | 0.31 | 1.48E-02 |
| *EPHB4* | *SPATA20* | 0.34 | 6.11E-03 |
| *EPHB4* | *SPATA21* | 0.31 | 1.31E-02 |
| *EPHB4* | *SPATA33* | 0.40 | 1.22E-03 |
| *EPHB4* | *SPATS2* | 0.31 | 1.27E-02 |
| *EPHB4* | *SPCS1* | -0.36 | 4.36E-03 |
| *EPHB4* | *SPDYE5* | 0.35 | 4.95E-03 |
| *EPHB4* | *SPECC1L* | 0.32 | 1.16E-02 |
| *EPHB4* | *SPEG* | 0.41 | 9.90E-04 |
| *EPHB4* | *SPEN* | 0.40 | 1.34E-03 |
| *EPHB4* | *SPHK1* | 0.33 | 8.90E-03 |
| *EPHB4* | *SPHK2* | 0.30 | 1.66E-02 |
| *EPHB4* | *SPOCK2* | 0.35 | 5.53E-03 |
| *EPHB4* | *SPON1* | 0.31 | 1.45E-02 |
| *EPHB4* | *SPPL2B* | 0.37 | 2.92E-03 |
| *EPHB4* | *SPRED2* | 0.33 | 9.82E-03 |
| *EPHB4* | *SPRY4* | 0.38 | 2.06E-03 |
| *EPHB4* | *SPTAN1* | 0.30 | 1.67E-02 |
| *EPHB4* | *SPTBN5* | 0.34 | 6.60E-03 |
| *EPHB4* | *SPTY2D1* | 0.34 | 6.33E-03 |
| *EPHB4* | *SRC* | 0.32 | 1.05E-02 |
| *EPHB4* | *SRCAP* | 0.42 | 6.03E-04 |
| *EPHB4* | *SRGAP2B* | 0.32 | 1.25E-02 |
| *EPHB4* | *SRR* | 0.34 | 6.34E-03 |
| *EPHB4* | *SRRM2-AS1* | 0.34 | 6.78E-03 |
| *EPHB4* | *SRRT* | 0.54 | 6.61E-06 |
| *EPHB4* | *SRSF10* | 0.30 | 1.64E-02 |
| *EPHB4* | *SRSF11* | 0.40 | 1.35E-03 |
| *EPHB4* | *SRSF2* | 0.30 | 1.62E-02 |
| *EPHB4* | *SRSF4* | 0.31 | 1.41E-02 |
| *EPHB4* | *SRSF6* | 0.32 | 1.23E-02 |
| *EPHB4* | *SRSF7* | 0.33 | 9.22E-03 |
| *EPHB4* | *SSBP3* | 0.32 | 1.10E-02 |
| *EPHB4* | *SSH1* | 0.31 | 1.58E-02 |
| *EPHB4* | *ST3GAL2* | 0.46 | 1.89E-04 |
| *EPHB4* | *ST3GAL3* | 0.40 | 1.41E-03 |
| *EPHB4* | *ST3GAL5* | 0.35 | 4.67E-03 |
| *EPHB4* | *ST5* | 0.39 | 1.64E-03 |
| *EPHB4* | *ST6GAL1* | 0.31 | 1.30E-02 |
| *EPHB4* | *ST8SIA1* | 0.32 | 1.14E-02 |
| *EPHB4* | *STAC3* | 0.32 | 1.11E-02 |
| *EPHB4* | *STARD3* | 0.35 | 5.69E-03 |
| *EPHB4* | *STARD4-AS1* | 0.42 | 6.86E-04 |
| *EPHB4* | *STARD9* | 0.35 | 5.47E-03 |
| *EPHB4* | *STAT2* | 0.36 | 3.97E-03 |
| *EPHB4* | *STAT3* | 0.38 | 2.49E-03 |
| *EPHB4* | *STAT6* | 0.37 | 2.78E-03 |
| *EPHB4* | *STEAP2* | 0.32 | 1.17E-02 |
| *EPHB4* | *STIM1* | 0.31 | 1.33E-02 |
| *EPHB4* | *STK11IP* | 0.47 | 1.32E-04 |
| *EPHB4* | *STK19* | 0.35 | 5.34E-03 |
| *EPHB4* | *STK25* | 0.39 | 1.65E-03 |
| *EPHB4* | *STK36* | 0.50 | 3.79E-05 |
| *EPHB4* | *STK38L* | 0.37 | 3.49E-03 |
| *EPHB4* | *STKLD1* | 0.38 | 2.36E-03 |
| *EPHB4* | *STOML1* | 0.31 | 1.54E-02 |
| *EPHB4* | *STRADA* | 0.44 | 3.48E-04 |
| *EPHB4* | *STRIP1* | 0.42 | 7.29E-04 |
| *EPHB4* | *STRN4* | 0.31 | 1.57E-02 |
| *EPHB4* | *STX16* | 0.32 | 1.14E-02 |
| *EPHB4* | *STX1B* | 0.36 | 4.53E-03 |
| *EPHB4* | *STXBP4* | 0.36 | 3.59E-03 |
| *EPHB4* | *SUCLG2* | -0.40 | 1.18E-03 |
| *EPHB4* | *SUGP1* | 0.32 | 1.14E-02 |
| *EPHB4* | *SUGP2* | 0.40 | 1.45E-03 |
| *EPHB4* | *SULF2* | 0.39 | 1.98E-03 |
| *EPHB4* | *SUN2* | 0.34 | 6.57E-03 |
| *EPHB4* | *SUPT6H* | 0.49 | 4.32E-05 |
| *EPHB4* | *SUPT7L* | 0.36 | 4.06E-03 |
| *EPHB4* | *SUSD6* | 0.34 | 7.65E-03 |
| *EPHB4* | *SV2A* | 0.34 | 6.35E-03 |
| *EPHB4* | *SWT1* | 0.50 | 3.10E-05 |
| *EPHB4* | *SYCP2L* | 0.37 | 3.46E-03 |
| *EPHB4* | *SYCP3* | 0.42 | 7.96E-04 |
| *EPHB4* | *SYMPK* | 0.49 | 5.61E-05 |
| *EPHB4* | *SYNE2* | 0.34 | 6.93E-03 |
| *EPHB4* | *SYNGAP1* | 0.59 | 5.19E-07 |
| *EPHB4* | *SYNJ2BP* | 0.35 | 5.05E-03 |
| *EPHB4* | *SYNRG* | 0.32 | 1.04E-02 |
| *EPHB4* | *SYP* | 0.31 | 1.38E-02 |
| *EPHB4* | *SYT14* | 0.38 | 2.09E-03 |
| *EPHB4* | *SZT2* | 0.38 | 2.26E-03 |
| *EPHB4* | *TAB1* | 0.40 | 1.35E-03 |
| *EPHB4* | *TAF1C* | 0.36 | 3.64E-03 |
| *EPHB4* | *TAF4* | 0.30 | 1.67E-02 |
| *EPHB4* | *TAF6* | 0.36 | 4.13E-03 |
| *EPHB4* | *TANC1* | 0.35 | 4.95E-03 |
| *EPHB4* | *TANGO6* | 0.39 | 1.53E-03 |
| *EPHB4* | *TAOK1* | 0.31 | 1.53E-02 |
| *EPHB4* | *TAOK2* | 0.31 | 1.52E-02 |
| *EPHB4* | *TAPBPL* | 0.33 | 9.57E-03 |
| *EPHB4* | *TAPT1-AS1* | 0.32 | 1.05E-02 |
| *EPHB4* | *TARBP1* | 0.41 | 8.89E-04 |
| *EPHB4* | *TAS2R14* | 0.49 | 5.97E-05 |
| *EPHB4* | *TATDN1* | -0.36 | 3.96E-03 |
| *EPHB4* | *TBC1D10B* | 0.33 | 9.07E-03 |
| *EPHB4* | *TBC1D16* | 0.45 | 2.38E-04 |
| *EPHB4* | *TBC1D17* | 0.41 | 9.81E-04 |
| *EPHB4* | *TBC1D20* | 0.32 | 1.15E-02 |
| *EPHB4* | *TBC1D24* | 0.30 | 1.71E-02 |
| *EPHB4* | *TBC1D2B* | 0.36 | 4.01E-03 |
| *EPHB4* | *TBC1D32* | 0.42 | 7.03E-04 |
| *EPHB4* | *TBC1D3L* | 0.40 | 1.38E-03 |
| *EPHB4* | *TBCD* | 0.34 | 7.05E-03 |
| *EPHB4* | *TBK1* | 0.35 | 6.03E-03 |
| *EPHB4* | *TBKBP1* | 0.36 | 3.98E-03 |
| *EPHB4* | *TCEB3* | 0.32 | 1.03E-02 |
| *EPHB4* | *TCF20* | 0.40 | 1.27E-03 |
| *EPHB4* | *TCF3* | 0.35 | 5.33E-03 |
| *EPHB4* | *TCF7* | 0.31 | 1.32E-02 |
| *EPHB4* | *TCHP* | 0.35 | 4.88E-03 |
| *EPHB4* | *TCTA* | 0.35 | 5.85E-03 |
| *EPHB4* | *TCTE3* | 0.33 | 9.74E-03 |
| *EPHB4* | *TCTEX1D2* | 0.33 | 9.79E-03 |
| *EPHB4* | *TCTN2* | 0.41 | 1.01E-03 |
| *EPHB4* | *TEAD1* | 0.32 | 1.02E-02 |
| *EPHB4* | *TEAD2* | 0.37 | 3.03E-03 |
| *EPHB4* | *TEAD3* | 0.35 | 4.80E-03 |
| *EPHB4* | *TECPR1* | 0.44 | 3.37E-04 |
| *EPHB4* | *TECPR2* | 0.32 | 1.24E-02 |
| *EPHB4* | *TEF* | 0.46 | 1.52E-04 |
| *EPHB4* | *TELO2* | 0.40 | 1.46E-03 |
| *EPHB4* | *TERC* | 0.33 | 8.23E-03 |
| *EPHB4* | *TET3* | 0.34 | 7.09E-03 |
| *EPHB4* | *TEX2* | 0.34 | 7.50E-03 |
| *EPHB4* | *TFAP2A-AS1* | 0.34 | 6.79E-03 |
| *EPHB4* | *TFCP2* | 0.33 | 9.75E-03 |
| *EPHB4* | *TFPT* | 0.38 | 2.63E-03 |
| *EPHB4* | *TGFB3* | 0.31 | 1.32E-02 |
| *EPHB4* | *TGIF2* | 0.35 | 5.45E-03 |
| *EPHB4* | *THADA* | 0.43 | 4.19E-04 |
| *EPHB4* | *THAP5* | 0.50 | 3.39E-05 |
| *EPHB4* | *THBS1* | 0.34 | 6.75E-03 |
| *EPHB4* | *THOC7* | -0.44 | 3.38E-04 |
| *EPHB4* | *THRA* | 0.45 | 2.24E-04 |
| *EPHB4* | *TIAF1* | 0.38 | 2.18E-03 |
| *EPHB4* | *TIAM2* | 0.34 | 7.70E-03 |
| *EPHB4* | *TIGD1* | 0.36 | 3.55E-03 |
| *EPHB4* | *TIGD3* | 0.30 | 1.76E-02 |
| *EPHB4* | *TIGD4* | 0.34 | 6.41E-03 |
| *EPHB4* | *TIGD5* | 0.30 | 1.65E-02 |
| *EPHB4* | *TIMM10B.1* | 0.49 | 5.39E-05 |
| *EPHB4* | *TLE2* | 0.33 | 7.79E-03 |
| *EPHB4* | *TLE3* | 0.40 | 1.26E-03 |
| *EPHB4* | *TM4SF18* | 0.31 | 1.57E-02 |
| *EPHB4* | *TMED8* | 0.37 | 3.25E-03 |
| *EPHB4* | *TMEM104* | 0.51 | 2.36E-05 |
| *EPHB4* | *TMEM106A* | 0.34 | 6.91E-03 |
| *EPHB4* | *TMEM120B* | 0.36 | 4.19E-03 |
| *EPHB4* | *TMEM130* | 0.34 | 7.29E-03 |
| *EPHB4* | *TMEM131* | 0.32 | 1.08E-02 |
| *EPHB4* | *TMEM132A* | 0.40 | 1.44E-03 |
| *EPHB4* | *TMEM143* | 0.43 | 5.10E-04 |
| *EPHB4* | *TMEM150C* | 0.30 | 1.74E-02 |
| *EPHB4* | *TMEM161B* | 0.33 | 8.92E-03 |
| *EPHB4* | *TMEM168* | 0.35 | 5.75E-03 |
| *EPHB4* | *TMEM178B* | 0.32 | 1.03E-02 |
| *EPHB4* | *TMEM181* | 0.34 | 6.57E-03 |
| *EPHB4* | *TMEM184B* | 0.47 | 1.16E-04 |
| *EPHB4* | *TMEM201* | 0.38 | 2.46E-03 |
| *EPHB4* | *TMEM220-AS1* | 0.34 | 6.63E-03 |
| *EPHB4* | *TMEM229B* | 0.32 | 1.13E-02 |
| *EPHB4* | *TMEM256-PLSCR3.1* | 0.30 | 1.64E-02 |
| *EPHB4* | *TMEM63C* | 0.34 | 7.63E-03 |
| *EPHB4* | *TMEM67* | 0.34 | 7.03E-03 |
| *EPHB4* | *TMEM81* | 0.31 | 1.39E-02 |
| *EPHB4* | *TMEM86B* | 0.33 | 9.22E-03 |
| *EPHB4* | *TMEM88* | 0.30 | 1.76E-02 |
| *EPHB4* | *TMEM94* | 0.51 | 2.45E-05 |
| *EPHB4* | *TMEM9B-AS1* | 0.30 | 1.64E-02 |
| *EPHB4* | *TMTC3* | 0.35 | 5.49E-03 |
| *EPHB4* | *TMUB2* | 0.35 | 4.87E-03 |
| *EPHB4* | *TNC* | 0.34 | 6.32E-03 |
| *EPHB4* | *TNFRSF10D* | 0.36 | 3.89E-03 |
| *EPHB4* | *TNFRSF1A* | 0.30 | 1.73E-02 |
| *EPHB4* | *TNFRSF25* | 0.47 | 1.09E-04 |
| *EPHB4* | *TNK2* | 0.43 | 4.28E-04 |
| *EPHB4* | *TNKS2* | 0.32 | 1.12E-02 |
| *EPHB4* | *TNNI2* | 0.42 | 7.08E-04 |
| *EPHB4* | *TNPO2* | 0.45 | 2.67E-04 |
| *EPHB4* | *TNRC18* | 0.47 | 1.09E-04 |
| *EPHB4* | *TNRC6C* | 0.30 | 1.67E-02 |
| *EPHB4* | *TOP3A* | 0.34 | 6.24E-03 |
| *EPHB4* | *TOP3B* | 0.40 | 1.22E-03 |
| *EPHB4* | *TOR2A* | 0.31 | 1.49E-02 |
| *EPHB4* | *TP53BP1* | 0.44 | 3.02E-04 |
| *EPHB4* | *TP53TG1* | 0.34 | 6.92E-03 |
| *EPHB4* | *TP63* | 0.36 | 3.92E-03 |
| *EPHB4* | *TP73* | 0.51 | 2.67E-05 |
| *EPHB4* | *TPCN2* | 0.38 | 2.56E-03 |
| *EPHB4* | *TPRA1* | 0.32 | 1.03E-02 |
| *EPHB4* | *TRAF1* | 0.30 | 1.73E-02 |
| *EPHB4* | *TRAF3* | 0.33 | 8.24E-03 |
| *EPHB4* | *TRAF3IP1* | 0.34 | 6.42E-03 |
| *EPHB4* | *TRAF3IP2* | 0.31 | 1.49E-02 |
| *EPHB4* | *TRAF3IP2-AS1* | 0.37 | 3.01E-03 |
| *EPHB4* | *TRAK2* | 0.38 | 2.50E-03 |
| *EPHB4* | *TRAM2* | 0.43 | 4.23E-04 |
| *EPHB4* | *TRANK1* | 0.32 | 1.09E-02 |
| *EPHB4* | *TRAPPC11* | 0.44 | 3.80E-04 |
| *EPHB4* | *TRIB2* | 0.42 | 7.93E-04 |
| *EPHB4* | *TRIM3* | 0.41 | 8.95E-04 |
| *EPHB4* | *TRIM37* | 0.32 | 1.25E-02 |
| *EPHB4* | *TRIM39* | 0.30 | 1.70E-02 |
| *EPHB4* | *TRIM4* | 0.52 | 1.29E-05 |
| *EPHB4* | *TRIM44* | 0.42 | 5.95E-04 |
| *EPHB4* | *TRIM45* | 0.43 | 5.47E-04 |
| *EPHB4* | *TRIM47* | 0.35 | 4.70E-03 |
| *EPHB4* | *TRIM56* | 0.45 | 2.84E-04 |
| *EPHB4* | *TRIM65* | 0.50 | 3.38E-05 |
| *EPHB4* | *TRIM68* | 0.44 | 3.00E-04 |
| *EPHB4* | *TRIM72* | 0.37 | 3.29E-03 |
| *EPHB4* | *TRIP11* | 0.36 | 3.63E-03 |
| *EPHB4* | *TRIP12* | 0.42 | 7.37E-04 |
| *EPHB4* | *TRIP6* | 0.50 | 3.10E-05 |
| *EPHB4* | *TRMT1* | 0.33 | 8.05E-03 |
| *EPHB4* | *TRPM4* | 0.31 | 1.45E-02 |
| *EPHB4* | *TRRAP* | 0.56 | 2.69E-06 |
| *EPHB4* | *TSC2* | 0.31 | 1.46E-02 |
| *EPHB4* | *TSC22D1-AS1* | 0.39 | 1.62E-03 |
| *EPHB4* | *TSEN54* | 0.40 | 1.20E-03 |
| *EPHB4* | *TSLP* | 0.33 | 8.52E-03 |
| *EPHB4* | *TSNAXIP1* | 0.30 | 1.64E-02 |
| *EPHB4* | *TSPAN10* | 0.36 | 3.95E-03 |
| *EPHB4* | *TSPAN9* | 0.37 | 3.31E-03 |
| *EPHB4* | *TSPYL4* | 0.31 | 1.31E-02 |
| *EPHB4* | *TSSC4* | 0.30 | 1.60E-02 |
| *EPHB4* | *TSTD2* | 0.32 | 1.10E-02 |
| *EPHB4* | *TTC1* | -0.31 | 1.31E-02 |
| *EPHB4* | *TTC17* | 0.33 | 9.17E-03 |
| *EPHB4* | *TTC21A* | 0.39 | 1.88E-03 |
| *EPHB4* | *TTC30A* | 0.33 | 7.80E-03 |
| *EPHB4* | *TTC30B* | 0.35 | 5.36E-03 |
| *EPHB4* | *TTC32* | 0.41 | 1.05E-03 |
| *EPHB4* | *TTC37* | 0.38 | 2.45E-03 |
| *EPHB4* | *TTC39C* | 0.31 | 1.54E-02 |
| *EPHB4* | *TTLL10* | 0.35 | 5.37E-03 |
| *EPHB4* | *TTLL5* | 0.49 | 4.64E-05 |
| *EPHB4* | *TTN* | 0.37 | 3.30E-03 |
| *EPHB4* | *TTYH3* | 0.40 | 1.10E-03 |
| *EPHB4* | *TUBGCP6* | 0.39 | 1.83E-03 |
| *EPHB4* | *TULP3* | 0.32 | 1.12E-02 |
| *EPHB4* | *TULP4* | 0.37 | 2.95E-03 |
| *EPHB4* | *TVP23C* | 0.36 | 4.07E-03 |
| *EPHB4* | *TXNDC16* | 0.38 | 2.22E-03 |
| *EPHB4* | *TYK2* | 0.43 | 4.55E-04 |
| *EPHB4* | *TYSND1* | 0.31 | 1.46E-02 |
| *EPHB4* | *TYW1* | 0.34 | 6.18E-03 |
| *EPHB4* | *U73166.2* | 0.50 | 3.92E-05 |
| *EPHB4* | *UAP1L1* | 0.38 | 2.00E-03 |
| *EPHB4* | *UBAP1L* | 0.42 | 6.63E-04 |
| *EPHB4* | *UBAP2L* | 0.31 | 1.33E-02 |
| *EPHB4* | *UBE2O* | 0.35 | 5.69E-03 |
| *EPHB4* | *UBE2QL1* | 0.34 | 6.76E-03 |
| *EPHB4* | *UBE3B* | 0.31 | 1.40E-02 |
| *EPHB4* | *UBE4B* | 0.37 | 3.44E-03 |
| *EPHB4* | *UBR3* | 0.38 | 2.03E-03 |
| *EPHB4* | *UBR4* | 0.35 | 5.31E-03 |
| *EPHB4* | *UBR7* | 0.31 | 1.31E-02 |
| *EPHB4* | *UBTF* | 0.35 | 5.46E-03 |
| *EPHB4* | *UBXN11* | 0.44 | 3.52E-04 |
| *EPHB4* | *UCN2* | 0.46 | 1.56E-04 |
| *EPHB4* | *ULBP1* | 0.38 | 2.26E-03 |
| *EPHB4* | *ULBP3* | 0.31 | 1.36E-02 |
| *EPHB4* | *ULK2* | 0.32 | 1.22E-02 |
| *EPHB4* | *UNC119* | 0.39 | 1.75E-03 |
| *EPHB4* | *UNC119B* | 0.46 | 1.77E-04 |
| *EPHB4* | *UQCRB* | -0.38 | 2.56E-03 |
| *EPHB4* | *URGCP* | 0.41 | 1.00E-03 |
| *EPHB4* | *USP19* | 0.35 | 5.33E-03 |
| *EPHB4* | *USP20* | 0.33 | 9.28E-03 |
| *EPHB4* | *USP21* | 0.36 | 3.63E-03 |
| *EPHB4* | *USP22* | 0.33 | 7.78E-03 |
| *EPHB4* | *USP27X-AS1* | 0.33 | 7.86E-03 |
| *EPHB4* | *USP31* | 0.36 | 4.15E-03 |
| *EPHB4* | *USP32* | 0.33 | 9.01E-03 |
| *EPHB4* | *USP34* | 0.31 | 1.28E-02 |
| *EPHB4* | *USP36* | 0.35 | 5.29E-03 |
| *EPHB4* | *USP37* | 0.40 | 1.23E-03 |
| *EPHB4* | *USP38* | 0.37 | 3.29E-03 |
| *EPHB4* | *USP42* | 0.47 | 1.26E-04 |
| *EPHB4* | *USP46* | 0.32 | 1.23E-02 |
| *EPHB4* | *USP5* | 0.31 | 1.55E-02 |
| *EPHB4* | *UTP20* | 0.32 | 1.00E-02 |
| *EPHB4* | *VAC14* | 0.32 | 1.10E-02 |
| *EPHB4* | *VAMP1* | 0.33 | 8.57E-03 |
| *EPHB4* | *VAMP2* | 0.31 | 1.39E-02 |
| *EPHB4* | *VASH1* | 0.31 | 1.50E-02 |
| *EPHB4* | *VASH2* | 0.37 | 3.06E-03 |
| *EPHB4* | *VDR* | 0.31 | 1.43E-02 |
| *EPHB4* | *VIPR1* | 0.35 | 5.08E-03 |
| *EPHB4* | *VPS13D* | 0.32 | 1.25E-02 |
| *EPHB4* | *VPS37D* | 0.44 | 4.08E-04 |
| *EPHB4* | *VPS39* | 0.32 | 1.06E-02 |
| *EPHB4* | *VPS41* | 0.35 | 4.91E-03 |
| *EPHB4* | *VPS50* | 0.33 | 8.43E-03 |
| *EPHB4* | *VWCE* | 0.32 | 1.22E-02 |
| *EPHB4* | *WAPL* | 0.31 | 1.34E-02 |
| *EPHB4* | *WAS* | 0.30 | 1.59E-02 |
| *EPHB4* | *WASH1* | 0.33 | 7.80E-03 |
| *EPHB4* | *WBSCR17* | 0.30 | 1.76E-02 |
| *EPHB4* | *WBSCR22* | 0.30 | 1.72E-02 |
| *EPHB4* | *WDFY3* | 0.33 | 8.59E-03 |
| *EPHB4* | *WDR25* | 0.31 | 1.48E-02 |
| *EPHB4* | *WDR27* | 0.34 | 7.60E-03 |
| *EPHB4* | *WDR36* | 0.33 | 9.45E-03 |
| *EPHB4* | *WDR59* | 0.31 | 1.27E-02 |
| *EPHB4* | *WDR62* | 0.33 | 9.23E-03 |
| *EPHB4* | *WDR81* | 0.40 | 1.40E-03 |
| *EPHB4* | *WDR90* | 0.52 | 1.43E-05 |
| *EPHB4* | *WDR92* | 0.41 | 9.00E-04 |
| *EPHB4* | *WFIKKN1* | 0.41 | 9.00E-04 |
| *EPHB4* | *WFS1* | 0.30 | 1.61E-02 |
| *EPHB4* | *WHSC1* | 0.33 | 9.61E-03 |
| *EPHB4* | *WHSC1L1* | 0.33 | 9.82E-03 |
| *EPHB4* | *WNK2* | 0.31 | 1.49E-02 |
| *EPHB4* | *WNT10A* | 0.33 | 8.05E-03 |
| *EPHB4* | *WNT3* | 0.44 | 3.41E-04 |
| *EPHB4* | *WNT3A* | 0.47 | 1.14E-04 |
| *EPHB4* | *WNT4* | 0.38 | 2.20E-03 |
| *EPHB4* | *WNT5B* | 0.44 | 3.39E-04 |
| *EPHB4* | *WNT6* | 0.47 | 1.21E-04 |
| *EPHB4* | *WNT7B* | 0.31 | 1.28E-02 |
| *EPHB4* | *WRAP73* | 0.39 | 1.69E-03 |
| *EPHB4* | *WTAP* | 0.31 | 1.41E-02 |
| *EPHB4* | *WWTR1-AS1* | 0.31 | 1.46E-02 |
| *EPHB4* | *XKR6* | 0.30 | 1.62E-02 |
| *EPHB4* | *XPO1* | 0.43 | 4.44E-04 |
| *EPHB4* | *XPO6* | 0.36 | 4.11E-03 |
| *EPHB4* | *XRCC1* | 0.31 | 1.52E-02 |
| *EPHB4* | *XRCC3* | 0.42 | 7.79E-04 |
| *EPHB4* | *XX-FW83563B9.5* | 0.34 | 7.07E-03 |
| *EPHB4* | *XXbac-BPG181B23.7* | 0.39 | 1.85E-03 |
| *EPHB4* | *XXbac-BPG181M17.6* | 0.35 | 4.65E-03 |
| *EPHB4* | *XXbac-BPGBPG55C20.2* | 0.41 | 9.87E-04 |
| *EPHB4* | *XYLT1* | 0.32 | 1.15E-02 |
| *EPHB4* | *XYLT2* | 0.40 | 1.33E-03 |
| *EPHB4* | *YAF2* | 0.30 | 1.70E-02 |
| *EPHB4* | *YLPM1* | 0.34 | 7.29E-03 |
| *EPHB4* | *YME1L1* | 0.38 | 2.29E-03 |
| *EPHB4* | *YTHDC2* | 0.49 | 5.28E-05 |
| *EPHB4* | *YWHAZ* | -0.31 | 1.48E-02 |
| *EPHB4* | *ZACN* | 0.37 | 3.32E-03 |
| *EPHB4* | *ZBED1* | 0.40 | 1.41E-03 |
| *EPHB4* | *ZBED3* | 0.35 | 5.60E-03 |
| *EPHB4* | *ZBED6CL* | 0.38 | 2.34E-03 |
| *EPHB4* | *ZBED9* | 0.35 | 5.14E-03 |
| *EPHB4* | *ZBTB17* | 0.39 | 1.53E-03 |
| *EPHB4* | *ZBTB22* | 0.30 | 1.65E-02 |
| *EPHB4* | *ZBTB3* | 0.32 | 1.18E-02 |
| *EPHB4* | *ZBTB37* | 0.33 | 8.15E-03 |
| *EPHB4* | *ZBTB39* | 0.39 | 1.95E-03 |
| *EPHB4* | *ZBTB40* | 0.31 | 1.36E-02 |
| *EPHB4* | *ZBTB42* | 0.30 | 1.71E-02 |
| *EPHB4* | *ZBTB48* | 0.44 | 3.10E-04 |
| *EPHB4* | *ZBTB49* | 0.33 | 8.96E-03 |
| *EPHB4* | *ZBTB8A* | 0.37 | 3.52E-03 |
| *EPHB4* | *ZC3H18* | 0.33 | 8.42E-03 |
| *EPHB4* | *ZC3H4* | 0.43 | 4.18E-04 |
| *EPHB4* | *ZC3H7B* | 0.52 | 1.76E-05 |
| *EPHB4* | *ZCCHC4* | 0.32 | 1.00E-02 |
| *EPHB4* | *ZCCHC8* | 0.34 | 7.32E-03 |
| *EPHB4* | *ZCWPW1* | 0.41 | 9.44E-04 |
| *EPHB4* | *ZCWPW2* | 0.32 | 1.19E-02 |
| *EPHB4* | *ZDHHC11B* | 0.39 | 1.77E-03 |
| *EPHB4* | *ZDHHC13* | 0.34 | 7.06E-03 |
| *EPHB4* | *ZDHHC17* | 0.34 | 7.62E-03 |
| *EPHB4* | *ZDHHC8* | 0.49 | 5.74E-05 |
| *EPHB4* | *ZER1* | 0.39 | 1.73E-03 |
| *EPHB4* | *ZFAND4* | 0.38 | 2.20E-03 |
| *EPHB4* | *ZFHX2* | 0.30 | 1.61E-02 |
| *EPHB4* | *ZFP1* | 0.41 | 1.05E-03 |
| *EPHB4* | *ZFP37* | 0.30 | 1.67E-02 |
| *EPHB4* | *ZFP64* | 0.39 | 1.51E-03 |
| *EPHB4* | *ZFP69B* | 0.40 | 1.37E-03 |
| *EPHB4* | *ZFP90* | 0.43 | 4.79E-04 |
| *EPHB4* | *ZFX* | 0.33 | 9.63E-03 |
| *EPHB4* | *ZFYVE16* | 0.36 | 4.08E-03 |
| *EPHB4* | *ZFYVE26* | 0.36 | 3.88E-03 |
| *EPHB4* | *ZFYVE9* | 0.44 | 3.57E-04 |
| *EPHB4* | *ZGLP1* | 0.40 | 1.41E-03 |
| *EPHB4* | *ZGRF1* | 0.31 | 1.47E-02 |
| *EPHB4* | *ZKSCAN1* | 0.46 | 1.92E-04 |
| *EPHB4* | *ZKSCAN3* | 0.30 | 1.74E-02 |
| *EPHB4* | *ZKSCAN5* | 0.52 | 1.26E-05 |
| *EPHB4* | *ZKSCAN8* | 0.37 | 3.03E-03 |
| *EPHB4* | *ZMAT2* | -0.31 | 1.58E-02 |
| *EPHB4* | *ZMIZ1* | 0.32 | 1.14E-02 |
| *EPHB4* | *ZMIZ2* | 0.46 | 1.72E-04 |
| *EPHB4* | *ZMYM3* | 0.35 | 5.03E-03 |
| *EPHB4* | *ZMYM4* | 0.37 | 2.85E-03 |
| *EPHB4* | *ZMYM6* | 0.34 | 6.08E-03 |
| *EPHB4* | *ZMYND10* | 0.36 | 4.52E-03 |
| *EPHB4* | *ZMYND15* | 0.37 | 2.92E-03 |
| *EPHB4* | *ZNF106* | 0.33 | 9.61E-03 |
| *EPHB4* | *ZNF107* | 0.42 | 7.32E-04 |
| *EPHB4* | *ZNF133* | 0.35 | 4.78E-03 |
| *EPHB4* | *ZNF138* | 0.36 | 4.51E-03 |
| *EPHB4* | *ZNF142* | 0.30 | 1.62E-02 |
| *EPHB4* | *ZNF148* | 0.45 | 2.19E-04 |
| *EPHB4* | *ZNF160* | 0.30 | 1.70E-02 |
| *EPHB4* | *ZNF17* | 0.32 | 1.07E-02 |
| *EPHB4* | *ZNF184* | 0.38 | 2.19E-03 |
| *EPHB4* | *ZNF189* | 0.30 | 1.68E-02 |
| *EPHB4* | *ZNF197* | 0.30 | 1.72E-02 |
| *EPHB4* | *ZNF205* | 0.31 | 1.27E-02 |
| *EPHB4* | *ZNF219* | 0.43 | 5.73E-04 |
| *EPHB4* | *ZNF225* | 0.39 | 1.66E-03 |
| *EPHB4* | *ZNF227* | 0.32 | 1.05E-02 |
| *EPHB4* | *ZNF230* | 0.34 | 6.58E-03 |
| *EPHB4* | *ZNF232* | 0.30 | 1.61E-02 |
| *EPHB4* | *ZNF233* | 0.39 | 1.75E-03 |
| *EPHB4* | *ZNF236* | 0.38 | 2.25E-03 |
| *EPHB4* | *ZNF248* | 0.31 | 1.36E-02 |
| *EPHB4* | *ZNF26* | 0.35 | 5.62E-03 |
| *EPHB4* | *ZNF263* | 0.32 | 1.14E-02 |
| *EPHB4* | *ZNF264* | 0.30 | 1.61E-02 |
| *EPHB4* | *ZNF268* | 0.30 | 1.64E-02 |
| *EPHB4* | *ZNF273* | 0.37 | 3.08E-03 |
| *EPHB4* | *ZNF275* | 0.33 | 9.69E-03 |
| *EPHB4* | *ZNF276* | 0.39 | 1.58E-03 |
| *EPHB4* | *ZNF285* | 0.36 | 3.94E-03 |
| *EPHB4* | *ZNF286A* | 0.31 | 1.53E-02 |
| *EPHB4* | *ZNF286B* | 0.36 | 4.54E-03 |
| *EPHB4* | *ZNF3* | 0.42 | 7.34E-04 |
| *EPHB4* | *ZNF318* | 0.31 | 1.58E-02 |
| *EPHB4* | *ZNF322* | 0.31 | 1.55E-02 |
| *EPHB4* | *ZNF324* | 0.30 | 1.60E-02 |
| *EPHB4* | *ZNF331* | 0.30 | 1.60E-02 |
| *EPHB4* | *ZNF335* | 0.41 | 9.97E-04 |
| *EPHB4* | *ZNF337* | 0.36 | 4.52E-03 |
| *EPHB4* | *ZNF33A* | 0.31 | 1.34E-02 |
| *EPHB4* | *ZNF34* | 0.31 | 1.31E-02 |
| *EPHB4* | *ZNF362* | 0.40 | 1.23E-03 |
| *EPHB4* | *ZNF384* | 0.47 | 1.20E-04 |
| *EPHB4* | *ZNF385D* | 0.31 | 1.45E-02 |
| *EPHB4* | *ZNF395* | 0.31 | 1.54E-02 |
| *EPHB4* | *ZNF407* | 0.45 | 2.05E-04 |
| *EPHB4* | *ZNF419* | 0.36 | 4.62E-03 |
| *EPHB4* | *ZNF432* | 0.48 | 8.02E-05 |
| *EPHB4* | *ZNF436* | 0.33 | 8.39E-03 |
| *EPHB4* | *ZNF442* | 0.35 | 5.97E-03 |
| *EPHB4* | *ZNF446* | 0.35 | 5.77E-03 |
| *EPHB4* | *ZNF449* | 0.44 | 3.17E-04 |
| *EPHB4* | *ZNF45* | 0.38 | 2.63E-03 |
| *EPHB4* | *ZNF454* | 0.31 | 1.27E-02 |
| *EPHB4* | *ZNF462* | 0.43 | 4.48E-04 |
| *EPHB4* | *ZNF470* | 0.33 | 9.85E-03 |
| *EPHB4* | *ZNF480* | 0.40 | 1.22E-03 |
| *EPHB4* | *ZNF487* | 0.39 | 1.52E-03 |
| *EPHB4* | *ZNF490* | 0.44 | 3.35E-04 |
| *EPHB4* | *ZNF497* | 0.38 | 2.62E-03 |
| *EPHB4* | *ZNF500* | 0.43 | 4.73E-04 |
| *EPHB4* | *ZNF501* | 0.33 | 8.02E-03 |
| *EPHB4* | *ZNF510* | 0.33 | 8.09E-03 |
| *EPHB4* | *ZNF512B* | 0.45 | 2.45E-04 |
| *EPHB4* | *ZNF518B* | 0.30 | 1.75E-02 |
| *EPHB4* | *ZNF526* | 0.38 | 2.05E-03 |
| *EPHB4* | *ZNF532* | 0.40 | 1.37E-03 |
| *EPHB4* | *ZNF540* | 0.31 | 1.33E-02 |
| *EPHB4* | *ZNF543* | 0.34 | 6.09E-03 |
| *EPHB4* | *ZNF544* | 0.32 | 1.05E-02 |
| *EPHB4* | *ZNF546* | 0.35 | 5.50E-03 |
| *EPHB4* | *ZNF548* | 0.33 | 9.59E-03 |
| *EPHB4* | *ZNF550* | 0.34 | 6.55E-03 |
| *EPHB4* | *ZNF552* | 0.30 | 1.69E-02 |
| *EPHB4* | *ZNF558* | 0.35 | 5.80E-03 |
| *EPHB4* | *ZNF559-ZNF177* | 0.37 | 2.79E-03 |
| *EPHB4* | *ZNF569* | 0.34 | 7.06E-03 |
| *EPHB4* | *ZNF580* | 0.35 | 5.13E-03 |
| *EPHB4* | *ZNF587B* | 0.33 | 9.00E-03 |
| *EPHB4* | *ZNF594* | 0.34 | 7.41E-03 |
| *EPHB4* | *ZNF605* | 0.32 | 1.08E-02 |
| *EPHB4* | *ZNF609* | 0.35 | 5.22E-03 |
| *EPHB4* | *ZNF618* | 0.40 | 1.24E-03 |
| *EPHB4* | *ZNF621* | 0.32 | 1.04E-02 |
| *EPHB4* | *ZNF628* | 0.37 | 2.71E-03 |
| *EPHB4* | *ZNF629* | 0.50 | 3.03E-05 |
| *EPHB4* | *ZNF641* | 0.40 | 1.26E-03 |
| *EPHB4* | *ZNF646* | 0.37 | 2.97E-03 |
| *EPHB4* | *ZNF649* | 0.32 | 1.26E-02 |
| *EPHB4* | *ZNF652* | 0.34 | 7.67E-03 |
| *EPHB4* | *ZNF668* | 0.38 | 2.24E-03 |
| *EPHB4* | *ZNF670-ZNF695* | 0.36 | 3.73E-03 |
| *EPHB4* | *ZNF671* | 0.34 | 7.37E-03 |
| *EPHB4* | *ZNF680* | 0.36 | 3.81E-03 |
| *EPHB4* | *ZNF684* | 0.39 | 1.59E-03 |
| *EPHB4* | *ZNF687* | 0.36 | 4.42E-03 |
| *EPHB4* | *ZNF692* | 0.34 | 7.04E-03 |
| *EPHB4* | *ZNF696* | 0.34 | 6.42E-03 |
| *EPHB4* | *ZNF697* | 0.31 | 1.44E-02 |
| *EPHB4* | *ZNF699* | 0.33 | 8.32E-03 |
| *EPHB4* | *ZNF703* | 0.37 | 3.06E-03 |
| *EPHB4* | *ZNF713* | 0.39 | 1.59E-03 |
| *EPHB4* | *ZNF736* | 0.33 | 8.91E-03 |
| *EPHB4* | *ZNF740* | 0.46 | 1.75E-04 |
| *EPHB4* | *ZNF747* | 0.37 | 2.96E-03 |
| *EPHB4* | *ZNF76* | 0.35 | 5.58E-03 |
| *EPHB4* | *ZNF771* | 0.33 | 9.79E-03 |
| *EPHB4* | *ZNF776* | 0.47 | 1.38E-04 |
| *EPHB4* | *ZNF781* | 0.38 | 2.54E-03 |
| *EPHB4* | *ZNF782* | 0.35 | 5.69E-03 |
| *EPHB4* | *ZNF783* | 0.40 | 1.35E-03 |
| *EPHB4* | *ZNF784* | 0.31 | 1.56E-02 |
| *EPHB4* | *ZNF785* | 0.46 | 1.53E-04 |
| *EPHB4* | *ZNF789* | 0.38 | 2.27E-03 |
| *EPHB4* | *ZNF790-AS1* | 0.33 | 8.43E-03 |
| *EPHB4* | *ZNF81* | 0.30 | 1.77E-02 |
| *EPHB4* | *ZNF816* | 0.32 | 1.18E-02 |
| *EPHB4* | *ZNF827* | 0.39 | 1.73E-03 |
| *EPHB4* | *ZNF830* | 0.37 | 3.25E-03 |
| *EPHB4* | *ZNF836* | 0.32 | 1.25E-02 |
| *EPHB4* | *ZNF837* | 0.52 | 1.41E-05 |
| *EPHB4* | *ZNF839* | 0.47 | 1.23E-04 |
| *EPHB4* | *ZNF84* | 0.39 | 1.69E-03 |
| *EPHB4* | *ZNF843* | 0.39 | 1.82E-03 |
| *EPHB4* | *ZNF845* | 0.32 | 1.12E-02 |
| *EPHB4* | *ZNF862* | 0.34 | 7.59E-03 |
| *EPHB4* | *ZNF865* | 0.43 | 5.44E-04 |
| *EPHB4* | *ZNF879* | 0.39 | 1.62E-03 |
| *EPHB4* | *ZNF891* | 0.31 | 1.36E-02 |
| *EPHB4* | *ZNF92* | 0.37 | 3.13E-03 |
| *EPHB4* | *ZNRD1-AS1* | 0.34 | 6.53E-03 |
| *EPHB4* | *ZNRF3* | 0.36 | 3.72E-03 |
| *EPHB4* | *ZPR1* | -0.31 | 1.28E-02 |
| *EPHB4* | *ZRSR2* | 0.33 | 7.92E-03 |
| *EPHB4* | *ZSCAN20* | 0.36 | 4.58E-03 |
| *EPHB4* | *ZSCAN21* | 0.45 | 2.08E-04 |
| *EPHB4* | *ZSCAN22* | 0.36 | 4.41E-03 |
| *EPHB4* | *ZSCAN25* | 0.63 | 4.57E-08 |
| *EPHB4* | *ZSCAN26* | 0.35 | 4.89E-03 |
| *EPHB4* | *ZSWIM1* | 0.31 | 1.56E-02 |
| *EPHB4* | *ZSWIM3* | 0.31 | 1.49E-02 |
| *EPHB4* | *ZSWIM8* | 0.35 | 5.09E-03 |
| *EPHB4* | *ZXDC* | 0.36 | 4.61E-03 |
| *EPHB4* | *ZZEF1* | 0.34 | 7.62E-03 |

| **Table S6. Clinical characteristics of 233 ESCC patients in survival analysis** | | | | | | | |
| --- | --- | --- | --- | --- | --- | --- | --- |
| Sample ID | Sex | Age (year) | Smoking status | Drinking status | TNM stage | Survival status | Survival time (month) |
| LZ-51 | Male | 65 | Non-smoker | Non-drinker | II | Deceased | 30.9 |
| LZ-93 | Female | 65 | Non-smoker | Non-drinker | II | Deceased | 28.9 |
| LZ-136 | Male | 69 | Smoker | Drinker | III | Deceased | 22.6 |
| LZ-148 | Male | 60 | Non-smoker | Non-drinker | II | Deceased | 9.4 |
| LZ-189 | Male | 68 | Smoker | Drinker | III | Deceased | 17.9 |
| LZ-206 | Female | 70 | Non-smoker | Non-drinker | III | Deceased | 23.8 |
| LZ-263 | Male | 65 | Smoker | Drinker | II | Deceased | 19.6 |
| LZ-295 | Male | 63 | Smoker | Drinker | II | Deceased | 16.6 |
| LZ-317 | Male | 60 | Non-smoker | Non-drinker | III | Deceased | 21.3 |
| LZ-379 | Male | 67 | Smoker | Drinker | III | Deceased | 20.4 |
| LZ-402 | Male | 56 | Non-smoker | Non-drinker | III | Deceased | 17.8 |
| LZ-635 | Male | 69 | Non-smoker | Non-drinker | III | Deceased | 28.8 |
| LZ-643 | Male | 67 | Non-smoker | Non-drinker | I | Deceased | 8.6 |
| LZ-690 | Male | 63 | Non-smoker | Non-drinker | III | Deceased | 25.3 |
| LZ-732 | Female | 61 | Non-smoker | Non-drinker | I | Deceased | 16.6 |
| LZ-772 | Male | 63 | Smoker | Drinker | II | Deceased | 20.0 |
| LZ-828 | Male | 64 | Smoker | Drinker | II | Deceased | 12.9 |
| LZ-886 | Male | 70 | Smoker | Drinker | III | Deceased | 10.4 |
| LZ-901 | Male | 54 | Smoker | Drinker | III | Deceased | 11.3 |
| LZ-915 | Male | 67 | Smoker | Drinker | III | Deceased | 14.2 |
| LZ-950 | Male | 67 | Non-smoker | Non-drinker | III | Deceased | 14.8 |
| LZ-941 | Female | 69 | Non-smoker | Non-drinker | II | Deceased | 13.8 |
| LZ-968 | Male | 65 | Smoker | Drinker | III | Deceased | 17.6 |
| LZ-1043 | Female | 68 | Non-smoker | Non-drinker | III | Deceased | 7.5 |
| LZ-1039 | Female | 65 | Non-smoker | Non-drinker | I | Deceased | 11.6 |
| LZ-1057 | Male | 70 | Smoker | Drinker | II | Deceased | 17.5 |
| LZ-1237 | Male | 82 | Smoker | Drinker | III | Deceased | 16.3 |
| LZ-1326 | Male | 57 | Smoker | Drinker | III | Deceased | 17.9 |
| LZ-1394 | Male | 68 | Non-smoker | Non-drinker | III | Deceased | 10.7 |
| LZ-1368 | Female | 76 | Smoker | Drinker | III | Deceased | 9.0 |
| LZ-2 | Male | 62 | Smoker | Drinker | III | Deceased | 3.0 |
| LZ-12 | Male | 77 | Non-smoker | Non-drinker | III | Deceased | 12.4 |
| LZ-34 | Female | 66 | Non-smoker | Non-drinker | III | Deceased | 9.0 |
| LZ-74 | Male | 56 | Smoker | Drinker | III | Deceased | 8.3 |
| LZ-166 | Male | 63 | Smoker | Drinker | III | Deceased | 6.5 |
| LZ-322 | Male | 57 | Smoker | Drinker | III | Deceased | 4.4 |
| LZ-472 | Female | 64 | Non-smoker | Non-drinker | III | Deceased | 6.6 |
| LZ-629 | Female | 73 | Non-smoker | Non-drinker | II | Deceased | 7.7 |
| LZ-999 | Male | 75 | Smoker | Drinker | III | Deceased | 3.2 |
| LZ-1424 | Female | 65 | Non-smoker | Non-drinker | II | Deceased | 1.0 |
| LZ-1207 | Male | 72 | Smoker | Drinker | II | Deceased | 4.4 |
| LZ-104 | Female | 67 | Non-smoker | Non-drinker | II | Alive | 37.3 |
| LZ-134 | Male | 64 | Smoker | Non-drinker | III | Alive | 36.8 |
| LZ-208 | Female | 61 | Non-smoker | Non-drinker | II | Alive | 35.9 |
| LZ-221 | Male | 66 | Smoker | Drinker | II | Alive | 35.7 |
| LZ-248 | Male | 53 | Smoker | Drinker | III | Deceased | 17.7 |
| LZ-262 | Female | 70 | Smoker | Non-drinker | II | Deceased | 27.8 |
| LZ-319 | Male | 62 | Smoker | Non-drinker | I | Alive | 34.2 |
| LZ-336 | Male | 63 | Smoker | Non-drinker | III | Alive | 33.9 |
| LZ-357 | Female | 64 | Non-smoker | Drinker | I | Deceased | 33.2 |
| LZ-354 | Male | 75 | Smoker | Non-drinker | I | Alive | 33.7 |
| LZ-390 | Male | 72 | Non-smoker | Non-drinker | III | Deceased | 21.3 |
| LZ-386 | Female | 67 | Smoker | Non-drinker | III | Deceased | 23.9 |
| LZ-401 | Male | 58 | Smoker | Non-drinker | II | Alive | 32.9 |
| LZ-408 | Male | 55 | Smoker | Drinker | I | Alive | 32.8 |
| LZ-419 | Male | 75 | Smoker | Drinker | II | Deceased | 17.1 |
| LZ-413 | Female | 63 | Non-smoker | Non-drinker | II | Alive | 32.8 |
| LZ-437 | Female | 57 | Non-smoker | Non-drinker | II | Alive | 32.3 |
| LZ-436 | Male | 73 | Smoker | Drinker | III | Alive | 32.3 |
| LZ-549 | Female | 63 | Non-smoker | Non-drinker | III | Deceased | 18.8 |
| LZ-545 | Male | 63 | Non-smoker | Non-drinker | III | Alive | 30.5 |
| LZ-564 | Male | 59 | Smoker | Non-drinker | I | Deceased | 11.6 |
| LZ-581 | Male | 70 | Non-smoker | Drinker | II | Alive | 30.0 |
| LZ-580 | Female | 77 | Non-smoker | Non-drinker | II | Alive | 30.0 |
| LZ-620 | Male | 72 | Smoker | Drinker | III | Deceased | 20.0 |
| LZ-655 | Male | 69 | Smoker | Non-drinker | I | Alive | 28.7 |
| LZ-654 | Male | 64 | Smoker | Non-drinker | III | Deceased | 24.2 |
| LZ-682 | Female | 66 | Non-smoker | Non-drinker | III | Deceased | 21.8 |
| LZ-680 | Male | 55 | Non-smoker | Non-drinker | I | Alive | 28.5 |
| LZ-696 | Male | 73 | Smoker | Drinker | I | Deceased | 4.4 |
| LZ-753 | Male | 44 | Smoker | Drinker | III | Deceased | 3.6 |
| LZ-748 | Male | 71 | Smoker | Drinker | I | Deceased | 22.9 |
| LZ-747 | Male | 62 | Non-smoker | Non-drinker | I | Alive | 27.4 |
| LZ-767 | Male | 60 | Smoker | Non-drinker | I | Alive | 27.1 |
| LZ-781 | Female | 50 | Non-smoker | Non-drinker | III | Alive | 26.9 |
| LZ-846 | Male | 75 | Smoker | Non-drinker | I | Alive | 26.1 |
| LZ-868 | Male | 62 | Smoker | N | I | Alive | 25.9 |
| LZ-869 | Female | 64 | Non-smoker | Non-drinker | I | Alive | 25.9 |
| LZ-191 | Male | 67 | Smoker | Drinker | II | Deceased | 6.7 |
| LZ-538 | Male | 56 | Smoker | Drinker | II | Deceased | 6.8 |
| LZ-546 | Male | 76 | Non-smoker | Non-drinker | III | Deceased | 7.3 |
| LZ-603 | Male | 66 | Smoker | Drinker | II | Deceased | 7.7 |
| LZ-622 | Male | 47 | Non-smoker | Non-drinker | II | Deceased | 5.7 |
| LZ-108 | Female | 65 | Non-smoker | Non-drinker | II | Alive | 8.7 |
| LZ-105 | Male | 56 | Smoker | Non-drinker | II | Alive | 8.9 |
| LZ-123 | Male | 74 | Smoker | Non-drinker | II | Alive | 8.7 |
| LZ-116 | Male | 62 | Smoker | Drinker | II | Alive | 8.7 |
| LZ-197 | Male | 50 | Smoker | Non-drinker | II | Alive | 8.3 |
| LZ-203 | Female | 65 | Non-smoker | Non-drinker | II | Alive | 8.3 |
| LZ-210 | Male | 69 | Smoker | Non-drinker | III | Alive | 8.2 |
| LZ-222 | Male | 56 | Smoker | Non-drinker | III | Alive | 7.9 |
| LZ-227 | Male | 52 | Smoker | Drinker | II | Alive | 8.1 |
| LZ-220 | Male | 57 | Smoker | Non-drinker | II | Alive | 8.1 |
| LZ-226 | Female | 42 | Non-smoker | Non-drinker | II | Alive | 8.0 |
| LZ-228 | Male | 43 | Smoker | Non-drinker | II | Alive | 8.2 |
| LZ-237 | Male | 75 | Smoker | Non-drinker | II | Alive | 8.1 |
| LZ-283 | Male | 66 | Smoker | Non-drinker | II | Alive | 7.8 |
| LZ-323 | Male | 66 | Smoker | Non-drinker | III | Alive | 7.6 |
| LZ-338 | Male | 46 | Smoker | Non-drinker | III | Alive | 7.5 |
| LZ-339 | Male | 64 | Smoker | Drinker | III | Alive | 7.5 |
| LZ-340 | Female | 65 | Non-smoker | Non-drinker | II | Alive | 7.5 |
| LZ-343 | Male | 66 | Smoker | Non-drinker | I | Alive | 7.3 |
| LZ-353 | Male | 67 | Smoker | Drinker | II | Alive | 7.2 |
| LZ-342 | Male | 57 | Smoker | Drinker | III | Alive | 7.4 |
| LZ-352 | Female | 70 | Non-smoker | Non-drinker | III | Alive | 7.1 |
| LZ-362 | Male | 52 | Smoker | Non-drinker | II | Alive | 7.2 |
| LZ-361 | Male | 65 | Smoker | Drinker | II | Alive | 7.5 |
| LZ-359 | Female | 70 | Non-smoker | Non-drinker | II | Alive | 7.5 |
| LZ-385 | Male | 60 | Smoker | Drinker | III | Alive | 7.3 |
| LZ-427 | Male | 53 | Non-smoker | Drinker | III | Alive | 8.1 |
| LZ-415 | Male | 66 | Smoker | Non-drinker | II | Alive | 8.2 |
| LZ-424 | Male | 51 | Smoker | Drinker | I | Alive | 8.2 |
| LZ-378 | Male | 62 | Non-smoker | Non-drinker | II | Alive | 7.2 |
| LZ-370 | Female | 60 | Non-smoker | Non-drinker | III | Alive | 7.3 |
| LZ-552 | Female | 69 | Non-smoker | Non-drinker | II | Alive | 8.3 |
| LZ-555 | Male | 68 | Smoker | Drinker | III | Alive | 8.3 |
| LZ-575 | Female | 72 | Non-smoker | Non-drinker | II | Alive | 8.0 |
| LZ-623 | Male | 69 | Smoker | Non-drinker | III | Alive | 8.3 |
| LZ-619 | Male | 64 | Smoker | Non-drinker | III | Alive | 8.3 |
| LZ-638 | Female | 58 | Non-smoker | Non-drinker | III | Alive | 8.1 |
| LZ-656 | Male | 66 | Smoker | Drinker | III | Alive | 8.0 |
| LZ-678 | Female | 62 | Non-smoker | Non-drinker | I | Alive | 8.1 |
| LZ-784 | Male | 56 | Smoker | Non-drinker | III | Alive | 7.2 |
| LZ-779 | Female | 65 | Non-smoker | Non-drinker | I | Alive | 7.2 |
| LZ-795 | Male | 55 | Smoker | Drinker | III | Alive | 7.1 |
| LZ-806 | Female | 66 | Non-smoker | Non-drinker | III | Alive | 7.0 |
| LZ-84 | Male | 67 | Non-smoker | Non-drinker | II | Deceased | 16.8 |
| LZ-97 | Male | 62 | Smoker | Drinker | III | Deceased | 16.7 |
| LZ-448 | Male | 68 | Smoker | Drinker | II | Deceased | 11.4 |
| LZ-455 | Male | 63 | Smoker | Drinker | III | Deceased | 16.4 |
| LZ-476 | Male | 53 | Smoker | Drinker | III | Deceased | 17.2 |
| LZ-480 | Female | 63 | Non-smoker | Non-drinker | III | Deceased | 21.2 |
| LZ-511 | Male | 65 | Non-smoker | Drinker | II | Deceased | 13.7 |
| LZ-28 | Male | 68 | Smoker | Drinker | IV | Alive | 9.3 |
| LZ-48 | Male | 69 | Smoker | Non-drinker | I | Alive | 9.1 |
| LZ-55 | Female | 55 | Non-smoker | Non-drinker | II | Alive | 9.0 |
| LZ-69 | Male | 65 | Smoker | Drinker | II | Alive | 9.0 |
| LZ-124 | Female | 62 | Non-smoker | Non-drinker | II | Alive | 8.7 |
| LZ-274 | Male | 76 | Non-smoker | Non-drinker | II | Alive | 12.3 |
| ESCC-3 | Male | 61 | Smoker | Drinker | II | Deceased | 12.0 |
| ESCC-10 | Male | 47 | Smoker | Drinker | III | Deceased | 7.0 |
| ESCC-12 | Male | 53 | Smoker | Drinker | III | Deceased | 15.0 |
| ESCC-16 | Male | 55 | Smoker | Drinker | III | Deceased | 9.0 |
| ESCC-19 | Male | 53 | Smoker | Drinker | III | Deceased | 10.0 |
| ESCC-21 | Male | 55 | Smoker | Drinker | III | Deceased | 28.0 |
| ESCC-23 | Female | 60 | Non-smoker | Non-drinker | III | Deceased | 33.0 |
| ESCC-24 | Male | 43 | Smoker | Drinker | III | Deceased | 8.0 |
| ESCC-26 | Male | 56 | Smoker | Drinker | II | Deceased | 6.0 |
| ESCC-27 | Male | 54 | Smoker | Non-drinker | III | Deceased | 9.0 |
| ESCC-35 | Male | 53 | Smoker | Drinker | III | Deceased | 32.0 |
| ESCC-36 | Male | 65 | Smoker | Drinker | II | Deceased | 6.0 |
| ESCC-39 | Male | 72 | Non-smoker | Drinker | II | Deceased | 6.0 |
| ESCC-42 | Male | 62 | Smoker | Drinker | III | Deceased | 28.0 |
| ESCC-48 | Male | 55 | Smoker | Drinker | III | Deceased | 23.0 |
| ESCC-50 | Male | 53 | Smoker | Drinker | II | Deceased | 11.0 |
| ESCC-54 | Male | 44 | Smoker | Drinker | III | Deceased | 10.0 |
| ESCC-55 | Male | 61 | Smoker | Non-drinker | IV | Deceased | 21.0 |
| ESCC-57 | Male | 68 | Smoker | Drinker | II | Deceased | 12.0 |
| ESCC-58 | Male | 47 | Smoker | Drinker | III | Deceased | 14.0 |
| ESCC-60 | Male | 54 | Smoker | Drinker | II | Alive | 18.0 |
| ESCC-61 | Male | 58 | Smoker | Drinker | III | Deceased | 9.0 |
| ESCC-62 | Male | 75 | Smoker | Drinker | III | Deceased | 33.0 |
| ESCC-64 | Male | 68 | Smoker | Drinker | III | Deceased | 21.0 |
| ESCC-65 | Male | 54 | Smoker | Drinker | III | Deceased | 10.0 |
| ESCC-125 | Male | 63 | Smoker | Drinker | III | Alive | 98.0 |
| ESCC-130 | Male | 58 | Smoker | Drinker | III | Deceased | 18.0 |
| ESCC-131 | Male | 50 | Non-smoker | Non-drinker | II | Alive | 96.0 |
| ESCC-132 | Male | 57 | Smoker | Drinker | III | Alive | 64.0 |
| ESCC-134 | Male | 59 | Smoker | Drinker | III | Deceased | 19.0 |
| ESCC-138 | Male | 54 | Smoker | Drinker | II | Alive | 95.0 |
| ESCC-140 | Male | 77 | Smoker | Drinker | III | Deceased | 6.0 |
| ESCC-142 | Male | 66 | Smoker | Drinker | II | Deceased | 11.0 |
| ESCC-143 | Male | 63 | Smoker | Non-drinker | III | Alive | 94.0 |
| ESCC-144 | Female | 64 | Non-smoker | Non-drinker | II | Deceased | 32.0 |
| ESCC-145 | Female | 45 | Non-smoker | Non-drinker | III | Alive | 94.0 |
| ESCC-149 | Male | 62 | Smoker | Drinker | III | Deceased | 9.0 |
| ESCC-150 | Male | 68 | Smoker | Drinker | III | Alive | 94.0 |
| ESCC-152 | Male | 62 | Smoker | Drinker | II | Deceased | 34.0 |
| ESCC-156 | Male | 54 | Smoker | Drinker | III | Deceased | 14.0 |
| ESCC-158 | Male | 63 | Smoker | Drinker | III | Deceased | 6.0 |
| ESCC-161 | Female | 67 | Non-smoker | Non-drinker | III | Alive | 92.0 |
| ESCC-162 | Male | 66 | Smoker | Non-drinker | II | Deceased | 11.0 |
| ESCC-168 | Male | 68 | Smoker | Drinker | II | Alive | 61.0 |
| ESCC-169 | Male | 68 | Non-smoker | Non-drinker | III | Deceased | 6.0 |
| ESCC-170 | Male | 60 | Smoker | Drinker | II | Alive | 58.0 |
| ESCC-171 | Male | 57 | Smoker | Drinker | III | Alive | 57.0 |
| ESCC-172 | Male | 70 | Non-smoker | Drinker | III | Deceased | 11.0 |
| ESCC-173 | Female | 62 | Non-smoker | Non-drinker | II | Alive | 89.0 |
| ESCC-175 | Male | 61 | Smoker | Drinker | III | Deceased | 6.0 |
| ESCC-178 | Male | 55 | Smoker | Non-drinker | III | Deceased | 7.0 |
| ESCC-179 | Male | 63 | Smoker | Drinker | III | Deceased | 51.0 |
| ESCC-182 | Male | 65 | Smoker | Drinker | III | Deceased | 11.0 |
| ESCC-185 | Male | 59 | Smoker | Drinker | III | Deceased | 16.0 |
| ESCC-191 | Female | 48 | Non-smoker | Non-drinker | III | Alive | 81.0 |
| ESCC-196 | Female | 63 | Non-smoker | Non-drinker | III | Deceased | 12.0 |
| ESCC-198 | Male | 52 | Smoker | Drinker | II | Deceased | 23.0 |
| ESCC-199 | Male | 56 | Smoker | Drinker | III | Deceased | 13.0 |
| ESCC-201 | Male | 58 | Smoker | Drinker | III | Alive | 87.0 |
| ESCC-206 | Male | 56 | Smoker | Drinker | III | Deceased | 9.0 |
| ESCC-208 | Male | 53 | Smoker | Drinker | III | Deceased | 7.0 |
| ESCC-210 | Male | 51 | Smoker | Drinker | II | Deceased | 22.0 |
| ESCC-213 | Male | 60 | Smoker | Drinker | III | Alive | 85.0 |
| ESCC-215 | Male | 61 | Smoker | Drinker | II | Alive | 84.0 |
| ESCC-220 | Male | 58 | Smoker | Drinker | III | Deceased | 6.0 |
| ESCC-222 | Male | 56 | Smoker | Drinker | III | Deceased | 26.0 |
| ESCC-223 | Male | 76 | Non-smoker | Drinker | III | Deceased | 11.0 |
| ESCC-224 | Female | 58 | Non-smoker | Drinker | III | Deceased | 12.0 |
| ESCC-225 | Male | 67 | Non-smoker | Drinker | II | Deceased | 6.0 |
| ESCC-234 | Male | 64 | Smoker | Drinker | III | Deceased | 16.0 |
| ESCC-235 | Female | 65 | Non-smoker | Non-drinker | III | Deceased | 7.0 |
| ESCC-239 | Male | 62 | Smoker | Drinker | III | Deceased | 7.0 |
| ESCC-240 | Male | 66 | Smoker | Drinker | III | Deceased | 12.0 |
| ESCC-243 | Male | 45 | Smoker | Drinker | III | Deceased | 9.0 |
| ESCC-245 | Male | 69 | Smoker | Drinker | III | Deceased | 9.0 |
| ESCC-246 | Male | 63 | Smoker | Drinker | III | Deceased | 8.0 |
| ESCC-249 | Male | 64 | Smoker | Drinker | III | Deceased | 7.0 |
| ESCC-E3 | Male | 75 | Smoker | Non-drinker | II | Alive | 60.0 |
| ESCC-E11 | Female | 56 | Non-smoker | Non-drinker | II | Alive | 59.0 |
| ESCC-E17 | Male | 62 | Smoker | Drinker | II | Alive | 28.0 |
| ESCC-E24 | Male | 60 | Smoker | Drinker | III | Alive | 58.0 |
| ESCC-E25 | Female | 72 | Non-smoker | Non-drinker | II | Alive | 26.0 |
| ESCC-E26 | Male | 49 | Smoker | Drinker | II | Alive | 58.0 |
| ESCC-E30 | Male | 54 | Smoker | Drinker | III | Deceased | 21.0 |
| ESCC-E33 | Male | 59 | Smoker | Drinker | III | Deceased | 7.0 |
| ESCC-E34 | Male | 64 | Smoker | Drinker | III | Alive | 58.0 |
| ESCC-E45 | Male | 61 | Smoker | Drinker | III | Deceased | 14.0 |
| ESCC-E47 | Male | 64 | Smoker | Drinker | III | Deceased | 12.0 |
| ESCC-E50 | Male | 67 | Smoker | Drinker | III | Alive | 24.0 |
| ESCC-E71 | Male | 57 | Smoker | Drinker | III | Alive | 53.0 |
| ESCC-E74 | Male | 71 | Smoker | Non-drinker | II | Alive | 53.0 |
| ESCC-E75 | Male | 64 | Smoker | Non-drinker | III | Alive | 53.0 |
| ESCC-E78 | Male | 66 | Smoker | Drinker | III | Alive | 52.0 |
| ESCC-E79 | Male | 62 | Smoker | Drinker | III | Alive | 52.0 |

| **Table S7. Correlation data of 46 overlapped transcription factors (TFs) with *EFNB1* and *EPHB4* in scRNA-seq data (GSE160269)** | | | | |
| --- | --- | --- | --- | --- |
| *TFs* | *EFNB1*-r | *EFNB1*-*P* value | *EPHB4*-r | *EPHB4*-*P* value |
| *TP63* | 0.43 | 3.28E-04 | 0.33 | 8.44E-03 |
| *ZNF263* | 0.38 | 1.69E-03 | 0.37 | 2.75E-03 |
| *YY1* | 0.38 | 1.72E-03 | 0.28 | 2.73E-02 |
| *CREB1* | 0.35 | 4.17E-03 | 0.49 | 4.53E-05 |
| *ZBTB7A* | 0.30 | 1.61E-02 | 0.28 | 2.73E-02 |
| *MAX* | 0.28 | 2.44E-02 | 0.16 | 2.07E-01 |
| *FOS* | 0.25 | 5.09E-02 | 0.28 | 2.38E-02 |
| *MAZ* | 0.23 | 7.10E-02 | 0.46 | 1.35E-04 |
| *ERG* | 0.22 | 7.72E-02 | 0.05 | 7.22E-01 |
| *NFYB* | 0.20 | 1.10E-01 | 0.18 | 1.49E-01 |
| *KLF5* | 0.19 | 1.29E-01 | 0.15 | 2.39E-01 |
| *SP4* | 0.19 | 1.31E-01 | 0.31 | 1.24E-02 |
| *E2F6* | 0.18 | 1.46E-01 | 0.26 | 3.77E-02 |
| *TCF3* | 0.17 | 1.92E-01 | 0.25 | 4.21E-02 |
| *TFAP2C* | 0.16 | 1.96E-01 | 0.32 | 9.60E-03 |
| *ELF1* | 0.15 | 2.22E-01 | 0.02 | 8.51E-01 |
| *E2F1* | 0.13 | 3.10E-01 | 0.26 | 3.90E-02 |
| *KLF9* | 0.13 | 3.20E-01 | 0.15 | 2.45E-01 |
| *IRF1* | 0.10 | 4.23E-01 | 0.25 | 4.58E-02 |
| *FLI1* | 0.09 | 4.59E-01 | 0.23 | 6.45E-02 |
| *CEBPA* | 0.09 | 4.81E-01 | 0.17 | 1.85E-01 |
| *BHLHE40* | 0.07 | 5.80E-01 | -0.22 | 8.10E-02 |
| *E2F4* | 0.05 | 7.09E-01 | -0.04 | 7.83E-01 |
| *SRF* | 0.04 | 7.31E-01 | 0.13 | 2.88E-01 |
| *NFYA* | 0.04 | 7.74E-01 | 0.21 | 9.81E-02 |
| *ARNT* | 0.04 | 7.75E-01 | 0.23 | 6.61E-02 |
| *SP1* | 0.03 | 8.15E-01 | 0.16 | 1.93E-01 |
| *CTCF* | 0.01 | 9.58E-01 | 0.16 | 2.02E-01 |
| *TP53* | 0.00 | 9.91E-01 | 0.28 | 2.79E-02 |
| *TAL1* | 0.00 | 9.82E-01 | 0.03 | 8.23E-01 |
| *HNF4G* | -0.03 | 8.40E-01 | 0.06 | 6.61E-01 |
| *TCF12* | -0.04 | 7.67E-01 | 0.29 | 1.84E-02 |
| *STAT1* | -0.06 | 6.32E-01 | -0.04 | 7.24E-01 |
| *FOXP1* | -0.06 | 6.27E-01 | 0.25 | 5.07E-02 |
| *FOXA1* | -0.07 | 5.89E-01 | 0.03 | 7.88E-01 |
| *MYB* | -0.11 | 3.79E-01 | 0.34 | 5.68E-03 |
| *SPI1* | -0.12 | 3.65E-01 | 0.10 | 4.51E-01 |
| *RUNX2* | -0.13 | 3.10E-01 | -0.05 | 6.83E-01 |
| *RUNX1* | -0.15 | 2.23E-01 | 0.22 | 7.77E-02 |
| *NR2F2* | -0.17 | 1.78E-01 | 0.27 | 3.20E-02 |
| *ETS1* | -0.17 | 1.67E-01 | -0.14 | 2.85E-01 |
| *KLF1* | / | / | / | / |
| *OTX2* | / | / | / | / |
| *HNF4A* | / | / | / | / |
| *FOXA2* | / | / | / | / |
| *GATA1* | / | / | / | / |

| **Supplementary Table 8. Sequences of siRNAs, shRNAs and primers** | |
| --- | --- |
| siRNA/shRNA/Primer | Sequence (5' → 3') |
| Human-si*EFNB1* #1 | UACUACUGAAGCUACGCAATT |
| Human-si*EFNB1* #2 | CGACAUCAUCAUUCCCUUATT |
| Human-si*EPHB4* #1 | AUGGGAGAGAAGCAGAAUATT |
| Human-si*EPHB4* #2 | AUCUGAAGUGGGUGACAUUTT |
| Human-si*CREB1* #1 | UCGAUAAAUCUAACAGUUATT |
| Human-si*CREB1* #2 | AUUAGCCCAGGUAUCUAUGTT |
| Human-si*ZNF263* #1 | AGCCAAAGAAACUCCAUUUTT |
| Human-si*ZNF263* #2 | UGUAAAGGAGAGGGCAUUATT |
| Human-si*TP63* #1 | UAGUCAUUUGAUUCGAGUATT |
| Human-si*TP63* #2 | GUGGAAUGACUUCAACUUUTT |
| Mouse-si*Efnb1* #1 | GCACUAUGAAGAUCGUUAUTT |
| Mouse-si*Efnb1* #2 | CUAAGUUCCUAAGUGGGAA TT |
| Mouse-*siEphb4* #1 | GAUCUGAAAUGGGUGACUUTT |
| Mouse-si*Ephb4* #2 | GUUAUGAUCCUCACGGAAUTT |
| Mouse-si*Creb1* #1 | CAAGAGAAUGUCGUAGAAATT |
| Mouse-si*Creb1* #2 | UGAUGGACAGCAGAUUCUATT |
| Mouse-si*Znf263* #1 | CGGAGUUCACAUCUUGUUATT |
| Mouse-si*Znf263* #2 | UCUAUCAAGGUAUAUGGUATT |
| Mouse-si*Trp63* #1 | CCCUCAGCACACGAUCGAATT |
| Mouse-si*Trp63* #2 | GCAGCAUUGUCAGUUUCUUTT |
| Human-shControl | TTCTCCGAACGTGTCACGT |
| Human-sh*EFNB1* #1 | CTTACGGACTACAGAGAACAA |
| Human-sh*EFNB1* #2 | CCAGAGCAGGAAATACGCTTT |
| Human-sh*EPHB4* #1 | GCGCACCTACGAAGTGTGTGA |
| Human-sh*EPHB4* #2 | GCACATGAAGTCCCAGGCCAA |
| *ZNF263_*primer_F | CTTGCGCTTCAGACGGTTC |
| *ZNF263 _*primer_R | CCCAAGCTCTCTCTGCATATCC |
| *CREB1*_primer_F | ATTCACAGGAGTCAGTGGATAGT |
| *CREB1*_primer_R | CACCGTTACAGTGGTGATGG |
| *TP63*_primer_F | GGACCAGCAGATTCAGAACGG |
| *TP63*_primer_R | AGGACACGTCGAAACTGTGC |
| *Zfp263*_primer_F | GCCTCCCATCTGCGATTTC |
| *Zfp263*_primer_R | CCTGGGGAAGGATAGTCAAGAA |
| *Creb1*_primer_F | AGCAGCTCATGCAACATCATC |
| *Creb1*_primer_R | AGTCCTTACAGGAAGACTGAACT |
| *Trp63*_primer_F | CCACAGTACACGAACCTGGG |
| *Trp63*_primer_R | TGGAAGGACACATCGAAGCTG |
| ChIP-*EFNB1*_primer_F | CTAGCATGCACTCCAGGGAT |
| ChIP-*EFNB1*_primer_R | GCATTGGGGTGTAAGGAAGGA |
| ChIP-EPHB4_primer_F | GTCGGGAAGTTGAGAGAAAGTT |
| ChIP-EPHB4_primer_R | GTGTGTGTTTTCTCTGCTCCCT |
| m-ephb4-f | GGAAACGGCGGATCTGAAATG |
| m-ephb4-r | TGGACGCTTCATGTCGCAC |
| m-efnb1-f | TGTGGCTATGGTCGTGCTG |
| m-efnb1-r | CCAAGCCCTTCCCACTTAGG |
| h-EFNB1-f | TGGAGCCCGTATCCTGGAG |
| h-EFNB1-r | TTGGGGTCGAGAACTGTGCTA |
| h-EPHB4-f | CCACCGGGAAGGTGAATGTC |
| h-EPHB4-r | CTGGGCGCACTTTTTGTAGAA |
| *CCND1*_primer_F | GCTGCGAAGTGGAAACCATC |
| *CCND1*_primer_R | CCTCCTTCTGCACACATTTGAA |
| *CCND3*_primer_F | TACCCGCCATCCATGATCG |
| *CCND3*_primer_R | AGGCAGTCCACTTCAGTGC |
| *CDC40*_primer_F | TGGCCGCTTCCTATGGTTC |
| *CDC40*_primer_R | GCGATTTAGTCAAGTGCATGAGG |
| *CDK2*_primer_F | CCAGGAGTTACTTCTATGCCTGA |
| *CDK2*_primer_R | TTCATCCAGGGGAGGTACAAC |
| *CEP250*_primer_F | AGCAACCCTTGTGAGGAAGC |
| *CEP250*_primer_R | TTTTCCATGTGCAGTCGAAGC |
| *POLR2A*_primer_F | GGGTGGCATCAAATACCCAGA |
| *POLR2A*_primer_R | AGACACAGCGCAAAACTTTCA |
| *RAD21*_primer_F | GGATAAGAAGCTAACCAAAGCCC |
| *RAD21*_primer_R | CTCCCAGTAAGAGATGTCCTGAT |
| *RAE1*_primer_F | CCTTGCCGGGGAACTTTCTTA |
| *RAE1*_primer_R | CTGGGCTTTTGGAATGGTCTG |
| *RBBP8*_primer_F | CAGGAACGAATCTTAGATGCACA |
| *RBBP8*_primer_R | GCCTGCTCTTAACCGATCTTCT |
| *RCC2*_primer_F | AAGGAGCGCGTCAAACTTGAA |
| *RCC2*_primer_R | GCTTGCTGTTTAGGCACTTCTT |
| *TOPBP1*_primer_F | TGTGACCCTTTTAGTGGCGTT |
| *TOPBP1*_primer_R | CTCTTGGGACACATCGCTGG |
| *UHRF1*_primer_F | GCCATACCCTCTTCGACTACG |
| *UHRF1*_primer_R | GCCCCAATTCCGTCTCATCC |
| *CDH1*_primer_F | CGAGAGCTACACGTTCACGG |
| *CDH1*_primer_R | GGGTGTCGAGGGAAAAATAGG |
| *DST*_primer_F | CTACCAGCACTCGAACCAGTC |
| *DST*_primer_R | GCCGAAGCTAATGCAAGAGTTG |
| *ITGAV*_primer_F | ATCTGTGAGGTCGAAACAGGA |
| *ITGAV*_primer_R | TGGAGCATACTCAACAGTCTTTG |
| *ITGA6*_primer_F | ATGCACGCGGATCGAGTTT |
| *ITGA6*_primer_R | TTCCTGCTTCGTATTAACATGCT |
| *ITGB1*_primer_F | CCTACTTCTGCACGATGTGATG |
| *ITGB1*_primer_R | CCTTTGCTACGGTTGGTTACATT |
| *SNAI1*_primer_F | TCGGAAGCCTAACTACAGCGA |
| *SNAI1*_primer_R | AGATGAGCATTGGCAGCGAG |
| *SNAI2*_primer_F | TGTGACAAGGAATATGTGAGCC |
| *SNAI2*_primer_R | TGAGCCCTCAGATTTGACCTG |
| *TNC*_primer_F | TCCCAGTGTTCGGTGGATCT |
| *TNC*_primer_R | TTGATGCGATGTGTGAAGACA |
| *VIM*_primer_F | AGTCCACTGAGTACCGGAGAC |
| *VIM*_primer_R | CATTTCACGCATCTGGCGTTC |
| *ZEB1*_primer_F | GATGATGAATGCGAGTCAGATGC |
| *ZEB1*_primer_R | ACAGCAGTGTCTTGTTGTTGT |
